# Practical Implications of the Non-Linear Relationship between the Test Positivity Rate and Malaria Incidence

**DOI:** 10.1371/journal.pone.0152410

**Published:** 2016-03-28

**Authors:** Ross M. Boyce, Raquel Reyes, Michael Matte, Moses Ntaro, Edgar Mulogo, Feng-Chang Lin, Mark J. Siedner

**Affiliations:** 1 Division of Infectious Diseases, University of North Carolina at Chapel Hill, Chapel Hill, United States of America; 2 Division of General Medicine, University of North Carolina at Chapel Hill, Chapel Hill, United States of America; 3 Department of Community Health, Mbarara University of Science & Technology, Mbarara, Uganda; 4 Department of Biostatistics, Gillings School of Global Public Health, University of North Carolina at Chapel Hill, Chapel Hill, United States of America; 5 Department of Medicine, Harvard Medical School, and Massachusetts General Hospital, Boston, United States of America; Institute of Tropical Medicine, JAPAN

## Abstract

**Background:**

The test positivity rate (TPR), defined as the number of laboratory-confirmed malaria tests per 100 suspected cases examined, is widely used by malaria surveillance programs as one of several key indicators of temporal trends in malaria incidence. However, there have been few studies using empiric data to examine the quantitative nature of this relationship.

**Methods:**

To characterize the relationship between the test positivity rate and the incidence of malaria, we fit regression models using the confirmed malaria case rate as the outcome of interest and TPR as the predictor of interest. We varied the relationship between the two by alternating linear and polynomial terms for TPR, and compared the goodness of fit of each model.

**Results:**

A total of 7,668 encounters for malaria diagnostic testing were recorded over the study period within a catchment area of 25,617 persons. The semi-annual TPR ranged from 4.5% to 59% and the case rates ranged from 0.5 to 560 per 1,000 persons. The best fitting model was an exponential growth model (R^2^ = 0.80, AIC = 637). At low transmission levels (TPR<10%), the correlation between TPR and CMCR was poor, with large reductions in the TPR, for example from 10% to 1%, was associated with a minimal change in the CMCR (3.9 to 1.7 cases per 1,000 persons). At higher transmission levels, the exponential relationship made relatively small changes in TPR suggestive of sizeable change in estimated malaria incidence, suggesting that TPR remains a valuable surveillance indicator in such settings.

**Conclusions:**

The TPR and the confirmed malaria case rate have a non-linear relationship, which is likely to have important implications for malaria surveillance programs, especially at the extremes of transmission.

## Background

Valid data describing the number and distribution of malaria cases is critical for the design and implementation of malaria control programs [1–3]. Timely and accurate measurement of disease incidence over time is, however, challenging in malaria-endemic countries. With a high frequency of malaria cases and limited resources for maintaining an extensive recording and reporting system, many national malaria surveillance programs rely on the reporting and use of aggregate data by district and higher administrative levels [3]. Thus, trends in malaria incidence are often estimated from the total number of cases reported through routine health management information systems (HMIS). While this approach is relatively simple and inexpensive [4]. it is susceptible to variations in local health seeking behaviors, diagnostic test utilization, and the completeness of record keeping and reporting [5, 6].

The test positivity rate (TPR), defined as the number of laboratory-confirmed malaria tests per 100 suspected cases examined, is one of several indicators used for estimating temporal trends in malaria incidence [3]. The positivity rate may be derived from either rapid diagnostic test positivity rates (RDT PR) or microscopy (SPR). Advantages of the TPR method are that it only considers laboratory confirmed cases of malaria and it is relatively easy to monitor at peripheral health facilities. A disadvantage of TPR is that it is potentially susceptible to bias from changes over time in diagnostic testing methods, health care-seeking behavior, and the incidence of non-malarial febrile illnesses, rather than changes in malaria incidence [7].

The TPR is one of the WHO’s ten core malaria indicators for malaria surveillance in the control phase [3]. The measure has been used to define malaria endemicity [8, 9]. is widely utilized to assess temporal trends in malaria incidence [5] and to evaluate the impact of malaria control interventions [10–12]. A TPR <5% during the peak malaria season also serves as an important milestone for countries moving towards malaria elimination. The WHO currently recommends that countries may consider entering a pre-elimination phase if the TPR during the peak malaria season is <5% or the incidence falls below 5 cases per 1,000, at which time changes in routine surveillance systems may be required [13, 14].

Despite the widespread adoption of the TPR as a practical measure of assessing temporal trends in malaria incidence, there have been few studies examining the practical implications and limitations of this method using empiric data [6, 7, 15, 16]. To better characterize the relationship between TPR and trends in the incidence of malaria we compared village-level TPR and the confirmed malaria case rate (CMCR), defined as the number of laboratory-confirmed malaria cases per 1,000 people, collected over a two-year period among patients presenting to a rural health center in Western Uganda.

## Methods

The Bugoye Level III Health Center (BHC), in the Kasese District of Western Uganda (0° 18’ North, 30° 5’ East), functions as the primary health center for the Bugoye sub-county, serving a rural population of approximately 50,000 residents. Like much of Uganda, the climate in Bugoye permits stable, year-round malaria transmission. There are traditionally annual peaks following the end of the rainy seasons in July and January, when test positivity rates can reach 50%, and low-transmission seasons in March and November when test positivity rates are typically in the 10–20% range [17]. RDTs were first introduced at BHC in 2011 and have become the primary method of malaria diagnosis [18].

Of the more than 40 villages in the health center catchment area, we purposefully selected 15 villages from the Bugoye and Mubuku Sub-Counties. These villages were chosen because of (1) their close proximity to the health center and (2) the absence of other health facilities providing malaria case management. Our intent was to select villages where the vast majority of suspected malaria cases present to BHC so that CMCR would closely approximate malaria incidence. Malaria diagnostic test results, defined as either microscopy or RDT, were obtained from health center laboratory registries. We did not include any cases that did not have available diagnostic testing results. We retrospectively examined results for a period of 24 months beginning in May 2012. For each record, we abstracted patient age, gender, village of residence, and diagnostic test result.

Our primary predictor of interest was TPR, which was calculated as the number of positive diagnostic tests as a proportion of the total tests performed among residents of each of the specified villages. In sensitivity analyses, we also separately calculated the SPR, using only microscopy results, and the RDT PR, using only RDT results. Our primary outcome of interest was the CMCR, which we calculated for each of the 15 selected villages in six-month intervals using laboratory-confirmed malaria episodes from BHC records to estimate village specific malaria case totals and 2014 census data to esimtate village population size. We chose a six-month period of observation because each six-month period in this region captures both a single dry and single rainy season. In sensitivity analyses, we altered the outcome, substituting periods of 3-month and 24-months for the 6-month observation period. The results of the sensitivity analyses were reported in terms of exponentiated coefficients, which are the change in odds in the multiplicative scale for a unit increase in the corresponding predictor variable holding other variables at certain value.

Data were entered into Microsoft Excel (Redmond, WA) and analyzed with Stata 12.1 (College Station, TX). We first summarized patient demographics and malaria seasonality by village. Next, we graphically depicted the relationship between the TPR and the CMCR by village. We then developed regression models, varying the relationship between TPR and CMCR using linear, logistic, polynomial, and exponential terms, and compared the goodness of fit between models using the Adjusted R^2^ and Akaike Information Criteria (AIC) [19]. We used the selected model to estimate the relationship between CMCR and TPR. We performed regression diagnostics by calculating Cook’s D [20] for each village to assess for high leverage and/or large residuals. Points with Cook’s D greater than 1 were excluded in secondary analyses.

Ethical approval for study procedures and data collection was provided by the institutional review boards of Partners Healthcare and the Mbarara University of Science and Technology. Written informed consent was not required by the ethical review committees due to the routine, de-identified nature of the data.

## Results

A total of 7,668 individual encounters for malaria diagnostic testing were recorded over the study period (S1 Table). Approximately 30% of patients presenting for testing were under the age of five years. The number of individuals tested as a percentage of the population ranged from 1.9% in Kisamba to 108% in Izinga. There were 6,811 RDTs and 1,081 thin/thick smears performed, with 224 (2.9%) individuals undergoing both diagnostic procedures. Of the RDTs, 2,335 (34.3%) were positive for malaria, while 500 (46.3%) of the smears were positive for malaria.

The overall CMCR in the selected villages was 59/1,000 persons per year. The lowest CMCRs were seen in the villages of Kisamba (1.3/1,000) and Kihindi (4.1/1,000), both of which are located on the mountain ridges with average elevations >1,600m. The highest six-month CMCR was seen in the village of Izinga (273.7/1,000), which lies between two major rivers at an elevation approximately 400m lower than low-incidence villages. Additional results, stratified by village, are shown in Table 1.

**Table 1:** Summary of diagnostic testing results by village over the 24-month study period.

Village	Population	Tested (n, %)	Age <5 (n, %)	Rainy Season (n, %)	TPR (%)	Total RDTs	RDT PR (%)	Total Slides	SPR (%)	CMCR*
Bugoye	1,549	1,499 (96.8)	341 (22.6)	801 (53.1)	29.2	1343	28.5	201	38.8	135.7
Bunyangoni	1,644	602 (36.6)	229 (37.8)	307 (50.7)	32.1	525	20.5	89	47.2	56.4
Ihani	714	137 (19.2)	36 (26.3)	73 (53.3)	40.2	123	41.5	15	26.7	37.0
Izinga	833	900 (108.0)	254 (28.0)	489 (53.9)	52.8	787	53.6	147	55.1	273.7
Kanyanamigho	1,808	908 (50.2)	257 (28.2)	402 (44.2)	42.4	786	42.0	142	43.7	102.2
Katumba	1,110	130 (11.7)	46 (35.4)	73 (56.2)	20.8	112	18.8	19	31.6	11.7
Kibirizi	1,113	71 (6.4)	19 (26.4)	33 (45.8)	22.5	64	21.9	10	30.0	6.9
Kihindi	936	44 (4.7)	24 (53.3)	23 (51.1)	18.2	35	8.6	10	50.0	4.1
Kikokera	588	200 (34.0)	47 (23.5)	88 (44.0)	36.5	175	37.7	31	32.3	59.6
Kisamba I & II	3,280	62 (18.9)	12 (19.4)	36 (58.1)	14.5	60	13.3	2	50.0	1.3
Maghoma	2,688	108 (4.0)	48 (44.4)	61 (56.5)	16.7	99	14.1	12	33.3	3.2
Muhambo	1,272	176 (13.8)	72 (40.5)	108 (60.7)	18.2	169	18.3	12	25.0	12.1
Muramba I & II	3,576	1,303 (36.4)	381 (29.2)	701 (53.6)	37.7	1186	37.1	162	51.9	65.9
Ndughutu E & W	2,538	815 (32.1)	274 (33.6)	432 (52.9)	31.4	723	30.0	118	46.6	48.4
Rwaking A & B	1,968	713 (36.2)	222 (31.2)	357 (50.1)	38.7	624	36.5	111	55.9	67.3
**TOTAL**	**25,617**	**7,763**	**2,262 (29.5)**	**3,984 (52.0)**	**35.9**	**6811**	**34.3**	**1081**	**46.3**	**59.0**

TPR = test positivity rate, RDT = rapid diagnostic test, RDT PR = rapid diagnostic test positivity rate, SPR = slide positivity rate;

*CMCR measured in annual number of malaria cases per 1,000 population.

**Fig 1:**
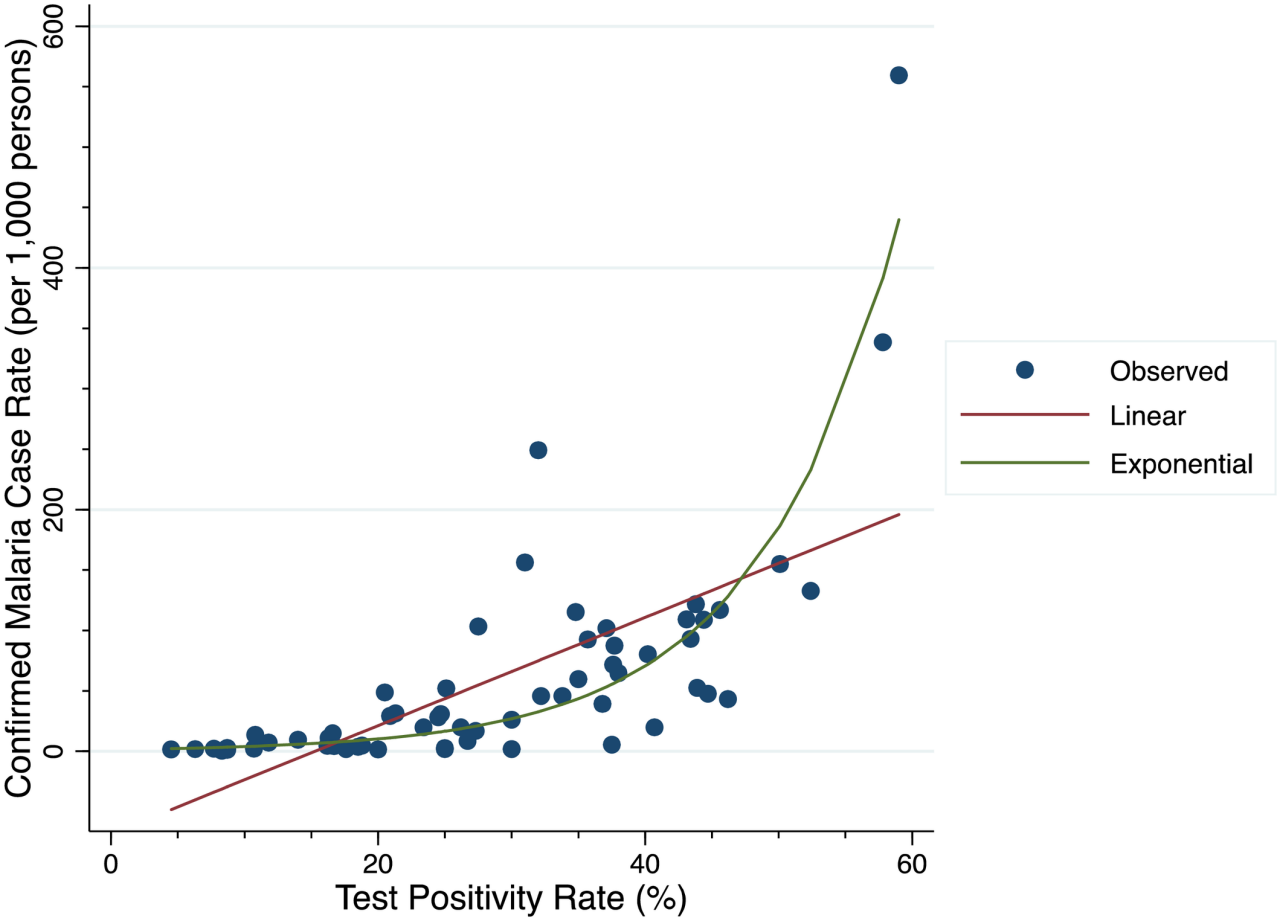
Linear (red) and exponential (green) models of the relationship between confirmed malaria case rate and test positivity rate at 6-month measurement intervals.

Fig 1 depicts the relationship between the TPR and CMCR. An exponential growth curve of the six-month CMCR was the best fitting model (Adjusted R^2^ = 0.80, AIC 637) compared to other models (Table 2). We found the linear model to poorly fit the data, with an adjusted R^2^ of 0.44 and AIC of 680 compared to the exponential model (Fig 1).

**Table 2:** Regression models of diagnostic test positivity rates (TPR) as predictors of confirmed malaria case rate (CMCR).

Model	Formula	Adjusted R^2^	AIC
Linear	*y = b*_*0*_ *+ b*_*1*_*(x)*	0.44	680
Logarithmic	*y = b*_*0*_ *+ (b*_*1*_ * ln(x))	0.25	694
Linear Spline at TPR 35%	*Y = bo + b1(x*_*x<35%*_*) + b2(x*_*x>35%*_*)*	0.56	666
Quadratic	*y = b*_*0*_ *+ b*_*1*_*x + b*_*2*_*x*^*2*^	0.58	661
Cubic	*y = b*_*0*_ *+ b*_*1*_*x + b*_*2*_*x*^*2*^ *+ b*_*3*_*x*^*3*^	0.69	645
Power	*y = b*_*0*_ * (x^*b1*^*)*	0.77	646
**Exponential**	***y = b***_***0***_ * b_***1***_^***x***^	**0.80**	**637**

AIC = Akaike Information Criteria.

Using the linear model, we would estimate negligible malaria transmission at TPR less than 15%, but a constant rate of increase of approximately 22 cases per 1,000 for each 5% increase in TPR above that level. In contrast, the exponential model estimates that an increase in TPR from 5% to 10% corresponds to a predicted increase in CMCR of only 1.5 cases per 1,000 (2.4 versus 3.9 cases per 1,000).

Of note, using the exponential model, a similar 5% absolute change in TPR from 30 to 35% corresponds to a predicted increase in CMCR of 27 to 44 cases per 1,000 (Table 3). In other words, the relationship between TPR and CMCR is non-linear, such that TPR below 10% signify relatively small changes in malaria case rates of less than 5 per 1,000, but relatively small changes in TPR above 10% correlate to larger changes in estimated malaria incidence over time.

**Table 3:** Comparison of predicted confirmed malaria case rates (CMCR) at specified test positivity rates (TPR) using both linear and exponential growth regression models.

TPR (%)	Linear Model Predicted CMCR	Exponential Model Predicted CMCR
5	< 0	2.4
10	< 0	3.9
20	21.2	10.3
40	110.8	70.5

While not the best-fitting model, a spline model with a knot set at 35% does highlight the non-linear nature of the data. The spline model predicts a trend towards a significant increase of 1.38 cases per 1,000 (95% CI -0.5–3.26, p = 0.15) for each single digit increase in the TPR at positivity rates below 35%, but much larger, significant increase of 10.8 cases per 1,000 (95% CI 7.58–14.1, p<0.001) at TPR for rates above 35%.

Sensitivity modeling excluding the village of Izinga, which was the only point with a Cook’s D greater than 1, did not significantly change the resulting coefficient. Additionally, we modeled the relationship between malaria incidence and both the RDT and slide positivity rates. Again, the exponential growth in CMCR remained significant, but the model fit with either the RDT PR or SPR was inferior. These results are summarized in Table 4.

**Table 4:** Sensitivity models exploring the quantitative relationship between test positivity rate (TPR) and confirmed malaria case rate (CMCR).

Model	Exponentiated Coefficient	95% CI	p-value
6 Month TPR*	1.10	1.07–1.13	<0.001
24 Month TPR	1.09	1.06–1.12	<0.001
3 Month TPR*^	1.05	1.02–1.09	<0.001
Excluding Izinga Village	1.06	1.00–1.12	<0.001
RDT PR	1.08	1.05–1.11	<0.001
SPR	1.01	1.00–1.03	<0.001

*Model adjusted for within-village clustering using robust errors.

^Model only includes villages for which at least 15 diagnostic tests were performed each quarter.

TPR = test positivity rate; RDT PR = rapid diagnostic test positivity rate; SPR = slide positivity rate.

## Discussion

We demonstrate an exponential, non-linear relationship between the TPR and CMCR among a rural population in a malaria-endemic region of Western Uganda. This finding has a number of practical implications for malaria surveillance and control programs especially at the extremes of transmission.

The impact of the observed model is perhaps most relevant at relatively low transmission levels, particularly when the TPR falls below 10% and the correlation between TPR and CMCR is poor. For example, a 90% reduction in the TPR from 10% to 1% correlates with only a 56% reduction in the CMCR from 3.9 to only 1.7 cases per 1,000. Our results would suggest that control programs should consider more active surveillance approaches to evaluate interventions in low transmission settings, especially during peak transmission periods as the pre-elimination threshold of 5% is approached.

Notwithstanding the poor correlation at low TPR, the exponential relationship makes relatively minimal changes in TPR suggestive of sizeable change in estimated malaria incidence. For example, we estimate that a similar absolute decrease in TPR from 20% to 10% corresponds to a 62% decrease in CMCR from 10.3 to 3.9 cases per 1,000. These results demonstrate that changes in TPR are more predictive of CMCR when test positivity is above 15%, and that TPR could remain a more valuable surveillance indicator in higher transmission settings.

**Fig 2:**
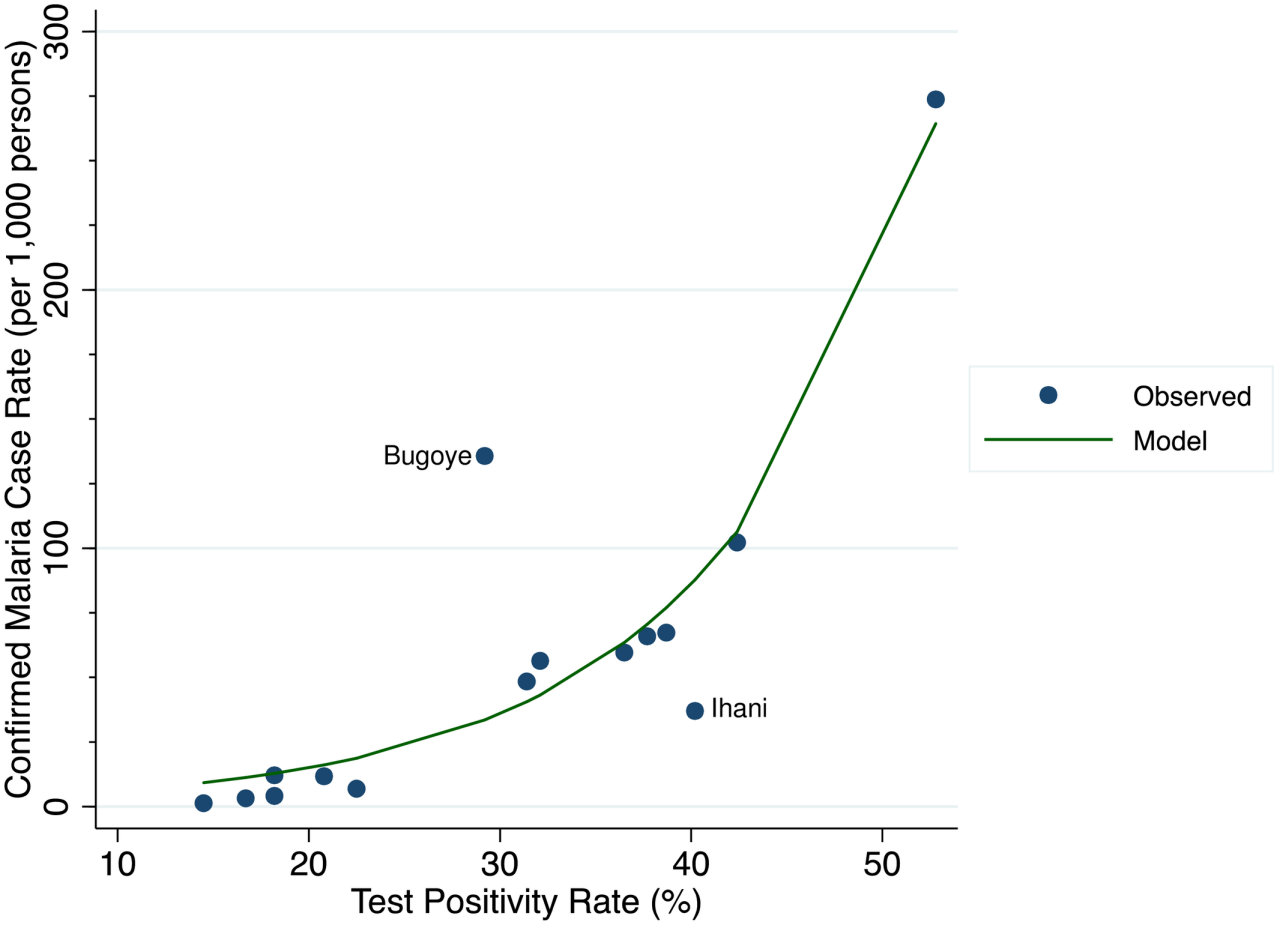
The relationship between the confirmed malaria case rate and test positivity rate for individual villages over the 24-month study period.

Two of the villages in our analysis appear to be outliers from our prediction models, and merit further examination. The first is the village of Ihani, which fell below the exponential fit curve (Fig 2). Ihani had a disproportionately high TPR (40.2%) with a relatively low CMCR (37 per 1,000) compared to other study villages. These findings would suggest that, although the incidence is relatively high, a significant number of malaria cases in Ihani are not being captured at the health center. Notably, of all the included villages, Ihani is closest to another Level III health facility, and thus we suspect that some residents sought care there. If this health-seeking behavior is confirmed, then the TPR-CMCR relationship could be a valuable tool in identifying missed malaria cases.

In contrast, in Bugoye village we see the opposite scenario: a high CMCR (135.7 per 1,000) with a relatively low TPR (29.2%). In this village, we observed that the number of individuals tested as a proportion of the population in Bugoye was much higher (96.8%) than most other villages. We suspect this was due to a misclassification bias, in that the village, parish, and sub-county are all named Bugoye, so staff might have recorded imprecise village residence data.

Our findings are largely consistent with prior work. In a well-defined cohort of Ugandan children who were observed over a period of four years with malaria diagnostic testing for any febrile illness, the investigators compared TPR with malaria incidence [7]. These authors concluded that changes in SPR were associated with changes in malaria incidence, but, as found in our study, the SPR and the incidence of malaria were neither linear nor proportional. In contrast, a large study using routinely collected data from a single county in China, where both *P*. *falciparum* and *P*. *vivax* are present, reported a linear correlation between SPR and the malaria case rate [15]. The SPR in this study, however, was limited to a range of values from less than 1% to approximately 14%, which was a much lower and narrower in distribution than we observed in Uganda. As our findings demonstrate, at SPRs less than 15%, the slope of the curve appears linear, and thus it is not surprising that a linear relationship between SPR and malaria incidence may emerge in such a setting.

Our results were robust to multiple sensitivity analyses. The estimates were similar after varying the measurement intervals, excluding outlying villages, and using different diagnostic testing modalities. Importantly, there were no major changes in national policy clinic staffing, or diagnostic testing strategies during the study observation period. Our study does have a number of methodological limitations. First, we did not directly measure malaria incidence. As such, we cannot be certain that (1) all suspected malaria cases presented to the health center and (2) these individuals underwent diagnostic testing with either RDTs or microscopy. Instead, we utilized the CMCR as a crude surrogate of malaria incidence. Given the remote nature of the study site, the purposeful selection of villages in close proximity to the health center, and the paucity of alternative treatment sites in the area, we believe the CMCR provides a reasonable estimate of clinical malaria incidence. Additionally, the WHO considers CMCR as the most important measure of progress and management in low-incidence areas [3], and thus a greater understanding of the relationship between TPR and CMCR has significant merit for malaria control. Second, our work is generalizable to similar areas of moderate transmission, but should not be used to make assumptions about these relationships in other settings. Finally, we relied on routinely collected HMIS data, which is often subject to incomplete reporting and/or errors. However, aside from the outlier villages discussed above, we do not suspect a differential bias that would influence our findings. Our large sample size and robustness of the model to multiple sensitivity analyses help mitigate concern for this bias.

## Conclusions

Our results demonstrate a non-linear relationship between TPR and CMCR. Malaria surveillance programs utilizing the TPR to estimate and monitor malaria incidence must interpret temporal changes in TPR with these findings in mind. Whereas the TPR remains an attractive measure given its reliance on laboratory-confirmed results and ease of ascertainment, it might be a crude reflection of changes in malaria incidence, particularly at positivity rates less than 10%.

## Supporting Information

S1 Table Diagnostic testing results. (PDF)

## References

[1] Breman JG, Holloway CN. Malaria surveillance counts. The American journal of tropical medicine and hygiene. 2007;77(6 Suppl):36–47. Epub 2008/01/31. . 18165473

[2] WHO. Malaria programme reviews: a manual for reviewing the performance of malaria control and elimination programmes. Geneva: World Health Organization, 2010.

[3] WHO. Disease surveillance for malaria control: an operational manual. Geneva: World Health Organization, 2012.

[4] WHO. Framework for monitoring progress and evaluating outcomes and impact. Geneva: World Health Organization/Roll Back Malaria, 2000.

[5] WHO. World malaria report. Geneva: World Health Organization, 2013.

[6] Francis D, Gasasira A, Kigozi R, Kigozi S, Nasr S, Kamya MR, et al Health facility-based malaria surveillance: the effects of age, area of residence and diagnostics on test positivity rates. Malaria journal. 2012;11:229 Epub 2012/07/10. 10.1186/1475-2875-11-229 ; PubMed Central PMCID: PMCPmc3444404. 22770511 PMC3444404

[7] Jensen TP, Bukirwa H, Njama-Meya D, Francis D, Kamya MR, Rosenthal PJ, et al Use of the slide positivity rate to estimate changes in malaria incidence in a cohort of Ugandan children. Malaria journal. 2009;8:213 Epub 2009/09/17. 10.1186/1475-2875-8-213 ; PubMed Central PMCID: PMCPmc2749863. 19754955 PMC2749863

[8] Hay SI, Guerra CA, Tatem AJ, Noor AM, Snow RW. The global distribution and population at risk of malaria: past, present, and future. The Lancet Infectious diseases. 2004;4(6):327–36. Epub 2004/06/03. 10.1016/s1473-3099(04)01043-6 ; PubMed Central PMCID: PMCPmc3145123. 15172341 PMC3145123

[9] Cibulskis RE, Aregawi M, Williams R, Otten M, Dye C. Worldwide Incidence of Malaria in 2009: Estimates, Time Trends, and a Critique of Methods. PLoS Med. 2011;8(12):e1001142 10.1371/journal.pmed.1001142 22205883 PMC3243721

[10] Ceesay SJ, Casals-Pascual C, Erskine J, Anya SE, Duah NO, Fulford AJ, et al Changes in malaria indices between 1999 and 2007 in The Gambia: a retrospective analysis. Lancet (London, England). 2008;372(9649):1545–54. Epub 2008/11/06. 10.1016/s0140-6736(08)61654-2 ; PubMed Central PMCID: PMCPmc2607025. 18984187 PMC2607025

[11] O'Meara WP, Bejon P, Mwangi TW, Okiro EA, Peshu N, Snow RW, et al Effect of a fall in malaria transmission on morbidity and mortality in Kilifi, Kenya. Lancet (London, England). 2008;372(9649):1555–62. Epub 2008/11/06. 10.1016/s0140-6736(08)61655-4 ; PubMed Central PMCID: PMCPmc2607008. 18984188 PMC2607008

[12] Lee PW, Liu CT, Rampao HS, do Rosario VE, Shaio MF. Pre-elimination of malaria on the island of Principe. Malaria journal. 2010;9:26 Epub 2010/01/22. 10.1186/1475-2875-9-26 ; PubMed Central PMCID: PMCPmc2823607. 20089158 PMC2823607

[13] WHO. Disease surveillance for malaria elimination: an operational manual. Geneva: World Health Organization, 2012.

[14] Partnership RBM. The Global Malaria Action Plan: For a malaria-free world. Geneva: The Roll Back Malaria Partnership, 2008.

[15] Bi Y, Hu W, Liu H, Xiao Y, Guo Y, Chen S, et al Can slide positivity rates predict malaria transmission? Malaria journal. 2012;11:117 Epub 2012/04/20. 10.1186/1475-2875-11-117 ; PubMed Central PMCID: PMCPmc3416572. 22513123 PMC3416572

[16] Joshi PL, Chandra R, Bhattacharya M, Vaish HC. Validity of using slide positivity rate (SPR) in identification of high risk malarious segments in rural areas. The Journal of communicable diseases. 1997;29(1):41–5. Epub 1997/03/01. . 9282528

[17] Yeka A, Gasasira A, Mpimbaza A, Achan J, Nankabirwa J, Nsobya S, et al Malaria in Uganda: challenges to control on the long road to elimination: I. Epidemiology and current control efforts. Acta tropica. 2012;121(3):184–95. Epub 2011/03/23. 10.1016/j.actatropica.2011.03.004 ; PubMed Central PMCID: PMCPmc3156969. 21420377 PMC3156969

[18] Boyce RM, Muiru A, Reyes R, Ntaro M, Mulogo E, Matte M, et al Impact of rapid diagnostic tests for the diagnosis and treatment of malaria at a peripheral health facility in Western Uganda: an interrupted time series analysis. Malaria journal. 2015;14:203 Epub 2015/05/15. 10.1186/s12936-015-0725-0 ; PubMed Central PMCID: PMCPmc4435913. 25971788 PMC4435913

[19] Akaike H. A new look at the statistical model identification. Automatic Control, IEEE Transactions on. 1974;19(6):716–23.

[20] Cook RD. Detection of Influential Observation in Linear Regression. Technometrics. 1977;19(1):15–8. 10.2307/1268249

