# Supplementary material for: Practical Implications of the Non-Linear Relationship between the Test Positivity Rate and Malaria Incidence
Source: PLoS One. 2016 Mar 28;11(3):e0152410. doi: 10.1371/journal.pone.0152410 (PMC4809590; doi:10.1371/journal.pone.0152410)
Supplement: S1 Table — (PDF) [file pone.0152410.s001.pdf]

Number	Date	Sex	Age	Village	RDT	Blood Smear
1	5/1/12	F	13.0	BUNYANGONI	Pos	
2	5/1/12	M	18.0	KAKINDO	Neg	
3	5/2/12	F	2.0	IHANI	Pos	
4	5/2/12	M	9.0	MURAMBA	Neg	
5	5/2/12	F	1.3	BUGOYE	Neg	
6	5/2/12	M	1.6	NDUGHUTU	Neg	
7	5/2/12	M	37.0	IBANDA	Neg	
8	5/2/12	F	12.0	KYARUGOMOKA	Neg	
9	5/2/12	F	28.0	KYARUGOMOKA	Neg	
10	5/2/12	F	12.0	KATOOKE	Neg	
11	5/2/12	F	52.0	BUZIBWERA	Neg	
12	5/2/12	F	54.0	MALIBA	Neg	
13	5/2/12	M	6.0	ISULE	Neg	
14	5/2/12	M	0.7	IHANI	Pos	
15	5/2/12	M	1.0	MALIBA	Neg	
16	5/2/12	F	25.0	BUGOYE	Neg	
17	5/2/12	F	18.0	NDUGHUTU	Neg	
18	5/2/12	M	0.2	BUNYANGONI	Neg	
19	5/2/12	M	11.0	KATUMBA	Neg	
20	5/2/12	F	13.0	KATUMBA	Neg	
21	5/2/12	F	4.0	MALIBA	Pos	
22	5/2/12	M	18.0	NDUGHUTU	Neg	
23	5/2/12	F	11.0	KIKOKERA	Neg	
24	5/2/12	F	20.0	MALIBA	Neg	
25	5/2/12	F	65.0	IHANI	Pos	
26	5/3/12	M	30.0	BUGOYE	Neg	
27	5/3/12	F	0.3	BUGOYE	Neg	
28	5/3/12	M	2.1	KANYAN	Pos	
29	5/3/12	F	26.0	KIRONGO	Neg	
30	5/3/12	M	4.0	RWAKING	Neg	
31	5/3/12	F	19.0	IHANI	Pos	
32	5/3/12	M	0.8	KATOOKE	Neg	
33	5/7/12	F	27.0	IHANI	Neg	
34	5/7/12	F	0.3	KIKOKERA	Neg	
35	5/7/12	M	16.0	KYABUGHA	Neg	
36	5/7/12	F	10.0	IHANI	Pos	
37	5/7/12	M	13.0	IZINGA	Neg	
38	5/7/12	F	18.0	IZINGA	Neg	
39	5/7/12	M	6.0	MALIBA	Pos	
40	5/7/12	F	0.3	MURAMBA	Neg	
41	5/7/12	F	2.0	MALIBA	Neg	
42	5/7/12	M	10.0	RWAKING	Pos	

43	5/7/12	F	23.0	KANYAN	Neg
44	5/7/12	M	0.9	KANYAN	Neg
45	5/7/12	F	4.0	NDUGHUTU	Neg
46	5/7/12	M	15.0	NDUGHUTU	Neg
47	5/7/12	M	4.0	NDUGHUTU	Neg
48	5/7/12	F	2.0	KIRULI	Neg
49	5/8/12	F	5.0	MALIBA	Pos
50	5/8/12	F	2.0	MALIBA	Neg
51	5/8/12	M	0.6	KANYAN	Neg
52	5/8/12	F	1.6	IHANI	Pos
53	5/8/12	F	15.0	KIKOKERA	Neg
54	5/8/12	M	0.8	KIRONGO	Neg
55	5/8/12	M	0.5	MURAMBA	Neg
56	5/8/12	M	37.0	MUBUKU	Pos
57	5/8/12	M	6.0	RWAKING	Neg
58	5/9/12	M	4.0	KANYAN	Neg
59	5/9/12	F	0.7	RWAKING	Neg
60	5/9/12	M	3.5	BUGOYE	Neg
61	5/9/12	M	0.4	MURAMBA	Neg
62	5/9/12	M	30.0	IHANI	Pos
63	5/9/12	M	12.0	IHANI	Neg
64	5/9/12	F	12.0	KANYAN	Pos
65	5/10/12	F	22.0	MUBUKU	Neg
66	5/10/12	F	30.0	IBANDA	Neg
67	5/10/12	M	1.3	BUGOYE	Neg
68	5/10/12	F	19.0	BUGOYE	Neg
69	5/10/12	F	24.0	BUGOYE	Neg
70	5/10/12	F	20.0	KIBOTA	Pos
71	5/11/12	M	1.2	KANYAN	Neg
72	5/11/12	F	2.5	NDUGHUTU	Neg
73	5/11/12	M	2.1	MAGHOMA	Neg
74	5/11/12	M	0.5	BUNYANGONI	Neg
75	5/11/12	F	1.0	BUNYANGONI	Neg
76	5/14/12	M	20.0	KANYAN	Neg
77	5/14/12	F	6.0	KAKINDO	Neg
78	5/14/12	M	3.0	KAKINDO	Neg
79	5/14/12	F	9.0	BUGOYE	Neg
80	5/14/12	M	5.0	MURAMBA	Neg
81	5/14/12	F	5.0	KANYAN	Neg
82	5/14/12	F	5.0	BUNYANGONI	Pos
83	5/14/12	F	20.0	IHANI	Pos
84	5/14/12	F	8.0	MURAMBA	Pos
85	5/14/12	M	12.0	MURAMBA	Neg

86	5/14/12	M	0.6	MURAMBA	Neg
87	5/14/12	F	32.0	IBANDA	Neg
88	5/14/12	F	4.5	MURAMBA	Neg
89	5/14/12	F	0.5	NDUGHUTU	Neg
90	5/14/12	F	9.0	KANYAN	Neg
91	5/14/12	M	1.3	BUGOYE	Neg
92	5/14/12	F	40.0	KIKOKERA	Neg
93	5/14/12	F	8.0	IHANI	Neg
94	5/14/12	F	21.0	BUGOYE	Neg
95	5/14/12	F	16.0	MURAMBA	Neg
96	5/14/12	M	5.0	BULINDUGURU	Neg
97	5/14/12	F	24.0	RWAKING	Neg
98	5/15/12	M	24.0	RWAKING	Neg
99	5/15/12	F	5.0	MURAMBA	Neg
100	5/15/12	M	0.4	BUNYANGONI	Neg
101	5/15/12	M	0.7	KANYAN	Neg
102	5/15/12	F	22.0	KISAMBA	Neg
103	5/15/12	M	66.0	BUGOYE	Neg
104	5/15/12	M	1.8	RWAKING	Neg
105	5/15/12	F	0.4	BUZIBWERA	Neg
106	5/15/12	F	0.8	NDUGHUTU	Neg
107	5/15/12	F	28.0	BUZIBWERA	Neg
108	5/15/12	F	20.0	RWAKING	Neg
109	5/17/12	M	8.0	KISANGA	Neg
110	5/17/12	F	20.0	KANYAN	Neg
111	5/17/12	M	1.2	NYAKARUNGU	Neg
112	5/17/12	F	62.0	MUBUKU	Neg
113	5/17/12	F	4.0	KYARUGOMOKA	Neg
114	5/17/12	M	5.0	KIBIRIZI	Neg
115	5/17/12	F	15.0	MURAMBA	Pos
116	5/17/12	M	62.0	KANYAN	Neg
117	5/17/12	F	2.0	BUNYANGONI	Neg
118	5/17/12	F	33.0	BUNYANGONI	Neg
119	5/17/12	F	40.0	MURAMBA	Neg
120	5/21/12	F	40.0	MURAMBA	Neg
121	5/21/12	F	12.0	KIKOKERA	Pos
122	5/21/12	F	0.8	BUNYANGONI	Neg
123	5/21/12	F	13.0	IZINGA	Pos
124	5/21/12	F	1.1	BUGOYE	Pos
125	5/21/12	M	9.0	BUNYANGONI	Neg
126	5/21/12	M	90.0	.	Neg
127	5/21/12	M	1.8	IHANI	Pos
128	5/21/12	M	12.0	MALIBA	Neg

129	5/21/12	M	56.0	IBANDA	Neg
130	5/21/12	M	0.5	MURAMBA	Neg
131	5/21/12	M	0.7	IZINGA	Neg
132	5/21/12	M	4.5	IZINGA	Neg
133	5/21/12	F	0.3	MALIBA	Neg
134	5/21/12	F	24.0	IZINGA	Neg
135	5/21/12	M	52.0	IZINGA	Neg
136	5/21/12	F	30.0	BUNSONGORA	Neg
137	5/21/12	M	3.5	MALIBA	Pos
138	5/21/12	M	13.0	RWAKING	Neg
139	5/21/12	M	1.0	MURAMBA	Neg
140	5/22/12	M	16.0	IZINGA	Pos
141	5/22/12	M	0.8	KYANYA	Neg
142	5/22/12	F	28.0	BUGOYE	Neg
143	5/22/12	F	3.0	NDUGHUTU	Pos
144	5/23/12	F	25.0	MURAMBA	Neg
145	5/24/12	M	6.7	MURAMBA	Pos
146	5/24/12	M	6.0	IBANDA	Neg
147	5/24/12	M	3.1	BUGOYE	Pos
148	5/28/12	F	18.0	IZINGA	Pos
149	5/28/12	F	4.0	IZINGA	Pos
150	5/28/12	M	1.5	KANYAN	Neg
151	5/28/12	F	1.5	KANYAN	Neg
152	5/28/12	M	31.0	KIRULI	Neg
153	5/28/12	M	31.0	MALIBA	Neg
154	5/28/12	F	20.0	MUHAMBO	Neg
155	5/28/12	F	15.0	KYARUGOMOKA	Pos
156	5/28/12	F	22.0	MURAMBA	Neg
157	5/28/12	M	60.0	BUHUNGA	Neg
158	5/28/12	M	8.0	MURAMBA	Neg
159	5/28/12	F	16.0	MURAMBA	Neg
160	5/28/12	F	30.0	IZINGA	Neg
161	5/28/12	M	0.8	BUNYANGONI	Neg
162	5/28/12	F	5.0	KATOOKE	Pos
163	5/29/12	M	0.1	MUHAMBO	Neg
164	5/29/12	F	24.0	KASANDERA	Pos
165	5/29/12	F	7.0	MALIBA	Neg
166	5/29/12	M	22.0	RWAKING	Neg
167	5/29/12	M	0.8	KYARUGOMOKA	Neg
168	5/29/12	F	33.0	KISANZI	Neg
169	5/29/12	M	0.4	MAGHOMA	Neg
170	5/29/12	M	3.0	MAGHOMA	Pos
171	5/29/12	M	2.5	BUNYANGONI	Pos

172	5/29/12	M	27.0	NDUGHUTU	Pos	
173	5/30/12	F	20.0	KYARUGOMOKA	Pos	
174	5/30/12	M	4.0	KATOOKE	Neg	
175	5/30/12	F	20.0	RWAKING	Neg	
176	5/30/12	F	1.0	KATOOKE	Neg	
177	5/31/12	F	35.0	BUGOYE	Neg	
178	5/31/12	M	23.0	BUGOYE	Neg	
179	5/31/12	M	5.0	KANYAN	Pos	
180	5/31/12	M	26.0	MALIBA	Neg	
181	5/31/12	M	3.0	NDUGHUTU	Neg	
182	5/31/12	M	1.3	KANYAN	Neg	
183	5/31/12	F	2.2	MURAMBA	Pos	
184	5/31/12	F	80.0	RWAKING	Pos	
185	5/31/12	F	24.0	BUGOYE	Neg	
186	6/1/12	M	3.0	KAKINDO	Pos	
187	6/1/12	F	4.0	KAKINDO	Pos	
188	6/1/12	F	22.0	BUGOYE	Neg	
189	6/1/12	M	22.0	KANYAN	Neg	
190	6/1/12	M	17.0	KANYAN	Neg	
191	6/1/12	F	30.0	MURAMBA	Neg	
192	6/1/12	F	40.0	KILEMBE	Neg	
193	6/1/12	F	19.0	.		
194	6/4/12	F	20.0	BUGOYE	Neg	
195	6/4/12	M	1.0	MURAMBA	Neg	
196	6/4/12	F	34.0	ISULE	Neg	
197	6/4/12	F	1.5	KANYAN	Neg	
198	6/4/12	F	40.0	MUHAMBO	Neg	
199	6/4/12	F	0.1	ISULE	Neg	
200	6/4/12	F	11.0	KIBIRIZI	Neg	
201	6/4/12	F	43.0	KIBIRIZI	Neg	
202	6/4/12	F	13.0	BUNYANGONI	Pos	
203	6/4/12	F	1.5	BUZIBWERA	Neg	
204	6/4/12	M	21.0	BUGOYE	Neg	
205	6/4/12	F	20.0	NDUGHUTU	Pos	
206	6/4/12	M	30.0	IHANI	Pos	
207	6/4/12	F	58.0	BUNYANGONI	Pos	
208	6/4/12	F	7.0	KIRULI	Pos	
209	6/4/12	M	3.5	MALIBA	Neg	
210	6/4/12	M	14.0	MUBUKU	Neg	
211	6/5/12	F	0.3	KANYAN		Neg
212	6/5/12	F	9.0	KANYAN		Neg
213	6/5/12	F	3.5	KANYAN		Pos
214	6/5/12	M	0.3	.		Neg

215	6/5/12	F	17.0	IHANI		Neg
216	6/5/12	F	2.5	RWAKING		Neg
217	6/5/12	F	19.0	KANYAN		Neg
218	6/5/12	M	3.0	BUGOYE		Neg
219	6/5/12	M	1.4	KYANYA		Neg
220	6/5/12	F	8.5	RWAKING		Neg
221	6/5/12	M	3.0	RWAKING		Neg
222	6/5/12	M	12.0	BUGOYE		Neg
223	6/5/12	F	32.0	NYAKARUNGU		Neg
224	6/5/12	F	32.0	IZINGA		Pos
225	6/5/12	F	10.0	KANYAN		Neg
226	6/5/12	M	15.0	IZINGA		Neg
227	6/5/12	F	1.4	KAKINDO		Pos
228	6/5/12	F	8.0	MURAMBA		Neg
229	6/5/12	F	0.3	BUNYANGONI		Neg
230	6/5/12	F	0.2	BUNYANGONI		Neg
231	6/5/12	F	3.2	BUGOYE		Neg
232	6/5/12	F	1.3	MAGHOMA		Pos
233	6/6/12	M	67.0	RWAKING	Neg	
234	6/6/12	F	0.8	KISANGA	Neg	
235	6/6/12	F	50.0	IHANI	Pos	
236	6/6/12	M	0.3	NDUGHUTU	Neg	
237	6/6/12	M	70.0	KANYAN		Neg
238	6/6/12	M	1.0	IBANDA		Neg
239	6/6/12	M	63.0	NDUGHUTU		Neg
240	6/6/12	M	3.5	IBANDA		Pos
241	6/6/12	F	24.0	NYAKARUNGU		Neg
242	6/6/12	F	8.0	IZINGA		Pos
243	6/6/12	M	3.0	KILEMBE		Pos
244	6/6/12	M	0.7	BUGOYE		Neg
245	6/6/12	F	20.0	MURAMBA		Pos
246	6/6/12	M	24.0	NYAKARUNGU		Neg
247	6/6/12	F	70.0	MURAMBA		Neg
248	6/7/12	M	8.0	MALIBA	Neg	
249	6/7/12	M	38.0	KIRONGO		Neg
250	6/7/12	F	17.0	KIBIRIZI		Neg
251	6/7/12	F	1.2	BUNYANGONI	Pos	
252	6/7/12	F	17.0	KIKOKERA		Neg
253	6/8/12	F	32.0	BUNYANGONI		Neg
254	6/8/12	M	1.4	BUNYANGONI		Neg
255	6/8/12	M	3.0	NDUGHUTU		Neg
256	6/8/12	F	24.0	MURAMBA		Neg
257	6/8/12	M	22.0	BUGOYE	Neg	

258	6/8/12	M	19.0	RWAKING		Neg
259	6/8/12	F	18.0	IBANDA		Neg
260	6/8/12	F	25.0	BUGOYE		Neg
261	6/8/12	F	24.0	KANYAN		Neg
262	6/8/12	M	0.3	MURAMBA		Neg
263	6/8/12	F	2.4	KANYAN	Pos	Pos
264	6/8/12	F	9.0	MURAMBA		Neg
265	6/8/12	F	17.0	RWAKING		Pos
266	6/8/12	F	11.0	MURAMBA	Neg	
267	6/8/12	F	14.0	IHANI	Pos	
268	6/8/12	M	0.8	KYANYA		Neg
269	6/11/12	M	14.0	MURAMBA		Neg
270	6/11/12	F	1.8	MURAMBA		Pos
271	6/11/12	F	5.0	KIBIRIZI		Pos
272	6/11/12	M	1.2	NDUGHUTU		Neg
273	6/11/12	F	1.1	BULINDUGURU		Neg
274	6/11/12	M	24.0	IBANDA		Neg
275	6/11/12	F	17.0	IZINGA		Neg
276	6/11/12	M	1.0	KANYAN		Pos
277	6/11/12	M	0.3	BUGOYE		Neg
278	6/11/12	M	25.0	IHANI		Neg
279	6/11/12	F	16.0	MAGHOMA		Neg
280	6/11/12	F	22.0	MURAMBA		Neg
281	6/12/12	F	19.0	NDUGHUTU		Pos
282	6/12/12	F	27.0	MASAKA		Neg
283	6/12/12	M	8.0	MURAMBA		Pos
284	6/12/12	M	11.0	MURAMBA	Pos	Neg
285	6/12/12	F	28.0	MURAMBA		Pos
286	6/12/12	M	1.0	BUNYANGONI		Pos
287	6/12/12	M	3.0	KANYAN		Pos
288	6/12/12	F	8.0	KAKINDO		Pos
289	6/12/12	F	9.0	RWAKING		Pos
290	6/12/12	M	0.4	NDUGHUTU		Pos
291	6/12/12	F	60.0	BUNYANGONI		Neg
292	6/12/12	F	5.0	KIKOKERA		Neg
293	6/12/12	F	0.5	.		Neg
294	6/12/12	M	2.5	BUGOYE	Neg	
295	6/12/12	F	38.0	RWAKING		Neg
296	6/12/12	F	39.0	MURAMBA		Neg
297	6/12/12	F	2.0	MURAMBA	Neg	
298	6/13/12	M	1.7	KANYAN		Pos
299	6/13/12	F	29.0	IHANI		Neg
300	6/13/12	M	17.0	MURAMBA		Neg

301	6/13/12	M	17.0	BUGOYE	Neg
302	6/14/12	F	3.0	BUZIBWERA	Pos
303	6/14/12	F	30.0	MALIBA	Neg
304	6/14/12	F	20.0	KYARUGOMOKA	Pos
305	6/14/12	M	2.5	KANYAN	Pos
306	6/15/12	F	0.6	MURAMBA	Pos
307	6/15/12	M	1.0	MURAMBA	Neg
308	6/15/12	M	1.3	IZINGA	Neg
309	6/15/12	F	4.2	IZINGA	Neg
310	6/15/12	M	2.0	BUNYANGONI	Pos
311	6/15/12	F	12.0	KANYAN	Pos
312	6/15/12	M	70.0	MALIBA	Neg
313	6/15/12	M	26.0	BUGOYE	Neg
314	6/15/12	M	0.7	MURAMBA	Neg
315	6/15/12	F	27.0	MURAMBA	Pos
316	6/15/12	F	1.0	IHANI	Pos
317	6/15/12	F	19.0	IHANI	Neg
318	6/15/12	M	16.0	BUGOYE	Neg
319	6/15/12	F	9.0	IBANDA	Neg
320	6/15/12	F	20.0	MURAMBA	Pos
321	6/18/12	F	3.0	MALIBA	Neg
322	6/18/12	M	14.0	IBANDA	Neg
323	6/18/12	M	6.0	MURAMBA	Neg
324	6/18/12	M	2.0	RWAKING	Pos
325	6/18/12	F	11.0	NYAKARUNGU	Neg
326	6/18/12	F	25.0	RWAKING	Pos
327	6/18/12	F	52.0	MALIBA	Neg
328	6/18/12	F	1.4	MURAMBA	Neg
329	6/18/12	F	23.0	KANYAN	Neg
330	6/18/12	M	2.0	MURAMBA	Neg
331	6/18/12	F	26.0	KYARUGOMOKA	Neg
332	6/18/12	F	50.0	MAGHOMA	Pos
333	6/18/12	M	1.2	KYARUGOMOKA	Neg
334	6/18/12	M	4.5	MUBUKU	Neg
335	6/18/12	M	1.1	RWAKING	Neg
336	6/18/12	M	0.8	BUNYANGONI	Neg
337	6/18/12	M	42.0	BUNYANGONI	Neg
338	6/19/12	M	4.0	IZINGA	Pos
339	6/19/12	F	2.0	IZINGA	Pos
340	6/20/12	F	28.0	IZINGA	Pos
341	6/20/12	F	46.0	IZINGA	Neg
342	6/20/12	M	10.0	RWAKING	Neg
343	6/20/12	M	70.0	MAGHOMA	Pos

344	6/20/12	M	2.5	BUGOYE		Neg
345	6/20/12	F	1.5	MALIBA		Neg
346	6/20/12	F	23.0	MALIBA		Neg
347	6/20/12	F	13.0	MURAMBA		Neg
348	6/20/12	M	5.0	KYARUGOMOKA		Neg
349	6/20/12	M	11.0	MALIBA		Neg
350	6/20/12	M	3.0	MALIBA		Neg
351	6/21/12	F	26.0	KYARUGOMOKA		Neg
352	6/21/12	F	12.0	KISANZI		Neg
353	6/21/12	F	27.0	BUGOYE		Neg
354	6/25/12	F	30.0	BUGOYE		Neg
355	6/25/12	F	10.0	NDUGHUTU		Neg
356	6/25/12	M	43.0	RWAKING		Pos
357	6/25/12	F	15.0	BUGOYE		Pos
358	6/25/12	F	25.0	BUNYANGONI		Pos
359	6/25/12	F	50.0	IZINGA		Pos
360	6/25/12	F	19.0	BULINDUGURU		Neg
361	6/25/12	M	13.0	KANYAN		Pos
362	6/25/12	M	27.0	MALIBA		Pos
363	6/25/12	M	35.0	KATUMBA		Neg
364	6/25/12	M	1.5	IZINGA		Neg
365	6/26/12	F	13.0	MURAMBA		Neg
366	6/26/12	M	7.0	BUNYANGONI		Pos
367	6/26/12	F	40.0	KAKINDO		Neg
368	6/26/12	F	4.0	KAKINDO		Pos
369	6/26/12	F	9.0	BUNYANGONI		Neg
370	6/26/12	F	20.0	BUGOYE		Neg
371	6/26/12	F	18.0	BUGOYE		Neg
372	6/26/12	F	19.0	KANYAN		Neg
373	6/26/12	M	50.0	BUNYANGONI		Neg
374	6/26/12	M	72.0	MURAMBA		Neg
375	6/26/12	M	4.0	KISANGA		Neg
376	6/26/12	M	1.5	BUGOYE		Neg
377	6/26/12	M	16.0	IZINGA		Neg
378	6/26/12	F	7.0	IZINGA		Neg
379	6/26/12	F	25.0	BUGOYE		Neg
380	6/26/12	F	16.0	KYANYA		Neg
381	6/26/12	F	8.0	KAKINDO		Neg
382	6/26/12	M	29.0	BUGOYE		Neg
383	6/26/12	M	15.0	BUGOYE		Neg
384	6/26/12	M	6.0	NDUGHUTU		Neg
385	6/27/12	M	23.0	BUNYANGONI		Neg
386	6/27/12	F	29.0	RWAKING	Neg	Neg

387	6/27/12	F	42.0	MURAMBA		Pos
388	6/27/12	F	10.0	IHANI		Pos
389	6/27/12	F	14.0	KANYAN	Neg	
390	6/27/12	F	47.0	KIKOKERA	Neg	
391	6/27/12	M	2.2	RWAKING	Pos	
392	6/27/12	F	3.0	KIKOKERA	Pos	
393	6/27/12	M	5.0	KIKOKERA	Pos	
394	6/27/12	F	0.7	MALIBA	Neg	
395	6/27/12	F	23.0	MURAMBA	Neg	
396	6/27/12	F	34.0	NYANGONGE	Neg	
397	6/27/12	F	30.0	MALIBA	Neg	
398	6/27/12	M	14.0	KANYAN	Pos	
399	6/27/12	F	0.2	IHANI	Neg	
400	6/27/12	M	1.5	MURAMBA	Neg	
401	6/27/12	M	1.0	IZINGA	Neg	
402	6/27/12	F	1.2	BUGOYE	Neg	
403	6/27/12	F	0.9	BUNYANGONI	Neg	
404	6/27/12	M	0.5	BUGOYE	Neg	
405	6/27/12	F	30.0	KATUMBA	Pos	
406	6/27/12	M	8.0	MURAMBA	Neg	
407	6/27/12	F	28.0	BUGOYE	Neg	
408	6/27/12	M	3.0	MALIBA	Pos	
409	6/27/12	F	5.0	MALIBA	Pos	
410	6/27/12	M	35.0	MALIBA	Neg	
411	7/3/12	F	24.0	BUGOYE	Neg	
412	7/3/12	M	3.5	BUNYANGONI	Neg	
413	7/3/12	M	53.0	KAKINDO	Neg	
414	7/3/12	F	75.0	IBANDA	Neg	
415	7/3/12	F	0.2	NYAKARUNGU	Neg	
416	7/3/12	M	0.1	MURAMBA	Neg	
417	7/3/12	M	4.0	IZINGA	Pos	
418	7/3/12	F	22.0	BUGOYE	Neg	
419	7/3/12	F	0.4	BUGOYE	Neg	
420	7/3/12	M	4.0	KAKINDO	Pos	
421	7/3/12	M	0.3	KANYAN	Neg	
422	7/3/12	F	7.0	RWAKING	Pos	
423	7/3/12	F	38.0	NDUGHUTU	Neg	
424	7/3/12	M	0.3	MUBUKU	Neg	
425	7/3/12	F	35.0	MUBUKU	Neg	
426	7/3/12	M	1.4	NYAKARUNGU	Pos	
427	7/3/12	M	1.5	MURAMBA	Pos	
428	7/3/12	F	24.0	KANYAN	Neg	
429	7/3/12	F	22.0	NDUGHUTU	Neg	

430	7/3/12	F	19.0	KANYAN	Neg	
431	7/3/12	M	2.5	KANYAN	Pos	
432	7/3/12	F	6.0	KANYAN	Neg	
433	7/3/12	M	1.1	KATOOKE	Pos	
434	7/3/12	M	0.8	MAGHOMA	Neg	
435	7/3/12	F	18.0	BUGOYE	Pos	
436	7/3/12	F	38.0	BUGOYE	Neg	
437	7/4/12	M	17.0	BUGOYE	Pos	
438	7/4/12	M	3.7	BUGOYE	Neg	
439	7/4/12	F	3.0	KANYAN	Pos	Pos
440	7/4/12	M	0.3	BUNYANGONI	Neg	
441	7/4/12	F	29.0	BUGOYE	Neg	
442	7/4/12	M	0.4	KANYAN	Pos	
443	7/4/12	F	19.0	KANYAN	Neg	
444	7/4/12	M	15.0	KANYAN	Pos	
445	7/4/12	F	0.4	NDUGHUTU	Neg	
446	7/4/12	F	7.0	IZINGA	Pos	
447	7/4/12	F	10.0	IZINGA	Pos	
448	7/4/12	M	5.0	IZINGA	Neg	
449	7/4/12	F	22.0	KANYAN	Neg	
450	7/4/12	M	5.0	NYAKARUNGU	Pos	
451	7/4/12	M	5.5	MURAMBA	Neg	
452	7/4/12	F	18.0	IZINGA	Neg	
453	7/4/12	M	1.4	IZINGA	Neg	
454	7/4/12	F	0.6	BUGOYE	Neg	
455	7/4/12	F	38.0	KATUMBA	Neg	
456	7/4/12	F	20.0	NYAKARUNGU	Pos	
457	7/4/12	F	23.0	MURAMBA	Pos	
458	7/4/12	M	0.4	KISAMBA	Neg	
459	7/4/12	F	8.0	NYAKARUNGU	Pos	
460	7/4/12	F	6.0	NYAKARUNGU	Pos	
461	7/4/12	F	67.0	BUGOYE	Neg	
462	7/4/12	M	0.3	MURAMBA	Pos	
463	7/5/12	M	30.0	BUGOYE	Neg	
464	7/5/12	F	3.0	KYARUGOMOKA	Pos	
465	7/5/12	M	2.5	BUGOYE	Pos	
466	7/5/12	M	50.0	BUGOYE	Neg	
467	7/5/12	F	23.0	BUGOYE	Neg	
468	7/5/12	M	3.0	KANYAN	Pos	
469	7/5/12	F	0.6	KANYAN	Pos	
470	7/5/12	F	32.0	BUGOYE	Pos	
471	7/5/12	M	3.0	IZINGA	Pos	
472	7/5/12	F	38.0	IZINGA	Neg	

473	7/5/12	F	23.0	RWAKING	Neg	
474	7/5/12	M	2.5	KINYABEBE	Neg	
475	7/5/12	F	0.1	BUNYANGONI	Neg	
476	7/5/12	M	5.0	RWAKING	Neg	
477	7/5/12	M	4.5	BUNYANGONI	Neg	
478	7/5/12	F	54.0	KIKOKERA	Pos	
479	7/5/12	F	75.0	BUGOYE	Neg	
480	7/6/12	M	7.0	BUNYANGONI	Pos	
481	7/6/12	M	50.0	BUNYANGONI	Neg	
482	7/6/12	F	50.0	NYAKARUNGU	Neg	
483	7/6/12	M	50.0	KANYAN	Neg	
484	7/6/12	M	16.0	BUZIBWERA	Pos	
485	7/6/12	M	4.0	NYAKABUGHA	Neg	
486	7/6/12	M	40.0	RWAKING	Pos	
487	7/6/12	F	20.0	MURAMBA	Neg	
488	7/6/12	F	16.0	IBANDA	Neg	
489	7/6/12	M	55.0	NYAKABUGHA	Pos	
490	7/6/12	M	14.0	RWAKING	Pos	Pos
491	7/6/12	F	35.0	MALIBA	Neg	
492	7/6/12	F	3.0	KYANYA	Neg	
493	7/6/12	F	1.0	KYANYA	Neg	
494	7/9/12	M	11.0	MALIBA	Pos	
495	7/9/12	F	14.0	BUZIBWERA	Neg	
496	7/9/12	F	35.0	BUZIBWERA	Neg	
497	7/9/12	F	6.0	KANYAN	Pos	
498	7/9/12	M	1.0	BUZIBWERA	Pos	
499	7/9/12	M	14.0	KANYAN	Pos	
500	7/9/12	F	10.0	KAKINDO	Pos	
501	7/9/12	F	1.7	BUGOYE	Pos	
502	7/9/12	F	8.0	BUGOYE	Pos	
503	7/9/12	F	37.0	BUGOYE	Neg	
504	7/9/12	F	16.0	KYANDALE	Neg	
505	7/9/12	M	7.0	KANYAN	Pos	
506	7/9/12	F	5.0	KANYAN	Neg	
507	7/9/12	F	14.0	BUZIBWERA	Neg	
508	7/9/12	F	2.0	MALIBA	Pos	
509	7/9/12	F	9.0	KAKINDO	Neg	
510	7/9/12	F	21.0	NYAKABUGHA	Neg	
511	7/9/12	F	3.0	RWAKING	Pos	
512	7/9/12	F	4.5	MURAMBA	Pos	
513	7/9/12	F	37.0	MALIBA	Pos	
514	7/9/12	F	1.3	KANYAN	Neg	
515	7/9/12	F	18.0	MALIBA	Neg	

516	7/9/12	F	1.3	IHANI	Pos	
517	7/9/12	M	24.0	BUGOYE	Neg	
518	7/9/12	F	9.8	BUNYANGONI	Neg	
519	7/9/12	M	24.0	RWAKING	Pos	
520	7/10/12	M	17.0	MURAMBA	Pos	
521	7/10/12	M	7.0	MURAMBA		Pos
522	7/10/12	F	53.0	BUGOYE	Neg	
523	7/10/12	F	3.0	KANYAN	Pos	
524	7/10/12	M	30.0	KANYAN	Neg	
525	7/10/12	F	40.0	KIKOKERA	Pos	
526	7/10/12	F	47.0	KIRONGO	Neg	
527	7/10/12	F	0.8	MURAMBA	Neg	
528	7/10/12	F	2.0	BUZIBWERA		
529	7/10/12	F	40.0	MALIBA	Neg	
530	7/10/12	F	4.0	BUGOYE	Pos	
531	7/10/12	M	23.0	BUNYANGONI	Neg	
532	7/10/12	F	13.0	BUGOYE	Pos	
533	7/10/12	F	3.1	IZINGA	Pos	
534	7/10/12	M	0.0	BUNYANGONI	Neg	
535	7/10/12	M	73.0	RWAKING	Neg	
536	7/10/12	M	1.1	NDUGHUTU	Neg	
537	7/10/12	F	1.3	KISORO	Pos	
538	7/10/12	F	20.0	IBANDA	Neg	
539	7/10/12	M	4.0	IBANDA	Pos	
540	7/10/12	M	43.0	KIRULI	Neg	
541	7/10/12	M	0.3	BUGOYE	Neg	
542	7/10/12	M	21.0	KIKOKERA	Pos	
543	7/10/12	F	50.0	BUNYANGONI	Pos	
544	7/10/12	F	25.0	MALIBA	Neg	
545	7/10/12	F	35.0	KISORO	Neg	
546	7/11/12	F	2.0	IHANI	Neg	
547	7/11/12	F	25.0	MURAMBA	Neg	
548	7/11/12	F	46.0	MALIBA	Neg	
549	7/11/12	M	0.3	MAGHOMA	Neg	
550	7/11/12	M	0.7	BUNYANGONI	Neg	
551	7/11/12	M	2.0	MALIBA	Pos	
552	7/11/12	M	1.6	MUHAMBO	Neg	
553	7/11/12	F	0.5	BUNYANGONI	Neg	
554	7/11/12	M	9.0	KIKOKERA	Pos	
555	7/11/12	F	6.0	IZINGA	Pos	
556	7/11/12	F	1.8	BUNYANGONI	Neg	
557	7/12/12	F	14.0	KIKOKERA	Neg	
558	7/12/12	F	24.0	KISOJO	Neg	

559	7/12/12	F	0.7	KIKOKERA	Pos
560	7/12/12	M	0.4	MURAMBA	Neg
561	7/12/12	M	4.4	IZINGA	Neg
562	7/12/12	M	16.0	KANYAN	Pos
563	7/12/12	M	0.7	BUNYANGONI	Neg
564	7/12/12	M	3.5	KANYAN	Pos
565	7/12/12	M	4.6	KYARUGOMOKA	Pos
566	7/12/12	F	0.7	KANYAN	Neg
567	7/12/12	F	1.0	KATUMBA	Neg
568	7/12/12	M	3.0	BUGOYE	Neg
569	7/12/12	F	54.0	BUNYANGONI	Pos
570	7/12/12	F	40.0	IBANDA	Neg
571	7/12/12	F	48.0	KAKINDO	Pos
572	7/12/12	M	7.0	KAKINDO	Pos
573	7/12/12	F	24.0	IZINGA	Pos
574	7/12/12	F	10.0	IZINGA	Pos
575	7/13/12	M	3.2	NDUGHUTU	Neg
576	7/13/12	M	0.2	KIRULI	Pos
577	7/13/12	F	20.0	BUZIBWERA	Pos
578	7/13/12	F	20.0	KANYAN	Neg
579	7/13/12	M	0.8	RWAKING	Neg
580	7/13/12	F	12.0	KANYAN	Neg
581	7/13/12	F	0.1	MUHAMBO	Neg
582	7/13/12	F	1.2	KIKOKERA	Pos
583	7/13/12	M	20.0	NDUGHUTU	Pos
584	7/13/12	F	30.0	KATUMBA	Neg
585	7/13/12	M	9.0	MURAMBA	Pos
586	7/13/12	M	0.5	KANYAN	Neg
587	7/13/12	F	20.0	NDUGHUTU	Neg
588	7/13/12	F	13.0	BUNYANGONI	Pos
589	7/13/12	M	6.0	KANYAN	Pos
590	7/13/12	F	11.0	KANYAN	Neg
591	7/13/12	F	3.0	KYARUGOMOKA	Pos
592	7/13/12	M	0.5	KYARUGOMOKA	Neg
593	7/13/12	F	8.0	KYARUGOMOKA	Neg
594	7/13/12	F	29.0	BUNYANGONI	Pos
595	7/13/12	F	22.0	KYARUGOMOKA	Neg
596	7/13/12	M	3.0	KYARUGOMOKA	Pos
597	7/13/12	F	29.0	IBANDA	Neg
598	7/13/12	F	40.0	KIKOKERA	Pos
599	7/13/12	F	26.0	KIKOKERA	Pos
600	7/13/12	F	22.0	MAGHOMA	Neg
601	7/13/12	F	10.0	KANYAN	Pos

602	7/13/12	F	13.0	IZINGA	Neg
603	7/13/12	F	12.0	KYARUGOMOKA	Pos
604	7/13/12	F	1.3	KIRONGO	Neg
605	7/16/12	M	2.0	KATUMBA	Neg
606	7/16/12	M	0.9	KATUMBA	Neg
607	7/16/12	M	0.4	BUNYANGONI	Neg
608	7/16/12	F	40.0	KANYAN	Neg
609	7/16/12	F	9.0	KANYAN	Pos
610	7/16/12	F	15.0	KANYAN	Neg
611	7/16/12	F	0.8	IZINGA	Neg
612	7/16/12	F	40.0	KANYAN	Pos
613	7/16/12	F	20.0	KANYAN	Pos
614	7/16/12	F	0.5	BUGOYE	Neg
615	7/16/12	F	3.0	KYARUGOMOKA	Pos
616	7/16/12	M	13.0	KYARUGOMOKA	Neg
617	7/16/12	F	5.1	KANYAN	Pos
618	7/16/12	M	13.0	IZINGA	Pos
619	7/16/12	F	21.0	IBANDA	Neg
620	7/16/12	F	30.0	KATUMBA	Neg
621	7/16/12	F	1.3	KIRONGO	Neg
622	7/16/12	M	14.0	KYARUGOMOKA	Pos
623	7/16/12	M	2.0	KANYAN	Pos
624	7/16/12	F	6.0	BUGOYE	Pos
625	7/16/12	M	11.0	KAKINDO	Pos
626	7/16/12	M	0.6	IZINGA	Pos
627	7/16/12	M	1.5	NYAKARUNGU	Pos
628	7/16/12	M	30.0	MUHAMBO	Pos
629	7/16/12	F	0.3	KANYAN	Pos
630	7/16/12	F	15.0	MURAMBA	Pos
631	7/16/12	M	5.0	MURAMBA	Neg
632	7/16/12	M	1.7	.	Pos
633	7/16/12	F	50.0	KANYAN	Pos
634	7/16/12	M	2.0	NYAKARUNGU	Pos
635	7/16/12	F	26.0	MURAMBA	Neg
636	7/16/12	F	1.8	NYAKARUNGU	Neg
637	7/16/12	F	14.0	BUGOYE	Neg
638	7/16/12	M	1.5	ISULE	Neg
639	7/16/12	M	0.5	NYANGONGE	Neg
640	7/16/12	F	11.0	IZINGA	Pos
641	7/16/12	M	14.0	BUNYANGONI	Pos
642	7/16/12	M	1.0	NDUGHUTU	Neg
643	7/16/12	F	23.0	MURAMBA	Pos
644	7/16/12	M	0.3	MURAMBA	Pos

645	7/17/12	M	20.0	MURAMBA	Neg	
646	7/17/12	M	35.0	RWAKING	Neg	
647	7/17/12	M	31.0	BUGOYE	Pos	
648	7/17/12	M	1.6	BUGOYE	Neg	
649	7/17/12	F	45.0	IZINGA	Neg	
650	7/17/12	F	25.0	NDUGHUTU	Neg	
651	7/17/12	M	0.8	MALIBA	Neg	
652	7/17/12	F	18.0	KANYAN	Neg	
653	7/17/12	F	18.0	BUGOYE	Neg	
654	7/17/12	F	90.0	BUGOYE	Neg	
655	7/17/12	F	10.0	KANYAN	Pos	
656	7/18/12	M	54.0	KATUMBA	Neg	
657	7/18/12	F	10.0	MURAMBA	Pos	
658	7/18/12	M	0.7	NDUGHUTU	Pos	
659	7/18/12	F	5.0	IZINGA	Pos	
660	7/18/12	F	3.0	MALIBA	Pos	
661	7/18/12	M	4.0	MALIBA	Pos	
662	7/18/12	F	17.0	KIKOKERA	Pos	
663	7/18/12	F	1.3	BUNYANGONI	Pos	
664	7/18/12	M	2.5	MALIBA	Neg	
665	7/18/12	M	40.0	NYAKARUNGU	Neg	
666	7/18/12	M	19.0	MALIBA	Neg	
667	7/19/12	F	0.2	BULYANDEKE		Neg
668	7/23/12	M	23.0	BUGOYE		Neg
669	7/23/12	F	1.8	KANYAN		Neg
670	7/23/12	M	5.0	KIKOKERA		Pos
671	7/23/12	F	3.0	NYAKARUNGU		Neg
672	7/23/12	F	2.0	BUNYANGONI		Pos
673	7/23/12	M	20.0	KIKOKERA		Neg
674	7/23/12	F	14.0	BUGOYE		Pos
675	7/23/12	M	1.5	KANYAN		Pos
676	7/23/12	F	7.0	KANYAN		Neg
677	7/23/12	F	0.5	KANYAN		Neg
678	7/23/12	M	10.0	MALIBA		Neg
679	7/23/12	M	4.3	MALIBA		Pos
680	7/23/12	F	20.0	MURAMBA		Neg
681	7/23/12	M	17.0	NDUGHUTU		Neg
682	7/23/12	F	7.0	KAKINDO		Neg
683	7/23/12	M	0.4	KAKINDO		Pos
684	7/23/12	M	1.0	BUZIBWERA		Pos
685	7/23/12	F	17.0	IZINGA		Neg
686	7/23/12	F	75.0	IZINGA		Pos
687	7/23/12	F	1.0	.		Neg

688	7/23/12	F	24.0	.	Pos
689	7/23/12	M	4.0	BUNYANGONI	Neg
690	7/23/12	F	6.0	IHANI	Pos
691	7/23/12	F	2.0	BUZIBWERA	Pos
692	7/23/12	F	1.1	MURAMBA	Neg
693	7/23/12	M	13.0	MURAMBA	Pos
694	7/23/12	F	45.0	KANYAN	Pos
695	7/24/12	M	64.0	BUNYANGONI	Neg
696	7/24/12	F	5.0	KAKINDO	Neg
697	7/24/12	F	17.0	KAKINDO	Pos
698	7/24/12	M	14.0	KANYAN	Pos
699	7/24/12	F	0.8	KATUMBA	Neg
700	7/24/12	F	35.0	BUZIBWERA	Pos
701	7/24/12	F	29.0	IZINGA	Neg
702	7/24/12	F	1.0	RWAKING	Neg
703	7/24/12	F	6.0	KANYAN	Pos
704	7/24/12	F	18.0	MALIBA	Neg
705	7/24/12	F	65.0	KIHINDI	Neg
706	7/25/12	M	2.0	MURAMBA	Neg
707	7/25/12	F	37.0	BUNYANGONI	Neg
708	7/25/12	F	54.0	BUNYANGONI	Neg
709	7/25/12	F	22.0	KANYAN	Neg
710	7/25/12	F	19.0	KIGHOME	Neg
711	7/25/12	F	38.0	IBANDA	Neg
712	7/25/12	F	20.0	BUHUNGA	Neg
713	7/26/12	M	1.0	BUNYANGONI	Neg
714	7/26/12	F	53.0	BUGOYE	Pos
715	7/26/12	F	18.0	NDUGHUTU	Pos
716	7/26/12	F	20.0	KANYAN	Pos
717	7/26/12	M	2.2	KYANYA	Neg
718	7/26/12	M	2.3	KANYAN	Neg
719	7/26/12	F	38.0	NYAKARUNGU	Neg
720	7/26/12	F	16.0	KABUGHO	Pos
721	7/26/12	F	17.0	BUGOYE	Pos
722	7/26/12	M	12.0	NDUGHUTU	Neg
723	7/26/12	F	22.0	KANYAN	Pos
724	7/27/12	M	24.0	BUGOYE	Pos
725	7/27/12	F	8.0	MALIBA	Pos
726	7/27/12	F	5.0	MALIBA	Pos
727	7/27/12	F	5.0	KANYAN	Neg
728	7/27/12	M	9.0	RWAKING	Pos
729	7/27/12	M	3.0	KANYAN	Pos
730	7/27/12	F	2.0	MURAMBA	Neg

731	7/27/12	F	13.0	BUNYANGONI	Neg
732	7/27/12	F	0.3	IZINGA	Neg
733	7/27/12	F	15.0	BUZIBWERA	Neg
734	7/27/12	F	50.0	KANYAN	Neg
735	7/27/12	M	0.4	KANYAN	Neg
736	7/27/12	M	11.0	BUGOYE	Neg
737	7/27/12	F	4.0	KANYAN	Pos
738	7/27/12	M	2.0	BUGOYE	Pos
739	7/27/12	F	1.0	KATUMBA	Neg
740	7/27/12	F	30.0	BUGOYE	Neg
741	7/27/12	M	22.0	IZINGA	Pos
742	7/30/12	M	30.0	IZINGA	Pos
743	7/30/12	F	40.0	KIBIKO	Neg
744	7/30/12	F	2.0	KANYAN	Pos
745	7/30/12	F	53.0	BUGOYE	Neg
746	7/30/12	M	2.4	BUNYANGONI	Neg
747	7/30/12	F	1.2	MURAMBA	Neg
748	7/30/12	M	13.0	IHANI	Neg
749	7/30/12	F	14.0	IBANDA	Neg
750	7/30/12	F	24.0	KANYAN	Neg
751	7/30/12	F	25.0	KISOJO	Pos
752	7/30/12	F	5.0	KANYAN	Neg
753	7/30/12	F	22.0	KANYAN	Neg
754	7/30/12	M	50.0	RWAKING	Neg
755	7/30/12	M	20.0	NDUGHUTU	Pos
756	7/30/12	F	.	NYAKABUGHA	Neg
757	7/30/12	F	.	BUNYANGONI	Neg
758	7/30/12	M	1.1	MURAMBA	Neg
759	7/30/12	F	17.0	IBANDA	Neg
760	7/30/12	F	0.4	MALIBA	Neg
761	7/31/12	M	0.6	NDUGHUTU	Neg
762	7/31/12	F	21.0	KANYAN	Neg
763	7/31/12	M	2.2	BUZIBWERA	Neg
764	7/31/12	M	17.0	KANYAN	Neg
765	7/31/12	M	4.4	KANYAN	Pos
766	7/31/12	M	3.0	KANYAN	Pos
767	7/31/12	F	0.3	KANYAN	Neg
768	7/31/12	M	1.2	RWAKING	Pos
769	7/31/12	F	46.0	NYAKARUNGU	Neg
770	7/31/12	F	2.2	IZINGA	Neg
771	7/31/12	M	14.0	IZINGA	Neg
772	7/31/12	F	18.0	IZINGA	Neg
773	7/31/12	F	37.0	MOPAZU	Pos

774	7/31/12	M	0.6	MUPAZU	Neg
775	7/31/12	M	5.0	KYARUGOMOKA	Neg
776	7/31/12	F	2.0	KYARUGOMOKA	Pos
777	7/31/12	F	54.0	MALIBA	Neg
778	7/31/12	F	20.0	BUZIBWERA	Neg
779	7/31/12	F	30.0	RWAKING	Neg
780	7/31/12	F	9.0	RWAKING	Neg
781	7/31/12	F	7.0	IZINGA	Neg
782	7/31/12	F	18.0	KATOOKE	Neg
783	8/1/12	M	2.5	MALIBA	Pos
784	8/1/12	F	20.0	KANYAN	Neg
785	8/1/12	M	3.0	IZINGA	Pos
786	8/1/12	F	36.0	MURAMBA	Pos
787	8/1/12	F	35.0	MUHAMBO	Neg
788	8/1/12	M	7.0	KANYAN	Neg
789	8/1/12	M	1.2	NYAKARUNGU	Neg
790	8/1/12	M	16.0	BUGOYE	Neg
791	8/1/12	F	17.0	KATOOKE	Neg
792	8/1/12	F	18.0	BUGOYE	Neg
793	8/1/12	F	11.0	BUNYANGONI	Neg
794	8/1/12	F	1.1	KABUKERA	Neg
795	8/1/12	F	21.0	NDUGHUTU	Neg
796	8/3/12	F	23.0	RWAKING	Neg
797	8/3/12	F	16.0	NDUGHUTU	Neg
798	8/3/12	F	12.0	IZINGA	Pos
799	8/3/12	F	19.0	NDUGHUTU	Neg
800	8/3/12	F	1.0	BUGOYE	Pos
801	8/3/12	F	21.0	NYAKARUNGU	Neg
802	8/3/12	M	1.4	MURAMBA	Neg
803	8/6/12	F	14.0	RWAKING	Pos
804	8/6/12	M	1.6	MURAMBA	Pos
805	8/6/12	F	28.0	KANYAN	Neg
806	8/6/12	M	1.0	KANYAN	Neg
807	8/6/12	F	22.0	IZINGA	Pos
808	8/6/12	F	3.0	KAKINDO	Neg
809	8/6/12	F	4.1	NDUGHUTU	Neg
810	8/6/12	M	13.0	IHANI	Neg
811	8/6/12	M	1.1	NDUGHUTU	Neg
812	8/7/12	M	1.5	BUGOYE	Pos
813	8/7/12	F	0.6	BUGOYE	Pos
814	8/7/12	F	17.0	KIKOKERA	Pos
815	8/7/12	M	7.0	BUGOYE	Pos
816	8/7/12	M	1.2	MUHAMBO	Pos

817	8/7/12	M	32.0	NDUGHUTU	Neg
818	8/7/12	M	47.0	KIKOKERA	Neg
819	8/7/12	M	29.0	BUGOYE	Neg
820	8/7/12	F	29.0	MUHAMBO	Neg
821	8/7/12	F	40.0	BUNYANGONI	Neg
822	8/9/12	F	25.0	MALIBA	Neg
823	8/9/12	F	23.0	MURAMBA	Neg
824	8/9/12	M	0.3	BUNYANGONI	Neg
825	8/9/12	M	2.0	BUGOYE	Neg
826	8/9/12	F	35.0	KISANZI	Neg
827	8/10/12	M	0.2	IZINGA	Neg
828	8/10/12	M	50.0	IZINGA	Neg
829	8/14/12	F	9.0	KIKOKERA	Neg
830	8/14/12	F	9.0	BUNYANGONI	Neg
831	8/14/12	F	16.0	NDUGHUTU	Neg
832	8/14/12	F	17.0	RWAKING	Pos
833	8/14/12	F	1.3	KANYAN	Neg
834	8/14/12	F	0.3	BUNYANGONI	Neg
835	8/14/12	F	2.4	KIRULI	Neg
836	8/14/12	F	14.0	NDUGHUTU	Pos
837	8/14/12	M	2.1	KANYAN	Neg
838	8/14/12	M	13.0	MALIBA	Pos
839	8/14/12	M	16.0	RWAKING	Pos
840	8/15/12	F	22.0	KATOOKE	Neg
841	8/15/12	M	0.3	KABUYIRI	Neg
842	8/15/12	F	20.0	BUGOYE	Neg
843	8/15/12	F	22.0	BUGOYE	Neg
844	8/16/12	M	7.0	KANYAN	Neg
845	8/16/12	M	13.0	MURAMBA	Neg
846	8/16/12	F	27.0	MALIBA	Neg
847	8/16/12	F	64.0	BUZIBWERA	Pos
848	8/16/12	F	22.0	RWAKING	Pos
849	8/16/12	F	4.0	ISULE	Pos
850	8/16/12	M	15.0	MUHAMBO	Neg
851	8/16/12	F	17.0	KANYAN	Neg
852	8/16/12	F	18.0	IZINGA	Pos
853	8/16/12	M	0.9	IZINGA	Pos
854	8/16/12	M	1.3	NDUGHUTU	Neg
855	8/16/12	F	14.0	MURAMBA	Pos
856	8/16/12	F	7.0	IZINGA	Pos
857	8/17/12	F	25.0	BUGOYE	Neg
858	8/17/12	M	22.0	NDUGHUTU	Neg
859	8/17/12	M	79.0	IBANDA	Pos

860	8/17/12	F	19.0	BUGOYE	Pos
861	8/17/12	F	20.0	BUGOYE	Neg
862	8/17/12	F	4.3	BUGOYE	Pos
863	8/17/12	F	19.0	MURAMBA	Neg
864	8/20/12	F	0.5	BUNYANGONI	Pos
865	8/20/12	M	22.0	NDUGHUTU	Pos
866	8/20/12	F	0.6	KYANYA	Neg
867	8/20/12	M	24.0	BUNYANGONI	Pos
868	8/20/12	M	54.0	KANYAN	Pos
869	8/20/12	F	4.6	KYARUGOMOKA	Pos
870	8/20/12	M	2.0	MALIBA	Neg
871	8/20/12	F	7.0	BUGOYE	Pos
872	8/20/12	F	20.0	MURAMBA	Neg
873	8/20/12	F	1.5	BUZIBWERA	Neg
874	8/20/12	F	50.0	KYANYA	Neg
875	8/20/12	F	40.0	IZINGA	Neg
876	8/20/12	F	20.0	BUNYANGONI	Neg
877	8/20/12	M	2.0	BUGOYE	Pos
878	8/20/12	M	0.5	BUNYANGONI	Neg
879	8/20/12	M	28.0	RWAKING	Neg
880	8/20/12	M	45.0	BIKONE	Pos
881	8/20/12	F	0.3	MALIBA	Neg
882	8/21/12	M	54.0	IZINGA	Pos
883	8/21/12	F	20.0	MURAMBA	Neg
884	8/21/12	F	25.0	KYARUGOMOKA	Neg
885	8/21/12	M	0.8	BUHUNGA	Neg
886	8/21/12	M	1.0	MAGHOMA	Neg
887	8/21/12	F	20.0	KATOOKE	Pos
888	8/21/12	F	0.2	KANYAN	Pos
889	8/22/12	F	54.0	KANYAN	Neg
890	8/22/12	M	23.0	KANYAN	Neg
891	8/22/12	M	0.8	BUNYANGONI	Neg
892	8/22/12	F	2.0	KIKOKERA	Neg
893	8/22/12	F	18.0	KATUMBA	Neg
894	8/22/12	F	13.0	IZINGA	Neg
895	8/22/12	F	19.0	BUGOYE	Neg
896	8/22/12	M	6.0	KANYAN	Neg
897	8/22/12	M	7.0	IZINGA	Neg
898	8/22/12	F	18.0	KIRULI	Neg
899	8/23/12	M	3.0	NDUGHUTU	Neg
900	8/23/12	M	22.0	BUGOYE	Pos
901	8/23/12	M	13.0	NYAKARUNGU	Pos
902	8/23/12	M	60.0	IZINGA	Pos

903	8/23/12	M	3.0	IZINGA		Pos
904	8/23/12	F	0.9	NYANGONGE		Neg
905	8/23/12	F	4.0	KANYAN		Pos
906	8/27/12	F	35.0	BUGOYE		Neg
907	8/27/12	F	40.0	BUGOYE		Neg
908	8/27/12	F	19.0	KANYAN		Neg
909	8/27/12	F	15.0	NYAKARUNGU		Neg
910	8/27/12	F	15.0	NDUGHUTU	Neg	
911	8/27/12	F	29.0	MUBUKU	Neg	
912	8/27/12	M	38.0	KAKINDO		Pos
913	8/27/12	F	1.5	MURAMBA		Neg
914	8/27/12	M	20.0	BUGOYE		Neg
915	8/27/12	F	16.0	NDUGHUTU		Neg
916	8/27/12	M	5.0	KANYAN		Pos
917	8/27/12	F	15.0	NYAKARUNGU	Neg	
918	8/27/12	M	1.0	NYAKARUNGU	Pos	
919	8/27/12	M	0.5	MALIBA	Neg	
920	8/27/12	M	1.0	KANYAN	Neg	
921	8/27/12	F	1.0	KYARUGOMOKA	Pos	
922	8/27/12	F	37.0	KYARUGOMOKA	Neg	
923	8/27/12	F	26.0	BUGOYE	Pos	
924	8/27/12	M	1.2	KISANZI	Pos	
925	8/27/12	M	40.0	BUNYANGONI	Neg	
926	8/27/12	F	4.4	BUGOYE	Neg	
927	8/27/12	M	25.0	MALIBA	Neg	
928	8/27/12	M	35.0	NDUGHUTU	Pos	
929	8/28/12	F	30.0	KANYAN	Neg	
930	8/28/12	F	23.0	RWAKING	Neg	
931	8/28/12	F	40.0	IZINGA	Pos	
932	8/28/12	F	51.0	KANYAN	Neg	Neg
933	8/28/12	M	37.0	MALIBA	Neg	
934	8/28/12	M	5.0	MURAMBA	Pos	
935	8/28/12	M	4.0	IZINGA	Pos	
936	8/28/12	M	8.0	MURAMBA	Neg	
937	8/28/12	F	3.5	MURAMBA	Neg	
938	8/28/12	M	73.0	BUGOYE	Neg	
939	8/28/12	F	0.8	MURAMBA	Neg	
940	8/28/12	F	40.0	MURAMBA	Neg	
941	8/28/12	M	8.0	RWAKING	Pos	
942	8/28/12	F	12.0	RWAKING	Pos	
943	8/28/12	M	5.0	RWAKING	Pos	
944	8/28/12	M	0.8	RWAKING	Neg	
945	8/29/12	F	56.0	KANYAN	Neg	

946	8/29/12	F	22.0	BUGOYE	Pos
947	8/29/12	F	52.0	KANYAN	Neg
948	8/29/12	M	33.0	NYAKARUNGU	Pos
949	8/29/12	F	34.0	KYARUGOMOKA	Neg
950	8/29/12	F	34.0	KANYAN	Neg
951	8/29/12	F	35.0	BUGOYE	Neg
952	8/29/12	F	32.0	IBANDA	Neg
953	8/29/12	F	14.0	MURAMBA	Neg
954	8/29/12	M	4.0	NYAKARUNGU	Pos
955	8/29/12	F	22.0	KAKINDO	Neg
956	8/29/12	F	20.0	KAKINDO	Neg
957	8/29/12	F	23.0	MALIBA	Neg
958	8/30/12	F	20.0	RWAKING	Neg
959	8/30/12	F	20.0	MALIBA	Neg
960	8/30/12	F	3.0	KIKOKERA	Pos
961	8/30/12	F	5.0	KIKOKERA	Pos
962	8/30/12	F	31.0	MURAMBA	Neg
963	8/30/12	M	27.0	NDUGHUTU	Neg
964	8/30/12	M	17.0	IBANDA	Neg
965	8/30/12	F	27.0	NDUGHUTU	Neg
966	8/30/12	F	12.0	IHANI	Neg
967	8/30/12	F	35.0	NYAKARUNGU	Neg
968	8/30/12	M	6.5	IZINGA	Pos
969	8/30/12	M	20.0	KIKOKERA	Neg
970	8/30/12	F	32.0	KANYAN	Neg
971	8/30/12	M	20.0	BUGOYE	Neg
972	8/30/12	F	32.0	MALIBA	Neg
973	8/30/12	M	1.8	MALIBA	Neg
974	8/30/12	F	13.0	MALIBA	Neg
975	8/30/12	F	18.0	IZINGA	Neg
976	8/31/12	M	22.0	BUNYANGONI	Neg
977	8/31/12	M	18.0	MURAMBA	Neg
978	8/31/12	F	35.0	NDUGHUTU	Neg
979	8/31/12	M	10.0	RWAKING	Neg
980	8/31/12	F	50.0	KANYAN	Neg
981	8/31/12	F	1.3	MAGHOMA	Neg
982	8/31/12	F	50.0	RWAKING	Neg
983	9/1/12	F	31.0	MUHAMBO	Neg
984	9/1/12	F	12.0	RWAKING	Neg
985	9/1/12	M	41.0	MALIBA	Neg
986	9/1/12	M	31.0	KATOOKE	Neg
987	9/1/12	M	4.0	KYARUGOMOKA	Pos
988	9/1/12	F	29.0	MUHAMBO	Neg

989	9/1/12	M	6.0	BUGOYE	Neg
990	9/1/12	M	26.0	KYANYA	Neg
991	9/1/12	F	8.0	KANYAN	Pos
992	9/1/12	M	2.1	IHANI	Neg
993	9/1/12	M	30.0	BUGOYE	Neg
994	9/1/12	F	1.2	NYAKALENGIJO	Neg
995	9/1/12	M	22.0	IBANDA	Neg
996	9/1/12	F	7.0	BUNYANGONI	Neg
997	9/1/12	F	10.0	KANYAN	Neg
998	9/1/12	F	35.0	KISAMBA	Neg
999	9/1/12	M	22.0	KISAMBA	Neg
1000	9/1/12	M	58.0	KANYAN	Neg
1001	9/1/12	F	0.4	BUNYANGONI	Neg
1002	9/1/12	F	70.0	KATUMBA	Neg
1003	9/1/12	F	18.0	BUGOYE	Neg
1004	9/1/12	F	22.0	KYANYA	Neg
1005	9/4/12	M	22.0	IZINGA	Neg
1006	9/4/12	M	2.0	BUGOYE	Neg
1007	9/4/12	F	38.0	RWAKING	Neg
1008	9/4/12	F	1.1	KANYAN	Neg
1009	9/4/12	M	0.8	IZINGA	Pos
1010	9/4/12	F	41.0	KANYAN	Neg
1011	9/4/12	M	2.0	MURAMBA	Neg
1012	9/4/12	M	0.3	NYAKARUNGU	Neg
1013	9/4/12	M	3.5	IZINGA	Pos
1014	9/4/12	F	5.0	KAKINDO	Pos
1015	9/4/12	F	2.1	MURAMBA	Neg
1016	9/4/12	F	24.0	RWAKING	Pos
1017	9/4/12	F	1.5	KYARUGOMOKA	Neg
1018	9/5/12	F	25.0	MALIBA	Neg
1019	9/5/12	F	0.8	IHANI	Neg
1020	9/5/12	F	35.0	KATOOKE	Neg
1021	9/5/12	F	38.0	NYAKARUNGU	Neg
1022	9/5/12	F	45.0	MURAMBA	Neg
1023	9/6/12	F	15.0	KAYOMBE	Neg
1024	9/6/12	F	1.3	RWAKING	Pos
1025	9/6/12	F	35.0	RWAKING	Neg
1026	9/6/12	F	30.0	IZINGA	Neg
1027	9/6/12	M	10.0	IZINGA	Neg
1028	9/6/12	F	38.0	KANYAN	Neg
1029	9/6/12	M	24.0	RWAKING	Neg
1030	9/6/12	F	40.0	MALIBA	Neg
1031	9/6/12	M	58.0	NDUGHUTU	Neg

1032	9/6/12	F	1.1	MURAMBA	Neg
1033	9/6/12	M	9.0	KANYAN	Pos
1034	9/6/12	M	2.0	KANYAN	Neg
1035	9/6/12	F	10.0	IZINGA	Neg
1036	9/7/12	M	1.1	NDUGHUTU	Neg
1037	9/7/12	F	53.0	BUGOYE	Neg
1038	9/7/12	M	52.0	MURAMBA	Pos
1039	9/7/12	F	29.0	MALIBA	Neg
1040	9/7/12	M	9.0	KANYAN	Neg
1041	9/7/12	F	35.0	KANYAN	Neg
1042	9/7/12	M	0.5	KAKINDO	Pos
1043	9/7/12	F	13.0	MURAMBA	Neg
1044	9/7/12	M	9.0	IZINGA	Pos
1045	9/7/12	M	1.7	BUGOYE	Pos
1046	9/7/12	F	38.0	KYARUGOMOKA	Neg
1047	9/7/12	F	8.0	KIKOKERA	Pos
1048	9/7/12	F	28.0	KISANZI	Neg
1049	9/7/12	F	3.0	MALIBA	Neg
1050	9/7/12	F	3.0	MALIBA	Pos
1051	9/10/12	F	19.0	RWAKING	Neg
1052	9/10/12	F	16.0	NDUGHUTU	Neg
1053	9/10/12	F	15.0	BUGOYE	Neg
1054	9/10/12	F	30.0	NDUGHUTU	Neg
1055	9/10/12	M	1.3	MUHAMBO	Neg
1056	9/10/12	F	3.0	MALIBA	Neg
1057	9/10/12	M	50.0	MALIBA	Pos
1058	9/10/12	M	3.5	IZINGA	Neg
1059	9/10/12	F	4.0	IZINGA	Pos
1060	9/10/12	F	11.0	BUGOYE	Pos
1061	9/10/12	M	0.9	BUZIBWERA	Pos
1062	9/10/12	F	4.0	MURAMBA	Neg
1063	9/10/12	M	1.5	NYAKARUNGU	Neg
1064	9/10/12	F	40.0	IHANI	Pos
1065	9/10/12	F	6.0	KYARUGOMOKA	Pos
1066	9/10/12	F	2.5	KYARUGOMOKA	Pos
1067	9/10/12	F	7.0	KYARUGOMOKA	Pos
1068	9/11/12	F	35.0	IBANDA	Neg
1069	9/11/12	M	25.0	RWAKING	Pos
1070	9/11/12	M	2.3	KANYAN	Pos
1071	9/11/12	M	3.0	IZINGA	Pos
1072	9/11/12	F	35.0	BUGOYE	Neg
1073	9/11/12	M	0.3	KIHINDI	Pos
1074	9/11/12	F	11.0	KANYAN	Neg

1075	9/11/12	M	17.0	MURAMBA	Neg
1076	9/11/12	F	20.0	BUNYANGONI	Neg
1077	9/11/12	F	2.5	BUNYANGONI	Neg
1078	9/11/12	F	3.0	KYARUGOMOKA	Pos
1079	9/12/12	F	22.0	RWAKING	Neg
1080	9/12/12	M	0.8	MURAMBA	Neg
1081	9/12/12	F	9.0	NDUGHUTU	Neg
1082	9/12/12	F	22.0	NYAKARUNGU	Pos
1083	9/12/12	F	63.0	BULYANDEKE	Neg
1084	9/12/12	F	0.5	KANYAN	Neg
1085	9/12/12	F	53.0	MURAMBA	Neg
1086	9/12/12	F	4.0	MALIBA	Pos
1087	9/12/12	F	19.0	MUKATI	Neg
1088	9/12/12	F	56.0	KATOOKE	Neg
1089	9/12/12	F	4.3	MURAMBA	Neg
1090	9/12/12	M	12.0	KYARUGOMOKA	Neg
1091	9/12/12	F	1.5	IBANDA	Neg
1092	9/12/12	F	0.5	MUHAMBO	Neg
1093	9/12/12	F	22.0	MURAMBA	Neg
1094	9/13/12	F	6.3	RWAKING	Neg
1095	9/13/12	M	5.5	KANYAN	Pos
1096	9/13/12	M	9.0	IZINGA	Neg
1097	9/13/12	F	36.0	BUGOYE	Neg
1098	9/13/12	F	40.0	RWAKING	Neg
1099	9/13/12	F	18.0	KIKOKERA	Neg
1100	9/13/12	M	13.0	KANYAN	Pos
1101	9/13/12	M	6.0	KABUKERA	Neg
1102	9/13/12	M	8.0	KANYAN	Pos
1103	9/14/12	F	15.0	IBANDA	Neg
1104	9/14/12	F	44.0	NDUGHUTU	Neg
1105	9/14/12	F	31.0	MURAMBA	Neg
1106	9/14/12	F	58.0	KASESE	Neg
1107	9/14/12	M	2.5	NDUGHUTU	Neg
1108	9/14/12	M	0.1	KIKOKERA	Neg
1109	9/14/12	F	19.0	KANYAN	Neg
1110	9/14/12	M	15.0	MURAMBA	Neg
1111	9/14/12	M	15.0	BUNYANGONI	Neg
1112	9/14/12	F	0.2	IZINGA	Pos
1113	9/14/12	F	2.0	BUGOYE	Neg
1114	9/14/12	F	25.0	KIRONGO	Pos
1115	9/17/12	M	1.5	MURAMBA	Neg
1116	9/17/12	F	18.0	BUGOYE	Neg
1117	9/17/12	F	1.7	KANYAN	Neg

1118	9/17/12	F	17.0	KIBOTA	Neg
1119	9/17/12	F	12.0	BUNYANGONI	Neg
1120	9/17/12	M	49.0	BUGOYE	Pos
1121	9/17/12	F	19.0	MURAMBA	Neg
1122	9/17/12	F	7.0	NDUGHUTU	Neg
1123	9/17/12	M	12.0	BUGOYE	Neg
1124	9/17/12	F	20.0	IZINGA	Neg
1125	9/17/12	F	19.0	BUGOYE	Neg
1126	9/17/12	F	30.0	KANYAN	Neg
1127	9/17/12	M	0.5	MURAMBA	Neg
1128	9/17/12	F	35.0	IZINGA	Neg
1129	9/17/12	M	27.0	MUHAMBO	Neg
1130	9/17/12	F	1.3	BUNYANGONI	Pos
1131	9/17/12	F	3.0	BUNYANGONI	Pos
1132	9/17/12	F	0.8	BUNYANGONI	Pos
1133	9/17/12	F	2.5	BUNYANGONI	Pos
1134	9/17/12	F	2.0	NDUGHUTU	Neg
1135	9/17/12	M	1.5	KANYAN	Neg
1136	9/17/12	F	26.0	MAGHOMA	Neg
1137	9/17/12	M	0.3	KATOOKE	Neg
1138	9/17/12	F	36.0	IBANDA	Neg
1139	9/19/12	F	18.0	RWAKING	Pos
1140	9/19/12	M	0.7	KIHINDI	Neg
1141	9/19/12	F	5.0	MUHAMBO	Neg
1142	9/19/12	F	16.0	BUGOYE	Neg
1143	9/19/12	M	0.2	IBANDA	Neg
1144	9/19/12	F	16.0	BUNYANGONI	Pos
1145	9/19/12	F	15.0	IZINGA	Neg
1146	9/19/12	F	23.0	NYAKARUNGU	Neg
1147	9/19/12	F	27.0	KIRULI	Neg
1148	9/19/12	F	39.0	KIRULI	Neg
1149	9/19/12	M	16.0	RWAKING	Neg
1150	9/19/12	F	20.0	MUBUKU	Neg
1151	9/19/12	M	73.0	RWAKING	Neg
1152	9/20/12	M	32.0	KANYAN	Neg
1153	9/20/12	M	43.0	BUGOYE	Neg
1154	9/20/12	F	45.0	MALIBA	Neg
1155	9/20/12	M	2.5	NDUGHUTU	Pos
1156	9/20/12	F	1.2	IBANDA	Neg
1157	9/20/12	M	0.4	NDUGHUTU	Neg
1158	9/20/12	F	4.3	MURAMBA	Neg
1159	9/20/12	F	32.0	MALIBA	Neg
1160	9/20/12	M	1.4	KATOOKE	Neg

1161	9/21/12	F	25.0	MALIBA	Pos	
1162	9/21/12	F	13.0	BUGOYE	Neg	
1163	9/21/12	F	18.0	BUGOYE	Neg	
1164	9/21/12	F	45.0	KANYAN	Pos	
1165	9/21/12	M	1.2	BUZIBWERA	Pos	
1166	9/21/12	F	36.0	MURAMBA	Neg	
1167	9/21/12	F	27.0	NYAKARUNGU	Neg	
1168	9/21/12	F	0.4	MUHAMBO	Neg	
1169	9/21/12	F	0.8	NDUGHUTU	Neg	
1170	9/21/12	F	2.0	MUHAMBO	Neg	
1171	9/21/12	F	37.0	BUGOYE	Neg	
1172	9/21/12	F	2.5	RWAKING	Pos	
1173	9/21/12	M	1.1	IBANDA	Neg	
1174	9/24/12	F	13.0	KANYAN	Neg	
1175	9/24/12	F	20.0	MURAMBA	Neg	
1176	9/24/12	M	22.0	IBANDA	Neg	
1177	9/24/12	M	0.1	MURAMBA	Neg	
1178	9/24/12	F	2.0	RWAKING	Neg	
1179	9/24/12	F	2.4	MALIBA	Neg	
1180	9/24/12	F	15.0	KAKINDO	Neg	
1181	9/24/12	F	3.0	IBANDA	Neg	
1182	9/24/12	M	20.0	MURAMBA	Neg	
1183	9/24/12	F	30.0	KATUMBA	Neg	
1184	9/24/12	F	20.0	IHANI	Pos	
1185	9/24/12	M	0.6	MALIBA	Neg	
1186	9/24/12	F	14.0	RWAKING	Pos	
1187	9/25/12	F	44.0	BUGOYE	Neg	
1188	9/25/12	F	60.0	IZINGA	Pos	
1189	9/25/12	M	23.0	IBANDA	Neg	
1190	9/25/12	M	0.5	MURAMBA	Neg	
1191	9/25/12	F	18.0	MURAMBA	Neg	
1192	9/25/12	M	9.0	BUGOYE	Neg	
1193	9/25/12	M	4.0	KANYAN	Neg	
1194	9/25/12	M	36.0	BUGOYE	Neg	
1195	9/25/12	F	3.5	IZINGA	Neg	
1196	9/25/12	F	9.0	IZINGA	Pos	
1197	9/25/12	M	2.3	BUGOYE	Pos	
1198	9/25/12	F	0.0	MURAMBA	Neg	
1199	9/25/12	F	35.0	KYARUGOMOKA	Neg	Neg
1200	9/25/12	M	2.5	IZINGA	Pos	
1201	9/26/12	M	53.0	KANYAN	Neg	
1202	9/26/12	F	39.0	KAKINDO	Neg	
1203	9/26/12	F	0.8	KANYAN	Neg	

1204	9/26/12	F	40.0	BULYANDEKE	Neg
1205	9/26/12	M	21.0	MUHAMBO	Neg
1206	9/26/12	F	22.0	KIRONGO	Neg
1207	9/26/12	M	0.8	RWAKING	Pos
1208	9/26/12	F	10.0	KYARUGOMOKA	Neg
1209	9/26/12	F	19.0	BIKONE	Neg
1210	9/27/12	M	60.0	MALIBA	Neg
1211	9/27/12	M	11.0	BUGOYE	Neg
1212	9/27/12	F	22.0	IZINGA	Neg
1213	9/27/12	F	19.0	MURAMBA	Neg
1214	9/27/12	F	4.0	KANYAN	Pos
1215	9/27/12	F	24.0	KIKOKERA	Pos
1216	9/27/12	F	20.0	RWAKING	Neg
1217	9/27/12	F	1.5	RWAKING	Neg
1218	9/27/12	F	1.6	IBANDA	Neg
1219	9/27/12	F	27.0	RWAKING	Neg
1220	9/27/12	F	17.0	MAGHOMA	Neg
1221	9/27/12	F	18.0	MURAMBA	Neg
1222	9/27/12	F	25.0	MURAMBA	Neg
1223	9/27/12	M	21.0	BUNYANGONI	Pos
1224	9/28/12	F	5.5	KYARUGOMOKA	Pos
1225	9/28/12	F	26.0	KISANZI	Neg
1226	9/28/12	F	60.0	NDUGHUTU	Neg
1227	9/28/12	F	1.3	RWAKING	Neg
1228	9/28/12	M	49.0	BUGOYE	Neg
1229	9/28/12	F	4.0	BUGOYE	Neg
1230	9/28/12	F	0.8	BUGOYE	Neg
1231	9/28/12	F	10.0	RWAKING	Neg
1232	9/28/12	F	25.0	MURAMBA	Neg
1233	9/28/12	M	27.0	BUGOYE	Pos
1234	9/28/12	F	50.0	BUGOYE	Neg
1235	9/28/12	M	1.3	MURAMBA	Neg
1236	9/28/12	F	20.0	BUGOYE	Neg
1237	9/28/12	F	50.0	BIKONE	Neg
1238	9/28/12	M	4.0	RWAKING	Neg
1239	9/28/12	F	25.0	NDUGHUTU	Neg
1240	10/1/12	F	40.0	RWAKING	Neg
1241	10/1/12	M	14.0	BUGOYE	Neg
1242	10/1/12	F	17.0	MURAMBA	Neg
1243	10/1/12	F	17.0	IZINGA	Neg
1244	10/1/12	M	50.0	IZINGA	Neg
1245	10/1/12	F	60.0	BUNYANGONI	Neg
1246	10/1/12	F	4.0	KISANZI	Neg

1247	10/1/12	F	50.0	MALIBA	Neg
1248	10/1/12	F	37.0	BUNYANGONI	Neg
1249	10/1/12	M	14.0	MALIBA	Neg
1250	10/1/12	F	60.0	IBANDA	Neg
1251	10/1/12	M	0.3	MURAMBA	Neg
1252	10/1/12	F	3.8	IBANDA	Neg
1253	10/1/12	F	36.0	BUGOYE	Neg
1254	10/2/12	f	49.0	BUGOYE	Neg
1255	10/2/12	F	7.0	KINASATTA	Neg
1256	10/2/12	M	2.5	RWAKING	Neg
1257	10/2/12	F	16.0	BUGOYE	Neg
1258	10/2/12	F	20.0	MURAMBA	Neg
1259	10/2/12	M	2.0	NDUGHUTU	Neg
1260	10/2/12	F	14.0	IZINGA	Pos
1261	10/2/12	M	76.0	IZINGA	Neg
1262	10/2/12	M	1.8	MURAMBA	Neg
1263	10/2/12	M	38.0	MURAMBA	Neg
1264	10/2/12	F	22.0	NDUGHUTU	Neg
1265	10/2/12	F	25.0	MALIBA	Neg
1266	10/2/12	F	40.0	RUBONI	Neg
1267	10/2/12	F	51.0	BUNYANGONI	Neg
1268	10/3/12	M	3.6	RWAKING	Neg
1269	10/3/12	F	40.0	MALIBA	Neg
1270	10/3/12	F	14.0	IBANDA	Neg
1271	10/3/12	F	25.0	BUNYANGONI	Neg
1272	10/3/12	M	35.0	MUBUKU	Neg
1273	10/3/12	M	60.0	BUNYANGONI	Neg
1274	10/3/12	F	23.0	KIGORO	Neg
1275	10/4/12	M	19.0	IBANDA	Neg
1276	10/4/12	F	42.0	RWAKING	Neg
1277	10/4/12	F	18.0	BUGOYE	Pos
1278	10/4/12	F	2.8	RWAKING	Neg
1279	10/4/12	F	22.0	BUNYANGONI	Neg
1280	10/4/12	F	8.0	BUGOYE	Pos
1281	10/5/12	F	18.0	KATUMBA	Neg
1282	10/5/12	F	28.0	BUNYANGONI	Neg
1283	10/5/12	M	25.0	RWAKING	Neg
1284	10/5/12	M	0.8	MURAMBA	Neg
1285	10/5/12	F	17.0	MURAMBA	Neg
1286	10/5/12	M	1.0	MALIBA	Neg
1287	10/5/12	M	0.8	BUGOYE	Neg
1288	10/8/12	M	4.0	KANYAN	Pos
1289	10/8/12	F	2.0	BUNYANGONI	Neg

1290	10/8/12	M	22.0	BUGOYE	Neg
1291	10/8/12	F	37.0	MURAMBA	Pos
1292	10/8/12	F	30.0	BUGOYE	Neg
1293	10/8/12	F	0.5	KANYAN	Neg
1294	10/8/12	F	28.0	BUGOYE	Neg
1295	10/8/12	F	20.0	BUNYANGONI	Neg
1296	10/8/12	M	19.0	MAGHOMA	Neg
1297	10/8/12	F	3.5	MURAMBA	Neg
1298	10/8/12	FF	1.8	MURAMBA	Neg
1299	10/8/12	M	0.2	MURAMBA	Neg
1300	10/8/12	F	30.0	MURAMBA	Neg
1301	10/8/12	F	2.0	BUNYANGONI	Neg
1302	10/8/12	M	0.5	KANYAN	Pos
1303	10/8/12	F	3.0	NDUGHUTU	Neg
1304	10/8/12	M	6.0	KIRULI	Neg
1305	10/8/12	F	17.0	IZINGA	Neg
1306	10/8/12	F	38.0	NDUGHUTU	Neg
1307	10/8/12	M	0.7	BUNYANGONI	Neg
1308	10/10/12	F	0.8	BUGOYE	Neg
1309	10/10/12	F	5.0	KIKOKERA	Neg
1310	10/10/12	M	0.7	NDUGHUTU	Neg
1311	10/10/12	M	0.7	BUNYANGONI	Neg
1312	10/10/12	F	17.0	KISOJO	Pos
1313	10/11/12	F	69.0	BUGOYE	Neg
1314	10/11/12	F	5.0	MURAMBA	Neg
1315	10/11/12	M	2.0	NDUGHUTU	Neg
1316	10/11/12	F	45.0	RWAKING	Neg
1317	10/12/12	F	13.0	NDUGHUTU	Neg
1318	10/12/12	M	35.0	KANYAN	Neg
1319	10/12/12	M	30.0	IZINGA	Neg
1320	10/12/12	M	0.3	MURAMBA	Neg
1321	10/12/12	M	20.0	MURAMBA	Neg
1322	10/12/12	F	26.0	IZINGA	Neg
1323	10/12/12	M	0.6	MUHAMBO	Neg
1324	10/12/12	M	50.0	MUHAMBO	Neg
1325	10/12/12	M	20.0	KIHARARA	Neg
1326	10/15/12	F	1.6	MURAMBA	Neg
1327	10/15/12	F	30.0	MURAMBA	Neg
1328	10/15/12	F	21.0	MURAMBA	Neg
1329	10/15/12	F	9.0	IZINGA	Pos
1330	10/15/12	F	30.0	KATUMBA	Neg
1331	10/15/12	M	27.0	MURAMBA	Neg
1332	10/15/12	F	35.0	MURAMBA	Neg

1333	10/15/12	M	4.0	KIKOKERA	Neg
1334	10/15/12	M	0.6	KIKOKERA	Neg
1335	10/15/12	F	17.0	BUGOYE	Neg
1336	10/15/12	F	1.5	ISULE	Neg
1337	10/15/12	M	.	MURAMBA	Neg
1338	10/15/12	F	17.0	BUGOYE	Neg
1339	10/15/12	M	1.3	KANYAN	Neg
1340	10/15/12	F	40.0	BUGOYE	Neg
1341	10/15/12	F	26.0	BUZIBWERA	Neg
1342	10/15/12	F	13.0	KISAMBA	Neg
1343	10/15/12	M	12.0	IZINGA	Pos
1344	10/15/12	M	0.2	BUGOYE	Neg
1345	10/15/12	F	19.0	MURAMBA	Neg
1346	10/15/12	M	60.0	BUGOYE	Neg
1347	10/15/12	M	3.4	NDUGHUTU	Neg
1348	10/15/12	M	0.2	NDUGHUTU	Neg
1349	10/15/12	F	32.0	KANYAN	Neg
1350	10/15/12	F	29.0	BUGOYE	Neg
1351	10/16/12	F	42.0	BUGOYE	Neg
1352	10/16/12	M	1.4	IHANI	Neg
1353	10/16/12	M	34.0	KANYAN	Neg
1354	10/16/12	M	3.5	IHANI	Neg
1355	10/16/12	M	20.0	MURAMBA	Neg
1356	10/16/12	F	12.0	BUGOYE	Pos
1357	10/16/12	F	20.0	MALIBA	Neg
1358	10/16/12	M	1.0	NDUGHUTU	Pos
1359	10/16/12	F	10.0	KISANZI	Neg
1360	10/16/12	F	4.0	BUGOYE	Pos
1361	10/16/12	M	20.0	BUGOYE	Neg
1362	10/16/12	M	24.0	RWAKING	Neg
1363	10/17/12	F	1.6	BUGOYE	Neg
1364	10/17/12	F	43.0	NDUGHUTU	Neg
1365	10/17/12	F	0.8	BUGOYE	Neg
1366	10/17/12	M	30.0	KISAMBA	Pos
1367	10/17/12	F	10.0	MURAMBA	Neg
1368	10/17/12	F	0.5	KISAMBA	Neg
1369	10/17/12	F	36.0	MUHAMBO	Neg
1370	10/17/12	F	28.0	BUGOYE	Neg
1371	10/17/12	M	2.0	MURAMBA	Neg
1372	10/17/12	F	18.0	BUGOYE	Neg
1373	10/18/12	M	24.0	KANYAN	Pos
1374	10/18/12	F	6.0	BUNYANGONI	Neg
1375	10/18/12	F	40.0	NDUGHUTU	Neg

1376	10/18/12	M	1.5	MUHAMBO	Neg
1377	10/18/12	F	36.0	IBANDA	Neg
1378	10/18/12	M	6.0	KATUMBA	Neg
1379	10/18/12	F	10.0	KATUMBA	Neg
1380	10/19/12	M	0.5	MURAMBA	Neg
1381	10/19/12	M	2.3	BUNYANGONI	Neg
1382	10/19/12	F	45.0	IZINGA	Neg
1383	10/19/12	F	0.3	NYAKARUNGU	Neg
1384	10/19/12	M	24.0	BUNYANGONI	Neg
1385	10/19/12	M	4.0	NDUGHUTU	Neg
1386	10/19/12	M	39.0	KYARUGOMOKA	Pos
1387	10/19/12	F	7.0	KYARUGOMOKA	Pos
1388	10/19/12	F	20.0	KANYAN	Neg
1389	10/19/12	M	0.8	MBUMURO	Neg
1390	10/19/12	F	15.0	MALIBA	Neg
1391	10/19/12	F	30.0	MALIBA	Neg
1392	10/22/12	F	31.0	BUGOYE	Neg
1393	10/22/12	F	1.9	BUGOYE	Neg
1394	10/22/12	M	5.0	BUGOYE	Neg
1395	10/22/12	F	28.0	BUGOYE	Neg
1396	10/22/12	M	3.0	IBANDA	Neg
1397	10/22/12	F	33.0	RWAKING	Neg
1398	10/22/12	F	32.0	RWAKING	Neg
1399	10/22/12	F	13.0	KAKINDO	Neg
1400	10/22/12	F	60.0	BUGOYE	Neg
1401	10/22/12	M	0.9	BUGOYE	Neg
1402	10/22/12	M	0.7	BUGOYE	Neg
1403	10/22/12	F	0.4	MURAMBA	Neg
1404	10/22/12	M	2.3	KISAMBA	Neg
1405	10/22/12	M	22.0	KISAMBA	Neg
1406	10/22/12	M	0.8	IBANDA	Neg
1407	10/24/12	F	30.0	KANYAN	Neg
1408	10/24/12	M	33.0	NYAKARUNGU	Neg
1409	10/24/12	F	51.0	KANYAN	Neg
1410	10/24/12	F	30.0	KISAMBA	Neg
1411	10/24/12	F	22.0	KATOOKE	Neg
1412	10/24/12	M	52.0	KISOJO	Neg
1413	10/24/12	F	59.0	IBANDA	Neg
1414	10/24/12	F	19.0	BUGOYE	Neg
1415	10/24/12	M	49.0	BUGOYE	Neg
1416	10/24/12	F	18.0	.	Neg
1417	10/24/12	F	20.0	MALIBA	Neg
1418	10/24/12	F	11.0	IZINGA	Neg

1419	10/25/12	M	18.0	BUGOYE	Pos
1420	10/25/12	F	26.0	MALIBA	Neg
1421	10/25/12	F	1.1	BUGOYE	Neg
1422	10/25/12	F	23.0	NDUGHUTU	Neg
1423	10/25/12	M	25.0	BUGOYE	Pos
1424	10/25/12	F	27.0	MALIBA	Neg
1425	10/26/12	F	18.0	.	Neg
1426	10/26/12	F	21.0	IZINGA	Neg
1427	10/26/12	F	1.1	BUGOYE	Pos
1428	10/26/12	M	6.5	IZINGA	Neg
1429	10/26/12	M	0.7	IZINGA	Neg
1430	10/26/12	F	0.3	IZINGA	Neg
1431	10/26/12	F	0.8	BUNYANGONI	Neg
1432	10/26/12	M	67.0	BUNSONGORA	Neg
1433	10/29/12	M	1.1	KANYAN	Neg
1434	10/29/12	M	7.0	MURAMBA	Neg
1435	10/29/12	F	20.0	NYAKARUNGU	Neg
1436	10/29/12	F	1.5	BUGOYE	Neg
1437	10/29/12	M	17.0	BUGOYE	Neg
1438	10/29/12	F	1.8	MURAMBA	Neg
1439	10/29/12	F	50.0	KIRULI	Neg
1440	10/29/12	F	38.0	KIRULI	Neg
1441	10/29/12	F	0.3	MURAMBA	Neg
1442	10/29/12	F	20.0	BUNYANGONI	Neg
1443	10/29/12	F	54.0	IZINGA	Neg
1444	10/29/12	F	9.0	RWAKING	Neg
1445	10/29/12	F	30.0	NDUGHUTU	Neg
1446	10/29/12	M	35.0	MALIBA	Neg
1447	10/29/12	F	18.0	MALIBA	Neg
1448	10/29/12	M	1.5	BUGOYE	Neg
1449	10/30/12	F	19.0	KANYAN	Neg
1450	10/30/12	F	40.0	MURAMBA	Neg
1451	10/30/12	F	45.0	KYANYA	Neg
1452	10/30/12	F	17.0	NYAKABUGHA	Neg
1453	10/30/12	M	3.5	KANYAN	Pos
1454	10/30/12	F	20.0	RWAKING	Neg
1455	10/30/12	F	0.5	KISENDERA	Neg
1456	10/30/12	F	27.0	BUGOYE	Neg
1457	10/30/12	M	1.3	NDUGHUTU	Neg
1458	10/30/12	F	24.0	BUGOYE	Neg
1459	10/30/12	M	55.0	IBANDA	Neg
1460	10/30/12	F	0.8	MURAMBA	Neg
1461	10/31/12	M	23.0	MUBUKU	Neg

1462	10/31/12	F	17.0	MUKATI	Neg	
1463	10/31/12	F	30.0	MALIBA	Neg	
1464	10/31/12	M	24.0	KATUMBA	Neg	Neg
1465	10/31/12	F	37.0	MALIBA	Neg	Neg
1466	10/31/12	M	1.0	MURAMBA	Neg	Neg
1467	10/31/12	F	7.0	MURAMBA	Neg	Neg
1468	10/31/12	M	8.0	IZINGA	Neg	Neg
1469	11/1/12	F	7.0	BUGOYE	Neg	
1470	11/1/12	M	49.0	KISAMBA	Neg	
1471	11/1/12	F	18.0	BUGOYE	Neg	
1472	11/1/12	M	7.0	BUNYANGONI	Neg	
1473	11/1/12	F	0.6	MALIBA	Neg	
1474	11/1/12	M	6.0	RWAKING	Neg	
1475	11/1/12	F	18.0	MURAMBA	Neg	
1476	11/1/12	F	22.0	NYAKARUNGU	Neg	
1477	11/1/12	M	4.5	BUGOYE		Pos
1478	11/1/12	F	20.0	BUGOYE	Neg	
1479	11/1/12	M	2.0	IHANI	Neg	
1480	11/1/12	M	2.0	IHANI	Neg	
1481	11/1/12	M	4.5	BUGOYE	Neg	
1482	11/2/12	F	0.8	BUGOYE	Neg	
1483	11/2/12	F	22.0	KAKINDO	Neg	
1484	11/2/12	F	37.0	MURAMBA	Neg	
1485	11/2/12	F	0.6	BUGOYE	Neg	
1486	11/2/12	M	4.0	.	Neg	
1487	11/2/12	F	18.0	IBANDA	Neg	
1488	11/2/12	M	0.7	IBANDA	Neg	
1489	11/2/12	M	1.3	IBANDA	Neg	
1490	11/5/12	M	40.0	KYANYA	Pos	
1491	11/5/12	F	2.0	BUNYANGONI	Pos	
1492	11/5/12	F	11.0	KAKINDO	Neg	
1493	11/5/12	M	25.0	MURAMBA	Neg	
1494	11/5/12	F	23.0	NDUGHUTU	Neg	
1495	11/5/12	F	37.0	BUGOYE	Neg	
1496	11/5/12	M	3.5	KAKINDO	Neg	
1497	11/5/12	F	19.0	KANYAN	Neg	
1498	11/5/12	F	31.0	IBANDA	Neg	
1499	11/5/12	F	55.0	MALIBA	Neg	
1500	11/5/12	F	30.0	MURAMBA	Neg	
1501	11/6/12	F	20.0	MURAMBA		Pos
1502	11/6/12	F	27.0	IBANDA	Neg	
1503	11/6/12	F	38.0	IZINGA	Pos	
1504	11/6/12	F	24.0	BUGOYE	Neg	

1505	11/6/12	F	14.0	KIKOKERA	Neg	
1506	11/6/12	M	8.0	KIKOKERA	Neg	
1507	11/6/12	F	6.0	NYAKARUNGU	Pos	
1508	11/6/12	M	1.5	NYAKARUNGU	Pos	
1509	11/6/12	F	37.0	BUGOYE	Neg	
1510	11/7/12	F	35.0	BUGOYE	Neg	
1511	11/7/12	M	25.0	MURAMBA	Neg	
1512	11/7/12	F	3.0	MALIBA	Neg	
1513	11/7/12	M	0.7	BUGOYE	Neg	
1514	11/7/12	M	8.0	BULINDUGURU	Neg	
1515	11/8/12	M	72.0	BUGOYE	Neg	
1516	11/8/12	F	39.0	MALIBA	Neg	
1517	11/8/12	M	18.0	BUNYANGONI	Neg	
1518	11/8/12	F	36.0	RWAKING	Neg	
1519	11/8/12	F	10.0	IZINGA	Neg	
1520	11/8/12	F	30.0	MAGHOMA	Neg	
1521	11/8/12	F	10.0	MURAMBA	Neg	
1522	11/8/12	F	21.0	KYANDALE	Neg	
1523	11/8/12	F	71.0	RWAKING	Neg	
1524	11/8/12	F	30.0	IZINGA	Neg	
1525	11/9/12		28.0	NDUGHUTU	Neg	
1526	11/9/12	M	70.0	RWAKING	Neg	
1527	11/9/12	F	17.0	IHANI	Neg	
1528	11/9/12	M	3.0	KIRULI	Neg	
1529	11/9/12	F	19.0	KATOOKE	Neg	
1530	11/9/12	M	80.0	MALIBA	Neg	
1531	11/9/12	M	0.8	MURAMBA	Neg	
1532	11/9/12	F	0.7	KATOOKE	Neg	
1533	11/9/12	F	25.0	KISANGA	Neg	
1534	11/9/12	F	22.0	MURAMBA	Neg	
1535	11/12/12	M	24.0	BUGOYE		Neg
1536	11/12/12	F	26.0	KYANDALE	Neg	
1537	11/12/12	M	30.0	IBANDA	Neg	
1538	11/12/12	F	30.0	IBANDA		
1539	11/12/12	F	22.0	MURAMBA	Neg	
1540	11/12/12	M	39.0	NDUGHUTU	Neg	
1541	11/12/12	M	45.0	BUGOYE	Neg	
1542	11/12/12	F	22.0	KATOOKE	Neg	
1543	11/12/12	M	1.7	KANYAN	Neg	
1544	11/12/12	M	3.0	KISAMBA	Neg	
1545	11/13/12	M	13.0	NDUGHUTU	Neg	
1546	11/13/12	M	14.0	BUGOYE	Neg	
1547	11/13/12	M	2.2	MALIBA	Neg	

1548	11/13/12	F	13.0	RWAKING	Neg	
1549	11/13/12	F	21.0	BUGOYE	Neg	
1550	11/13/12	F	35.0	KANYAN	Neg	
1551	11/13/12	F	28.0	KYANDALE	Neg	
1552	11/13/12	F	45.0	IZINGA	Neg	
1553	11/13/12	M	1.2	MALIBA	Neg	Neg
1554	11/13/12	F	19.0	KYARUGOMOKA	Neg	
1555	11/13/12	M	30.0	KIRONGO	Neg	
1556	11/14/12		1.4	KANYAN	Neg	
1557	11/14/12	F	40.0	MALIBA	Neg	
1558	11/14/12	M	7.0	MURAMBA	Neg	
1559	11/14/12	F	35.0	MALIBA	Neg	
1560	11/14/12	F	30.0	MALIBA	Neg	
1561	11/14/12	F	0.3	BUGOYE	Neg	
1562	11/14/12	F	18.0	KIKOKERA	Neg	
1563	11/14/12	M	35.0	MALIBA	Neg	
1564	11/14/12	M	26.0	BUGOYE	Pos	
1565	11/14/12	F	34.0	KIKOKERA	Neg	
1566	11/14/12	M	38.0	BUZIBWERA	Neg	
1567	11/14/12	M	2.5	KABUGHO	Pos	
1568	11/14/12	F	3.0	NDUGHUTU	Neg	Neg
1569	11/14/12	F	4.0	BUNYANGONI	Neg	
1570	11/14/12	F	17.0	NDUGHUTU	Neg	
1571	11/14/12	F	18.0	BUGOYE	Neg	
1572	11/14/12	M	2.3	IBANDA	Neg	
1573	11/14/12	F	32.0	BUGOYE	Neg	
1574	11/14/12	F	25.0	KIRONGO	Neg	
1575	11/16/12	M	0.9	IBANDA	Neg	
1576	11/16/12	F	3.0	MURAMBA	Neg	
1577	11/16/12	F	12.0	NDUGHUTU	Neg	
1578	11/19/12	F	21.0	MURAMBA	Neg	
1579	11/19/12	M	2.8	MURAMBA	Neg	
1580	11/19/12	F	23.0	IZINGA	Neg	
1581	11/19/12	M	23.0	NDUGHUTU	Neg	
1582	11/19/12	F	28.0	KIKOKERA	Neg	
1583	11/19/12	M	20.0	NYAKARUNGU	Pos	
1584	11/19/12	M	2.0	MURAMBA	Neg	
1585	11/19/12	M	5.5	MURAMBA	Neg	
1586	11/20/12	F	5.0	MALIBA	Neg	
1587	11/20/12	F	1.3	BUGOYE	Neg	
1588	11/20/12	F	8.0	MURAMBA	Neg	
1589	11/20/12	F	1.5	MURAMBA	Neg	
1590	11/20/12	M	7.0	NDUGHUTU	Neg	

1591	11/20/12	F	0.1	NDUGHUTU	Neg
1592	11/20/12	F	0.8	MURAMBA	Neg
1593	11/20/12	F	59.0	NDUGHUTU	Neg
1594	11/20/12	M	3.0	NDUGHUTU	Neg
1595	11/20/12	M	34.0	KIRONGO	Neg
1596	11/20/12	F	0.4	IHANI	Neg
1597	11/21/12	F	23.0	IZINGA	Neg
1598	11/21/12	F	0.3	IZINGA	Neg
1599	11/21/12	M	7.0	IBANDA	Neg
1600	11/21/12	M	1.0	BUNYANGONI	Neg
1601	11/21/12	M	2.0	BUNYANGONI	Neg
1602	11/21/12	M	1.0	KIRONGO	Neg
1603	11/21/12	M	28.0	IZINGA	Neg
1604	11/21/12	M	1.3	BUGOYE	Neg
1605	11/21/12	F	0.6	MURAMBA	Neg
1606	11/21/12	M	55.0	KANYAN	Neg
1607	11/21/12	F	1.7	NDUGHUTU	Neg
1608	11/21/12	M	17.0	KAKINDO	Neg
1609	11/21/12	F	50.0	IBANDA	Neg
1610	11/22/12	F	60.0	BUGOYE	Neg
1611	11/22/12	F	22.0	IZINGA	Neg
1612	11/22/12	F	15.0	KANYAN	Neg
1613	11/22/12	F	5.0	KANYAN	Neg
1614	11/22/12	F	1.6	BUGOYE	Neg
1615	11/22/12	F	25.0	KANYAN	Neg
1616	11/22/12	F	9.0	RWAKING	Pos
1617	11/22/12	F	49.0	RWAKING	Neg
1618	11/22/12	M	2.0	BUNYANGONI	Neg
1619	11/22/12	M	0.7	KIHINDI	Neg
1620	11/22/12	F	15.0	BUZIBWERA	Neg
1621	11/22/12	F	1.3	IZINGA	Pos
1622	11/22/12	F	19.0	KATUMBA	Neg
1623	11/22/12	M	8.0	MALIBA	Pos
1624	11/23/12	M	1.1	KANYAN	Neg
1625	11/23/12	M	2.0	MALIBA	Pos
1626	11/23/12	M	4.0	MURAMBA	Neg
1627	11/23/12	M	4.0	BUGOYE	Neg
1628	11/23/12	F	1.7	IZINGA	Pos
1629	11/23/12	M	30.0	NDUGHUTU	Neg
1630	11/27/12	F	2.0	IZINGA	Pos
1631	11/27/12	M	2.9	KANYAN	Pos
1632	11/27/12	M	0.6	NYAKARUNGU	Neg
1633	11/27/12	F	4.0	KYARUGOMOKA	Neg

1634	11/27/12	M	3.0	BUGOYE	Pos
1635	11/27/12	F	22.0	MUBUKU	Neg
1636	11/27/12	M	1.2	KYARUGOMOKA	Neg
1637	11/28/12	F	46.0	BUNYANGONI	Neg
1638	11/28/12	M	27.0	MURAMBA	Neg
1639	11/28/12	M	35.0	NDUGHUTU	Neg
1640	11/28/12	F	40.0	KAKINDO	Neg
1641	11/28/12	F	70.0	KATOOKE	Neg
1642	11/28/12	F	83.0	IBANDA	Neg
1643	11/28/12	M	1.5	IHANI	Neg
1644	11/28/12	F	32.0	NDUGHUTU	Neg
1645	11/28/12	M	2.5	BUNYANGONI	Neg
1646	11/28/12	F	45.0	IBANDA	Neg
1647	11/28/12	F	40.0	KIRULI	Neg
1648	11/28/12	F	12.0	MALIBA	Neg
1649	11/28/12	F	20.0	IBANDA	Neg
1650	11/28/12	F	23.0	KANYAN	Neg
1651	11/28/12	F	18.0	MALIBA	Neg
1652	11/28/12	M	2.0	KANYAN	Pos
1653	11/28/12	M	2.5	MURAMBA	Neg
1654	11/28/12	F	23.0	BUNYANGONI	Neg
1655	12/3/12	M	22.0	MALIBA	Pos
1656	12/3/12	M	36.0	BUGOYE	Neg
1657	12/3/12	F	25.0	IBANDA	Neg
1658	12/3/12	M	1.5	MURAMBA	Neg
1659	12/3/12	M	1.2	KYANYA	Neg
1660	12/3/12	M	24.0	BUNYANGONI	Neg
1661	12/3/12	F	25.0	KANYAN	Neg
1662	12/3/12	F	1.0	MURAMBA	Neg
1663	12/3/12	F	5.0	KAKINDO	Neg
1664	12/3/12	F	3.0	MALIBA	Neg
1665	12/3/12	M	2.0	MURAMBA	Neg
1666	12/3/12	M	36.0	BUGOYE	Neg
1667	12/4/12	M	3.1	KANYAN	Pos
1668	12/4/12	M	22.0	BUGOYE	Neg
1669	12/4/12	M	43.0	BUNYANGONI	Neg
1670	12/4/12	F	29.0	BUGOYE	Neg
1671	12/4/12	F	3.0	BUGOYE	Neg
1672	12/4/12	M	0.1	BUGOYE	Neg
1673	12/4/12	M	1.9	KANYAN	Neg
1674	12/4/12	F	4.0	KANYAN	Neg
1675	12/4/12	F	22.0	KANYAN	Neg
1676	12/4/12	M	46.0	MURAMBA	Neg

1677	12/4/12	M	2.0	KANYAN	Pos	
1678	12/4/12	M	28.0	NDUGHUTU	Pos	
1679	12/6/12	F	1.6	KIRONGO	Pos	
1680	12/6/12	F	24.0	KANYAN	Neg	
1681	12/6/12	F	.	BUGOYE	Neg	
1682	12/6/12	F	27.0	IBANDA	Neg	
1683	12/6/12	F	6.0	NYAKARUNGU	Pos	
1684	12/6/12	F	13.0	BUGOYE	Pos	
1685	12/6/12	F	20.0	BUGOYE	Neg	
1686	12/6/12	M	13.0	KANYAN	Pos	
1687	12/6/12	F	80.0	BUGOYE	Neg	
1688	12/6/12	F	4.3	MURAMBA	Neg	
1689	12/6/12	F	2.0	RWAKING	Neg	
1690	12/6/12	F	35.0	NDUGHUTU	Neg	
1691	12/6/12	F	17.0	BUGOYE	Neg	
1692	12/6/12	F	4.0	NDUGHUTU	Pos	
1693	12/6/12	M	9.0	KANYAN	Pos	
1694	12/6/12	M	13.0	KANYAN	Pos	
1695	12/7/12	M	54.0	BUNYANGONI	Neg	
1696	12/7/12	F	5.2	BUNYANGONI	Neg	
1697	12/7/12	F	74.0	BUNYANGONI	Neg	
1698	12/7/12	F	25.0	NDUGHUTU	Neg	
1699	12/10/12	F	24.0	KYANYA	Neg	
1700	12/10/12	M	28.0	BUGOYE	Neg	
1701	12/10/12	F	4.0	NDUGHUTU	Pos	Neg
1702	12/10/12	F	18.0	KANYAN	Neg	
1703	12/10/12	F	18.0	MURAMBA	Neg	
1704	12/10/12	M	34.0	NDUGHUTU	Neg	
1705	12/10/12	F	32.0	MALIBA	Pos	
1706	12/10/12	M	8.5	MURAMBA	Neg	
1707	12/10/12	F	32.0	BUNYANGONI	Neg	
1708	12/10/12	F	42.0	MALIBA	Neg	
1709	12/10/12	F	1.3	BUGOYE	Pos	
1710	12/10/12	M	1.2	MALIBA	Neg	
1711	12/10/12	M	1.0	KATOOKE	Neg	
1712	12/10/12	F	42.0	MALIBA	Neg	
1713	12/10/12	F	10.0	BUGOYE	Neg	
1714	12/11/12	F	35.0	RWAKING	Neg	
1715	12/11/12	M	20.0	MAGHOMA	Neg	
1716	12/11/12	M	1.8	BUGOYE	Pos	
1717	12/11/12	F	17.0	RWAKING	Neg	
1718	12/11/12	M	17.0	BUGOYE	Neg	
1719	12/11/12	M	18.0	RWAKING	Neg	

1720	12/11/12	F	0.4	MURAMBA	Neg
1721	12/11/12	F	3.0	KANYAN	Pos
1722	12/11/12	F	35.0	KATUMBA	Neg
1723	12/11/12	M	1.0	BUGOYE	Neg
1724	12/11/12	F	1.6	KANYAN	Neg
1725	12/11/12	F	22.0	MALIBA	Neg
1726	12/11/12	M	30.0	NYAKABUGHA	Neg
1727	12/12/12	F	1.0	.	Neg
1728	12/12/12	F	28.0	KYARUGOMOKA	Neg
1729	12/12/12	F	48.0	MURAMBA	Neg
1730	12/12/12	F	40.0	NDUGHUTU	Neg
1731	12/12/12	F	0.5	MURAMBA	Neg
1732	12/12/12	F	4.1	KIBOTA	Neg
1733	12/12/12	M	20.0	BUGOYE	Neg
1734	12/12/12	F	39.0	BUNYANGONI	Neg
1735	12/12/12	M	2.2	NDUGHUTU	Neg
1736	12/12/12	F	9.0	MALIBA	Neg
1737	12/12/12	M	25.0	KISOJO	Neg
1738	12/12/12	M	0.8	KANYAN	Neg
1739	12/12/12	F	28.0	MURAMBA	Neg
1740	12/12/12	M	2.0	KISANZI	Pos
1741	12/12/12	F	19.0	MAGHOMA	Pos
1742	12/12/12	F	4.0	MALIBA	Neg
1743	12/12/12	F	2.0	BUGOYE	Neg
1744	12/12/12	F	38.0	NYAKARUNGU	Neg
1745	12/12/12	F	40.0	RWAKING	Neg
1746	12/12/12	M	0.3	MURAMBA	Neg
1747	12/12/12	M	3.2	MALIBA	Neg
1748	12/13/12	M	2.0	RWAKING	Pos
1749	12/13/12	F	5.0	RWAKING	Neg
1750	12/13/12	F	1.0	KANYAN	Pos
1751	12/13/12	F	23.0	NDUGHUTU	Neg
1752	12/13/12	F	52.0	MALIBA	Neg
1753	12/13/12	F	4.0	BUZIBWERA	Pos
1754	12/13/12	M	1.5	MALIBA	Pos
1755	12/13/12	M	49.0	KAKINDO	Neg
1756	12/13/12	M	25.0	BUGOYE	Neg
1757	12/13/12	F	0.7	NDUGHUTU	Neg
1758	12/13/12	F	1.5	MALIBA	Neg
1759	12/13/12	M	0.6	MALIBA	Pos
1760	12/13/12	M	0.8	MALIBA	Neg
1761	12/13/12	F	4.5	KAKINDO	Neg
1762	12/13/12	F	0.8	KAKINDO	Neg

1763	12/13/12	F	0.7	BUGOYE	Neg
1764	12/13/12	F	10.0	BUGOYE	Pos
1765	12/14/12	F	30.0	BUGOYE	Neg
1766	12/14/12	M	0.6	MURAMBA	Neg
1767	12/14/12	M	51.0	BUGOYE	Neg
1768	12/14/12	F	0.5	NDUGHUTU	Neg
1769	12/14/12	F	3.0	MALIBA	Neg
1770	12/14/12	F	35.0	IBANDA	Neg
1771	12/14/12	F	30.0	RWAKING	Neg
1772	12/14/12	F	45.0	MUKATI	Neg
1773	12/14/12	F	19.0	NDUGHUTU	Neg
1774	12/14/12	M	11.0	BUGOYE	Neg
1775	12/14/12	M	0.7	MURAMBA	Neg
1776	12/14/12	M	18.0	KANYAN	Pos
1777	12/14/12	F	20.0	RWAKING	Neg
1778	12/14/12	F	16.0	KANYAN	Neg
1779	12/17/12	M	0.9	KANYAN	Neg
1780	12/17/12	F	33.0	RWAKING	Neg
1781	12/17/12	M	8.0	NDUGHUTU	Neg
1782	12/17/12	F	7.0	MALIBA	Neg
1783	12/17/12	F	3.0	MALIBA	Neg
1784	12/17/12	F	17.0	KANYAN	Pos
1785	12/17/12	F	21.0	MALIBA	Neg
1786	12/17/12	F	18.0	IHANI	Neg
1787	12/17/12	M	2.5	IZINGA	Neg
1788	12/17/12	M	1.3	NDUGHUTU	Neg
1789	12/17/12	F	4.0	KANYAN	Pos
1790	12/17/12	F	16.0	NYAKARUNGU	Pos
1791	12/17/12	M	1.6	MURAMBA	Neg
1792	12/17/12	F	35.0	MURAMBA	Neg
1793	12/17/12	F	25.0	KATUMBA	Neg
1794	12/17/12	F	8.0	NDUGHUTU	Neg
1795	12/17/12	F	18.0	MURAMBA	Neg
1796	12/17/12	F	7.0	KANYAN	Pos
1797	12/17/12	M	2.0	KANYAN	Pos
1798	12/17/12	F	22.0	NDUGHUTU	Neg
1799	12/17/12	F	18.0	NYAKARUNGU	Neg
1800	12/17/12	M	1.3	MURAMBA	Neg
1801	12/18/12	F	24.0	NDUGHUTU	Neg
1802	12/18/12	F	50.0	IBANDA	Neg
1803	12/18/12	M	40.0	KATOOKE	Neg
1804	12/18/12	F	18.0	KANYAN	Neg
1805	12/18/12	F	19.0	MURAMBA	Neg

1806	12/18/12	F	28.0	RWAKING	Neg	
1807	12/18/12	F	40.0	KANYAN	Pos	
1808	12/18/12	M	1.5	MALIBA	Pos	
1809	12/18/12	F	2.5	MURAMBA	Pos	
1810	12/18/12	F	40.0	MURAMBA	Neg	
1811	12/18/12	F	1.3	RWAKING	Neg	
1812	12/18/12	F	17.0	MALIBA	Neg	
1813	12/18/12	M	7.0	MURAMBA	Neg	
1814	12/18/12	M	0.3	BUHUNGA	Neg	
1815	12/18/12	F	10.0	MALIBA	Neg	
1816	12/18/12	M	2.0	IZINGA	Pos	
1817	12/18/12	F	40.0	MALIBA	Neg	
1818	12/18/12	M	5.0	BUGOYE	Pos	
1819	12/18/12	M	20.0	MALIBA	Neg	
1820	12/18/12	F	5.0	RWAKING	Pos	
1821	12/18/12	F	39.0	MALIBA	Neg	
1822	12/18/12	F	39.0	BUGOYE	Neg	
1823	12/18/12	F	24.0	MALIBA	Neg	
1824	12/18/12	F	20.0	BUGOYE	Neg	
1825	12/18/12	F	20.0	BUNYANGONI	Neg	
1826	12/18/12	M	10.0	KANYAN	Neg	
1827	12/18/12	F	20.0	MAGHOMA	Neg	
1828	12/18/12	M	0.8	MALIBA	Neg	
1829	12/18/12	F	2.3	MALIBA	Neg	
1830	12/18/12	M	2.3	MALIBA	Neg	
1831	12/18/12	F	20.0	MALIBA	Pos	
1832	12/18/12	M	2.0	KYARUGOMOKA	Pos	
1833	12/20/12	M	1.6	KANYAN	Pos	
1834	12/20/12	M	35.0	MALIBA	Neg	
1835	12/20/12	F	2.3	MURAMBA	Neg	
1836	12/20/12	M	0.5	MURAMBA	Neg	
1837	12/20/12	F	20.0	KANYAN	Neg	
1838	12/20/12	M	14.0	MURAMBA	Neg	
1839	12/20/12	F	21.0	RWAKING		Pos
1840	12/20/12	F	0.0	KANYAN	Neg	
1841	12/20/12	F	36.0	KATOOKE	Neg	
1842	12/20/12	F	45.0	IZINGA	Neg	
1843	12/21/12	F	30.0	IZINGA	Neg	
1844	12/21/12	F	19.0	KANYAN	Neg	
1845	12/21/12	M	1.3	IBANDA	Neg	
1846	12/21/12	F	70.0	NDUGHUTU	Neg	
1847	12/21/12	F	10.0	IBANDA	Neg	
1848	12/21/12	F	50.0	RWAKING	Neg	

1849	12/21/12	F	3.0	IZINGA	Neg
1850	12/21/12	F	23.0	BUNYANGONI	Neg
1851	12/21/12	F	14.0	BUNYANGONI	Neg
1852	12/21/12	M	4.0	KANYAN	Pos
1853	12/21/12	F	10.0	RWAKING	Pos
1854	12/21/12	F	18.0	BUGOYE	Neg
1855	12/21/12	M	3.0	IZINGA	Neg
1856	12/21/12	F	60.0	KYANYA	Neg
1857	12/21/12	F	13.0	MALIBA	Neg
1858	12/21/12	M	0.9	NDUGHUTU	Neg
1859	12/21/12	F	9.0	IZINGA	Neg
1860	12/21/12	F	32.0	IZINGA	Neg
1861	12/21/12	M	2.0	KIBOTA	Neg
1862	12/21/12	M	0.7	KANYAN	Neg
1863	12/21/12	F	33.0	IZINGA	Neg
1864	1/2/13	F	32.0	BUGOYE	Neg
1865	1/2/13	F	28.0	BUGOYE	Neg
1866	1/2/13	F	30.0	BUGOYE	Neg
1867	1/2/13	F	5.0	BUGOYE	Pos
1868	1/2/13	M	6.0	KANYAN	Neg
1869	1/2/13	M	3.0	KANYAN	Pos
1870	1/2/13	F	39.0	BUGOYE	Neg
1871	1/2/13	M	50.0	BUNYANGONI	Neg
1872	1/2/13	F	20.0	MURAMBA	Neg
1873	1/2/13	M	22.0	KIRONGO	Neg
1874	1/2/13	M	28.0	NDUGHUTU	Neg
1875	1/2/13	F	0.6	IZINGA	Neg
1876	1/2/13	F	2.5	BUNYANGONI	Neg
1877	1/2/13	F	27.0	IBANDA	Neg
1878	1/2/13	M	10.0	BUGOYE	Pos
1879	1/2/13	M	11.0	BUGOYE	Pos
1880	1/2/13	M	2.0	NDUGHUTU	Neg
1881	1/2/13	M	16.0	NYAKARUNGU	Pos
1882	1/2/13	M	28.0	IBANDA	Pos
1883	1/2/13	F	50.0	MALIBA	Neg
1884	1/2/13	F	24.0	MALIBA	Pos
1885	1/2/13	F	2.0	BUGOYE	Neg
1886	1/2/13	F	0.7	MURAMBA	Neg
1887	1/2/13	F	29.0	KANYAN	Neg
1888	1/2/13	M	30.0	MALIBA	Neg
1889	1/2/13	F	7.0	MALIBA	Neg
1890	1/2/13	F	0.5	KIKOKERA	Neg
1891	1/2/13	F	10.0	MALIBA	Pos

1892	1/2/13	M	18.0	BUGOYE	Pos
1893	1/4/13	F	20.0	MURAMBA	Neg
1894	1/4/13	F	17.0	MURAMBA	Pos
1895	1/4/13	F	1.5	KAKINDO	Pos
1896	1/4/13	F	16.0	BUNYANGONI	Neg
1897	1/4/13	F	49.0	BUGOYE	Neg
1898	1/4/13	F	22.0	BUGOYE	Neg
1899	1/4/13	F	30.0	KANYAN	Neg
1900	1/4/13	F	5.0	KISANGA	Neg
1901	1/4/13	F	44.0	RWAKING	Neg
1902	1/4/13	F	27.0	BUGOYE	Neg
1903	1/4/13	F	0.3	BUGOYE	Neg
1904	1/4/13	M	58.0	KAKINDO	Neg
1905	1/4/13	M	24.0	BUGOYE	Neg
1906	1/4/13	M	19.0	KANYAN	Neg
1907	1/4/13	M	8.0	KANYAN	Neg
1908	1/4/13	M	20.0	NDUGHUTU	Neg
1909	1/4/13	F	12.0	IBANDA	Pos
1910	1/4/13	F	1.4	KAKINDO	Pos
1911	1/4/13	M	47.0	BUNYANGONI	Neg
1912	1/4/13	M	19.0	KANYAN	Pos
1913	1/4/13	M	38.0	KANYAN	Neg
1914	1/4/13	F	52.0	KANYAN	Neg
1915	1/4/13	F	22.0	BUGOYE	Neg
1916	1/4/13	F	40.0	MURAMBA	Neg
1917	1/4/13	F	36.0	BUGOYE	Neg
1918	1/4/13	M	3.4	KYARUGOMOKA	Neg
1919	1/4/13	M	31.0	KYARUGOMOKA	Neg
1920	1/4/13	M	18.0	MURAMBA	Neg
1921	1/4/13	F	21.0	IBANDA	Neg
1922	1/4/13	M	25.0	MALIBA	Neg
1923	1/4/13	F	21.0	KISANGA	Neg
1924	1/4/13	F	14.0	MURAMBA	Neg
1925	1/7/13	F	65.0	RWAKING	Neg
1926	1/7/13	F	40.0	KISAMBA	Neg
1927	1/7/13	M	2.0	MALIBA	Neg
1928	1/7/13	F	27.0	KIRULI	Neg
1929	1/7/13	F	52.0	MUHAMBO	Neg
1930	1/7/13	F	25.0	BUGOYE	Neg
1931	1/7/13	M	0.4	BUGOYE	Neg
1932	1/7/13	F	0.3	BUGOYE	Pos
1933	1/7/13	M	0.8	KIHINDI	Neg
1934	1/7/13	M	15.0	KIRONGO	Neg

1935	1/7/13	F	55.0	MUHAMBO	Neg
1936	1/7/13	F	14.0	MALIBA	Pos
1937	1/7/13	M	6.0	KANYAN	Pos
1938	1/7/13	F	8.0	NYAKARUNGU	Neg
1939	1/7/13	F	9.0	RWAKING	Neg
1940	1/7/13	F	28.0	BUGOYE	Neg
1941	1/7/13	M	4.0	KATUMBA	Neg
1942	1/8/13	M	1.1	KATUMBA	Neg
1943	1/8/13	M	30.0	MURAMBA	Neg
1944	1/8/13	F	26.0	IZINGA	Neg
1945	1/8/13	M	20.0	BUGOYE	Pos
1946	1/8/13	M	12.0	NDUGHUTU	Neg
1947	1/8/13	M	6.0	KANYAN	Pos
1948	1/8/13	F	53.0	BUGOYE	Neg
1949	1/8/13	F	27.0	MALIBA	Neg
1950	1/8/13	M	2.2	NDUGHUTU	Neg
1951	1/8/13	M	5.0	IBANDA	Neg
1952	1/8/13	M	25.0	MALIBA	Neg
1953	1/8/13	F	32.0	BUGOYE	Neg
1954	1/8/13	M	3.0	KIRULI	Neg
1955	1/8/13	M	23.0	MALIBA	Neg
1956	1/8/13	M	7.0	KATUMBA	Neg
1957	1/8/13	F	19.0	RWAKING	Neg
1958	1/8/13	F	1.2	MURAMBA	Neg
1959	1/8/13	F	33.0	IHANI	Neg
1960	1/9/13	M	1.5	KANYAN	Neg
1961	1/9/13	F	81.0	RWAKING	Neg
1962	1/9/13	F	46.0	BUGOYE	Neg
1963	1/9/13	M	14.0	BUGOYE	Neg
1964	1/9/13	M	1.0	NDUGHUTU	Neg
1965	1/9/13	F	35.0	BUGOYE	Neg
1966	1/9/13	M	0.8	NDUGHUTU	Neg
1967	1/9/13	M	34.0	MURAMBA	Neg
1968	1/9/13	F	24.0	KISAMBA	Neg
1969	1/9/13	M	58.0	MALIBA	Neg
1970	1/9/13	F	40.0	KANYAN	Neg
1971	1/9/13	F	21.0	MURAMBA	Neg
1972	1/9/13	M	39.0	IZINGA	Neg
1973	1/9/13	M	28.0	MALIBA	Neg
1974	1/9/13	F	20.0	MURAMBA	Neg
1975	1/9/13	M	17.0	BUGOYE	Neg
1976	1/9/13	F	30.0	KAKINDO	Neg
1977	1/9/13	F	18.0	KYARUGOMOKA	Neg

1978	1/9/13	F	10.0	IHANI	Neg
1979	1/10/13	F	0.5	KANYAN	Pos
1980	1/10/13	F	52.0	KANYAN	Neg
1981	1/10/13	F	33.0	KANYAN	Pos
1982	1/10/13	F	0.3	KANYAN	Pos
1983	1/10/13	F	8.0	IHANI	Neg
1984	1/10/13	M	3.0	KANYAN	Pos
1985	1/10/13	F	0.2	KIKOKERA	Neg
1986	1/10/13	M	22.0	NDUGHUTU	Pos
1987	1/10/13	F	0.8	KIRULI	Neg
1988	1/10/13	F	6.0	KANYAN	Pos
1989	1/10/13	F	1.5	KANYAN	Neg
1990	1/10/13	M	19.0	MALIBA	Neg
1991	1/10/13	M	17.0	MALIBA	Pos
1992	1/10/13	F	19.0	MURAMBA	Pos
1993	1/10/13	F	24.0	BOMORO	Neg
1994	1/10/13	M	0.5	MUBUKU	Neg
1995	1/10/13	F	19.0	MUBUKU	Pos
1996	1/10/13	M	4.5	NDUGHUTU	Neg
1997	1/10/13	F	2.5	MURAMBA	Neg
1998	1/10/13	F	20.0	MURAMBA	Neg
1999	1/10/13	F	25.0	NYANGONGE	Neg
2000	1/10/13	M	18.0	IBANDA	Neg
2001	1/11/13	F	20.0	BUGOYE	Neg
2002	1/11/13	M	40.0	KANYAN	Pos
2003	1/11/13	F	17.0	MALIBA	Neg
2004	1/11/13	M	40.0	KIRONGO	Neg
2005	1/11/13	F	1.8	BUGOYE	Neg
2006	1/11/13	M	8.0	IZINGA	Pos
2007	1/11/13	M	1.5	MURAMBA	Neg
2008	1/11/13	M	35.0	NDUGHUTU	Neg
2009	1/11/13	F	17.0	IHANI	Pos
2010	1/11/13	F	6.0	BUGOYE	Neg
2011	1/11/13	F	30.0	NYAKARUNGU	Pos
2012	1/11/13	F	23.0	KANYAN	Neg
2013	1/11/13	F	48.0	BUGOYE	Neg
2014	1/14/13	M	0.4	KANYAN	Neg
2015	1/14/13	F	1.5	RWAKING	Neg
2016	1/14/13	M	1.4	KANYAN	Neg
2017	1/14/13	F	20.0	KANYAN	Neg
2018	1/14/13	M	53.0	KANYAN	Neg
2019	1/14/13	F	12.0	MALIBA	Neg
2020	1/14/13	F	41.0	NDUGHUTU	Neg

2021	1/14/13	F	30.0	KANYAN	Neg
2022	1/14/13	F	54.0	IHANI	Neg
2023	1/14/13	F	20.0	BUGOYE	Neg
2024	1/14/13	F	38.0	BUGOYE	Neg
2025	1/14/13	M	4.0	KATUMBA	Neg
2026	1/14/13	F	4.0	IHANI	Neg
2027	1/14/13	F	23.0	IHANI	Neg
2028	1/14/13	F	23.0	KYANYA	Pos
2029	1/14/13	F	0.5	IZINGA	Neg
2030	1/14/13	M	1.0	MALIBA	Neg
2031	1/14/13	F	20.0	MALIBA	Neg
2032	1/14/13	F	6.0	KIRONGO	Neg
2033	1/14/13	M	3.5	RWAKING	Neg
2034	1/14/13	F	1.5	.	Pos
2035	1/14/13	F	65.0	MALIBA	Neg
2036	1/14/13	M	35.0	MALIBA	Neg
2037	1/14/13	F	46.0	NYAKARUNGU	Neg
2038	1/15/13	M	14.0	MURAMBA	Pos
2039	1/15/13	M	30.0	KIKOKERA	Neg
2040	1/15/13	F	13.0	MURAMBA	Neg
2041	1/15/13	F	1.3	KIRULI	Neg
2042	1/15/13	F	29.0	KYANYA	Neg
2043	1/15/13	F	1.4	RWAKING	Neg
2044	1/15/13	F	4.0	KANYAN	Pos
2045	1/15/13	M	3.0	KANYAN	Pos
2046	1/15/13	F	24.0	KIKOKERA	Neg
2047	1/15/13	M	3.0	KIKOKERA	Neg
2048	1/15/13	M	5.0	MURAMBA	Neg
2049	1/15/13	M	8.0	KANYAN	Pos
2050	1/15/13	F	5.0	KANYAN	Neg
2051	1/15/13	F	26.0	KIKOKERA	Neg
2052	1/15/13	M	2.5	KANYAN	Neg
2053	1/15/13	M	13.0	KANYAN	Pos
2054	1/16/13	M	0.6	KANYAN	Neg
2055	1/16/13	F	20.0	KANYAN	Neg
2056	1/16/13	M	60.0	MALIBA	Neg
2057	1/16/13	M	14.0	BUGOYE	Pos
2058	1/16/13	F	29.0	NDUGHUTU	Neg
2059	1/16/13	F	10.0	BUNYANGONI	Neg
2060	1/16/13	F	17.0	BUGOYE	Neg
2061	1/16/13	F	0.8	MALIBA	Pos
2062	1/16/13	F	44.0	NDUGHUTU	Neg
2063	1/16/13	F	50.0	BUHUNGA	Neg

2064	1/16/13	M	26.0	KIRONGO	Neg
2065	1/16/13	F	30.0	.	Neg
2066	1/16/13	F	1.1	MALIBA	Neg
2067	1/16/13	M	8.0	MALIBA	Neg
2068	1/16/13	F	4.0	BUGOYE	Neg
2069	1/16/13	F	5.0	NDUGHUTU	Pos
2070	1/16/13	F	0.2	BUNYANGONI	Neg
2071	1/16/13	F	35.0	MUKATI	Neg
2072	1/17/13	F	1.1	KIKOKERA	Neg
2073	1/17/13	M	27.0	BUGOYE	Neg
2074	1/17/13	F	13.0	MURAMBA	Neg
2075	1/17/13	F	53.0	KATOOKE	Neg
2076	1/17/13	M	1.0	BUNYANGONI	Neg
2077	1/17/13	F	10.0	MURAMBA	Neg
2078	1/17/13	M	5.0	MALIBA	Neg
2079	1/17/13	F	18.0	KIKOKERA	Neg
2080	1/17/13	F	52.0	KIRONGO	Neg
2081	1/17/13	M	1.1	NDUGHUTU	Neg
2082	1/17/13	M	40.0	KANYAN	Pos
2083	1/17/13	M	6.0	KISANZI	Pos
2084	1/17/13	F	50.0	KISANZI	Neg
2085	1/17/13	F	27.0	KIKOKERA	Neg
2086	1/17/13	M	52.0	MALIBA	Neg
2087	1/17/13	M	4.0	KANYAN	Neg
2088	1/18/13	F	22.0	IZINGA	Neg
2089	1/18/13	F	34.0	KANYAN	Neg
2090	1/18/13	F	44.0	RWAKING	Neg
2091	1/18/13	F	12.0	BUGOYE	Pos
2092	1/18/13	F	0.0	RWAKING	Neg
2093	1/18/13	F	17.0	BUGOYE	Pos
2094	1/18/13	F	19.0	ISULE	Neg
2095	1/18/13	F	0.8	MURAMBA	Neg
2096	1/18/13	F	0.8	NYAKARUNGU	Neg
2097	1/18/13	M	0.1	KANYAN	Neg
2098	1/18/13	F	23.0	KAKINDO	Neg
2099	1/18/13	M	95.0	NDUGHUTU	Neg
2100	1/18/13	M	19.0	MALIBA	Pos
2101	1/18/13	F	50.0	BUGOYE	Neg
2102	1/18/13	F	22.0	RWAKING	Neg
2103	1/18/13	F	18.0	IZINGA	Neg
2104	1/18/13	M	0.4	MURAMBA	Pos
2105	1/18/13	F	21.0	BUGOYE	Neg
2106	1/18/13	M	16.0	RWAKING	Neg

2107	1/21/13	M	27.0	MURAMBA	Neg
2108	1/21/13	F	9.0	KANYAN	Pos
2109	1/21/13	F	19.0	KANYAN	Neg
2110	1/21/13	M	17.0	BUNYANGONI	Neg
2111	1/21/13	F	23.0	NYAKARUNGU	Pos
2112	1/21/13	F	2.2	KANYAN	Neg
2113	1/21/13	F	0.2	BUGOYE	Neg
2114	1/21/13	M	14.0	BUGOYE	Neg
2115	1/21/13	F	24.0	NDUGHUTU	Neg
2116	1/21/13	F	17.0	NDUGHUTU	Neg
2117	1/21/13	F	20.0	MALIBA	Neg
2118	1/21/13	F	20.0	KIRULI	Pos
2119	1/21/13	M	33.0	RWAKING	Neg
2120	1/21/13	F	4.0	KANYAN	Pos
2121	1/21/13	F	18.0	KANYAN	Neg
2122	1/21/13	M	9.0	NDUGHUTU	Neg
2123	1/21/13	M	6.0	RUKOKI	Neg
2124	1/21/13	F	9.0	KANYAN	Pos
2125	1/21/13	F	30.0	KINGOYE	Neg
2126	1/21/13	M	2.5	KANYAN	Neg
2127	1/21/13	F	19.0	MURAMBA	Pos
2128	1/21/13	F	3.8	IBANDA	Neg
2129	1/21/13	M	6.0	IBANDA	Neg
2130	1/21/13	F	2.0	MAGHOMA	Neg
2131	1/21/13	F	60.0	BUGOYE	Neg
2132	1/21/13	F	33.0	KANYAN	Pos
2133	1/21/13	F	38.0	BUGOYE	Neg
2134	1/21/13	F	2.5	BUGOYE	Neg
2135	1/21/13	F	1.5	BUGOYE	Neg
2136	1/21/13	F	0.3	BUNYANGONI	Neg
2137	1/21/13	F	35.0	MALIBA	Neg
2138	1/21/13	M	27.0	RWAKING	Neg
2139	1/21/13	F	37.0	ISULE	Neg
2140	1/21/13	F	40.0	MALIBA	Neg
2141	1/21/13	M	65.0	.	Neg
2142	1/21/13	M	37.0	KAKINDO	Neg
2143	1/21/13	M	2.0	MURAMBA	Neg
2144	1/21/13	M	8.0	KAKINDO	Neg
2145	1/21/13	M	5.0	MALIBA	Neg
2146	1/21/13	F	1.0	KANYAN	Neg
2147	1/21/13	F	15.0	KANYAN	Pos
2148	1/21/13	F	25.0	KAKINDO	Neg
2149	1/21/13	F	12.0	RWAKING	Neg

2150	1/22/13	M	74.0	BUGOYE	Neg
2151	1/22/13	F	2.0	KANYAN	Pos
2152	1/22/13	F	12.0	IZINGA	Pos
2153	1/22/13	M	38.0	MALIBA	Neg
2154	1/22/13	F	36.0	IZINGA	Neg
2155	1/22/13	M	1.5	NDUGHUTU	Neg
2156	1/22/13	F	3.0	BULYANDEKE	Neg
2157	1/22/13	M	1.6	KANYAN	Neg
2158	1/22/13	F	45.0	BUNYANGONI	Neg
2159	1/22/13	F	18.0	BUGOYE	Neg
2160	1/22/13	F	14.0	KISAMBA	Pos
2161	1/22/13	M	1.0	NDUGHUTU	Pos
2162	1/22/13	F	5.0	NDUGHUTU	Pos
2163	1/22/13	F	39.0	MURAMBA	Neg
2164	1/22/13	F	62.0	NDUGHUTU	Pos
2165	1/22/13	M	12.0	MALIBA	Pos
2166	1/22/13	M	3.5	IZINGA	Pos
2167	1/22/13	F	17.0	MALIBA	Neg
2168	1/22/13	M	65.0	IZINGA	Neg
2169	1/22/13	M	1.3	NDUGHUTU	Neg
2170	1/22/13	M	4.0	KATOOKE	Pos
2171	1/22/13	M	0.5	MURAMBA	Pos
2172	1/23/13	M	1.0	KIHINDI	Neg
2173	1/23/13	F	3.0	IZINGA	Neg
2174	1/23/13	F	3.0	NDUGHUTU	Neg
2175	1/23/13	F	10.0	BUNYANGONI	Pos
2176	1/23/13	F	8.0	NDUGHUTU	Pos
2177	1/23/13	M	50.0	MALIBA	Neg
2178	1/23/13	M	63.0	IZINGA	Neg
2179	1/23/13	F	50.0	MALIBA	Neg
2180	1/23/13	F	0.7	KIRONGO	Neg
2181	1/23/13	F	55.0	KANYAN	Pos
2182	1/23/13	M	1.5	NDUGHUTU	Neg
2183	1/23/13	F	1.7	KANYAN	Neg
2184	1/23/13	M	22.0	RWAKING	Neg
2185	1/23/13	M	13.0	RWAKING	Neg
2186	1/23/13	F	7.0	KISAMBA	Pos
2187	1/23/13	F	54.0	BUNYANGONI	Neg
2188	1/23/13	F	28.0	MALIBA	Neg
2189	1/23/13	F	21.0	BUGOYE	Neg
2190	1/23/13	F	29.0	RWAKING	Neg
2191	1/23/13	M	5.0	RWAKING	Pos
2192	1/23/13	F	1.5	.	Neg

2193	1/23/13	M	3.0	RWAKING	Pos
2194	1/23/13	F	0.8	IHANI	Neg
2195	1/24/13	F	33.0	MALIBA	Neg
2196	1/24/13	M	17.0	BUGOYE	Pos
2197	1/24/13	F	4.0	IZINGA	Pos
2198	1/24/13	F	1.8	IZINGA	Neg
2199	1/24/13	M	60.0	NYAKARUNGU	Neg
2200	1/24/13	F	2.5	BUGOYE	Neg
2201	1/24/13	F	15.0	NYABISUSI	Neg
2202	1/24/13	F	2.0	IZINGA	Neg
2203	1/24/13	F	36.0	KABUGHO	Neg
2204	1/24/13	F	20.0	MURAMBA	Pos
2205	1/24/13	M	9.0	KYANYA	Neg
2206	1/24/13	F	27.0	MALIBA	Neg
2207	1/24/13	F	31.0	BUGOYE	Neg
2208	1/24/13	F	14.0	NDUGHUTU	Neg
2209	1/24/13	M	10.0	BUGOYE	Neg
2210	1/24/13	F	3.5	KANYAN	Neg
2211	1/24/13	M	53.0	KANYAN	Neg
2212	1/24/13	F	70.0	KYARUGOMOKA	Neg
2213	1/24/13	M	0.8	BUNYANGONI	Neg
2214	1/24/13	M	1.7	BUNYANGONI	Neg
2215	1/24/13	F	70.0	IBANDA	Neg
2216	1/24/13	F	60.0	IBANDA	Neg
2217	1/24/13	F	12.0	KISANZI	Pos
2218	1/24/13	M	30.0	NDUGHUTU	Pos
2219	1/24/13	F	3.0	RWAKING	Neg
2220	1/24/13	F	60.0	BUNYANGONI	Neg
2221	1/24/13	F	0.3	MURAMBA	Neg
2222	1/24/13	M	0.8	KIKOKERA	Neg
2223	1/24/13	M	18.0	IHANI	Neg
2224	1/25/13	M	10.0	KIBOTA	Pos
2225	1/25/13	F	16.0	KANYAN	Pos
2226	1/25/13	F	25.0	MALIBA	Neg
2227	1/25/13	F	0.4	KIHINDI	Neg
2228	1/25/13	M	24.0	KATUMBA	Neg
2229	1/25/13	F	15.0	KANYAN	Pos
2230	1/25/13	M	3.5	KANYAN	Pos
2231	1/25/13	M	8.0	IZINGA	Pos
2232	1/25/13	F	13.0	NDUGHUTU	Pos
2233	1/25/13	M	0.3	KIRONGO	Neg
2234	1/25/13	F	52.0	KIRONGO	Neg
2235	1/25/13	M	6.0	KANYAN	Pos

2236	1/25/13	F	22.0	IBANDA	Pos
2237	1/28/13	M	4.0	IBANDA	Neg
2238	1/28/13	F	41.0	KYARUGOMOKA	Neg
2239	1/28/13	F	38.0	NYAMBUKU	Neg
2240	1/28/13	F	24.0	MAGHOMA	Neg
2241	1/28/13	M	5.0	MAGHOMA	Neg
2242	1/28/13	F	12.0	RWAKING	Neg
2243	1/31/13	F	21.0	KIRULI	Neg
2244	1/31/13	M	21.0	KANYAN	Neg
2245	1/31/13	M	1.5	KANYAN	Pos
2246	1/31/13	F	13.0	NDUGHUTU	Pos
2247	1/31/13	M	45.0	BUGOYE	Neg
2248	1/31/13	F	5.8	KIKOKERA	Neg
2249	1/31/13	M	0.3	MURAMBA	Neg
2250	1/31/13	M	24.0	KANYAN	Neg
2251	1/31/13	F	46.0	KIKOKERA	Neg
2252	1/31/13	M	1.0	KATUMBA	Neg
2253	1/31/13	F	32.0	RWAKING	Pos
2254	1/31/13	M	1.8	NDUGHUTU	Neg
2255	1/31/13	M	14.0	KANYAN	Pos
2256	1/31/13	M	0.5	BUGOYE	Neg
2257	1/31/13	F	16.0	NDUGHUTU	Neg
2258	1/31/13	M	9.0	NDUGHUTU	Neg
2259	1/31/13	F	29.0	KATOOKE	Neg
2260	1/31/13	F	19.0	KIKOKERA	Pos
2261	1/31/13	F	1.8	KISANZI	Neg
2262	1/31/13	F	7.0	NDUGHUTU	Pos
2263	2/1/13	M	10.0	BUGOYE	Pos
2264	2/1/13	M	2.6	RWAKING	Pos
2265	2/1/13	F	17.0	IHANI	Pos
2266	2/1/13	M	5.0	MURAMBA	Pos
2267	2/1/13	F	23.0	MAGHOMA	Neg
2268	2/1/13	F	7.0	KYARUGOMOKA	Pos
2269	2/1/13	F	2.1	KIBIRIZI	Pos
2270	2/1/13	M	0.6	NDUGHUTU	Neg
2271	2/1/13	M	1.6	KIRONGO	Neg
2272	2/1/13	M	2.0	KIRONGO	Neg
2273	2/1/13	F	29.0	MURAMBA	Neg
2274	2/4/13	M	2.0	MALIBA	Neg
2275	2/4/13	M	31.0	NDUGHUTU	Neg
2276	2/4/13	F	0.7	MURAMBA	Neg
2277	2/4/13	M	15.0	MALIBA	Neg
2278	2/4/13	M	5.0	KYANYA	Neg

2279	2/4/13	M	17.0	BUGOYE	Pos
2280	2/4/13	F	21.0	KANYAN	Pos
2281	2/4/13	F	1.8	KANYAN	Pos
2282	2/4/13	F	1.6	KANYAN	Pos
2283	2/4/13	M	4.5	KANYAN	Neg
2284	2/4/13	F	17.0	KATUMBA	Neg
2285	2/4/13	F	48.0	KIBOTA	Neg
2286	2/4/13	F	4.0	NDUGHUTU	Neg
2287	2/4/13	F	0.7	NDUGHUTU	Neg
2288	2/4/13	M	0.1	KIRONGO	Neg
2289	2/4/13	F	25.0	KANYAN	Neg
2290	2/4/13	F	0.1	KANYAN	Neg
2291	2/4/13	F	13.0	RWAKING	Neg
2292	2/4/13	F	29.0	NDUGHUTU	Pos
2293	2/4/13	M	1.5	NDUGHUTU	Neg
2294	2/4/13	M	15.0	IBANDA	Neg
2295	2/4/13	F	8.0	MURAMBA	Neg
2296	2/4/13	F	50.0	KATOOKE	Pos
2297	2/4/13	F	55.0	KAKINDO	Pos
2298	2/4/13	F	16.0	NDUGHUTU	Neg
2299	2/5/13	M	16.0	RWAKING	Pos
2300	2/5/13	M	3.0	KIKOKERA	Pos
2301	2/5/13	F	6.0	KANYAN	Pos
2302	2/5/13	M	0.8	NYAKARUNGU	Neg
2303	2/5/13	F	0.6	KIRULI	Pos
2304	2/5/13	F	2.0	KIRULI	Neg
2305	2/5/13	F	1.3	NDUGHUTU	Neg
2306	2/5/13	M	0.5	NDUGHUTU	Neg
2307	2/5/13	F	13.0	KIKOKERA	Neg
2308	2/5/13	F	70.0	KATOOKE	Neg
2309	2/5/13	M	14.0	KANYAN	Pos
2310	2/6/13	F	0.6	MALIBA	Neg
2311	2/6/13	M	10.0	NDUGHUTU	Neg
2312	2/6/13	M	12.0	KANYAN	Neg
2313	2/6/13	F	40.0	NDUGHUTU	Neg
2314	2/6/13	M	1.2	MURAMBA	Neg
2315	2/6/13	M	12.0	KANYAN	Pos
2316	2/6/13	F	1.5	NDUGHUTU	Neg
2317	2/6/13	F	13.0	BUGOYE	Pos
2318	2/6/13	F	46.0	MALIBA	Pos
2319	2/6/13	M	12.0	BUNYANGONI	Neg
2320	2/6/13	F	39.0	KATOOKE	Pos
2321	2/6/13	F	21.0	MURAMBA	Neg

2322	2/6/13	F	27.0	IHANI	Neg
2323	2/6/13	F	42.0	MURAMBA	Neg
2324	2/6/13	F	36.0	KABUGHO	Pos
2325	2/6/13	M	12.0	IZINGA	Neg
2326	2/6/13	M	3.0	KATOOKE	Neg
2327	2/6/13	F	24.0	IZINGA	Neg
2328	2/6/13	M	1.5	IZINGA	Neg
2329	2/6/13	M	48.0	KABUGHO	Pos
2330	2/6/13	F	0.3	NYAKARUNGU	Neg
2331	2/6/13	F	7.0	NYAKARUNGU	Neg
2332	2/8/13	M	1.5	MAGHOMA	Neg
2333	2/8/13	F	35.0	IZINGA	Pos
2334	2/8/13	F	8.0	IZINGA	Neg
2335	2/8/13	F	50.0	MURAMBA	Neg
2336	2/8/13	F	80.0	MURAMBA	Neg
2337	2/8/13	F	15.0	BUGOYE	Neg
2338	2/8/13	M	6.0	KANYAN	Pos
2339	2/8/13	M	1.0	KATUMBA	Neg
2340	2/8/13	F	47.0	RWAKING	Pos
2341	2/8/13	F	40.0	NDUGHUTU	Neg
2342	2/8/13	F	3.5	KANYAN	Neg
2343	2/8/13	M	3.0	NYAKARUNGU	Neg
2344	2/8/13	M	2.0	NYAKARUNGU	Neg
2345	2/8/13	M	1.5	KYARUGOMOKA	Neg
2346	2/8/13	F	30.0	MWAKATHI	Neg
2347	2/8/13	F	18.0	MURAMBA	Neg
2348	2/12/13	M	74.0	BUGOYE	Neg
2349	2/12/13	F	15.0	NDUGHUTU	Pos
2350	2/12/13	M	80.0	MURAMBA	Neg
2351	2/12/13	F	24.0	RWAKING	Pos
2352	2/12/13	F	8.0	RWAKING	Pos
2353	2/12/13	M	14.0	NDUGHUTU	Pos
2354	2/12/13	F	53.0	KANYAN	Pos
2355	2/12/13	F	12.0	KAKINDO	Pos
2356	2/12/13	F	2.9	RWAKING	Neg
2357	2/12/13	F	0.6	MURAMBA	Neg
2358	2/12/13	M	2.0	NDUGHUTU	Neg
2359	2/12/13	M	2.8	KANYAN	Pos
2360	2/12/13	F	21.0	IZINGA	Pos
2361	2/12/13	F	7.0	RWAKING	Neg
2362	2/12/13	F	20.0	NDUGHUTU	Neg
2363	2/12/13	M	7.0	MURAMBA	Pos
2364	2/12/13	M	0.9	KIKOKERA	Neg

2365	2/12/13	M	2.0	KANYAN	Pos
2366	2/12/13	M	4.5	KANYAN	Pos
2367	2/12/13	M	3.5	KIKOKERA	Neg
2368	2/12/13	M	3.0	BULINDUGURU	Neg
2369	2/12/13	F	19.0	KANYAN	Pos
2370	2/12/13	F	75.0	MURAMBA	Neg
2371	2/12/13	F	18.0	KATOOKE	Pos
2372	2/12/13	M	0.4	MAGHOMA	Neg
2373	2/12/13	F	25.0	BUGOYE	Neg
2374	2/12/13	F	24.0	KATUMBA	Neg
2375	2/12/13	M	0.6	KISANGA	Neg
2376	2/12/13	F	16.0	NYAKALENGIJO	Pos
2377	2/12/13	M	20.0	BUGOYE	Neg
2378	2/12/13	F	4.5	KIHINDI	Neg
2379	2/12/13	F	40.0	MUHAMBO	Neg
2380	2/12/13	F	13.0	RWAKING	Neg
2381	2/12/13	F	14.0	KANYAN	Pos
2382	2/12/13	F	15.0	KANYAN	Pos
2383	2/12/13	M	2.0	KANYAN	Pos
2384	2/12/13	F	18.0	BUGOYE	Pos
2385	2/12/13	M	0.4	KANYAN	Neg
2386	2/12/13	F	18.0	BUGOYE	Neg
2387	2/12/13	F	13.0	IZINGA	Pos
2388	2/12/13	M	20.0	IZINGA	Pos
2389	2/12/13	M	3.0	IZINGA	Neg
2390	2/12/13	M	1.3	NDUGHUTU	Neg
2391	2/12/13	F	15.0	MURAMBA	Pos
2392	2/12/13	F	23.0	BUGOYE	Neg
2393	2/12/13	F	57.0	KANYAN	Neg
2394	2/12/13	F	25.0	BUGOYE	Pos
2395	2/12/13	F	6.0	KANYAN	Pos
2396	2/13/13	F	14.0	KIRONGO	Pos
2397	2/13/13	F	10.0	IZINGA	Pos
2398	2/13/13	F	28.0	BUGOYE	Neg
2399	2/13/13	F	0.3	KANYAN	Pos
2400	2/13/13	F	1.4	KANYAN	Neg
2401	2/13/13	F	12.0	MUHAMBO	Pos
2402	2/13/13	F	10.0	NDUGHUTU	Pos
2403	2/13/13	M	10.0	BUHUNGA	Neg
2404	2/13/13	M	10.0	NDUGHUTU	Pos
2405	2/13/13	F	6.0	KANYAN	Pos
2406	2/13/13	F	14.0	KANYAN	Pos
2407	2/13/13	F	20.0	IBANDA	Neg

2408	2/13/13	F	29.0	MAGHOMA	Neg	
2409	2/13/13	F	18.0	BUGOYE	Neg	
2410	2/13/13	M	60.0	IZINGA	Neg	
2411	2/13/13	F	13.0	BUGOYE	Neg	
2412	2/13/13	M	3.0	KIRONGO	Neg	
2413	2/13/13	F	32.0	MALIBA	Neg	
2414	2/13/13	M	77.0	KISANZI	Pos	
2415	2/13/13	M	2.5	KIKOKERA	Pos	
2416	2/13/13	F	5.0	KIRONGO	Neg	
2417	2/13/13	F	10.0	KANYAN	Pos	
2418	2/13/13	M	12.0	MUBUKU	Pos	
2419	2/14/13	F	59.0	IZINGA	Neg	
2420	2/14/13	F	19.0	MURAMBA	Neg	
2421	2/14/13	F	16.0	MURAMBA	Pos	
2422	2/14/13	F	25.0	MURAMBA	Neg	
2423	2/14/13	M	0.7	MURAMBA	Neg	
2424	2/14/13	M	87.0	BUZIBWERA	Neg	
2425	2/14/13	M	10.0	MURAMBA	Neg	
2426	2/14/13	F	7.0	IZINGA	Pos	
2427	2/14/13	M	30.0	IZINGA	Neg	
2428	2/14/13	M	6.0	KIKOKERA	Neg	
2429	2/14/13	M	6.0	KIKOKERA	Neg	
2430	2/14/13	M	18.0	NDUGHUTU	Pos	
2431	2/14/13	F	35.0	KINYANGONGE	Neg	
2432	2/14/13	F	40.0	MURAMBA	Neg	
2433	2/14/13	F	20.0	KINYANGONGE	Pos	
2434	2/14/13	M	55.0	BUNYANGONI	Neg	
2435	2/15/13	M	2.0	KISANZI	Neg	
2436	2/15/13	F	4.5	BUGOYE	Pos	
2437	2/15/13	M	12.0	MURAMBA	Neg	
2438	2/15/13	F	5.0	RWAKING	Pos	
2439	2/15/13	F	12.0	BUNYANGONI	Neg	
2440	2/15/13	M	3.0	BUGOYE	Pos	
2441	2/15/13	F	34.0	NYAKARUNGU	Neg	
2442	2/15/13	M	3.0	BUGOYE	Neg	
2443	2/15/13	F	19.0	BUNYANGONI	Neg	
2444	2/15/13	F	49.0	IZINGA	Neg	
2445	2/15/13	F	12.0	NYABISUSI	Pos	
2446	2/15/13	F	30.0	NYABISUSI	Neg	
2447	2/15/13	F	2.3	NYABISUSI	Neg	
2448	2/15/13	F	8.5	NYABISUSI	Pos	
2449	2/15/13	F	38.0	BIKONE	Neg	
2450	2/15/13	M	0.7	MURAMBA		Neg

2451	2/15/13	F	6.0	MURAMBA	Pos
2452	2/15/13	M	20.0	NYAKALENGIJO	Neg
2453	2/15/13	F	0.6	KANYAN	Pos
2454	2/15/13	F	20.0	MURAMBA	Neg
2455	2/15/13	F	55.0	KANYAN	Neg
2456	2/15/13	F	25.0	MALIBA	Neg
2457	2/15/13	F	2.4	MALIBA	Neg
2458	2/15/13	F	19.0	MURAMBA	Neg
2459	2/15/13	F	0.1	RWAKING	Neg
2460	2/15/13	F	0.9	BUGOYE	Neg
2461	2/15/13	F	17.0	MALIBA	Neg
2462	2/15/13	F	25.0	BUNYANGONI	Neg
2463	2/15/13	M	1.5	MURAMBA	Neg
2464	2/15/13	F	23.0	BUGOYE	Pos
2465	2/15/13	F	17.0	KIKOKERA	Neg
2466	2/15/13	F	4.0	BUNYANGONI	Pos
2467	2/15/13	F	28.0	NDUGHUTU	Pos
2468	2/15/13	F	8.0	BUNYANGONI	Neg
2469	2/15/13	F	1.2	MAGHOMA	Pos
2470	2/15/13	F	15.0	BUGOYE	Neg
2471	2/15/13	M	0.3	RUENDABA	Neg
2472	2/15/13	M	16.0	IZINGA	Neg
2473	2/15/13	F	25.0	RUENDABA	Neg
2474	2/15/13	F	8.0	KANYAN	Pos
2475	2/15/13	F	6.5	KATUMBA	Neg
2476	2/15/13	M	12.0	KANYAN	Pos
2477	2/15/13	F	12.0	MALIBA	Neg
2478	2/19/13	F	2.0	BUGOYE	Neg
2479	2/19/13	F	10.0	BUNYANGONI	Pos
2480	2/19/13	F	19.0	BUNYANGONI	Neg
2481	2/19/13	F	21.0	KIKOKERA	Neg
2482	2/19/13	M	40.0	KANYAN	Neg
2483	2/19/13	F	0.4	KANYAN	Pos
2484	2/19/13	F	38.0	IBANDA	Neg
2485	2/19/13	M	12.0	IHANI	Pos
2486	2/19/13	F	33.0	KIRONGO	Pos
2487	2/19/13	M	0.9	MALIBA	Pos
2488	2/19/13	F	20.0	BUGOYE	Pos
2489	2/19/13	F	35.0	KATOOKE	Pos
2490	2/19/13	F	8.0	KANYAN	Pos
2491	2/19/13	M	3.0	KANYAN	Pos
2492	2/19/13	F	4.5	MURAMBA	Neg
2493	2/19/13	M	1.5	KIKOKERA	Neg

2494	2/19/13	F	10.0	KANYAN	Pos
2495	2/19/13	F	0.4	MURAMBA	Neg
2496	2/19/13	F	1.8	KIBIRIZI	Neg
2497	2/19/13	M	75.0	KIRONGO	Neg
2498	2/19/13	F	12.0	RWAKING	Pos
2499	2/19/13	F	20.0	RWAKING	Neg
2500	2/19/13	F	15.0	NDUGHUTU	Neg
2501	2/20/13	F	9.0	BUGOYE	Pos
2502	2/20/13	F	3.0	NYAKARUNGU	Pos
2503	2/20/13	M	6.0	MURAMBA	Neg
2504	2/20/13	M	6.0	KISANGA	Neg
2505	2/20/13	M	5.0	BUZIBWERA	Neg
2506	2/20/13	F	20.0	RWAKING	Neg
2507	2/20/13	F	17.0	KISANGA	Neg
2508	2/20/13	M	33.0	BUGOYE	Neg
2509	2/20/13	F	25.0	KIRONGO	Pos
2510	2/20/13	F	1.5	BUGOYE	Neg
2511	2/20/13	F	45.0	BUGOYE	Neg
2512	2/20/13	M	23.0	KANYAN	Neg
2513	2/20/13	F	2.4	KANYAN	Neg
2514	2/21/13	F	0.1	RWAKING	Neg
2515	2/21/13	M	1.0	MURAMBA	Neg
2516	2/21/13	F	4.0	KANYAN	Pos
2517	2/21/13	F	19.0	KANYAN	Pos
2518	2/21/13	M	0.6	MALIBA	Neg
2519	2/21/13	M	2.0	RWAKING	Neg
2520	2/21/13	F	15.0	BUGOYE	Neg
2521	2/21/13	F	60.0	KIRONGO	Pos
2522	2/21/13	M	22.0	RWAKING	Pos
2523	2/21/13	F	58.0	IZINGA	Neg
2524	2/21/13	F	2.0	KANYAN	Pos
2525	2/21/13	M	2.5	NYAKABUGHA	Neg
2526	2/21/13	M	15.0	MURAMBA	Neg
2527	2/21/13	F	0.6	BUGOYE	Neg
2528	2/21/13	M	5.0	MURAMBA	Pos
2529	2/22/13	M	1.1	MURAMBA	Pos
2530	2/22/13	M	0.5	MURAMBA	Neg
2531	2/22/13	M	4.0	KANYAN	Pos
2532	2/22/13	F	2.7	MURAMBA	Pos
2533	2/22/13	F	21.0	RWAKING	Neg
2534	2/22/13	F	22.0	IZINGA	Pos
2535	2/22/13	F	45.0	KANYAN	Neg
2536	2/22/13	F	0.3	MURAMBA	Neg

2537	2/22/13	M	18.0	KANYAN	Neg
2538	2/22/13	F	30.0	KANYAN	Neg
2539	2/22/13	F	2.0	BUGOYE	Pos
2540	2/22/13	F	31.0	KIHINDI	Neg
2541	2/22/13	M	6.0	KISANGA	Pos
2542	2/22/13	M	1.5	KIHINDI	Neg
2543	2/22/13	F	1.8	KIRONGO	Pos
2544	2/22/13	M	1.1	MURAMBA	Neg
2545	2/22/13	M	65.0	KANYAN	Pos
2546	2/22/13	M	22.0	KANYAN	Pos
2547	2/22/13	F	65.0	KIBIRIZI	Pos
2548	2/22/13	F	4.0	KISANZI	Neg
2549	2/22/13	F	16.0	NDUGHUTU	Pos
2550	2/22/13	F	12.0	ISULE	Pos
2551	2/22/13	F	0.5	MURAMBA	Neg
2552	2/22/13	F	30.0	NDUGHUTU	Pos
2553	2/22/13	F	26.0	MUHAMBO	Neg
2554	2/22/13	M	0.3	BUZIBWERA	Neg
2555	2/22/13	F	6.1	KANYAN	Pos
2556	2/22/13	F	30.0	MALIBA	Neg
2557	2/22/13	M	0.4	MAGHOMA	Neg
2558	2/22/13	F	2.1	BUNYANGONI	Neg
2559	2/22/13	M	2.0	BUZIBWERA	Neg
2560	2/22/13	F	0.3	BUNYANGONI	Neg
2561	2/22/13	F	30.0	BUNYANGONI	Neg
2562	2/22/13	F	0.6	MURAMBA	Neg
2563	2/22/13	F	1.7	KYANYA	Neg
2564	2/22/13	M	4.0	KIRONGO	Pos
2565	2/22/13	M	0.3	KYANYA	Neg
2566	2/22/13	F	24.0	MALIBA	Pos
2567	2/22/13	F	18.0	BUGOYE	Neg
2568	2/22/13	M	1.2	IHANI	Neg
2569	2/22/13	F	35.0	.	Neg
2570	2/22/13	M	4.0	KISOJO	Pos
2571	2/22/13	F	19.0	KATUMBA	Neg
2572	2/22/13	F	1.6	KATUMBA	Neg
2573	2/22/13	M	0.9	MUHAMBO	Neg
2574	2/26/13	F	18.0	BUGOYE	Neg
2575	2/26/13	F	1.8	BUGOYE	Neg
2576	2/26/13	M	0.7	BUGOYE	Neg
2577	2/26/13	F	12.0	BUNYANGONI	Neg
2578	2/26/13	F	2.3	KANYAN	Pos
2579	2/26/13	F	0.4	BUGOYE	Neg

2580	2/26/13	F	21.0	BUGOYE	Neg
2581	2/26/13	F	18.0	BUGOYE	Neg
2582	2/26/13	M	3.5	KAKINDO	Pos
2583	2/26/13	M	3.4	KYARUGOMOKA	Neg
2584	2/26/13	M	39.0	MUHAMBO	Neg
2585	2/26/13	M	58.0	KAKINDO	Neg
2586	2/26/13	M	10.0	BUGOYE	Pos
2587	2/26/13	F	1.7	BUNYANGONI	Neg
2588	2/26/13	M	42.0	BUGOYE	Pos
2589	2/26/13	M	0.5	BUGOYE	Neg
2590	2/26/13	M	6.0	IZINGA	Pos
2591	2/26/13	F	50.0	MALIBA	Neg
2592	2/26/13	F	17.0	BUGOYE	Pos
2593	2/26/13	M	38.0	IBANDA	Neg
2594	2/27/13	F	23.0	MURAMBA	Neg
2595	2/27/13	M	1.2	KANYAN	Neg
2596	2/27/13	F	9.0	IBANDA	Neg
2597	2/27/13	F	27.0	NDUGHUTU	Pos
2598	2/27/13	F	20.0	RWAKING	Neg
2599	2/27/13	M	19.0	KATUMBA	Neg
2600	2/27/13	F	39.0	NYAKARUNGU	Neg
2601	2/27/13	F	13.0	KIRULI	Neg
2602	2/27/13	F	8.5	MUHAMBO	Neg
2603	2/27/13	M	46.0	MURAMBA	Neg
2604	2/27/13	F	24.0	KATOOKE	Neg
2605	2/27/13	M	29.0	IZINGA	Neg
2606	2/27/13	F	7.0	KIRONGO	Pos
2607	2/27/13	F	12.0	KIRONGO	Pos
2608	2/27/13	F	35.0	KANYAN	Neg
2609	2/27/13	F	8.0	IZINGA	Pos
2610	2/27/13	M	20.0	BUGOYE	Pos
2611	2/27/13	F	28.0	RWAKING	Neg
2612	2/27/13	M	1.8	RWAKING	Neg
2613	2/27/13	F	30.0	MALIBA	Neg
2614	2/27/13	F	15.0	MURAMBA	Pos
2615	2/27/13	M	3.0	KYARUGOMOKA	Neg
2616	2/27/13	M	27.0	NYAKARUNGU	Neg
2617	2/27/13	M	5.0	NDUGHUTU	Pos
2618	2/27/13	F	7.0	NDUGHUTU	Pos
2619	2/27/13	F	15.0	KATOOKE	Neg
2620	2/27/13	M	4.0	BUGOYE	Pos
2621	2/28/13	F	16.0	BUGOYE	Pos
2622	2/28/13	F	46.0	BUGOYE	Neg

2623	2/28/13	M	5.0	BUGOYE	Neg
2624	2/28/13	M	6.0	KANYAN	Pos
2625	2/28/13	M	4.0	KANYAN	Pos
2626	2/28/13	F	3.0	BUGOYE	Neg
2627	2/28/13	F	5.0	KANYAN	Pos
2628	2/28/13	M	0.8	IHANI	Neg
2629	2/28/13	F	50.0	MURAMBA	Neg
2630	2/28/13	M	35.0	RWAKING	Neg
2631	2/28/13	F	31.0	BUGOYE	Neg
2632	2/28/13	F	40.0	BUNYANGONI	Neg
2633	2/28/13	M	30.0	BUGOYE	Neg
2634	2/28/13	M	1.3	BUGOYE	Neg
2635	2/28/13	F	0.5	KASANDERA	Neg
2636	2/28/13	F	40.0	RWAKING	Neg
2637	2/28/13	F	2.0	KYANYA	Pos
2638	2/28/13	F	2.0	BUNYANGONI	Neg
2639	2/28/13	M	26.0	KANYAN	Pos
2640	2/28/13	F	25.0	NDUGHUTU	Neg
2641	2/28/13	F	1.0	BUGOYE	Neg
2642	2/28/13	M	13.0	BUGOYE	Neg
2643	2/28/13	F	5.0	NDUGHUTU	Neg
2644	2/28/13	F	35.0	BUNYANGONI	Neg
2645	2/28/13	F	33.0	MALIBA	Neg
2646	2/28/13	F	4.0	IZINGA	Pos
2647	3/1/13	M	27.0	RWAKING	Neg
2648	3/1/13	F	22.0	BUNYANGONI	Neg
2649	3/1/13	F	5.0	KANYAN	Pos
2650	3/1/13	F	18.0	BUGOYE	Neg
2651	3/1/13	F	15.0	BUGOYE	Neg
2652	3/1/13	F	27.0	KANYAN	Pos
2653	3/1/13	M	1.5	KANYAN	Neg
2654	3/1/13	F	20.0	MURAMBA	Neg
2655	3/1/13	F	35.0	MALIBA	Pos
2656	3/1/13	F	1.5	KAKINDO	Neg
2657	3/1/13	F	17.0	BUGOYE	Pos
2658	3/1/13	M	3.0	MALIBA	Neg
2659	3/1/13	F	68.0	KANYAN	Pos
2660	3/1/13	F	5.0	KANYAN	Pos
2661	3/1/13	F	18.0	KAKINDO	Neg
2662	3/1/13	F	32.0	BUGOYE	Neg
2663	3/1/13	F	1.3	MAGHOMA	Neg
2664	3/1/13	M	2.1	BUZIBWERA	Neg
2665	3/1/13	F	20.0	KATOOKE	Neg

2666	3/1/13	M	5.0	IZINGA	Neg
2667	3/1/13	M	2.0	NDUGHUTU	Neg
2668	3/1/13	F	22.0	KISAMBA	Neg
2669	3/1/13	F	20.0	KATOOKE	Neg
2670	3/1/13	F	0.7	MURAMBA	Neg
2671	3/1/13	F	0.7	KANYAN	Neg
2672	3/1/13	F	2.1	KANYAN	Pos
2673	3/1/13	F	26.0	NDUGHUTU	Neg
2674	3/4/13	F	30.0	KANYAN	Neg
2675	3/4/13	F	24.0	IZINGA	Neg
2676	3/4/13	M	2.0	BUGOYE	Neg
2677	3/4/13	M	2.1	KANYAN	Pos
2678	3/4/13	F	30.0	BUNYANGONI	Pos
2679	3/4/13	M	24.0	IHANI	Neg
2680	3/4/13	M	9.0	KISANZI	Pos
2681	3/4/13	F	34.0	KANYAN	Neg
2682	3/4/13	F	1.5	KIBIRIZI	Neg
2683	3/4/13	M	1.8	MURAMBA	Neg
2684	3/4/13	M	8.0	KYANYA	Neg
2685	3/4/13	F	28.0	IBANDA	Neg
2686	3/4/13	M	0.7	MURAMBA	Neg
2687	3/4/13	F	24.0	MURAMBA	Neg
2688	3/4/13	M	4.5	NDUGHUTU	Neg
2689	3/4/13	M	0.5	MURAMBA	Neg
2690	3/4/13	F	0.1	NDUGHUTU	Neg
2691	3/4/13	M	58.0	KAKINDO	Neg
2692	3/4/13	F	40.0	MALIBA	Neg
2693	3/4/13	M	0.8	KATOOKE	Neg
2694	3/4/13	F	67.0	MURAMBA	Neg
2695	3/4/13	M	2.0	KANYAN	Pos
2696	3/4/13	M	37.0	MALIBA	Pos
2697	3/4/13	F	1.6	MURAMBA	Neg
2698	3/4/13	F	1.0	MALIBA	Neg
2699	3/4/13	M	0.8	MURAMBA	Neg
2700	3/4/13	F	18.0	KIBOTA	Neg
2701	3/4/13	F	1.3	MALIBA	Pos
2702	3/5/13	M	11.0	KANYAN	Pos
2703	3/5/13	F	35.0	BUGOYE	Neg
2704	3/5/13	F	0.8	BUGOYE	Neg
2705	3/5/13	F	12.0	BUGOYE	Neg
2706	3/5/13	F	22.0	NDUGHUTU	Neg
2707	3/5/13	M	3.0	BUNYANGONI	Neg
2708	3/5/13	F	15.0	BUGOYE	Neg

2709	3/5/13	M	7.0	MALIBA	Neg
2710	3/5/13	M	0.5	MALIBA	Neg
2711	3/5/13	F	23.0	KIBIRIZI	Neg
2712	3/5/13	M	5.0	MUKATI	Neg
2713	3/5/13	F	23.0	MURAMBA	Pos
2714	3/5/13	F	17.0	RWAKING	Pos
2715	3/5/13	F	2.5	BUGOYE	Pos
2716	3/5/13	M	7.5	KANYAN	Pos
2717	3/5/13	M	2.5	MURAMBA	Neg
2718	3/5/13	F	9.0	BUGOYE	Neg
2719	3/5/13	F	6.0	KANYAN	Pos
2720	3/5/13	M	13.0	BUGOYE	Neg
2721	3/5/13	F	24.0	KANYAN	Pos
2722	3/5/13	M	20.0	BUGOYE	Neg
2723	3/5/13	F	17.0	MALIBA	Neg
2724	3/5/13	F	37.0	MURAMBA	Neg
2725	3/5/13	M	2.5	RWAKING	Neg
2726	3/5/13	F	4.0	MURAMBA	Pos
2727	3/6/13	M	40.0	BUGOYE	Neg
2728	3/6/13	F	3.0	BUGOYE	Neg
2729	3/6/13	M	2.0	BUGOYE	Neg
2730	3/6/13	F	82.0	RWAKING	Neg
2731	3/6/13	F	2.0	MURAMBA	Pos
2732	3/6/13	M	4.0	MALIBA	Neg
2733	3/6/13	F	3.0	IZINGA	Neg
2734	3/6/13	F	8.5	KATUMBA	Neg
2735	3/6/13	F	5.0	MUKATI	Neg
2736	3/6/13	F	18.0	BUGOYE	Neg
2737	3/6/13	F	2.6	BUGOYE	Neg
2738	3/6/13	F	26.0	KARAMBI	Neg
2739	3/6/13	M	30.0	NDUGHUTU	Neg
2740	3/6/13	M	0.1	MURAMBA	Neg
2741	3/6/13	F	60.0	MALIBA	Neg
2742	3/6/13	M	1.2	BUNYANGONI	Neg
2743	3/6/13	F	6.0	NYAKALENGIJO	Pos
2744	3/7/13	F	60.0	BUGOYE	Neg
2745	3/7/13	F	26.0	BUGOYE	Neg
2746	3/7/13	F	61.0	IZINGA	Neg
2747	3/7/13	F	100.0	MALIBA	Neg
2748	3/7/13	F	17.0	BUGOYE	Neg
2749	3/7/13	F	29.0	MALIBA	Neg
2750	3/7/13	F	38.0	KANYAN	Pos
2751	3/7/13	M	2.0	IZINGA	Pos

2752	3/7/13	M	19.0	BUGOYE	Neg
2753	3/7/13	F	56.0	KASESE	Neg
2754	3/7/13	F	12.0	MALIBA	Pos
2755	3/7/13	F	13.0	MALIBA	Pos
2756	3/7/13	M	14.0	MALIBA	Pos
2757	3/7/13	F	3.5	KANYAN	Neg
2758	3/7/13	M	15.0	BUGOYE	Neg
2759	3/7/13	F	7.0	KANYAN	Pos
2760	3/7/13	F	3.0	MALIBA	Pos
2761	3/7/13	F	12.0	KANYAN	Pos
2762	3/7/13	F	39.0	RWAKING	Neg
2763	3/7/13	M	4.0	KANYAN	Pos
2764	3/11/13	F	65.0	KISAMBA	Neg
2765	3/11/13	F	15.0	BUGOYE	Neg
2766	3/11/13	M	3.5	RWAKING	Neg
2767	3/11/13	F	55.0	KIKOKERA	Neg
2768	3/11/13	F	15.0	BUGOYE	Neg
2769	3/11/13	M	2.2	KANYAN	Pos
2770	3/11/13	F	3.0	MALIBA	Neg
2771	3/11/13	F	29.0	BUHUNGA	Neg
2772	3/11/13	F	18.0	MURAMBA	Neg
2773	3/11/13	M	4.0	NYAKARUNGU	Neg
2774	3/11/13	F	30.0	KANYAN	Neg
2775	3/11/13	F	17.0	KYARUGOMOKA	Neg
2776	3/11/13	F	14.0	MURAMBA	Pos
2777	3/11/13	F	33.0	IZINGA	Neg
2778	3/11/13	M	17.0	IZINGA	Neg
2779	3/11/13	M	12.0	KANYAN	Pos
2780	3/11/13	M	5.0	KIBOTA	Neg
2781	3/11/13	F	46.0	IZINGA	Pos
2782	3/11/13	M	2.0	KAKINDO	Neg
2783	3/11/13	F	16.0	MURAMBA	Neg
2784	3/11/13	F	15.0	KANYAN	Neg
2785	3/11/13	F	19.0	MURAMBA	Neg
2786	3/11/13	F	0.5	BUNYANGONI	Neg
2787	3/11/13	M	3.0	BUGOYE	Neg
2788	3/11/13	M	2.5	KYARUGOMOKA	Pos
2789	3/11/13	M	50.0	BULINDUGURU	Neg
2790	3/11/13	M	64.0	NDUGHUTU	Neg
2791	3/11/13	F	0.3	KIRULI	Neg
2792	3/11/13	M	7.0	BUNYANGONI	Neg
2793	3/12/13	M	26.0	RWAKING	Neg
2794	3/12/13	M	54.0	BUGOYE	Neg

2795	3/12/13	F	17.0	BUGOYE	Neg	
2796	3/12/13	M	30.0	KATUMBA	Neg	
2797	3/12/13	F	9.0	KYARUGOMOKA	Pos	
2798	3/12/13	F	10.0	BUNYANGONI	Neg	
2799	3/12/13	F	66.0	BUNYANGONI	Neg	
2800	3/12/13	F	80.0	BUGOYE	Neg	
2801	3/12/13	F	5.0	BUGOYE	Neg	
2802	3/12/13	M	19.0	MALIBA	Neg	
2803	3/12/13	F	18.0	BUGOYE	Neg	
2804	3/12/13	F	29.0	KYANYA	Neg	
2805	3/12/13	F	1.3	BUZIBWERA	Neg	
2806	3/12/13	M	13.0	NDUGHUTU	Neg	
2807	3/12/13	F	24.0	IBANDA		Neg
2808	3/12/13	M	17.0	BUGOYE	Pos	
2809	3/12/13	M	19.0	KATOOKE	Pos	
2810	3/12/13	F	14.0	BUGOYE	Pos	
2811	3/13/13	M	3.0	IZINGA		Neg
2812	3/13/13	F	35.0	MALIBA		Neg
2813	3/13/13	M	19.0	MURAMBA		Neg
2814	3/13/13	F	1.4	KANYAN		Neg
2815	3/13/13	M	10.0	NDUGHUTU		Pos
2816	3/13/13	F	15.0	NDUGHUTU		Pos
2817	3/13/13	F	40.0	MURAMBA	Pos	
2818	3/13/13	F	65.0	MUHAMBO	Pos	
2819	3/13/13	M	2.5	KIBOTA	Pos	
2820	3/13/13	F	62.0	KIBOTA	Neg	
2821	3/13/13	F	15.0	BUGOYE	Neg	
2822	3/13/13	F	32.0	BUNYANGONI	Neg	
2823	3/13/13	F	10.0	KANYAN	Pos	
2824	3/13/13	F	3.0	BUNYANGONI	Pos	
2825	3/13/13	M	10.0	KANYAN	Pos	
2826	3/13/13	F	17.0	IBANDA	Neg	
2827	3/13/13	F	0.7	IBANDA	Pos	
2828	3/13/13	F	1.3	MIHUNGA	Neg	
2829	3/13/13	M	4.0	NDUGHUTU	Neg	
2830	3/13/13	F	62.0	MURAMBA	Neg	
2831	3/13/13	F	23.0	BUNYANGONI	Neg	
2832	3/13/13	M	12.0	RWAKING	Pos	
2833	3/13/13	F	5.0	NDUGHUTU	Pos	
2834	3/13/13	M	12.0	KANYAN	Neg	
2835	3/13/13	M	6.0	NDUGHUTU	Neg	
2836	3/14/13	M	5.0	MURAMBA	Neg	
2837	3/14/13	F	10.0	MURAMBA	Pos	

2838	3/14/13	F	21.0	MURAMBA	Neg	
2839	3/14/13	M	18.0	MUHAMBO	Pos	
2840	3/14/13	F	12.0	KIRONGO	Pos	
2841	3/14/13	M	1.5	BUZIBWERA	Neg	
2842	3/14/13	F	50.0	KAYEMBE	Neg	
2843	3/14/13	F	39.0	KAKINDO	Neg	
2844	3/14/13	F	48.0	BUHWEZA	Neg	
2845	3/14/13	M	0.3	RWAKING	Neg	
2846	3/14/13	M	7.0	NDUGHUTU	Neg	
2847	3/14/13	F	44.0	NDUGHUTU	Neg	
2848	3/14/13	F	6.0	NYAKARUNGU	Pos	
2849	3/14/13	M	8.0	NYAKARUNGU	Pos	
2850	3/14/13	F	4.0	NYAKARUNGU	Neg	
2851	3/14/13	M	4.0	NDUGHUTU	Neg	
2852	3/14/13	F	3.5	MURAMBA	Pos	
2853	3/14/13	F	32.0	MURAMBA	Neg	
2854	3/14/13	M	51.0	BUNYANGONI	Neg	
2855	3/14/13	F	25.0	.	Neg	
2856	3/14/13	F	13.0	MURAMBA	Neg	
2857	3/14/13	M	20.0	KIKOKERA	Neg	
2858	3/14/13	F	19.0	BUNYANGONI	Neg	
2859	3/15/13	M	5.0	BUGOYE	Pos	
2860	3/15/13	F	25.0	MALIBA	Pos	
2861	3/15/13	F	11.0	BUGOYE		Pos
2862	3/15/13	F	22.0	MURAMBA		Pos
2863	3/15/13	F	20.0	NYAKALENGIJO		Pos
2864	3/15/13	F	50.0	MALIBA		Pos
2865	3/15/13	F	60.0	KYANYA	Neg	Neg
2866	3/18/13	F	19.0	BUGOYE	Neg	Neg
2867	3/18/13	F	20.0	MURAMBA		Pos
2868	3/18/13	F	5.0	RWAKING		Pos
2869	3/18/13	M	23.0	MALIBA		Pos
2870	3/18/13	F	75.0	BULYANDEKE	Neg	
2871	3/18/13	F	38.0	RWAKING	Neg	
2872	3/18/13	M	60.0	KATUMBA	Neg	
2873	3/18/13	F	6.5	MURAMBA	Neg	
2874	3/18/13	F	41.0	KYANYA	Neg	
2875	3/18/13	F	4.0	KYANYA	Neg	
2876	3/18/13	F	34.0	NYAKARUNGU	Neg	
2877	3/18/13	M	23.0	IZINGA	Pos	
2878	3/18/13	F	1.7	KATUMBA	Neg	
2879	3/18/13	F	65.0	NYAKARUNGU	Neg	
2880	3/18/13	F	8.0	KANYAN	Neg	

2881	3/18/13	F	28.0	NYAKARUNGU	Neg	
2882	3/18/13	M	1.7	BUGOYE	Neg	
2883	3/18/13	F	13.0	BUGOYE	Neg	
2884	3/18/13	M	45.0	NYAKARUNGU	Neg	
2885	3/18/13	M	44.0	KATABELKERE	Neg	
2886	3/18/13	F	19.0	KATOOKE	Pos	
2887	3/18/13	F	13.0	BUGOYE	Pos	
2888	3/18/13	F	35.0	MALIBA	Neg	
2889	3/18/13	F	0.3	MALIBA	Neg	
2890	3/18/13	M	6.0	KYARUGOMOKA	Neg	Neg
2891	3/18/13	F	25.0	KAMABWE	Neg	
2892	3/18/13	F	50.0	NDUGHUTU	Neg	
2893	3/18/13	F	20.0	BUGOYE	Pos	
2894	3/18/13	M	8.0	MUKATI	Pos	
2895	3/18/13	M	23.0	MUKATI	Pos	
2896	3/18/13	F	1.3	BUNYANGONI	Neg	
2897	3/18/13	F	34.0	KYABIKULE	Neg	
2898	3/18/13	M	4.0	BUGOYE	Neg	
2899	3/18/13	F	33.0	IBANDA	Neg	
2900	3/18/13	F	1.7	KISANZI	Neg	
2901	3/18/13	F	9.0	BUGOYE	Neg	
2902	3/18/13	M	5.0	BUGOYE	Neg	
2903	3/18/13	F	6.0	BUGOYE	Neg	
2904	3/18/13	F	20.0	RWAKING	Neg	
2905	3/18/13	F	8.0	MURAMBA	Pos	
2906	3/18/13	F	0.8	MALIBA	Neg	
2907	3/18/13	M	4.0	BUNYANGONI	Neg	
2908	3/18/13	M	1.3	BUNYANGONI	Neg	
2909	3/18/13	F	1.4	MAGHOMA	Neg	
2910	3/19/13	F	23.0	BUGOYE		Pos
2911	3/19/13	F	15.0	BUGOYE		Neg
2912	3/19/13	F	39.0	NDUGHUTU		Pos
2913	3/19/13	M	50.0	RWAKING		Pos
2914	3/19/13	F	6.0	RWAKING		Pos
2915	3/19/13	M	1.1	BUZIBWERA	Neg	Neg
2916	3/19/13	F	70.0	BUGOYE	Pos	Pos
2917	3/19/13	F	10.0	BUGOYE		Neg
2918	3/19/13	F	50.0	MALIBA		Neg
2919	3/19/13	F	2.5	MURAMBA		Neg
2920	3/19/13	M	6.1	KIBIRIZI		Pos
2921	3/19/13	M	50.0	BUNYANGONI		Pos
2922	3/19/13	F	38.0	KATUMBA		Pos
2923	3/19/13	M	3.0	BUGOYE		Neg

2924	3/19/13	F	20.0	BUNYANGONI		Neg
2925	3/19/13	F	8.0	IZINGA		Pos
2926	3/19/13	F	24.0	KISAMBA	Pos	
2927	3/19/13	M	1.3	KATOOKE		Neg
2928	3/19/13	M	5.0	KANYAN		Pos
2929	3/19/13	F	19.0	KATUMBA		Neg
2930	3/20/13	F	44.0	NDUGHUTU		Pos
2931	3/20/13	F	18.0	BUNYANGONI		Neg
2932	3/20/13	F	1.5	RWAKING		Pos
2933	3/20/13	M	1.5	MALIBA	Neg	
2934	3/20/13	F	27.0	MALIBA	Neg	
2935	3/20/13	F	6.0	RWAKING		Pos
2936	3/20/13	M	2.0	BUZIBWERA		Pos
2937	3/20/13	F	23.0	IZINGA		Pos
2938	3/20/13	F	56.0	MALIBA		Pos
2939	3/20/13	F	16.0	MALIBA		Pos
2940	3/20/13	F	13.0	NDUGHUTU		Pos
2941	3/20/13	F	20.0	MALIBA		Neg
2942	3/20/13	F	12.0	BUNYANGONI		Neg
2943	3/20/13	F	16.0	KIRULI		Neg
2944	3/20/13	F	24.0	MALIBA		Pos
2945	3/20/13	F	0.4	BULYANDEKE		Neg
2946	3/20/13	M	8.0	BULYANDEKE		Neg
2947	3/20/13	F	27.0	BULYANDEKE		Neg
2948	3/20/13	F	0.7	BUNYANGONI		Neg
2949	3/20/13	M	14.0	KATOOKE		Neg
2950	3/20/13	M	60.0	MALIBA		Pos
2951	3/20/13	F	4.0	MURAMBA		Neg
2952	3/20/13	M	52.0	KIRULI	Pos	
2953	3/20/13	F	25.0	MURAMBA		Pos
2954	3/20/13	F	40.0	KYARUGOMOKA		Neg
2955	3/20/13	F	5.0	KYARUGOMOKA		Pos
2956	3/20/13	F	17.0	BUHUNGA		Neg
2957	3/20/13	M	5.0	BUHUNGA		Pos
2958	3/20/13	F	14.0	.		Pos
2959	3/20/13	M	32.0	IBANDA		Pos
2960	3/20/13	M	0.2	MAGHOMA		Neg
2961	3/20/13	M	1.0	.		Neg
2962	3/21/13	M	22.0	KANYAN		Neg
2963	3/21/13	F	0.8	BUGOYE	Neg	Neg
2964	3/21/13	F	2.5	KANYAN		Neg
2965	3/21/13	M	42.0	IZINGA		Neg
2966	3/21/13	F	32.0	MALIBA		Neg

2967	3/21/13	M	0.1	KANYAN	Neg
2968	3/21/13	M	5.0	KATEBE	Pos
2969	3/21/13	F	27.0	KIKOKERA	Neg
2970	3/21/13	M	1.6	KATOOKE	Neg
2971	3/21/13	M	0.5	KANYAN	Pos
2972	3/21/13	F	10.0	IZINGA	Neg
2973	3/21/13	M	4.0	MURAMBA	Neg
2974	3/21/13	M	1.8	NYAKARUNGU	Neg
2975	3/21/13	M	2.0	KAKIRO	Neg
2976	3/21/13	M	5.0	RWAKING	Neg
2977	3/21/13	F	22.0	KANYAN	Neg
2978	3/21/13	F	28.0	ISULE	Neg
2979	3/21/13	F	18.0	KANYAN	Pos
2980	3/21/13	F	23.0	NDUGHUTU	Pos
2981	3/21/13	F	1.5	BUHUNGA	Neg
2982	3/22/13	M	15.0	KANYAN	Pos
2983	3/22/13	F	20.0	KANYAN	Neg
2984	3/22/13	M	4.0	BUGOYE	Pos
2985	3/22/13	M	2.0	NDUGHUTU	Pos
2986	3/22/13	F	19.0	KATUMBA	Pos
2987	3/22/13	M	18.0	KATUMBA	Neg
2988	3/22/13	M	45.0	MALIBA	Neg
2989	3/22/13	F	18.0	IZINGA	Neg
2990	3/25/13	M	26.0	NYAKARUNGU	Pos
2991	3/25/13	M	1.5	IBANDA	Neg
2992	3/25/13	F	19.0	IBANDA	Neg
2993	3/25/13	F	30.0	KANYAN	Pos
2994	3/25/13	M	25.0	IZINGA	Pos
2995	3/25/13	F	17.0	BUGOYE	Pos
2996	3/25/13	F	35.0	NYAKABUGHA	Neg
2997	3/25/13	F	0.4	MAGHOMA	Neg
2998	3/25/13	F	2.0	NYAKARUNGU	Pos
2999	3/25/13	F	1.1	NYAKARUNGU	Pos
3000	3/25/13	M	2.0	IZINGA	Neg
3001	3/25/13	F	4.0	IZINGA	Neg
3002	3/25/13	F	23.0	RWAKING	Neg
3003	3/25/13	F	20.0	KATUMBA	Pos
3004	3/26/13	F	0.3	KIRONGO	Pos
3005	3/26/13	F	24.0	BUGOYE	Pos
3006	3/26/13	M	2.3	BUGOYE	Pos
3007	3/26/13	F	1.5	BUGOYE	Pos
3008	3/26/13	F	19.0	BUNYANGONI	Pos
3009	3/26/13	M	12.0	KIHINDI	Pos

3010	3/26/13	M	38.0	NYAKARUNGU	Pos
3011	3/26/13	F	24.0	IZINGA	Neg
3012	3/26/13	F	23.0	KISAMBA	Neg
3013	3/26/13	F	72.0	BUNYANGONI	Neg
3014	3/26/13	M	6.0	BUGOYE	Pos
3015	3/26/13	M	27.0	BUGOYE	Neg
3016	3/26/13	M	29.0	BUGOYE	Neg
3017	3/26/13	F	8.0	.	Neg
3018	3/27/13	F	55.0	.	Neg
3019	3/27/13	F	0.8	.	Pos
3020	3/27/13	M	7.0	.	Pos
3021	3/27/13	M	1.9	.	Neg
3022	3/27/13	M	4.0	MURAMBA	Neg
3023	3/27/13	F	24.0	NDUGHUTU	Neg
3024	3/27/13	F	0.5	MURAMBA	Pos
3025	3/27/13	M	32.0	NYAKARUNGU	Neg
3026	3/27/13	F	0.8	.	Neg
3027	3/27/13	F	3.0	.	Neg
3028	3/27/13	M	2.3	IZINGA	Pos
3029	3/27/13	F	0.4	MURAMBA	Pos
3030	3/27/13	F	2.0	MALIBA	Pos
3031	3/27/13	M	1.0	KATOOKE	Neg
3032	3/27/13	M	0.3	KATUMBA	Neg
3033	3/27/13	F	0.3	BUNYANGONI	Pos
3034	3/28/13	M	0.6	KANYAN	Neg
3035	3/28/13	M	60.0	BUGOYE	Neg
3036	3/28/13	F	1.3	MAGHOMA	Pos
3037	3/28/13	F	49.0	BUNYANGONI	Pos
3038	3/28/13	F	24.0	MURAMBA	Pos
3039	3/28/13	F	7.0	BUGOYE	Pos
3040	3/28/13	F	46.0	IZINGA	Pos
3041	3/28/13	F	27.0	RWAKING	Pos
3042	3/28/13	M	18.0	MURAMBA	Neg
3043	3/28/13	F	30.0	BUGOYE	Neg
3044	3/28/13	F	40.0	BUGOYE	Neg
3045	4/2/13	M	35.0	BUGOYE	Pos
3046	4/2/13	F	66.0	NDUGHUTU	Pos
3047	4/2/13	M	40.0	BUGOYE	Neg
3048	4/2/13	F	8.0	MURAMBA	Neg
3049	4/2/13	F	35.0	IBANDA	Neg
3050	4/2/13	F	30.0	BUGOYE	Pos
3051	4/2/13	M	40.0	BUGOYE	Pos
3052	4/2/13	M	68.0	MUHAMBO	Neg

3053	4/2/13	F	56.0	BUNYANGONI	Neg
3054	4/2/13	M	14.0	KIRULI	Neg
3055	4/2/13	M	49.0	NDUGHUTU	Neg
3056	4/2/13	F	18.0	MAGHOMA	Neg
3057	4/2/13	F	21.0	BIKONE	Neg
3058	4/2/13	M	1.0	BUNYANGONI	Neg
3059	4/2/13	F	28.0	BUGOYE	Neg
3060	4/3/13	F	1.5	MUHAMBO	Neg
3061	4/3/13	F	11.0	RWAKING	Pos
3062	4/3/13	M	1.0	KANYAN	Neg
3063	4/3/13	M	14.0	MUBUKU	Neg
3064	4/3/13	F	32.0	MUBUKU	Neg
3065	4/3/13	F	19.0	BUNYANGONI	Neg
3066	4/3/13	F	45.0	NDUGHUTU	Neg
3067	4/3/13	M	3.0	MURAMBA	Neg
3068	4/3/13	F	6.0	IZINGA	Pos
3069	4/3/13	M	5.0	IZINGA	Pos
3070	4/3/13	M	13.0	KEMIHOKO	Neg
3071	4/3/13	F	33.0	KANYAN	Neg
3072	4/3/13	F	40.0	IZINGA	Neg
3073	4/3/13	M	0.4	KATOOKE	Neg
3074	4/3/13	M	0.4	MALIBA	Neg
3075	4/3/13	F	3.0	KYANYA	Neg
3076	4/3/13	F	12.0	KAKINDO	Neg
3077	4/3/13	F	0.4	NDUGHUTU	Neg
3078	4/3/13	M	1.3	IZINGA	Pos
3079	4/3/13	F	33.0	IZINGA	Neg
3080	4/3/13	M	0.8	IZINGA	Neg
3081	4/3/13	F	27.0	KIRULI	Neg
3082	4/3/13	M	2.0	MURAMBA	Pos
3083	4/4/13	F	17.0	IZINGA	Neg
3084	4/4/13	F	1.3	KATUMBA	Neg
3085	4/4/13	F	30.0	NYAKABUGHA	Neg
3086	4/4/13	F	15.0	BUGOYE	Neg
3087	4/4/13	F	19.0	KYARUGOMOKA	Pos
3088	4/4/13	M	4.0	IZINGA	Pos
3089	4/4/13	M	1.3	IZINGA	Pos
3090	4/4/13	F	25.0	MURAMBA	Pos
3091	4/4/13	F	22.0	KANYAN	Pos
3092	4/4/13	F	0.4	MALIBA	Pos
3093	4/4/13	M	6.0	IZINGA	Pos
3094	4/4/13	M	10.0	KATUMBA	Pos
3095	4/4/13	M	3.0	NDUGHUTU	Pos

3096	4/4/13	F	2.0	NDUGHUTU		Pos
3097	4/4/13	F	50.0	NYAKALENGIJO		Pos
3098	4/4/13	F	30.0	NDUGHUTU		Neg
3099	4/4/13	F	47.0	MUBUKU		Neg
3100	4/5/13	M	2.5	MALIBA		Pos
3101	4/5/13	F	3.0	NDUGHUTU		Pos
3102	4/5/13	F	20.0	BUNYANGONI		Pos
3103	4/5/13	F	18.0	BUGOYE		Neg
3104	4/5/13	M	38.0	BUGOYE		Neg
3105	4/5/13	M	4.0	IZINGA		Neg
3106	4/5/13	M	24.0	KANYAN		Pos
3107	4/5/13	F	30.0	KANYAN		Neg
3108	4/5/13	M	0.8	BUGOYE		Neg
3109	4/5/13	M	0.4	BUGOYE		Neg
3110	4/5/13	F	21.0	MALIBA		Neg
3111	4/5/13	F	19.0	IZINGA		Pos
3112	4/5/13	F	18.0	NDUGHUTU		Neg
3113	4/5/13	M	11.0	BUGOYE		Pos
3114	4/5/13	F	0.7	NDUGHUTU		Neg
3115	4/5/13	F	0.1	NDUGHUTU		Pos
3116	4/5/13	M	1.3	NDUGHUTU	Neg	Neg
3117	4/5/13	F	28.0	NDUGHUTU		Pos
3118	4/5/13	F	9.0	BUGOYE		Pos
3119	4/5/13	M	12.0	MURAMBA		Pos
3120	4/5/13	M	13.0	MALIBA		Pos
3121	4/5/13	M	0.6	MALIBA		Neg
3122	4/5/13	F	22.0	KISORO		Pos
3123	4/8/13	F	38.0	MURAMBA		Pos
3124	4/8/13	F	0.3	IZINGA		Neg
3125	4/8/13	M	39.0	IBANDA		Pos
3126	4/8/13	F	14.0	BUGOYE		Neg
3127	4/8/13	F	2.0	BUNYANGONI		Pos
3128	4/8/13	F	21.0	BUGOYE		Neg
3129	4/8/13	M	1.0	BUGOYE		Neg
3130	4/8/13	M	40.0	IBANDA		Neg
3131	4/8/13	F	25.0	KYANYA		Neg
3132	4/8/13	F	20.0	KYANYA		Pos
3133	4/8/13	F	0.6	BUNYANGONI		Neg
3134	4/8/13	M	9.0	NDUGHUTU		Neg
3135	4/8/13	M	1.0	MALIBA		Pos
3136	4/8/13	F	23.0	BUGOYE		Neg
3137	4/9/13	M	80.0	RWAKING		Neg
3138	4/9/13	F	30.0	KYARUGOMOKA		Pos

3139	4/9/13	F	0.3	IZINGA		Pos
3140	4/9/13	M	3.0	BUGOYE		Neg
3141	4/9/13	F	1.3	IZINGA		Neg
3142	4/9/13	M	0.4	KIHINDI		Pos
3143	4/9/13	F	1.1	NYANGONGE		Pos
3144	4/9/13	F	6.0	RWAKING		Neg
3145	4/9/13	M	1.0	MURAMBA		Neg
3146	4/10/13	F	10.0	BUGOYE		Pos
3147	4/10/13	F	4.0	BUGOYE		Pos
3148	4/10/13	F	66.0	MURAMBA		Pos
3149	4/10/13	M	1.0	IZINGA		Neg
3150	4/10/13	M	0.8	BUNYANGONI		Neg
3151	4/10/13	F	10.0	BUGOYE	Pos	
3152	4/10/13	F	22.0	NYAKABUGHA	Neg	
3153	4/10/13	F	1.0	NYAKABUGHA	Neg	
3154	4/10/13	F	19.0	KATUMBA		Neg
3155	4/10/13	F	25.0	KATUMBA		Neg
3156	4/10/13	M	20.0	MALIBA		Pos
3157	4/11/13	F	5.0	BUGOYE	Pos	Pos
3158	4/11/13	F	26.0	RWAKING	Neg	
3159	4/11/13	F	30.0	MALIBA		Neg
3160	4/11/13	F	19.0	BUGOYE	Pos	
3161	4/11/13	F	44.0	NDUGHUTU	Neg	
3162	4/11/13	F	31.0	NDUGHUTU	Pos	
3163	4/11/13	M	28.0	KIKOKERA	Neg	Neg
3164	4/11/13	M	2.3	BUNYANGONI	Neg	
3165	4/11/13	F	10.0	IZINGA	Pos	
3166	4/11/13	F	15.0	BUNYANGONI	Neg	
3167	4/11/13	F	12.0	IZINGA	Pos	
3168	4/11/13	F	75.0	KATOOKE	Neg	
3169	4/11/13	F	1.0	MURAMBA	Neg	
3170	4/11/13	M	30.0	KIRULI	Neg	
3171	4/11/13	F	19.0	KYARUGOMOKA	Pos	
3172	4/12/13	F	19.0	BUGOYE	Pos	
3173	4/12/13	F	35.0	BUGOYE	Neg	
3174	4/12/13	M	29.0	IBANDA	Neg	
3175	4/12/13	F	6.0	RWAKING	Pos	
3176	4/12/13	M	0.3	MAGHOMA	Neg	
3177	4/12/13	M	0.5	BUNYANGONI	Neg	
3178	4/12/13	F	18.0	BUGOYE	Neg	
3179	4/12/13	M	1.5	NDUGHUTU	Neg	
3180	4/12/13	M	1.8	NDUGHUTU	Neg	
3181	4/12/13	F	27.0	KIKOKERA	Neg	

3182	4/12/13	F	1.3	IZINGA	Neg	
3183	4/12/13	F	3.0	RWAKING	Pos	
3184	4/12/13	M	1.0	KATUMBA		Neg
3185	4/12/13	F	18.0	BUZIBWERA	Pos	
3186	4/12/13	F	6.0	KANYAN	Pos	
3187	4/12/13	F	23.0	MAGHOMA	Neg	
3188	4/12/13	F	19.0	NDUGHUTU		Pos
3189	4/15/13	F	56.0	KYARUGOMOKA		Neg
3190	4/15/13	F	16.0	BUGOYE		Neg
3191	4/15/13	F	6.0	BUGOYE		Pos
3192	4/15/13	F	7.0	RWAKING		Pos
3193	4/15/13	F	1.5	RWAKING		Neg
3194	4/15/13	F	25.0	NDUGHUTU		Pos
3195	4/15/13	F	2.6	NDUGHUTU	Neg	
3196	4/15/13	M	20.0	BUGOYE	Pos	
3197	4/15/13	F	2.5	NDUGHUTU	Neg	
3198	4/15/13	M	22.0	RWAKING	Pos	
3199	4/15/13	M	4.5	MALIBA	Neg	
3200	4/15/13	F	0.3	MURAMBA	Neg	
3201	4/15/13	F	0.8	MURAMBA	Neg	
3202	4/15/13	F	60.0	NYANGONGE	Neg	
3203	4/15/13	M	8.0	BUGOYE	Neg	
3204	4/15/13	F	22.0	BUGOYE	Pos	
3205	4/15/13	F	60.0	BUGOYE	Neg	
3206	4/15/13	M	22.0	KANYAN	Neg	
3207	4/15/13	F	19.0	IZINGA	Neg	
3208	4/15/13	F	5.0	NDUGHUTU	Neg	
3209	4/15/13	F	0.8	NDUGHUTU	Neg	
3210	4/15/13	F	40.0	MALIBA	Neg	
3211	4/15/13	F	1.0	BUGOYE		Pos
3212	4/15/13	F	2.5	KYARUGOMOKA		Pos
3213	4/16/13	M	40.0	KIRONGO	Neg	
3214	4/16/13	F	23.0	MURAMBA	Neg	
3215	4/16/13	F	25.0	KANYAN	Neg	
3216	4/16/13	M	2.5	IZINGA	Neg	
3217	4/16/13	F	21.0	MALIBA	Pos	
3218	4/16/13	M	6.0	KIRONGO	Pos	
3219	4/16/13	M	6.0	KYARUGOMOKA	Pos	
3220	4/16/13	F	14.0	MALIBA	Neg	
3221	4/16/13	F	42.0	MURAMBA	Neg	
3222	4/16/13	F	6.0	NDUGHUTU	Neg	
3223	4/16/13	M	6.0	KAKINDO	Pos	
3224	4/16/13	F	2.0	KIHINDI	Neg	

3225	4/16/13	F	2.0	KAKINDO	Pos	
3226	4/16/13	F	0.6	KATOOKE	Neg	
3227	4/17/13	F	4.5	BUGOYE	Neg	
3228	4/17/13	F	40.0	KIRONGO	Neg	
3229	4/17/13	M	5.0	IZINGA	Pos	
3230	4/17/13	F	20.0	IZINGA	Pos	
3231	4/17/13	F	34.0	KYARUGOMOKA	Pos	
3232	4/17/13	F	16.0	KANYAN	Pos	
3233	4/17/13	F	12.0	IZINGA	Neg	
3234	4/17/13	F	24.0	NYAKARUNGU	Pos	
3235	4/17/13	M	7.0	MURAMBA	Pos	
3236	4/17/13	F	5.0	NDUGHUTU	Pos	
3237	4/17/13	M	2.0	NDUGHUTU	Pos	
3238	4/17/13	F	13.0	NDUGHUTU	Pos	
3239	4/17/13	F	0.8	KIHINDI	Neg	
3240	4/17/13	F	0.6	RWAKING	Neg	
3241	4/17/13	F	0.7	KIHINDI	Neg	
3242	4/17/13	F	18.0	BUGOYE	Pos	
3243	4/17/13	M	42.0	IZINGA	Neg	
3244	4/17/13	M	10.0	KANYAN	Pos	
3245	4/17/13	M	2.5	RWAKING	Pos	
3246	4/17/13	F	1.3	KISANZI	Neg	
3247	4/17/13	F	0.8	RWAKING	Neg	
3248	4/17/13	F	34.0	MURAMBA	Neg	
3249	4/17/13	F	30.0	NDUGHUTU	Pos	
3250	4/17/13	F	20.0	IBANDA	Neg	
3251	4/17/13	F	3.5	BUZIBWERA	Pos	
3252	4/17/13	F	45.0	BUNYANGONI	Neg	
3253	4/17/13	M	1.3	NDUGHUTU	Neg	
3254	4/17/13	F	2.0	RWAKING	Pos	
3255	4/17/13	F	32.0	MURAMBA	Neg	
3256	4/18/13	F	4.0	MALIBA	Pos	
3257	4/18/13	F	1.4	NDUGHUTU	Neg	
3258	4/18/13	M	0.5	BUGOYE	Pos	
3259	4/18/13	M	3.0	MALIBA	Pos	
3260	4/18/13	F	11.0	MALIBA	Neg	
3261	4/18/13	F	1.3	MURAMBA	Neg	
3262	4/18/13	F	11.0	NDUGHUTU	Neg	
3263	4/18/13	M	7.0	MALIBA		Neg
3264	4/18/13	M	1.2	BUZIBWERA	Neg	
3265	4/18/13	F	15.0	BUGOYE	Neg	
3266	4/18/13	M	2.0	BUZIBWERA	Pos	
3267	4/18/13	M	3.0	BUZIBWERA	Pos	

3268	4/18/13	M	3.0	MALIBA	Neg	
3269	4/18/13	F	4.0	BUNYANGONI	Neg	
3270	4/18/13	M	25.0	BUNYANGONI	Pos	
3271	4/19/13	M	22.0	BUNYANGONI	Pos	
3272	4/19/13	M	24.0	IZINGA	Pos	
3273	4/19/13	F	0.8	KISANZI	Neg	
3274	4/19/13	M	60.0	MALIBA	Pos	
3275	4/19/13	F	32.0	MALIBA	Pos	
3276	4/19/13	F	55.0	MALIBA	Pos	
3277	4/19/13	F	30.0	KISANZI	Pos	
3278	4/19/13	F	23.0	MUHAMBO	Neg	
3279	4/19/13	F	25.0	KANYAN	Pos	
3280	4/19/13	F	30.0	KYANJIMA	Neg	
3281	4/19/13	F	23.0	RWAKING	Neg	
3282	4/19/13	F	11.0	IZINGA	Neg	
3283	4/19/13	F	15.0	MURAMBA	Neg	
3284	4/19/13	F	60.0	KANYAN	Pos	
3285	4/22/13	F	18.0	MURAMBA	Pos	
3286	4/22/13	F	19.0	MURAMBA	Pos	
3287	4/22/13	M	50.0	MURAMBA	Neg	
3288	4/22/13	M	24.0	MURAMBA	Pos	
3289	4/22/13	F	22.0	MALIBA	Pos	
3290	4/22/13	F	26.0	MURAMBA	Neg	
3291	4/22/13	F	9.0	MURAMBA	Pos	
3292	4/22/13	F	35.0	BUGOYE	Neg	
3293	4/22/13	M	14.0	IBANDA	Neg	
3294	4/22/13	M	32.0	BUGOYE	Neg	
3295	4/22/13	F	2.0	NDUGHUTU	Pos	
3296	4/22/13	F	29.0	MALIBA	Neg	
3297	4/22/13	M	2.0	KISANZI	Pos	
3298	4/22/13	F	39.0	KANYAN	Neg	
3299	4/22/13	M	54.0	MUBUKU	Neg	
3300	4/22/13	F	23.0	MALIBA	Pos	
3301	4/22/13	M	2.0	BUNYANGONI	Neg	
3302	4/22/13	F	0.6	NYAKARUNGU	Pos	
3303	4/22/13	F	28.0	IBANDA		Neg
3304	4/22/13	F	4.0	BUGOYE	Neg	
3305	4/22/13	F	20.0	MALIBA	Pos	
3306	4/22/13	F	60.0	IBANDA	Neg	
3307	4/22/13	F	3.0	BUNYANGONI	Pos	
3308	4/22/13	F	0.6	BUNYANGONI	Pos	
3309	4/22/13	F	38.0	MURAMBA	Pos	
3310	4/22/13	F	5.0	MALIBA	Pos	

3311	4/22/13	M	2.3	BUGOYE	Neg	
3312	4/22/13	F	1.0	KATOOKE	Neg	
3313	4/22/13	M	2.5	MALIBA	Neg	
3314	4/22/13	F	28.0	IBANDA	Pos	
3315	4/22/13	M	0.5	MURAMBA	Neg	
3316	4/22/13	M	8.0	KYARUGOMOKA	Pos	
3317	4/22/13	F	0.6	BUGOYE	Neg	
3318	4/22/13	F	20.0	BUGOYE	Neg	
3319	4/22/13	F	26.0	BUGOYE	Pos	
3320	4/23/13	F	30.0	MALIBA	Pos	Pos
3321	4/23/13	F	11.0	MURAMBA	Pos	
3322	4/23/13	F	35.0	KANYAN	Neg	
3323	4/23/13	F	35.0	KANYAN	Pos	
3324	4/23/13	F	19.0	IZINGA	Neg	
3325	4/23/13	M	1.1	IZINGA	Pos	
3326	4/23/13	F	21.0	KASANDERA	Neg	
3327	4/23/13	M	5.0	MURAMBA	Neg	
3328	4/23/13	M	72.0	IZINGA	Neg	
3329	4/23/13	M	5.0	NDUGHUTU	Pos	
3330	4/23/13	M	1.8	NDUGHUTU		Neg
3331	4/23/13	M	29.0	KANYAN	Pos	
3332	4/23/13	F	19.0	KISAMBA	Neg	
3333	4/23/13	M	2.5	MURAMBA	Pos	
3334	4/23/13	F	0.5	NDUGHUTU	Neg	
3335	4/23/13	F	50.0	MALIBA		Neg
3336	4/23/13	F	0.8	IZINGA	Neg	
3337	4/23/13	F	18.0	MAGHOMA	Pos	
3338	4/23/13	F	18.0	BUGOYE	Neg	
3339	4/24/13	F	20.0	IZINGA		Neg
3340	4/24/13	F	35.0	IZINGA	Neg	
3341	4/24/13	F	23.0	IBANDA	Neg	
3342	4/24/13	F	52.0	BUGOYE	Neg	
3343	4/24/13	F	62.0	NDUGHUTU	Neg	
3344	4/24/13	F	5.5	IBANDA	Neg	
3345	4/24/13	F	2.0	KAKINDO	Pos	
3346	4/24/13	F	28.0	KAKINDO	Neg	
3347	4/24/13	F	7.0	IHANI	Neg	
3348	4/24/13	F	30.0	MURAMBA	Neg	
3349	4/24/13	F	65.0	KISAMBA	Neg	
3350	4/24/13	F	21.0	KYARUGOMOKA	Neg	
3351	4/24/13	M	30.0	BUZIBWERA		Neg
3352	4/24/13	F	2.0	BUHUNGA	Neg	
3353	4/24/13	F	23.0	NDUGHUTU	Neg	

3354	4/24/13	F	24.0	MURAMBA	Neg	
3355	4/24/13	F	40.0	MURAMBA	Neg	
3356	4/24/13	F	13.0	MALIBA	Neg	
3357	4/24/13	F	0.5	RWAKING	Neg	
3358	4/24/13	M	9.0	MURAMBA	Neg	
3359	4/24/13	F	2.5	KATUMBA	Neg	
3360	4/25/13	F	46.0	RWAKING	Pos	
3361	4/25/13	M	1.8	KATUMBA	Neg	
3362	4/25/13	F	26.0	IZINGA	Neg	
3363	4/25/13	F	42.0	MURAMBA	Neg	
3364	4/25/13	F	24.0	MUKATI	Neg	
3365	4/25/13	F	42.0	KIKOKERA	Neg	
3366	4/25/13	M	6.0	KANYAN	Neg	
3367	4/25/13	M	1.3	IZINGA		Neg
3368	4/25/13	M	0.6	IBANDA	Neg	
3369	4/25/13	F	0.8	RWAKING	Neg	
3370	4/25/13	M	1.0	IZINGA	Pos	
3371	4/25/13	M	19.0	MALIBA	Neg	
3372	4/25/13	F	3.0	KATOOKE	Neg	
3373	4/25/13	M	4.0	KAKINDO	Neg	
3374	4/25/13	M	8.0	BUGOYE	Pos	
3375	4/25/13	F	4.0	KOHABUKRNENE	Neg	
3376	4/26/13	F	18.0	MUBUKU	Pos	
3377	4/26/13	M	10.0	MURAMBA	Pos	
3378	4/26/13	F	18.0	MAGHOMA	Pos	
3379	4/26/13	F	5.0	NDUGHUTU	Neg	
3380	4/26/13	F	2.8	BUNYANGONI	Pos	
3381	4/26/13	F	19.0	IZINGA	Pos	
3382	4/26/13	F	17.0	BUNYANGONI	Neg	
3383	4/29/13	F	22.0	IBANDA	Pos	
3384	4/29/13	M	25.0	RWAKING	Neg	
3385	4/29/13	M	0.2	KIKOKERA	Neg	
3386	4/29/13	M	16.0	MALIBA	Pos	
3387	4/29/13	F	30.0	BUGOYE	Neg	
3388	4/29/13	F	6.0	KANYAN	Pos	
3389	4/29/13	M	13.0	KASANDERA	Neg	
3390	4/29/13	F	10.0	KASANDERA	Neg	
3391	4/29/13	F	6.0	IBANDA	Neg	
3392	4/29/13	F	18.0	BUGOYE	Pos	
3393	4/29/13	F	9.0	MALIBA	Neg	
3394	4/29/13	F	34.0	KANYAN	Neg	
3395	4/29/13	F	20.0	BUGOYE	Neg	
3396	4/29/13	F	47.0	MALIBA	Neg	

3397	4/29/13	F	0.8	NYAKARUNGU	Neg	
3398	4/29/13	M	14.0	KANYAN	Pos	
3399	4/29/13	M	9.0	RWAKING	Pos	
3400	4/29/13	M	1.0	MALIBA	Neg	
3401	4/29/13	M	14.0	RWAKING	Pos	
3402	4/29/13	M	34.0	IZINGA	Pos	
3403	4/29/13	F	1.5	KANYAN		Neg
3404	4/29/13	F	12.0	KISANZI	Neg	
3405	4/29/13	F	6.0	MAGHOMA	Neg	
3406	4/29/13	F	3.0	BUNYANGONI	Neg	
3407	4/29/13	F	22.0	IZINGA	Pos	
3408	4/29/13	F	8.0	MURAMBA	Neg	
3409	4/29/13	F	45.0	BUNYANGONI	Neg	
3410	4/29/13	F	33.0	IZINGA	Neg	
3411	4/29/13	F	21.0	RWAKING	Neg	
3412	4/30/13	M	6.5	BUNYANGONI	Pos	
3413	4/30/13	F	2.0	BUNYANGONI	Pos	
3414	4/30/13	F	3.0	KANYAN	Neg	Neg
3415	4/30/13	M	0.8	BUNYANGONI	Neg	
3416	4/30/13	M	54.0	IZINGA	Neg	
3417	4/30/13	F	47.0	MURAMBA	Neg	
3418	4/30/13	M	50.0	MALIBA	Neg	
3419	4/30/13	F	22.0	KYARUGOMOKA	Neg	
3420	4/30/13	F	45.0	MALIBA	Neg	
3421	4/30/13	M	0.7	BUGOYE	Neg	
3422	4/30/13	M	11.0	BUZIBWERA	Neg	
3423	5/2/13	M	12.0	MURAMBA	Pos	
3424	5/2/13	F	38.0	IZINGA	Neg	
3425	5/2/13	F	70.0	IZINGA	Neg	
3426	5/2/13	F	0.3	RWAKING	Neg	
3427	5/2/13	F	16.0	MUBUKU	Pos	
3428	5/2/13	M	8.0	RWAKING	Pos	
3429	5/2/13	F	10.0	NDUGHUTU	Pos	
3430	5/2/13	F	17.0	MURAMBA	Neg	
3431	5/2/13	F	22.0	KIRONGO	Neg	
3432	5/2/13	M	0.7	RWAKING	Neg	
3433	5/2/13	F	40.0	KANYAN	Neg	
3434	5/2/13	M	4.0	KAKINDO	Neg	
3435	5/2/13	M	35.0	MURAMBA	Neg	
3436	5/2/13	F	41.0	BUGOYE	Neg	
3437	5/2/13	M	8.0	NDUGHUTU	Pos	
3438	5/2/13	M	4.0	IZINGA	Pos	
3439	5/2/13	F	20.0	BUNYANGONI	Neg	

3440	5/2/13	F	37.0	BUGOYE	Pos
3441	5/2/13	F	29.0	MAGHOMA	Neg
3442	5/2/13	F	1.2	KYANYA	Neg
3443	5/2/13	F	0.6	IZINGA	Neg
3444	5/2/13	M	14.0	MALIBA	Neg
3445	5/6/13	M	16.0	MURAMBA	Pos
3446	5/6/13	F	16.0	IHANI	Pos
3447	5/6/13	F	21.0	RWAKING	Neg
3448	5/6/13	F	1.0	BUGOYE	Neg
3449	5/6/13	F	3.0	MURAMBA	Pos
3450	5/6/13	M	5.0	RWAKING	Pos
3451	5/6/13	F	18.0	KASANDERA	Pos
3452	5/6/13	F	1.5	NDUGHUTU	Pos
3453	5/6/13	M	23.0	RWAKING	Pos
3454	5/7/13	M	21.0	BUZIBWERA	Neg
3455	5/7/13	M	33.0	BUGOYE	Pos
3456	5/7/13	F	1.7	NDUGHUTU	Neg
3457	5/7/13	F	20.0	NDUGHUTU	Neg
3458	5/7/13	F	4.0	RWAKING	Neg
3459	5/8/13	F	3.0	BUNYANGONI	Pos
3460	5/8/13	F	20.0	BUNYANGONI	Neg
3461	5/8/13	F	39.0	KANYAN	Neg
3462	5/8/13	M	14.0	KANYAN	Neg
3463	5/8/13	F	33.0	RWAKING	Neg
3464	5/8/13	M	10.0	NDUGHUTU	Neg
3465	5/8/13	F	34.0	NDUGHUTU	Neg
3466	5/8/13	F	0.5	NDUGHUTU	Neg
3467	5/8/13	F	14.0	IZINGA	Pos
3468	5/8/13	F	44.0	KIBIRIZI	Neg
3469	5/8/13	F	19.0	RWAKING	Neg
3470	5/8/13	M	1.1	RWAKING	Neg
3471	5/8/13	F	54.0	BUNYANGONI	Neg
3472	5/8/13	F	37.0	BUGOYE	Neg
3473	5/8/13	F	35.0	IBANDA	Neg
3474	5/8/13	M	47.0	NDUGHUTU	Pos
3475	5/8/13	F	50.0	KANYAN	Neg
3476	5/8/13	F	35.0	KIKOKERA	Neg
3477	5/8/13	F	18.0	IBANDA	Neg
3478	5/8/13	M	1.5	KATOOKE	Neg
3479	5/8/13	M	0.3	BUNYANGONI	Neg
3480	5/9/13	M	4.0	RWAKING	Pos
3481	5/9/13	F	15.0	RWAKING	Pos
3482	5/9/13	F	1.5	RWAKING	Neg

3483	5/9/13	M	50.0	RWAKING	Neg	
3484	5/9/13	F	9.0	MURAMBA	Neg	
3485	5/9/13	M	18.0	BUGOYE	Pos	
3486	5/9/13	M	27.0	NDUGHUTU	Neg	
3487	5/9/13	F	24.0	MURAMBA	Neg	
3488	5/9/13	M	35.0	KYAYABO	Neg	
3489	5/9/13	F	2.0	IZINGA	Neg	
3490	5/9/13	F	35.0	BUGOYE	Neg	
3491	5/9/13	M	18.0	NDUGHUTU	Neg	
3492	5/9/13	F	22.0	BUNYANGONI	Neg	
3493	5/9/13	M	1.3	KYANYA	Neg	
3494	5/9/13	F	0.5	IHANI	Neg	
3495	5/9/13	F	1.5	MWARU	Neg	
3496	5/9/13	F	30.0	IZINGA	Neg	
3497	5/9/13	M	13.0	NDUGHUTU	Neg	
3498	5/9/13	F	8.0	BUGOYE	Pos	
3499	5/9/13	F	3.0	BUGOYE	Neg	
3500	5/9/13	F	38.0	BUNYANGONI	Neg	
3501	5/10/13	M	14.0	RWAKING	Neg	
3502	5/10/13	F	3.8	BUNYANGONI	Neg	
3503	5/10/13	F	22.0	BUNYANGONI	Pos	
3504	5/10/13	F	1.3	MURAMBA	Neg	
3505	5/10/13	F	35.0	MURAMBA	Pos	
3506	5/10/13	M	13.0	NDUGHUTU	Neg	
3507	5/10/13	M	0.3	IZINGA	Pos	
3508	5/10/13	F	60.0	BUHUNGA	Neg	
3509	5/10/13	F	22.0	ZERIYA	Neg	
3510	5/10/13	F	30.0	RWAKING	Pos	
3511	5/10/13	F	8.0	NDUGHUTU	Pos	
3512	5/10/13	M	0.6	BUGOYE	Neg	
3513	5/10/13	F	1.2	.	Neg	
3514	5/10/13	F	31.0	KATOOKE	Neg	
3515	5/10/13	M	4.0	KEMIKORO	Pos	
3516	5/10/13	F	5.0	KEMIKORO	Pos	
3517	5/10/13	F	16.0	MURAMBA	Pos	
3518	5/13/13	F	22.0	BUNYANGONI	Pos	
3519	5/13/13	F	18.0	BUGOYE	Neg	
3520	5/13/13	M	19.0	KANYAN		Pos
3521	5/13/13	M	2.5	BUNYANGONI	Neg	
3522	5/13/13	M	4.5	RWAKING	Pos	
3523	5/13/13	F	17.0	IBANDA	Neg	
3524	5/13/13	F	15.0	BUGOYE	Neg	
3525	5/13/13	F	0.5	MURAMBA	Neg	

3526	5/13/13	F	4.5	BUGOYE	Neg	
3527	5/13/13	F	0.4	KIRONGO	Neg	
3528	5/13/13	M	17.0	RWAKING	Neg	
3529	5/13/13	F	29.0	IBANDA	Neg	
3530	5/13/13	F	0.4	MURAMBA	Neg	
3531	5/13/13	M	13.0	KANYAN	Neg	
3532	5/13/13	F	25.0	IZINGA	Neg	
3533	5/13/13	M	1.8	KISANZI	Pos	
3534	5/13/13	F	22.0	KISANZI	Neg	
3535	5/13/13	F	20.0	KANYAN	Neg	
3536	5/13/13	F	27.0	KIHINDI	Neg	
3537	5/13/13	M	3.0	NDUGHUTU	Neg	
3538	5/13/13	F	17.0	KISANGA	Neg	
3539	5/14/13	F	43.0	MURAMBA	Pos	
3540	5/14/13	F	10.0	MURAMBA	Neg	
3541	5/14/13	F	38.0	IZINGA	Neg	
3542	5/14/13	F	6.0	BUNYANGONI	Neg	
3543	5/14/13	F	50.0	BUNYANGONI	Neg	
3544	5/14/13	F	0.8	IZINGA	Neg	
3545	5/14/13	F	3.0	BUNYANGONI	Pos	Pos
3546	5/14/13	F	80.0	BUGOYE	Neg	
3547	5/14/13	M	13.0	IZINGA	Pos	
3548	5/14/13	M	5.0	MURAMBA	Pos	
3549	5/14/13	M	0.4	NDUGHUTU	Pos	
3550	5/14/13	M	6.0	RWAKING	Pos	
3551	5/14/13	F	0.4	NDUGHUTU	Neg	
3552	5/14/13	F	28.0	IZINGA	Neg	
3553	5/14/13	F	26.0	BUGOYE	Neg	
3554	5/14/13	F	24.0	BUGOYE	Neg	
3555	5/15/13	F	23.0	NDUGHUTU	Neg	
3556	5/15/13	F	46.0	RWAKING		Neg
3557	5/15/13	M	12.0	MURAMBA	Neg	
3558	5/15/13	F	39.0	BUGOYE	Neg	
3559	5/15/13	F	33.0	KIBIRIZI	Neg	
3560	5/15/13	M	1.0	IZINGA	Neg	
3561	5/15/13	M	3.0	IZINGA	Neg	
3562	5/15/13	M	20.0	BUGOYE	Neg	
3563	5/15/13	F	4.0	NDUGHUTU	Neg	
3564	5/15/13	M	10.0	.	Neg	
3565	5/15/13	M	3.0	KYARUGOMOKA	Neg	
3566	5/15/13	F	75.0	MURAMBA	Neg	
3567	5/15/13	F	22.0	KYANYA	Neg	
3568	5/15/13	F	2.7	IZINGA	Neg	

3569	5/15/13	F	30.0	.	Neg	
3570	5/15/13	M	26.0	BUGOYE	Neg	
3571	5/15/13	F	17.0	KANYAN	Neg	
3572	5/15/13	F	18.0	BUGOYE	Neg	
3573	5/15/13	M	28.0	KASANDERA	Neg	
3574	5/15/13	F	60.0	BUGOYE	Neg	
3575	5/15/13	F	20.0	KATOOKE	Neg	
3576	5/15/13	F	27.0	KASANDERA	Neg	
3577	5/15/13	F	3.0	MUHAMBO	Neg	
3578	5/16/13	F	21.0	KYARUGOMOKA	Neg	
3579	5/16/13	M	1.3	KASASE	Neg	
3580	5/16/13	F	27.0	KATOOKE	Pos	Pos
3581	5/16/13	F	16.0	BUNYANGONI		Neg
3582	5/16/13	F	12.0	MURAMBA	Neg	
3583	5/16/13	F	20.0	BUGOYE	Neg	
3584	5/16/13	F	28.0	MALIBA	Neg	
3585	5/16/13	F	75.0	MURAMBA	Neg	
3586	5/16/13	F	23.0	MUBUKU	Neg	
3587	5/16/13	F	37.0	MAGHOMA	Neg	
3588	5/16/13	F	0.4	KATUMBA	Neg	
3589	5/16/13	M	0.5	NDUGHUTU	Neg	
3590	5/16/13	F	21.0	MURAMBA	Neg	
3591	5/16/13	M	5.0	BUGOYE	Pos	
3592	5/16/13	F	3.0	BUGOYE	Neg	
3593	5/16/13	F	0.5	IZINGA	Neg	
3594	5/16/13	F	24.0	BUGOYE	Neg	
3595	5/16/13	F	28.0	ISULE	Neg	Pos
3596	5/17/13	F	48.0	KABUKERA	Pos	
3597	5/17/13	M	12.0	RWAKING	Pos	
3598	5/20/13	M	48.0	BUGOYE	Neg	
3599	5/20/13	F	22.0	KANYAN	Neg	
3600	5/20/13	F	40.0	MURAMBA	Neg	
3601	5/20/13	F	46.0	MURAMBA	Neg	
3602	5/20/13	M	18.0	KANYAN	Neg	
3603	5/20/13	M	38.0	MALIBA	Neg	
3604	5/20/13	M	1.1	MALIBA	Pos	
3605	5/20/13	F	24.0	BUGOYE	Neg	
3606	5/20/13	M	23.0	KIKOKERA	Neg	
3607	5/20/13	F	25.0	RWAKING	Neg	
3608	5/20/13	F	22.0	MURAMBA	Neg	
3609	5/20/13	M	17.0	IHANI	Neg	
3610	5/20/13	F	26.0	KYARUGOMOKA	Neg	
3611	5/20/13	F	32.0	NYAKALENGIJO	Pos	

3612	5/20/13	F	57.0	KANYAN	Neg
3613	5/20/13	F	44.0	KANYAN	Neg
3614	5/20/13	F	1.3	KIRULI	Neg
3615	5/20/13	F	30.0	IBANDA	Neg
3616	5/20/13	F	43.0	IBANDA	Neg
3617	5/20/13	M	3.8	IBANDA	Neg
3618	5/20/13	M	21.0	BUGOYE	Neg
3619	5/21/13	M	52.0	MURAMBA	Neg
3620	5/21/13	M	0.8	MALIBA	Neg
3621	5/21/13	F	19.0	NDUGHUTU	Pos
3622	5/21/13	F	4.0	MURAMBA	Neg
3623	5/21/13	F	15.0	BUGOYE	Neg
3624	5/21/13	F	0.9	KIRULI	Neg
3625	5/21/13	F	35.0	KIRULI	Neg
3626	5/21/13	F	2.0	KIRULI	Neg
3627	5/21/13	F	15.0	NDUGHUTU	Pos
3628	5/21/13	M	4.0	BUNYANGONI	Neg
3629	5/21/13	F	26.0	BUNYANGONI	Neg
3630	5/22/13	F	80.0	IBANDA	Neg
3631	5/22/13	M	14.0	IBANDA	Neg
3632	5/22/13	M	1.2	KIKOKERA	Neg
3633	5/22/13	M	40.0	RWAKING	Neg
3634	5/22/13	M	52.0	MURAMBA	Neg
3635	5/22/13	F	80.0	BUGOYE	Neg
3636	5/22/13	F	18.0	IBANDA	Neg
3637	5/22/13	F	36.0	BULYANDEKE	Neg
3638	5/22/13	F	1.5	NYAKARUNGU	Neg
3639	5/22/13	M	1.0	NYAKARUNGU	Neg
3640	5/22/13	F	20.0	KATOOKE	Neg
3641	5/22/13	F	7.0	KANYAN	Neg
3642	5/22/13	F	24.0	MURAMBA	Pos
3643	5/22/13	M	0.5	BUGOYE	Neg
3644	5/22/13	F	0.8	KANYAN	Neg
3645	5/22/13	F	21.0	NYABISUSI	Neg
3646	5/22/13	M	3.3	KISANZI	Pos
3647	5/22/13	F	23.0	KISANZI	Neg
3648	5/22/13	F	45.0	RWAKING	Neg
3649	5/22/13	F	16.0	MURAMBA	Neg
3650	5/22/13	F	64.0	NDUGHUTU	Neg
3651	5/23/13	F	39.0	MUHAMBO	Neg
3652	5/23/13	F	29.0	BUNYANGONI	Neg
3653	5/23/13	F	0.1	MUHAMBO	Neg
3654	5/23/13	F	0.4	IZINGA	Neg

3655	5/23/13	M	3.0	IZINGA	Neg
3656	5/23/13	F	0.7	KIKOKERA	Neg
3657	5/23/13	F	26.0	BUNYANGONI	Neg
3658	5/23/13	F	2.1	NDUGHUTU	Neg
3659	5/23/13	F	23.0	NDUGHUTU	Neg
3660	5/23/13	M	0.8	RWAKING	Pos
3661	5/23/13	M	3.0	NDUGHUTU	Neg
3662	5/23/13	F	30.0	RWAKING	Neg
3663	5/23/13	F	55.0	BUGOYE	Neg
3664	5/23/13	F	0.5	KYARUGOMOKA	Pos
3665	5/24/13	F	1.3	MURAMBA	Neg
3666	5/24/13	F	45.0	KABUKERA	Neg
3667	5/24/13	F	5.0	MURAMBA	Neg
3668	5/24/13	F	13.0	IZINGA	Neg
3669	5/24/13	M	30.0	KATOOKE	Neg
3670	5/24/13	M	10.0	IZINGA	Neg
3671	5/24/13	F	7.0	BUNYANGONI	Pos
3672	5/24/13	F	25.0	BUNYANGONI	Neg
3673	5/24/13	F	28.0	NDUGHUTU	Pos
3674	5/24/13	F	39.0	KYANYA	Neg
3675	5/24/13	M	14.0	IZINGA	Neg
3676	5/27/13	F	22.0	IBANDA	Pos
3677	5/27/13	F	30.0	KIKOKERA	Neg
3678	5/27/13	F	2.3	KATOOKE	Neg
3679	5/27/13	F	28.0	IZINGA	Neg
3680	5/27/13	F	61.0	NYAKARUNGU	Neg
3681	5/27/13	F	20.0	RWAKING	Pos
3682	5/27/13	F	36.0	MAGHOMA	Neg
3683	5/27/13	F	20.0	MALIBA	Pos
3684	5/27/13	F	0.3	BUNYANGONI	Pos
3685	5/27/13	F	19.0	.	Neg
3686	5/27/13	F	20.0	RWAKING	Neg
3687	5/27/13	F	24.0	NDUGHUTU	Neg
3688	5/27/13	F	23.0	NDUGHUTU	Pos
3689	5/27/13	F	4.5	BUNYANGONI	Neg
3690	5/27/13	F	1.5	BUNYANGONI	Neg
3691	5/27/13	F	45.0	MALIBA	Pos
3692	5/27/13	F	39.0	IBANDA	Neg
3693	5/27/13	F	18.0	NDUGHUTU	Neg
3694	5/27/13	F	18.0	NDUGHUTU	Neg
3695	5/27/13	F	28.0	MURAMBA	Neg
3696	5/27/13	F	50.0	MAGHOMA	Neg
3697	5/27/13	M	24.0	RWAKING	Neg

3698	5/27/13	M	54.0	KANYAN	Pos
3699	5/27/13	F	18.0	IBANDA	Neg
3700	5/27/13	F	0.5	IZINGA	Neg
3701	5/27/13	F	20.0	NDUGHUTU	Neg
3702	5/27/13	M	7.0	RWAKING	Neg
3703	5/27/13	F	3.0	BUGOYE	Pos
3704	5/27/13	F	2.5	KYANYA	Pos
3705	5/27/13	F	4.5	KANYAN	Neg
3706	5/27/13	M	1.0	MURAMBA	Neg
3707	5/27/13	M	0.7	MALIBA	Neg
3708	5/27/13	F	27.0	IZINGA	Neg
3709	5/28/13	M	27.0	BUGOYE	Pos
3710	5/28/13	M	20.0	MURAMBA	Pos
3711	5/28/13	M	5.0	IZINGA	Pos
3712	5/28/13	M	9.0	KIKOKERA	Pos
3713	5/28/13	F	60.0	MURAMBA	Neg
3714	5/28/13	F	15.0	BUGOYE	Neg
3715	5/28/13	M	65.0	MURAMBA	Neg
3716	5/28/13	F	4.0	MURAMBA	Neg
3717	5/28/13	F	2.5	KIBOTA	Pos
3718	5/28/13	M	12.0	RWAKING	Pos
3719	5/28/13	F	46.0	MURAMBA	Neg
3720	5/28/13	F	12.0	MURAMBA	Pos
3721	5/28/13	F	40.0	MURAMBA	Neg
3722	5/28/13	F	14.0	BUGOYE	Neg
3723	5/29/13	M	17.0	MURAMBA	Neg
3724	5/29/13	F	3.5	BUGOYE	Pos
3725	5/29/13	F	7.0	IHANI	Pos
3726	5/29/13	F	40.0	MURAMBA	Neg
3727	5/29/13	F	52.0	.	Neg
3728	5/29/13	F	6.0	KANYAN	Pos
3729	5/29/13	F	30.0	BUZIBWERA	Neg
3730	5/29/13	F	5.0	NDUGHUTU	Pos
3731	5/29/13	F	5.0	BUGOYE	Pos
3732	5/29/13	F	8.0	BUGOYE	Pos
3733	5/29/13	F	38.0	BUGOYE	Neg
3734	5/29/13	F	5.0	BUGOYE	Pos
3735	5/30/13	F	38.0	NYAKARUNGU	Neg
3736	5/30/13	F	23.0	IHANI	Pos
3737	5/30/13	F	8.0	NYAKARUNGU	Pos
3738	5/30/13	F	5.5	MALIBA	Neg
3739	5/30/13	M	0.9	MURAMBA	Neg
3740	5/30/13	M	18.0	NDUGHUTU	Neg

3741	5/30/13	F	6.0	IZINGA	Pos
3742	5/31/13	F	50.0	MURAMBA	Neg
3743	5/31/13	F	46.0	IZINGA	Neg
3744	5/31/13	F	16.0	KIKOKERA	Neg
3745	5/31/13	M	42.0	BUVATO	Neg
3746	5/31/13	F	50.0	MURAMBA	Neg
3747	5/31/13	M	22.0	NYAKARUNGU	Neg
3748	5/31/13	F	25.0	KATOOKE	Neg
3749	5/31/13	F	2.0	MALIBA	Pos
3750	5/31/13	F	5.0	BUGOYE	Neg
3751	5/31/13	M	1.5	MALIBA	Pos
3752	5/31/13	F	20.0	MALIBA	Pos
3753	5/31/13	F	21.0	MUBUKU	Neg
3754	5/31/13	M	.	MALIBA	Neg
3755	5/31/13	F	40.0	MURAMBA	Neg
3756	5/31/13	F	70.0	KIRONGO	Neg
3757	5/31/13	F	14.0	KATUMBA	Neg
3758	6/4/13	F	21.0	KISANZI	Neg
3759	6/4/13	F	50.0	MURAMBA	Neg
3760	6/4/13	M	8.0	MURAMBA	Neg
3761	6/4/13	F	28.0	BUGOYE	Neg
3762	6/4/13	F	15.0	BUGOYE	Neg
3763	6/4/13	M	5.0	NDUGHUTU	Pos
3764	6/4/13	F	60.0	MAGHOMA	Neg
3765	6/4/13	M	62.0	IZINGA	Neg
3766	6/4/13	F	16.0	BUGOYE	Neg
3767	6/4/13	M	0.5	KYANYA	Neg
3768	6/4/13	F	10.0	KYANYA	Neg
3769	6/4/13	F	2.5	KYANYA	Neg
3770	6/4/13	F	30.0	IBANDA	Neg
3771	6/4/13	M	4.0	BUNYANGONI	Neg
3772	6/4/13	F	.	.	Neg
3773	6/4/13	F	32.0	MURAMBA	Pos
3774	6/4/13	F	30.0	.	Neg
3775	6/4/13	F	5.0	BUGOYE	Neg
3776	6/4/13	M	0.8	KATUMBA	Neg
3777	6/4/13	F	47.0	NDUGHUTU	Neg
3778	6/4/13	F	5.0	MUBUKU	Neg
3779	6/4/13	F	37.0	MURAMBA	Neg
3780	6/4/13	F	0.4	NDUGHUTU	Neg
3781	6/4/13	F	5.0	KATOOKE	Neg
3782	6/4/13	F	39.0	NDUGHUTU	Neg
3783	6/4/13	M	73.0	BUNYANGONI	Neg

3784	6/4/13	F	44.0	MALIBA	Neg	
3785	6/4/13	M	5.0	NDUGHUTU	Pos	
3786	6/4/13	F	38.0	KIRONGO	Neg	
3787	6/4/13	M	22.0	BUGOYE	Neg	
3788	6/5/13	M	8.0	MALIBA		Neg
3789	6/5/13	F	25.0	MURAMBA	Neg	
3790	6/5/13	F	1.3	RWAKING	Neg	
3791	6/5/13	F	19.0	NDUGHUTU	Neg	
3792	6/5/13	F	3.0	BUZIBWERA	Pos	
3793	6/5/13	M	56.0	MALIBA	Neg	
3794	6/5/13	F	16.0	KANYAN	Neg	
3795	6/5/13	M	3.0	BUZIBWERA	Neg	
3796	6/5/13	M	35.0	BUGOYE	Neg	
3797	6/5/13	M	54.0	MURAMBA	Neg	
3798	6/5/13	M	50.0	MALIBA	Neg	
3799	6/5/13	M	3.2	KAKINDO	Pos	
3800	6/5/13	F	13.0	BUGOYE	Pos	
3801	6/5/13	F	19.0	KAKINDO	Neg	
3802	6/5/13	F	28.0	IHANI	Pos	
3803	6/5/13	M	45.0	KIRULI	Neg	
3804	6/5/13	M	3.0	NYAKARUNGU	Pos	
3805	6/5/13	F	30.0	IHANI	Neg	
3806	6/5/13	M	23.0	BUNYANGONI	Neg	
3807	6/5/13	F	52.0	KYARUGOMOKA	Neg	
3808	6/5/13	F	18.0	MUBUKU	Neg	
3809	6/5/13	F	24.0	BUNYANGONI	Pos	
3810	6/5/13	F	18.0	MURAMBA	Pos	
3811	6/5/13	F	19.0	BUGOYE	Neg	
3812	6/5/13	F	70.0	MURAMBA	Pos	Neg
3813	6/5/13	M	1.7	MURAMBA	Neg	
3814	6/5/13	F	30.0	KASESE	Neg	
3815	6/5/13	F	32.0	MALIBA	Pos	
3816	6/5/13	F	0.8	IBANDA	Neg	
3817	6/5/13	F	2.3	IBANDA	Neg	
3818	6/5/13	F	40.0	BUGOYE	Neg	
3819	6/5/13	F	35.0	MALIBA	Neg	
3820	6/5/13	F	28.0	KATUMBA	Neg	
3821	6/5/13	F	35.0	MALIBA	Pos	
3822	6/5/13	F	5.0	KIBIRIZI	Neg	
3823	6/5/13	F	5.0	MALIBA	Neg	
3824	6/5/13	F	42.0	MALIBA	Neg	
3825	6/6/13	F	45.0	KANYAN	Neg	
3826	6/6/13	F	40.0	MURAMBA	Neg	

3827	6/6/13	F	20.0	KAGANDO	Neg	
3828	6/6/13	M	15.0	MUBUKU	Pos	
3829	6/6/13	M	4.0	KYANYA	Neg	
3830	6/6/13	F	42.0	KYANYA	Neg	
3831	6/6/13	F	2.0	KYARUGOMOKA	Neg	
3832	6/6/13	F	15.0	MURAMBA	Neg	
3833	6/6/13	M	4.0	MUHAMBO	Neg	
3834	6/6/13	F	21.0	MURAMBA	Pos	
3835	6/6/13	F	1.3	KISAMBA	Pos	
3836	6/6/13	M	4.0	KISANZI	Pos	
3837	6/6/13	F	40.0	BUGOYE	Pos	
3838	6/6/13	F	28.0	BUNYANGONI	Neg	
3839	6/6/13	M	5.0	BUNYANGONI	Neg	
3840	6/6/13	F	2.0	IBANDA	Neg	
3841	6/6/13	F	30.0	IZINGA	Pos	
3842	6/6/13	F	0.8	RWAKING	Neg	
3843	6/6/13	F	20.0	RWAKING	Pos	
3844	6/7/13	M	30.0	MUHAMBO	Neg	
3845	6/7/13	F	35.0	BUNYANGONI		Neg
3846	6/7/13	F	4.0	MALIBA	Neg	
3847	6/7/13	F	15.0	NYAKARUNGU	Pos	
3848	6/7/13	F	19.0	KANYAN	Neg	
3849	6/7/13	M	52.0	RWAKING	Pos	
3850	6/7/13	F	25.0	RWAKING	Neg	
3851	6/7/13	F	38.0	KAKINDO	Neg	
3852	6/7/13	F	0.7	RWAKING	Neg	
3853	6/7/13	F	70.0	MURAMBA	Pos	
3854	6/7/13	F	1.3	KISAMBA	Neg	
3855	6/7/13	F	30.0	MUBUKU	Neg	
3856	6/7/13	M	2.5	MUBUKU	Neg	
3857	6/10/13	F	24.0	KANYAN	Neg	
3858	6/10/13	F	9.0	NDUGHUTU	Pos	
3859	6/10/13	F	4.0	KANYAN	Neg	Neg
3860	6/10/13	F	16.0	MALIBA	Neg	
3861	6/10/13	F	38.0	KANYAN	Pos	
3862	6/10/13	F	12.0	MALIBA	Pos	
3863	6/10/13	M	56.0	IBANDA	Neg	Neg
3864	6/10/13	F	18.0	IZINGA	Pos	
3865	6/10/13	F	8.0	MALIBA	Pos	
3866	6/10/13	F	24.0	KATOOKE	Neg	
3867	6/10/13	F	56.0	NDUGHUTU	Neg	
3868	6/10/13	F	6.0	IBANDA	Neg	
3869	6/10/13	F	25.0	MALIBA	Neg	

3870	6/10/13	F	11.0	KATOOKE	Neg	
3871	6/10/13	F	36.0	KATOOKE	Neg	
3872	6/11/13	F	2.0	NYAKARUNGU		Pos
3873	6/11/13	F	25.0	MALIBA	Pos	
3874	6/11/13	F	8.0	KANYAN	Pos	
3875	6/11/13	F	30.0	MURAMBA	Neg	
3876	6/11/13	M	65.0	KATOOKE	Pos	
3877	6/11/13	F	2.0	KIRONGO	Neg	
3878	6/11/13	F	56.0	KATOOKE	Neg	
3879	6/11/13	M	13.0	IBANDA	Neg	
3880	6/11/13	F	29.0	BUZIBWERA	Neg	
3881	6/11/13	M	8.0	BUGOYE	Pos	Pos
3882	6/11/13	F	15.0	BUGOYE	Pos	
3883	6/11/13	M	25.0	KISANGA	Neg	
3884	6/11/13	F	28.0	BUGOYE	Neg	
3885	6/11/13	F	35.0	BUGOYE	Neg	
3886	6/11/13	F	1.0	BUNYANGONI	Neg	
3887	6/11/13	F	20.0	NDUGHUTU	Neg	
3888	6/11/13	M	3.0	KANYAN	Neg	Neg
3889	6/11/13	F	17.0	MALIBA	Neg	
3890	6/11/13	M	2.0	BUGOYE	Neg	
3891	6/11/13	F	12.0	KANYAN	Pos	
3892	6/11/13	F	6.0	BUZIBWERA	Pos	
3893	6/11/13	M	12.0	BUNYANGONI	Pos	
3894	6/11/13	M	11.0	KIRONGO	Pos	
3895	6/12/13	F	8.0	KANYAN		
3896	6/12/13	M	2.8	KIRONGO	Neg	
3897	6/12/13	F	12.0	BUGOYE	Neg	
3898	6/12/13	F	35.0	RWAKING	Neg	
3899	6/12/13	F	36.0	.	Neg	
3900	6/12/13	F	54.0	BUNYANGONI	Neg	
3901	6/12/13	M	10.0	BUNYANGONI	Pos	
3902	6/12/13	M	3.8	BUNYANGONI	Neg	
3903	6/12/13	F	32.0	BUGOYE	Neg	
3904	6/12/13	F	3.0	KIRONGO	Neg	
3905	6/12/13	M	13.0	NDUGHUTU	Pos	
3906	6/12/13	M	1.3	BUNYANGONI	Neg	
3907	6/12/13	M	11.0	KYARUGOMOKA	Neg	
3908	6/12/13	F	3.5	IBANDA	Neg	
3909	6/12/13	F	6.0	MALIBA	Pos	
3910	6/12/13	F	6.0	MALIBA	Pos	
3911	6/12/13	F	39.0	MAGHOMA	Neg	
3912	6/12/13	F	2.0	NDUGHUTU	Neg	

3913	6/12/13	F	19.0	NDUGHUTU	Pos	
3914	6/12/13	F	0.5	MAGHOMA	Neg	
3915	6/12/13	F	40.0	MURAMBA	Neg	
3916	6/12/13	F	52.0	MUHAMBO	Neg	
3917	6/12/13	F	15.0	IBANDA	Neg	
3918	6/12/13	F	18.0	NYAKARUNGU	Neg	
3919	6/13/13	F	1.4	BUGOYE	Neg	
3920	6/13/13	M	2.5	IHANI	Neg	
3921	6/13/13	F	34.0	NYAKABUGHA	Neg	
3922	6/13/13	F	52.0	MALIBA	Neg	
3923	6/13/13	F	45.0	NDUGHUTU	Neg	
3924	6/13/13	M	10.0	KYARUGOMOKA	Pos	
3925	6/13/13	F	70.0	KATOOKE	Neg	
3926	6/13/13	F	4.0	NDUGHUTU	Neg	
3927	6/13/13	M	8.0	BUNYANGONI	Pos	
3928	6/13/13	F	40.0	KATOOKE	Pos	
3929	6/13/13	M	1.3	BUZIBWERA	Neg	
3930	6/13/13	F	35.0	KATUMBA	Pos	
3931	6/13/13	F	44.0	MUHAMBO	Neg	
3932	6/13/13	F	28.0	IZINGA	Neg	
3933	6/13/13	F	16.0	BUGOYE	Neg	
3934	6/13/13	F	2.0	IHANI	Pos	
3935	6/14/13	F	35.0	MALIBA	Neg	
3936	6/14/13	F	40.0	MAGHOMA	Neg	
3937	6/14/13	F	16.0	BUGOYE	Pos	
3938	6/14/13	M	1.3	BUZIBWERA	Pos	
3939	6/14/13	F	22.0	BUGOYE		Neg
3940	6/14/13	F	16.0	BUGOYE	Neg	
3941	6/14/13	M	3.0	NDUGHUTU	Pos	
3942	6/14/13	M	37.0	BUGOYE	Neg	
3943	6/14/13	F	18.0	MALIBA	Pos	
3944	6/14/13	M	0.4	IZINGA	Neg	
3945	6/14/13	M	87.0	KISANGA	Pos	
3946	6/14/13	M	14.0	MURAMBA	Neg	
3947	6/14/13	M	11.0	RWAKING	Pos	
3948	6/14/13	F	21.0	KIHINDI	Neg	
3949	6/14/13	M	1.5	NDUGHUTU	Neg	
3950	6/14/13	F	4.0	KYANYA	Pos	
3951	6/14/13	F	40.0	NYAKABUGHA	Neg	
3952	6/14/13	M	9.0	BUZIBWERA	Pos	
3953	6/14/13	F	46.0	KIRONGO	Neg	
3954	6/14/13	F	20.0	MURAMBA	Neg	
3955	6/14/13	F	4.0	KIRONGO	Pos	

3956	6/14/13	M	1.4	KIRONGO	Pos	
3957	6/14/13	F	24.0	KIKOKERA	Pos	
3958	6/14/13	M	3.0	RWAKING	Neg	
3959	6/14/13	M	24.0	RWAKING	Neg	
3960	6/17/13	F	20.0	KIHINDI	Neg	
3961	6/17/13	F	4.0	KANYAN	Pos	
3962	6/17/13	M	0.3	MUHAMBO	Neg	
3963	6/17/13	F	42.0	KANYAN	Pos	
3964	6/17/13	F	50.0	BUNYANGONI	Pos	
3965	6/17/13	F	4.0	RWAKING	Pos	
3966	6/17/13	M	10.0	KANYAN	Neg	
3967	6/17/13	F	20.0	MURAMBA	Pos	
3968	6/17/13	M	30.0	IBANDA	Neg	
3969	6/17/13	F	28.0	NYAKARUNGU	Neg	
3970	6/18/13	F	35.0	BUGOYE	Neg	
3971	6/18/13	M	10.0	BUGOYE		Pos
3972	6/18/13	F	10.0	BUGOYE	Pos	
3973	6/18/13	F	4.0	KISANZI	Pos	
3974	6/18/13	F	30.0	NDUGHUTU	Neg	
3975	6/18/13	F	1.1	NYAKABUGHA	Neg	
3976	6/18/13	M	13.0	MURAMBA	Neg	
3977	6/18/13	F	1.5	MURAMBA	Neg	
3978	6/18/13	M	9.0	NDUGHUTU	Pos	
3979	6/18/13	M	63.0	NDUGHUTU	Neg	
3980	6/18/13	F	22.0	BUNYANGONI	Pos	
3981	6/18/13	F	2.0	RWAKING	Neg	
3982	6/18/13	F	14.0	NDUGHUTU	Pos	
3983	6/18/13	F	10.0	IBANDA	Neg	
3984	6/18/13	F	23.0	MUBUKU	Neg	
3985	6/18/13	F	45.0	IHANI	Neg	
3986	6/18/13	M	1.5	NDUGHUTU	Pos	
3987	6/18/13	F	21.0	KEMIHOKO	Pos	
3988	6/18/13	M	2.0	MURAMBA	Pos	
3989	6/18/13	F	21.0	KYANYA	Neg	
3990	6/18/13	M	10.0	RASHIDI	Pos	
3991	6/18/13	F	6.0	KATOOKE	Neg	
3992	6/19/13	M	4.5	IBANDA	Pos	
3993	6/19/13	M	65.0	KIRONGO	Neg	
3994	6/19/13	F	18.0	BUGOYE	Neg	
3995	6/19/13	F	47.0	MURAMBA	Neg	
3996	6/19/13	F	18.0	KATOOKE	Neg	
3997	6/19/13	F	16.0	NDUGHUTU	Neg	
3998	6/19/13	F	2.6	KANYAN	Neg	

3999	6/19/13	F	68.0	KIRONGO	Neg	
4000	6/19/13	F	1.2	RWAKING	Neg	
4001	6/19/13	F	10.0	MURAMBA	Pos	
4002	6/19/13	F	1.4	KISANZI	Pos	
4003	6/19/13	M	26.0	MALIBA	Neg	
4004	6/19/13	M	2.0	MALIBA	Pos	
4005	6/19/13	F	48.0	HIMA	Neg	
4006	6/19/13	F	22.0	MALIBA	Neg	
4007	6/19/13	M	0.9	MURAMBA	Neg	
4008	6/19/13	F	18.0	KIGANDO	Neg	
4009	6/19/13	F	24.0	BUGOYE	Neg	
4010	6/19/13	M	23.0	KIKOKERA	Pos	
4011	6/20/13	F	18.0	BUGOYE	Neg	
4012	6/20/13	M	0.8	MALIBA	Neg	
4013	6/20/13	F	45.0	BUGOYE		Pos
4014	6/20/13	F	1.5	BUGOYE	Neg	
4015	6/20/13	M	3.5	IBANDA	Neg	
4016	6/20/13	F	15.0	BUGOYE	Pos	
4017	6/20/13	F	29.0	KIKOKERA	Pos	
4018	6/20/13	F	1.4	KAKINDO	Pos	
4019	6/20/13	F	28.0	KIKOKERA	Neg	
4020	6/20/13	F	0.5	BUNYANGONI	Neg	
4021	6/20/13	F	23.0	BUNYANGONI	Neg	
4022	6/20/13	M	4.0	KIKOKERA	Pos	
4023	6/20/13	F	2.5	KIKOKERA	Pos	
4024	6/20/13	M	6.5	KAKINDO	Pos	
4025	6/20/13	F	9.0	KAKINDO	Pos	
4026	6/20/13	F	2.5	IZINGA	Neg	
4027	6/20/13	M	0.5	MURAMBA	Neg	
4028	6/20/13	F	46.0	BUZIBWERA	Neg	
4029	6/20/13	F	18.0	MALIBA	Neg	
4030	6/20/13	F	3.5	MUBUKU	Pos	
4031	6/20/13	F	19.0	MALIBA	Neg	
4032	6/20/13	F	2.5	NDUGHUTU	Neg	
4033	6/20/13	M	11.0	RWAKING	Pos	
4034	6/20/13	F	3.0	KIKOKERA	Pos	
4035	6/20/13	F	18.0	IZINGA	Neg	
4036	6/20/13	F	6.0	IHANI	Pos	
4037	6/20/13	F	14.0	KISOJO	Pos	
4038	6/20/13	F	38.0	KISANZI	Neg	
4039	6/22/13	F	10.0	MURAMBA		Pos
4040	6/22/13	F	17.0	KIKOKERA	Neg	
4041	6/22/13	F	13.0	BUGOYE	Pos	

4042	6/22/13	F	18.0	KANYAN	Neg
4043	6/22/13	F	12.0	BUGOYE	Pos
4044	6/22/13	F	50.0	IZINGA	Neg
4045	6/22/13	F	12.0	MALIBA	Pos
4046	6/22/13	F	0.8	IZINGA	Neg
4047	6/22/13	F	11.0	IZINGA	Pos
4048	6/22/13	M	2.5	IBANDA	Neg
4049	6/22/13	F	19.0	KANYAN	Neg
4050	6/22/13	F	37.0	BUNYANGONI	Pos
4051	6/22/13	M	1.0	NDUGHUTU	Neg
4052	6/22/13	F	26.0	MALIBA	Neg
4053	6/22/13	M	17.0	MURAMBA	Pos
4054	6/22/13	F	32.0	BULINDUGURU	Neg
4055	6/22/13	F	23.0	IZINGA	Neg
4056	6/22/13	M	26.0	MUHAMBO	Neg
4057	6/22/13	F	50.0	IZINGA	Neg
4058	6/22/13	F	0.9	MURAMBA	Neg
4059	6/22/13	F	9.0	BUZIBWERA	Pos
4060	6/22/13	F	5.5	RWAKING	Pos
4061	6/22/13	M	3.0	BUGOYE	Pos
4062	6/22/13	F	80.0	KISANZI	Pos
4063	6/23/13	F	17.0	MAGHOMA	Neg
4064	6/23/13	F	0.8	BUNYANGONI	Neg
4065	6/23/13	F	24.0	ISULE	Neg
4066	6/23/13	M	6.0	RWAKING	Pos
4067	6/23/13	F	42.0	KAKINDO	Pos
4068	6/23/13	M	15.0	KYARUGOMOKA	Pos
4069	6/23/13	F	40.0	KYARUGOMOKA	Pos
4070	6/23/13	F	27.0	MALIBA	Neg
4071	6/23/13	M	33.0	MALIBA	Pos
4072	6/23/13	F	26.0	MALIBA	Neg
4073	6/23/13	F	63.0	BUGOYE	Neg
4074	6/23/13	F	13.0	BUGOYE	Pos
4075	6/23/13	M	10.0	MALIBA	Neg
4076	6/23/13	F	58.0	NYAKARUNGU	Neg
4077	6/23/13	F	1.9	KYANYA	Neg
4078	6/23/13	F	1.5	KYANYA	Pos
4079	6/23/13	M	38.0	BUHUNGA	Neg
4080	6/23/13	M	55.0	IZINGA	Pos
4081	6/23/13	M	58.0	MURAMBA	Neg
4082	6/23/13	F	50.0	MURAMBA	Neg
4083	6/23/13	F	4.0	MAGHOMA	Pos
4084	6/23/13	F	27.0	KIHINDI	Neg

4085	6/23/13	F	9.0	MURAMBA	Neg	
4086	6/23/13	F	25.0	KIKOKERA	Neg	
4087	6/23/13	M	17.0	BUGOYE	Neg	
4088	6/23/13	F	30.0	BUZIBWERA	Neg	
4089	6/23/13	F	20.0	MURAMBA	Neg	
4090	6/23/13	M	2.0	BUZIBWERA	Pos	
4091	6/23/13	M	1.5	MURAMBA	Pos	
4092	6/23/13	M	1.0	KIRITERE	Neg	
4093	6/23/13	F	34.0	.	Neg	
4094	6/23/13	F	16.0	MURAMBA	Neg	
4095	6/23/13	F	1.3	KABUGHO	Neg	
4096	6/25/13	F	14.0	KAKINDO	Neg	
4097	6/25/13	M	37.0	NYANGONGE	Neg	
4098	6/25/13	M	28.0	MAGHOMA	Neg	
4099	6/25/13	M	21.0	MUBUKU	Neg	
4100	6/26/13	F	16.0	KIKOKERA	Pos	
4101	6/26/13	M	62.0	IBANDA	Neg	
4102	6/26/13	M	12.0	BUGOYE	Neg	
4103	6/26/13	F	3.0	RWAKING	Pos	
4104	6/26/13	F	30.0	MURAMBA	Neg	
4105	6/26/13	M	16.0	NYAKALENGIJO	Pos	
4106	6/26/13	F	17.0	KANYAN	Neg	
4107	6/26/13	F	13.0	BUNYANGONI	Neg	
4108	6/26/13	F	1.6	RWAKING	Pos	
4109	6/26/13	M	4.0	KISANGA	Pos	
4110	6/26/13	M	6.0	KISANGA	Pos	
4111	6/26/13	F	13.0	IZINGA	Pos	
4112	6/26/13	F	13.0	KIRONGO	Pos	
4113	6/26/13	F	14.0	BUNYANGONI	Pos	
4114	6/26/13	F	35.0	RWAKING	Neg	
4115	6/26/13	F	8.0	BUGOYE	Neg	
4116	6/26/13	M	1.7	KISANZI	Pos	
4117	6/26/13	F	35.0	BUGOYE	Neg	
4118	6/26/13	F	3.5	BUNYANGONI	Neg	
4119	6/26/13	F	1.8	NDUGHUTU	Neg	
4120	6/26/13	F	23.0	RWAKING	Neg	
4121	6/26/13	F	53.0	MALIBA	Neg	
4122	6/26/13	M	1.0	MALIBA	Pos	
4123	6/26/13	M	1.5	MALIBA	Pos	
4124	6/26/13	F	30.0	KISENDERA	Neg	
4125	6/27/13	F	35.0	NDUGHUTU	Neg	Neg
4126	6/27/13	F	1.1	MURAMBA	Neg	
4127	6/27/13	F	3.0	RWAKING	Pos	

4128	6/27/13	M	10.0	BUNYANGONI	Neg
4129	6/27/13	F	26.0	MURAMBA	Neg
4130	6/27/13	F	20.0	RWAKING	Neg
4131	6/27/13	F	2.0	MALIBA	Pos
4132	6/27/13	F	5.0	MURAMBA	Neg
4133	6/27/13	F	7.0	KIKOKERA	Pos
4134	6/27/13	M	40.0	IZINGA	Neg
4135	6/27/13	M	39.0	MURAMBA	Neg
4136	6/27/13	F	55.0	MALIBA	Pos
4137	6/27/13	F	30.0	NYAKARUNGU	Pos
4138	6/27/13	F	1.9	NYAKARUNGU	Pos
4139	6/27/13	F	4.5	NDUGHUTU	Neg
4140	6/27/13	F	32.0	BUHUNGA	Neg
4141	6/27/13	F	18.0	MALIBA	Pos
4142	6/27/13	F	20.0	MALIBA	Pos
4143	6/27/13	M	15.0	NDUGHUTU	Neg
4144	6/27/13	F	0.6	IZINGA	Neg
4145	6/27/13	F	65.0	NDUGHUTU	Neg
4146	6/27/13	F	20.0	NDUGHUTU	Neg
4147	6/27/13	F	35.0	MURAMBA	Pos
4148	6/27/13	F	33.0	KANYAN	Neg
4149	6/27/13	F	9.5	NDUGHUTU	Neg
4150	6/27/13	M	0.8	KIRONGO	Neg
4151	6/27/13	F	5.0	MALIBA	Pos
4152	6/28/13	F	9.0	KAKINDO	Pos
4153	6/28/13	F	17.0	IZINGA	Pos
4154	6/28/13	M	1.5	BUGOYE	Neg
4155	6/28/13	M	5.0	KANYAN	Neg
4156	6/28/13	F	2.5	BUNYANGONI	Neg
4157	6/28/13	F	38.0	SINONYIRI	Neg
4158	6/28/13	F	3.0	BUNYANGONI	Pos
4159	6/28/13	F	8.0	BUNYANGONI	Pos
4160	6/28/13	M	12.0	BUGOYE	Neg
4161	6/28/13	M	16.0	MURAMBA	Neg
4162	6/28/13	F	11.0	BUNYANGONI	Pos
4163	6/28/13	F	13.0	NYAKALENGIJO	Pos
4164	6/28/13	F	34.0	NYAKALENGIJO	Pos
4165	6/28/13	F	25.0	IHANI	Pos
4166	6/28/13	M	35.0	IHANI	Neg
4167	6/28/13	F	67.0	KEMIHOKO	Neg
4168	6/28/13	F	27.0	NDUGHUTU	Neg
4169	6/28/13	M	3.0	RWAKING	Pos
4170	6/28/13	M	18.0	MURAMBA	Neg

4171	6/28/13	F	23.0	MUZAHURA	Neg
4172	7/1/13	F	0.5	RWAKING	Neg
4173	7/1/13	F	2.5	NYAKABUGHA	Pos
4174	7/1/13	F	23.0	MALIBA	Pos
4175	7/1/13	F	11.0	RWAKING	Pos
4176	7/1/13	M	4.0	RWAKING	Pos
4177	7/1/13	M	18.0	RWAKING	Pos
4178	7/1/13	F	1.3	IZINGA	Neg
4179	7/1/13	F	17.0	IBANDA	Neg
4180	7/1/13	F	1.5	KAKINDO	Pos
4181	7/1/13	M	10.0	MURAMBA	Pos
4182	7/1/13	F	50.0	MAGHOMA	Neg
4183	7/1/13	F	18.0	BUGOYE	Neg
4184	7/1/13	M	2.0	BUGOYE	Neg
4185	7/1/13	F	22.0	KATOOKE	Neg
4186	7/1/13	F	20.0	RWAKING	Pos
4187	7/1/13	F	37.0	KISANGA	Neg
4188	7/1/13	M	13.0	KYANYA	Pos
4189	7/1/13	M	2.1	KANYAN	Neg
4190	7/1/13	F	3.0	MALIBA	Pos
4191	7/1/13	M	8.0	MUKATI	Neg
4192	7/1/13	F	27.0	KANYAN	Neg
4193	7/1/13	F	21.0	KYARUGOMOKA	Pos
4194	7/1/13	M	3.0	MALIBA	Neg
4195	7/1/13	F	14.0	MAGHOMA	Neg
4196	7/1/13	F	15.0	MURAMBA	Neg
4197	7/1/13	F	18.0	MURAMBA	Neg
4198	7/1/13	M	0.0	KATOOKE	Pos
4199	7/1/13	M	2.5	MALIBA	Pos
4200	7/1/13	M	2.4	KAKINDO	Pos
4201	7/1/13	M	4.0	KAKINDO	Pos
4202	7/1/13	M	32.0	NDUGHUTU	Pos
4203	7/1/13	F	25.0	KAKINDO	Pos
4204	7/1/13	F	25.0	KISOJO	Neg
4205	7/2/13	F	38.0	BUGOYE	Pos
4206	7/2/13	M	9.0	IZINGA	Pos
4207	7/2/13	F	70.0	IZINGA	Pos
4208	7/2/13	M	1.3	MURAMBA	Neg
4209	7/2/13	M	28.0	KYARUGOMOKA	Neg
4210	7/2/13	M	7.0	IBANDA	Neg
4211	7/2/13	M	13.0	RWAKING	Pos
4212	7/2/13	F	5.0	KAKINDO	Pos
4213	7/2/13	F	50.0	MURAMBA	Neg

4214	7/2/13	F	9.0	BUNYANGONI	Neg
4215	7/2/13	F	22.0	BUNYANGONI	Neg
4216	7/2/13	F	9.0	KISANZI	Pos
4217	7/2/13	F	49.0	KISANZI	Neg
4218	7/2/13	F	19.0	NDUGHUTU	Pos
4219	7/2/13	M	12.0	NDUGHUTU	Pos
4220	7/2/13	F	33.0	RWAKING	Neg
4221	7/2/13	F	3.0	BUNYANGONI	Pos
4222	7/2/13	F	6.0	BUNYANGONI	Pos
4223	7/2/13	F	18.0	KATUMBA	Neg
4224	7/2/13	F	0.8	IZINGA	Neg
4225	7/2/13	F	33.0	RWAKING	Neg
4226	7/2/13	F	46.0	RWAKING	Neg
4227	7/2/13	F	40.0	IZINGA	Pos
4228	7/2/13	F	16.0	MURAMBA	Pos
4229	7/2/13	F	0.6	RWAKING	Pos
4230	7/2/13	F	15.0	IHANI	Pos
4231	7/2/13	M	4.0	BUGOYE	Pos
4232	7/2/13	F	1.0	KATUMBA	Pos
4233	7/2/13	F	20.0	BUZIBWERA	Pos
4234	7/2/13	F	8.0	BUZIBWERA	Pos
4235	7/2/13	F	7.0	BUZIBWERA	Pos
4236	7/2/13	F	20.0	KISAMBA	Neg
4237	7/2/13	F	30.0	KAKINDO	Pos
4238	7/3/13	F	28.0	KISANGA	Pos
4239	7/3/13	F	18.0	KIRONGO	Pos
4240	7/3/13	F	42.0	IZINGA	Pos
4241	7/3/13	F	12.0	NDUGHUTU	Pos
4242	7/3/13	F	6.0	BUGOYE	Neg
4243	7/3/13	M	5.0	MURAMBA	Neg
4244	7/3/13	M	13.0	RWAKING	Pos
4245	7/3/13	M	40.0	MURAMBA	Neg
4246	7/3/13	F	23.0	MURAMBA	Pos
4247	7/3/13	F	30.0	KIKOKERA	Neg
4248	7/3/13	M	3.0	BUGOYE	Neg
4249	7/3/13	F	22.0	BUZIBWERA	Pos
4250	7/3/13	M	6.0	NDUGHUTU	Pos
4251	7/4/13	M	6.0	MURAMBA	Pos
4252	7/4/13	F	12.0	MALIBA	Pos
4253	7/4/13	F	11.0	MALIBA	Pos
4254	7/4/13	F	12.0	KIKOKERA	Pos
4255	7/4/13	F	26.0	KATOOKE	Neg
4256	7/4/13	F	22.0	IZINGA	Pos

4257	7/4/13	F	18.0	IZINGA	Neg	
4258	7/4/13	M	7.0	RWAKING	Neg	
4259	7/4/13	M	3.0	KANYAN	Neg	
4260	7/4/13	F	3.0	KYARUGOMOKA	Pos	
4261	7/4/13	M	4.0	NDUGHUTU	Neg	
4262	7/4/13	M	52.0	MALIBA	Pos	
4263	7/4/13	F	11.0	KANYAN	Pos	
4264	7/4/13	F	0.1	NDUGHUTU	Neg	
4265	7/4/13	F	13.0	BUGOYE	Neg	
4266	7/4/13	F	17.0	IBANDA	Neg	
4267	7/4/13	F	30.0	KYANYA	Pos	
4268	7/4/13	M	7.0	KISOJO	Pos	
4269	7/4/13	M	12.0	KISOJO	Pos	
4270	7/4/13	M	5.0	KISOJO	Neg	
4271	7/4/13	F	45.0	MALIBA	Neg	
4272	7/4/13	F	34.0	BUNYANGONI	Pos	
4273	7/4/13	F	1.2	BUNYANGONI	Neg	
4274	7/4/13	F	9.0	IZINGA	Pos	
4275	7/4/13	M	5.0	IZINGA	Pos	
4276	7/4/13	F	5.0	KIRONGO	Pos	
4277	7/4/13	F	7.0	KIRONGO	Pos	
4278	7/4/13	M	2.0	MURAMBA	Pos	
4279	7/4/13	F	15.0	BUZIBWERA	Pos	
4280	7/4/13	F	28.0	KYANYA	Neg	
4281	7/5/13	F	20.0	KANYAN	Neg	
4282	7/5/13	M	1.3	IZINGA		Pos
4283	7/5/13	M	7.0	IHANI	Neg	
4284	7/5/13	F	42.0	RWAKING	Neg	
4285	7/5/13	F	56.0	MUBUKU	Pos	
4286	7/5/13	F	40.0	RWAKING	Pos	
4287	7/5/13	M	14.0	IBANDA	Pos	
4288	7/5/13	F	37.0	KYARUGOMOKA	Pos	
4289	7/5/13	F	20.0	RWAKING	Neg	
4290	7/5/13	F	18.0	MALIBA	Pos	
4291	7/5/13	F	25.0	KYARUGOMOKA	Pos	
4292	7/5/13	F	50.0	MURAMBA	Neg	
4293	7/5/13	F	25.0	BUGOYE	Neg	
4294	7/5/13	F	1.5	BUGOYE	Neg	
4295	7/5/13	M	40.0	KATUMBA	Pos	
4296	7/5/13	F	1.5	NYAKARUNGU	Neg	
4297	7/5/13	M	39.0	NYAKARUNGU	Pos	
4298	7/5/13	M	2.5	RWAKING	Pos	
4299	7/5/13	F	40.0	KYARUGOMOKA	Neg	

4300	7/5/13	F	32.0	BUGOYE	Neg	
4301	7/5/13	M	5.0	MALIBA	Pos	
4302	7/5/13	M	13.0	MALIBA	Pos	
4303	7/5/13	F	22.0	KYARUGOMOKA	Pos	
4304	7/5/13	M	2.5	NDUGHUTU	Neg	
4305	7/5/13	F	25.0	NDUGHUTU	Neg	
4306	7/5/13	M	27.0	RWAKING	Pos	
4307	7/8/13	F	18.0	IBANDA	Pos	
4308	7/8/13	M	35.0	BUGOYE	Neg	
4309	7/8/13	M	66.0	KISENDERA	Neg	
4310	7/8/13	F	0.5	MALIBA	Pos	
4311	7/8/13	F	35.0	MALIBA	Pos	
4312	7/8/13	F	27.0	BUNYANGONI	Neg	
4313	7/8/13	F	25.0	KISAMBA	Neg	
4314	7/8/13	F	80.0	MUHAMBO	Neg	
4315	7/8/13	F	1.5	RWAKING	Pos	
4316	7/8/13	F	23.0	RWAKING	Pos	
4317	7/8/13	F	3.0	KIKOKERA	Neg	
4318	7/8/13	F	32.0	BUNYANGONI	Neg	
4319	7/8/13	F	0.3	KANYAN	Neg	Neg
4320	7/8/13	F	11.0	RWAKING	Pos	
4321	7/8/13	M	26.0	KIKOKERA	Pos	
4322	7/8/13	F	38.0	MALIBA	Pos	
4323	7/8/13	M	19.0	RWAKING	Pos	
4324	7/8/13	F	14.0	BUZIBWERA	Pos	
4325	7/8/13	F	16.0	BUGOYE	Neg	
4326	7/8/13	M	12.0	BUZIBWERA	Neg	
4327	7/8/13	M	14.0	BUNYANGONI	Pos	
4328	7/8/13	M	1.0	MALIBA	Neg	
4329	7/8/13	F	3.0	NYAMBUKU	Pos	
4330	7/8/13	M	30.0	MALIBA	Neg	
4331	7/8/13	F	1.5	KYANYA	Pos	
4332	7/8/13	F	6.0	KIKOKERA	Pos	
4333	7/8/13	F	9.0	KAKINDO	Neg	
4334	7/8/13	M	41.0	MUBUKU	Neg	
4335	7/8/13	M	20.0	MBUMURO	Pos	
4336	7/8/13	M	1.5	MALIBA	Neg	
4337	7/8/13	M	3.5	RWAKING	Neg	
4338	7/8/13	F	9.0	KISANGA	Pos	
4339	7/8/13	M	3.0	KISANGA	Pos	
4340	7/8/13	F	15.0	KISANGA	Neg	
4341	7/8/13	M	7.0	KISANGA	Pos	
4342	7/8/13	M	1.7	NDUGHUTU	Pos	

4343	7/8/13	F	17.0	KIRULI		Pos
4344	7/8/13	F	17.0	KISANZI	Pos	
4345	7/8/13	F	17.0	NYANGONGE	Neg	
4346	7/8/13	F	19.0	KANYAN	Neg	
4347	7/8/13	F	19.0	KIRULI	Neg	
4348	7/8/13	M	0.6	KATOOKE	Neg	
4349	7/8/13	M	1.5	KIRULI	Pos	
4350	7/8/13	M	0.6	MUHAMBO	Neg	
4351	7/8/13	F	1.5	NYANGONGE	Neg	
4352	7/8/13	F	48.0	BUTABUYA	Neg	
4353	7/8/13	F	18.0	MALIBA	Pos	
4354	7/9/13	M	36.0	KIKOKERA	Neg	
4355	7/9/13	F	2.0	MALIBA	Neg	
4356	7/9/13	M	22.0	IZINGA	Neg	
4357	7/9/13	M	5.0	RWAKING	Pos	
4358	7/9/13	F	0.4	RWAKING	Pos	
4359	7/9/13	F	23.0	RWAKING	Pos	
4360	7/9/13	F	23.0	RWAKING	Neg	
4361	7/9/13	F	38.0	BUGOYE	Neg	
4362	7/9/13	F	15.0	NDUGHUTU	Neg	
4363	7/9/13	F	3.5	MALIBA	Neg	
4364	7/9/13	F	29.0	MALIBA	Pos	
4365	7/9/13	F	13.0	RWAKING	Pos	
4366	7/9/13	M	0.5	RWAKING	Neg	
4367	7/9/13	F	68.0	RWAKING	Pos	
4368	7/9/13	M	1.3	MALIBA	Pos	
4369	7/9/13	F	5.5	BUNYANGONI	Neg	
4370	7/9/13	M	2.5	BUNYANGONI	Pos	
4371	7/9/13	M	25.0	KISANGA	Neg	
4372	7/9/13	M	14.0	RWAKING	Pos	
4373	7/9/13	M	16.0	KISANZI	Pos	
4374	7/9/13	M	11.0	KIBOTA	Neg	
4375	7/9/13	M	3.0	MURAMBA	Pos	
4376	7/9/13	F	15.0	IZINGA	Neg	
4377	7/9/13	F	6.0	.	Pos	
4378	7/9/13	F	9.0	BUGOYE	Neg	
4379	7/9/13	F	50.0	KANYAN	Pos	
4380	7/9/13	M	70.0	IBANDA	Pos	
4381	7/9/13	M	74.0	KYANYA	Neg	
4382	7/9/13	F	7.0	KIKOKERA	Pos	
4383	7/9/13	F	8.0	KIKOKERA	Pos	
4384	7/9/13	F	15.0	BUGOYE	Neg	
4385	7/9/13	F	1.3	MALIBA	Pos	

4386	7/9/13	F	18.0	MURAMBA	Pos
4387	7/9/13	F	28.0	NDUGHUTU	Neg
4388	7/9/13	F	46.0	MURAMBA	Neg
4389	7/9/13	F	39.0	MALIBA	Pos
4390	7/9/13	F	65.0	IBANDA	Neg
4391	7/9/13	M	21.0	BUZIBWERA	Pos
4392	7/9/13	F	24.0	BUGOYE	Neg
4393	7/9/13	F	30.0	IBANDA	Neg
4394	7/9/13	M	5.0	NDUGHUTU	Pos
4395	7/9/13	M	29.0	NDUGHUTU	Neg
4396	7/9/13	M	13.0	MURAMBA	Neg
4397	7/9/13	M	28.0	MUHAMBO	Neg
4398	7/11/13	M	3.0	MALIBA	Pos
4399	7/11/13	M	30.0	MALIBA	Neg
4400	7/11/13	F	28.0	NYAKARUNGU	Pos
4401	7/11/13	F	1.5	NYAKARUNGU	Pos
4402	7/11/13	F	40.0	BULYANDEKE	Neg
4403	7/11/13	M	46.0	RWAKING	Neg
4404	7/11/13	F	22.0	BULYANDEKE	Pos
4405	7/11/13	F	32.0	RWAKING	Neg
4406	7/11/13	F	7.0	BULYANDEKE	Pos
4407	7/11/13	F	1.8	RWAKING	Pos
4408	7/11/13	F	20.0	KYANYA	Neg
4409	7/11/13	M	7.0	RWAKING	Pos
4410	7/11/13	F	11.0	NDUGHUTU	Neg
4411	7/12/13	F	17.0	BUGOYE	Neg
4412	7/12/13	M	23.0	KANYAN	Neg
4413	7/12/13	F	0.8	RWAKING	Neg
4414	7/12/13	F	25.0	KIKOKERA	Pos
4415	7/12/13	M	9.0	RWAKING	Pos
4416	7/12/13	M	1.3	KIRULI	Pos
4417	7/12/13	F	20.0	RWAKING	Neg
4418	7/12/13	F	17.0	KYALYANGA	Pos
4419	7/15/13	F	26.0	KANYAN	Pos
4420	7/15/13	F	41.0	KIKOKERA	Pos
4421	7/15/13	F	10.0	KANYAN	Pos
4422	7/15/13	M	6.5	BULYANDEKE	Pos
4423	7/15/13	F	58.0	KIBIRIZI	Neg
4424	7/15/13	F	1.5	KANYAN	Pos
4425	7/15/13	M	10.0	IZINGA	Pos
4426	7/15/13	F	5.0	KAKINDO	Neg
4427	7/15/13	F	8.0	KAKINDO	Pos
4428	7/15/13	F	11.0	KAKINDO	Neg

4429	7/15/13	M	47.0	KAKINDO	Neg
4430	7/15/13	M	13.0	KAKINDO	Neg
4431	7/15/13	F	43.0	KEMIHOKO	Neg
4432	7/15/13	F	38.0	KYANYA	Neg
4433	7/15/13	M	3.3	MURAMBA	Pos
4434	7/15/13	F	2.5	MALIBA	Pos
4435	7/15/13	M	23.0	BUGOYE	Neg
4436	7/15/13	F	45.0	KIBOTA	Pos
4437	7/15/13	M	5.0	BUGOYE	Pos
4438	7/15/13	F	54.0	BUGOYE	Neg
4439	7/15/13	F	5.5	BUGOYE	Pos
4440	7/15/13	M	30.0	MALIBA	Pos
4441	7/15/13	F	3.0	MALIBA	Pos
4442	7/15/13	F	22.0	KANYAN	Pos
4443	7/15/13	M	40.0	BUGOYE	Neg
4444	7/15/13	F	3.0	BUGOYE	Pos
4445	7/15/13	F	30.0	KISANZI	Neg
4446	7/15/13	F	29.0	IBANDA	Neg
4447	7/15/13	F	21.0	MALIBA	Pos
4448	7/15/13	M	3.0	MURAMBA	Pos
4449	7/15/13	M	7.0	NDUGHUTU	Pos
4450	7/15/13	F	14.0	NDUGHUTU	Pos
4451	7/15/13	M	10.0	NDUGHUTU	Pos
4452	7/15/13	M	3.5	IZINGA	Pos
4453	7/15/13	M	1.7	MALIBA	Neg
4454	7/15/13	F	51.0	MALIBA	Neg
4455	7/15/13	F	40.0	IZINGA	Neg
4456	7/15/13	F	6.0	RWAKING	Pos
4457	7/15/13	F	24.0	RWAKING	Pos
4458	7/15/13	F	25.0	NDUGHUTU	Neg
4459	7/15/13	F	1.3	KANYAN	Pos
4460	7/15/13	M	5.5	KANYAN	Neg
4461	7/15/13	F	18.0	MALIBA	Pos
4462	7/15/13	M	24.0	RWAKING	Neg
4463	7/15/13	M	19.0	IBANDA	Neg
4464	7/15/13	F	18.0	IZINGA	Neg
4465	7/15/13	F	4.0	BUGOYE	Neg
4466	7/15/13	F	4.0	KYANYA	Neg
4467	7/15/13	F	6.0	BUGOYE	Pos
4468	7/15/13	M	5.0	IZINGA	Pos
4469	7/15/13	F	12.0	IZINGA	Pos
4470	7/15/13	M	60.0	KISOJO	Pos
4471	7/15/13	F	4.0	KISOJO	Pos

4472	7/15/13	F	0.1	KANYAN	Neg
4473	7/15/13	M	12.0	NDUGHUTU	Pos
4474	7/15/13	F	28.0	MUHAMBO	Neg
4475	7/15/13	F	4.0	BIKONE	Pos
4476	7/16/13	M	7.0	NYAKALENGIJO	Pos
4477	7/16/13	M	12.0	NYAKALENGIJO	Pos
4478	7/16/13	M	2.0	BUZIBWERA	Pos
4479	7/16/13	M	19.0	BUNYANGONI	Neg
4480	7/16/13	F	0.7	BUGOYE	Neg
4481	7/16/13	M	4.0	IZINGA	Pos
4482	7/16/13	M	21.0	BUGOYE	Neg
4483	7/16/13	M	11.0	IBANDA	Neg
4484	7/16/13	M	12.0	IBANDA	Neg
4485	7/16/13	M	50.0	IBANDA	Neg
4486	7/16/13	F	17.0	BUGOYE	Neg
4487	7/16/13	F	0.6	BUNYANGONI	Neg
4488	7/16/13	M	1.0	MURAMBA	Pos
4489	7/16/13	M	60.0	BUNYANGONI	Neg
4490	7/16/13	M	18.0	KANYAN	Pos
4491	7/16/13	F	22.0	KAKINDO	Neg
4492	7/16/13	F	32.0	KANYAN	Pos
4493	7/16/13	M	7.0	KANYAN	Neg
4494	7/16/13	F	18.0	BUGOYE	Pos
4495	7/16/13	F	39.0	KAKINDO	Neg
4496	7/16/13	M	0.5	RWAKING	Pos
4497	7/16/13	F	20.0	RWAKING	Neg
4498	7/16/13	F	23.0	KAKINDO	Pos
4499	7/16/13	M	10.0	KYANYA	Neg
4500	7/16/13	F	18.0	IZINGA	Pos
4501	7/16/13	F	18.0	BUGOYE	Neg
4502	7/16/13	M	0.8	RWAKING	Pos
4503	7/16/13	F	6.0	BUGOYE	Neg
4504	7/16/13	F	40.0	KIKOKERA	Neg
4505	7/16/13	F	6.0	BUGOYE	Pos
4506	7/16/13	F	22.0	BUHUNGA	Neg
4507	7/16/13	M	3.0	IZINGA	Neg
4508	7/16/13	M	10.0	MALIBA	Pos
4509	7/16/13	M	72.0	BUNYANGONI	Neg
4510	7/17/13	F	1.5	KANYAN	Pos
4511	7/17/13	M	6.0	KANYAN	Neg
4512	7/17/13	M	3.5	MURAMBA	Pos
4513	7/17/13	F	4.5	KANYAN	Pos
4514	7/17/13	M	6.0	KANYAN	Pos

4515	7/17/13	F	32.0	KANYAN	Neg
4516	7/17/13	F	30.0	BUGOYE	Neg
4517	7/17/13	F	30.0	KIKOKERA	Neg
4518	7/17/13	M	2.0	ISENGERA	Neg
4519	7/17/13	F	10.0	IZINGA	Neg
4520	7/17/13	F	38.0	MALIBA	Pos
4521	7/17/13	F	28.0	KIKOKERA	Pos
4522	7/17/13	F	5.5	BUNYANGONI	Pos
4523	7/17/13	F	53.0	NDUGHUTU	Pos
4524	7/17/13	F	22.0	IBANDA	Pos
4525	7/17/13	F	24.0	IBANDA	Pos
4526	7/17/13	F	5.0	KAKINDO	Pos
4527	7/17/13	M	7.0	KAKINDO	Pos
4528	7/17/13	F	18.0	IBANDA	Pos
4529	7/17/13	F	19.0	NDUGHUTU	Pos
4530	7/17/13	M	3.0	BUNYANGONI	Neg
4531	7/17/13	M	2.0	NDUGHUTU	Neg
4532	7/17/13	M	3.0	BUGOYE	Neg
4533	7/17/13	M	6.0	KAKINDO	Neg
4534	7/17/13	F	5.0	IHANI	Neg
4535	7/17/13	F	24.0	KAKINDO	Neg
4536	7/17/13	F	21.0	KYANYA	Neg
4537	7/17/13	F	61.0	IBANDA	Pos
4538	7/17/13	F	20.0	MALIBA	Pos
4539	7/17/13	M	9.0	MURAMBA	Neg
4540	7/17/13	F	18.0	IHANI	Neg
4541	7/17/13	F	17.0	KIGANDO	Pos
4542	7/17/13	F	18.0	IZINGA	Pos
4543	7/17/13	M	12.0	KATUMBA	Pos
4544	7/17/13	F	32.0	KAKINDO	Neg
4545	7/18/13	F	21.0	BUGOYE	Pos
4546	7/18/13	M	43.0	IBANDA	Neg
4547	7/18/13	M	27.0	MALIBA	Neg
4548	7/18/13	F	30.0	IBANDA	Neg
4549	7/18/13	F	19.0	KANYAN	Pos
4550	7/18/13	M	4.0	NDUGHUTU	Pos
4551	7/18/13	F	11.0	IZINGA	Neg
4552	7/18/13	F	29.0	MALIBA	Pos
4553	7/18/13	M	5.0	NDUGHUTU	Pos
4554	7/18/13	F	1.6	KANYAN	Pos
4555	7/18/13	M	45.0	KISANGA	Pos
4556	7/18/13	F	50.0	BUGOYE	Neg
4557	7/18/13	F	11.0	NDUGHUTU	Neg

4558	7/18/13	F	25.0	IZINGA	Pos	
4559	7/18/13	M	51.0	IZINGA	Neg	
4560	7/18/13	F	21.0	KYANYA	Neg	
4561	7/18/13	F	5.0	NDUGHUTU	Pos	
4562	7/18/13	M	9.0	KANYAN	Pos	
4563	7/18/13	F	17.0	KANYAN	Pos	
4564	7/18/13	F	6.0	MURAMBA	Pos	
4565	7/18/13	F	55.0	KISANGA	Pos	
4566	7/18/13	F	28.0	BUNYANGONI	Pos	
4567	7/18/13	F	1.5	BUHUNGA	Neg	
4568	7/18/13	M	3.5	MURAMBA	Pos	
4569	7/18/13	M	8.0	KANYAN	Pos	
4570	7/18/13	F	4.0	KANYAN	Pos	
4571	7/18/13	F	5.0	IZINGA	Pos	
4572	7/18/13	M	45.0	KIRULI	Neg	
4573	7/18/13	F	1.4	MALIBA	Pos	
4574	7/18/13	M	0.5	KANYAN	Pos	
4575	7/18/13	M	18.0	KIHINDI	Pos	
4576	7/19/13	F	9.0	KANYAN	Pos	
4577	7/19/13	F	36.0	IBANDA	Neg	
4578	7/19/13	F	5.0	KANYAN	Pos	
4579	7/19/13	M	1.3	NDUGHUTU	Neg	Neg
4580	7/22/13	F	24.0	BUNYANGONI		Pos
4581	7/22/13	M	14.0	MURAMBA		Pos
4582	7/22/13	F	22.0	KIKOKERA		Pos
4583	7/22/13	F	30.0	KIKOKERA		Neg
4584	7/22/13	M	27.0	KIKOKERA		Neg
4585	7/22/13	F	9.0	MALIBA		Pos
4586	7/22/13	F	27.0	KIKOKERA		Pos
4587	7/22/13	F	60.0	IZINGA		Pos
4588	7/22/13	F	3.8	KIKOKERA		Neg
4589	7/22/13	F	28.0	KIKOKERA		Neg
4590	7/22/13	M	17.0	KIKOKERA		Pos
4591	7/22/13	F	17.0	BUGOYE		Pos
4592	7/22/13	F	26.0	MURAMBA		Neg
4593	7/22/13	F	57.0	KIKOKERA		Neg
4594	7/22/13	F	38.0	IHANI		Neg
4595	7/22/13	F	70.0	IHANI		Neg
4596	7/22/13	F	27.0	NDUGHUTU		Neg
4597	7/22/13	F	4.0	RWAKING		Pos
4598	7/22/13	F	16.0	RWAKING		Pos
4599	7/22/13	M	10.0	KAKINDO		Neg
4600	7/22/13	F	40.0	KANYAN		Neg

4601	7/22/13	M	0.4	BUNYANGONI	Pos
4602	7/22/13	F	41.0	KANYAN	Neg
4603	7/22/13	F	5.0	NDUGHUTU	Neg
4604	7/22/13	M	3.0	.	Pos
4605	7/22/13	M	70.0	KIBOTA	Pos
4606	7/22/13	F	4.0	MALIBA	Pos
4607	7/22/13	M	30.0	IHANI	Neg
4608	7/22/13	M	60.0	RWAKING	Neg
4609	7/22/13	F	3.0	NDUGHUTU	Pos
4610	7/22/13	F	23.0	NDUGHUTU	Neg
4611	7/22/13	F	13.0	KYARUGOMOKA	Pos
4612	7/22/13	F	1.2	IBANDA	Neg
4613	7/22/13	F	37.0	IBANDA	Neg
4614	7/22/13	M	5.0	MURAMBA	Pos
4615	7/22/13	M	14.0	IZINGA	Neg
4616	7/22/13	M	14.0	NDUGHUTU	Pos
4617	7/22/13	F	40.0	MALIBA	Neg
4618	7/22/13	F	20.0	IBANDA	Neg
4619	7/22/13	F	10.0	BUNYANGONI	Pos
4620	7/22/13	F	8.5	IZINGA	Pos
4621	7/22/13	F	46.0	IZINGA	Neg
4622	7/22/13	F	4.0	KIBIRIZI	Neg
4623	7/22/13	M	3.0	MUKATI	Pos
4624	7/22/13	F	9.0	IZINGA	Pos
4625	7/22/13	F	19.0	RWAKING	Neg
4626	7/22/13	F	9.0	MALIBA	Pos
4627	7/22/13	M	7.0	MALIBA	Neg
4628	7/22/13	M	40.0	KYANYA	Pos
4629	7/22/13	M	37.0	RWAKING	Neg
4630	7/22/13	F	50.0	KANYAN	Pos
4631	7/23/13	F	23.0	MALIBA	Neg
4632	7/23/13	M	8.0	KAKINDO	Pos
4633	7/23/13	M	6.0	MUWRO	Pos
4634	7/23/13	M	1.1	IZINGA	Neg
4635	7/23/13	M	5.0	BUZIBWERA	Neg
4636	7/23/13	F	2.5	BUZIBWERA	Pos
4637	7/23/13	F	50.0	MURAMBA	Pos
4638	7/23/13	M	5.0	NDUGHUTU	Pos
4639	7/23/13		6.0	.	Pos
4640	7/23/13	F	3.0	IZINGA	Pos
4641	7/23/13	F	4.0	KYANYA	Pos
4642	7/23/13	F	9.0	MALIBA	Pos
4643	7/23/13	F	5.0	IBANDA	Pos

4644	7/23/13	F	45.0	IZINGA	Neg
4645	7/23/13	F	3.0	BUZIBWERA	Neg
4646	7/23/13	F	18.0	NDUGHUTU	Pos
4647	7/23/13	F	39.0	NDUGHUTU	Neg
4648	7/23/13	M	0.4	MUHAMBO	Neg
4649	7/23/13	F	15.0	IBANDA	Neg
4650	7/23/13	F	73.0	RWAKING	Neg
4651	7/24/13	F	1.0	KANYAN	Neg
4652	7/24/13	F	66.0	NDUGHUTU	Neg
4653	7/24/13	F	16.0	KANYAN	Pos
4654	7/24/13	F	2.4	MURAMBA	Pos
4655	7/24/13	F	1.0	MURAMBA	Neg
4656	7/24/13	F	2.6	BUGOYE	Pos
4657	7/24/13	F	5.0	IBANDA	Neg
4658	7/24/13	F	4.5	RWAKING	Neg
4659	7/24/13	F	20.0	KANYAN	Neg
4660	7/24/13	F	9.0	IZINGA	Pos
4661	7/24/13	F	46.0	MALIBA	Neg
4662	7/24/13	F	8.0	MURAMBA	Pos
4663	7/24/13	F	24.0	MALIBA	Neg
4664	7/24/13	F	52.0	MUBUKU	Neg
4665	7/24/13	F	20.0	BUGOYE	Neg
4666	7/24/13	F	9.0	MURAMBA	Pos
4667	7/24/13	F	10.0	KIBOTA	Neg
4668	7/24/13	M	3.0	KIBOTA	Pos
4669	7/24/13	F	7.0	NYAKARUNGU	Neg
4670	7/24/13	M	17.0	BUNYANGONI	Neg
4671	7/24/13	F	60.0	IZINGA	Neg
4672	7/24/13	F	20.0	BUGOYE	Neg
4673	7/24/13	F	0.6	BULYANDEKE	Neg
4674	7/24/13	F	48.0	HIMA	Neg
4675	7/24/13	M	2.0	IZINGA	Neg
4676	7/25/13	F	2.0	NDUGHUTU	Pos
4677	7/25/13	F	42.0	NDUGHUTU	Neg
4678	7/25/13	F	81.0	NDUGHUTU	Neg
4679	7/25/13	F	14.0	KANYAN	Neg
4680	7/25/13	F	0.5	RWAKING	Neg
4681	7/25/13	M	22.0	NDUGHUTU	Neg
4682	7/25/13	M	2.0	BUGOYE	Neg
4683	7/25/13	M	4.0	BUGOYE	Pos
4684	7/25/13	M	9.0	IZINGA	Pos
4685	7/25/13	M	3.0	NDUGHUTU	Neg
4686	7/25/13	M	3.0	NDUGHUTU	Neg

4687	7/25/13	M	3.0	BUGOYE	Pos
4688	7/25/13	F	3.6	BUGOYE	Neg
4689	7/25/13	F	8.0	.	Pos
4690	7/25/13	F	10.0	BUGOYE	Neg
4691	7/25/13	M	5.0	MURAMBA	Neg
4692	7/25/13	F	45.0	NDUGHUTU	Neg
4693	7/25/13	F	28.0	IZINGA	Pos
4694	7/25/13	F	21.0	BUNYANGONI	Pos
4695	7/25/13	M	60.0	RWAKING	Pos
4696	7/25/13	M	25.0	BUHUNGA	Pos
4697	7/25/13	F	60.0	RWAKING	Neg
4698	7/25/13	F	2.0	IZINGA	Neg
4699	7/25/13	F	26.0	RWAKING	Neg
4700	7/25/13	F	76.0	IZINGA	Neg
4701	7/25/13	M	1.0	IZINGA	Neg
4702	7/25/13	F	9.0	MALIBA	Neg
4703	7/25/13	M	5.0	BUGOYE	Pos
4704	7/25/13	F	19.0	MURAMBA	Pos
4705	7/25/13	F	10.0	NDUGHUTU	Pos
4706	7/25/13	F	4.0	MURAMBA	Pos
4707	7/26/13	F	33.0	IZINGA	Neg
4708	7/26/13	F	8.0	RWAKING	Neg
4709	7/26/13	M	25.0	KANYAN	Pos
4710	7/26/13	M	10.0	NDUGHUTU	Neg
4711	7/26/13	F	2.0	NDUGHUTU	Neg
4712	7/26/13	F	60.0	KAKINDO	Pos
4713	7/26/13	F	1.6	MALIBA	Neg
4714	7/26/13	F	60.0	MALIBA	Pos
4715	7/26/13	F	0.7	BUGOYE	Pos
4716	7/26/13	F	18.0	BUGOYE	Neg
4717	7/26/13	F	1.9	RWAKING	Neg
4718	7/26/13	F	19.0	RWAKING	Neg
4719	7/26/13	M	0.5	KANYAN	Neg
4720	7/26/13	F	70.0	KISANGA	Neg
4721	7/26/13	F	77.0	BUGOYE	Pos
4722	7/26/13	F	3.0	MURAMBA	Pos
4723	7/26/13	F	0.2	KANYAN	Neg
4724	7/26/13	F	17.0	KANYAN	Neg
4725	7/26/13	F	20.0	MALIBA	Pos
4726	7/26/13	F	4.0	MALIBA	Neg
4727	7/26/13	M	7.0	KISANZI	Pos
4728	7/26/13	M	4.0	.	Pos
4729	7/26/13	M	1.5	BUNYANGONI	Pos

4730	7/26/13	F	14.0	MURAMBA	Pos
4731	7/26/13	M	21.0	KANYAN	Pos
4732	7/29/13	F	20.0	BUGOYE	Neg
4733	7/29/13	M	12.0	KISENDERA	Neg
4734	7/29/13	F	22.0	KISOJO	Neg
4735	7/29/13	F	29.0	IBANDA	Neg
4736	7/29/13	F	54.0	KANYAN	Pos
4737	7/29/13	F	17.0	IHANI	Neg
4738	7/29/13	F	20.0	BUZIBWERA	Pos
4739	7/29/13	F	21.0	BUGOYE	Neg
4740	7/29/13	F	26.0	KANYAN	Pos
4741	7/29/13	F	15.0	MURAMBA	Neg
4742	7/29/13	F	0.8	KIBIRIZI	Neg
4743	7/29/13	M	8.0	RWAKING	Neg
4744	7/29/13	F	21.0	RWAKING	Neg
4745	7/29/13	F	35.0	KIRONGO	Pos
4746	7/29/13	F	40.0	BUGOYE	Neg
4747	7/29/13	F	16.0	NDUGHUTU	Neg
4748	7/29/13	F	30.0	KISANZI	Neg
4749	7/29/13	F	25.0	IZINGA	Neg
4750	7/29/13	F	2.3	NYAKARUNGU	Neg
4751	7/30/13	M	3.0	RWAKING	Pos
4752	7/30/13	F	34.0	RWAKING	Pos
4753	7/30/13	F	1.0	RWAKING	Neg
4754	7/30/13	F	22.0	IBANDA	Neg
4755	7/30/13	F	0.8	BUGOYE	Neg
4756	7/30/13	F	75.0	BUNYANGONI	Pos
4757	7/30/13	F	70.0	IBANDA	Neg
4758	7/30/13	F	32.0	KANYAN	Pos
4759	7/30/13	M	12.0	IZINGA	Neg
4760	7/30/13	M	17.0	KAKINDO	Pos
4761	7/30/13	F	20.0	IBANDA	Pos
4762	7/30/13	F	4.0	BUGOYE	Pos
4763	7/30/13	F	45.0	IBANDA	Neg
4764	7/30/13	M	2.0	BUNYANGONI	Pos
4765	7/31/13	F	0.6	BUNYANGONI	Neg
4766	7/31/13	M	3.0	BUNYANGONI	Pos
4767	7/31/13	F	50.0	IZINGA	Neg
4768	7/31/13	F	2.0	IZINGA	Pos
4769	7/31/13	M	22.0	KATUMBA	Pos
4770	7/31/13	M	2.0	RWAKING	Pos
4771	7/31/13	M	0.1	NYAKARUNGU	Neg
4772	7/31/13	F	34.0	IZINGA	Neg

4773	7/31/13	F	22.0	NYAKARUNGU	Neg
4774	7/31/13	F	1.5	RWAKING	Pos
4775	7/31/13	M	80.0	RWAKING	Neg
4776	7/31/13	M	7.0	RWAKING	Neg
4777	7/31/13	F	70.0	KIHINDI	Pos
4778	7/31/13	F	60.0	KAKINDO	Pos
4779	7/31/13	F	19.0	BUGOYE	Neg
4780	7/31/13	F	45.0	RWAKING	Pos
4781	8/1/13	F	28.0	BUGOYE	Neg
4782	8/1/13	F	30.0	MALIBA	Neg
4783	8/1/13	F	19.0	BUNYANGONI	Neg
4784	8/1/13	F	13.0	BUNYANGONI	Neg
4785	8/1/13	F	32.0	MURAMBA	Neg
4786	8/1/13	F	1.5	KATOOKE	Pos
4787	8/1/13	F	20.0	KIKOKERA	Neg
4788	8/1/13	F	26.0	IZINGA	Pos
4789	8/1/13	F	1.2	RWAKING	Pos
4790	8/1/13	M	5.0	MURAMBA	Pos
4791	8/1/13	F	5.0	MALIBA	Neg
4792	8/1/13	F	13.0	KIKOKERA	Neg
4793	8/1/13	F	39.0	RWAKING	Neg
4794	8/1/13	F	7.0	KANYAN	Pos
4795	8/1/13	F	80.0	KANYAN	Pos
4796	8/1/13	F	21.0	BUGOYE	Pos
4797	8/1/13	F	22.0	MALIBA	Neg
4798	8/1/13	F	64.0	KAKINDO	Neg
4799	8/1/13	F	68.0	KYARUGOMOKA	Neg
4800	8/2/13	F	24.0	BUGOYE	Pos
4801	8/2/13	F	28.0	BUGOYE	Pos
4802	8/2/13	F	7.0	MALIBA	Neg
4803	8/2/13	M	9.0	MALIBA	Neg
4804	8/2/13	F	3.0	MALIBA	Pos
4805	8/2/13	F	19.0	MALIBA	Neg
4806	8/2/13	F	28.0	MALIBA	Pos
4807	8/2/13	M	24.0	BUGOYE	Pos
4808	8/2/13	M	13.0	NDUGHUTU	Pos
4809	8/2/13	F	1.0	IZINGA	Pos
4810	8/2/13	F	3.0	IZINGA	Pos
4811	8/2/13	F	45.0	RWAKING	Neg
4812	8/2/13	M	1.0	BUGOYE	Neg
4813	8/2/13	F	26.0	BUZIBWERA	Pos
4814	8/2/13	M	0.0	KISANGA	Neg
4815	8/2/13	F	.	NDUGHUTU	Pos

4816	8/2/13	F	0.7	NDUGHUTU	Neg
4817	8/2/13	M	3.5	IZINGA	Pos
4818	8/2/13	F	11.0	MURAMBA	Neg
4819	8/2/13	M	2.0	MALIBA	Pos
4820	8/2/13	F	36.0	KISAMBA	Pos
4821	8/2/13	F	38.0	KABUKERA	Pos
4822	8/2/13	F	11.0	KABUKERA	Pos
4823	8/2/13	M	6.0	KISENDERA	Pos
4824	8/2/13	F	42.0	BUGOYE	Pos
4825	8/5/13	M	25.0	RWAKING	Neg
4826	8/5/13	F	3.0	BUNYANGONI	Pos
4827	8/5/13	F	24.0	RWAKING	Pos
4828	8/5/13	F	8.0	KANYAN	Pos
4829	8/5/13	F	35.0	RWAKING	Pos
4830	8/5/13	F	17.0	KEMIHOKO	Pos
4831	8/5/13	F	15.0	MURAMBA	Pos
4832	8/5/13	F	23.0	MALIBA	Neg
4833	8/5/13	M	8.0	MALIBA	Neg
4834	8/5/13	F	16.0	BUGOYE	Neg
4835	8/5/13	F	36.0	MALIBA	Neg
4836	8/5/13	M	82.0	NYAKARUNGU	Neg
4837	8/5/13	F	3.5	RWAKING	Pos
4838	8/5/13	M	5.5	RWAKING	Pos
4839	8/5/13	M	20.0	KYARUGOMOKA	Neg
4840	8/5/13	F	40.0	MALIBA	Neg
4841	8/5/13	M	55.0	MURAMBA	Neg
4842	8/5/13	M	60.0	BUGOYE	Neg
4843	8/5/13	M	4.0	BUGOYE	Neg
4844	8/5/13	F	10.0	NYAKARUNGU	Neg
4845	8/5/13	F	5.0	NYAKARUNGU	Neg
4846	8/5/13	F	85.0	KAKINDO	Neg
4847	8/5/13	M	45.0	BUGOYE	Pos
4848	8/5/13	F	7.0	MALIBA	Neg
4849	8/5/13	F	2.0	NYAKARUNGU	Pos
4850	8/5/13	F	5.0	MALIBA	Neg
4851	8/5/13	M	2.0	MALIBA	Neg
4852	8/5/13	F	2.5	MALIBA	Pos
4853	8/5/13	M	32.0	MALIBA	Pos
4854	8/5/13	F	30.0	MALIBA	Neg
4855	8/5/13	F	4.0	KANYAN	Pos
4856	8/5/13	M	5.0	KISANZI	Neg
4857	8/5/13	F	0.6	KANYAN	Neg
4858	8/5/13	M	45.0	BUZIBWERA	Neg

4859	8/5/13	F	6.0	KANYAN	Pos
4860	8/5/13	M	21.0	BUGOYE	Neg
4861	8/6/13	M	36.0	NYAKARUNGU	Pos
4862	8/6/13	F	8.0	IBANDA	Pos
4863	8/6/13	M	20.0	IBANDA	Pos
4864	8/6/13	F	34.0	MUBUKU	Neg
4865	8/6/13	F	15.0	BUGOYE	Neg
4866	8/6/13	M	16.0	NDUGHUTU	Pos
4867	8/6/13	M	4.0	MALIBA	Pos
4868	8/6/13	F	17.0	MALIBA	Pos
4869	8/6/13	F	12.0	BUGOYE	Neg
4870	8/6/13	M	30.0	BUGOYE	Neg
4871	8/6/13	F	13.0	BUGOYE	Neg
4872	8/6/13	F	14.0	MURAMBA	Neg
4873	8/6/13	M	2.0	IZINGA	Pos
4874	8/6/13	M	18.0	MURAMBA	Pos
4875	8/6/13	M	18.0	MURAMBA	Neg
4876	8/6/13	F	5.0	KISANZI	Neg
4877	8/6/13	F	32.0	MURAMBA	Neg
4878	8/6/13	F	18.0	BUGOYE	Neg
4879	8/6/13	F	31.0	BUNYANGONI	Neg
4880	8/6/13	F	18.0	RWAKING	Pos
4881	8/6/13	F	37.0	BUGOYE	Pos
4882	8/6/13	F	35.0	IZINGA	Neg
4883	8/6/13	M	7.0	IZINGA	Pos
4884	8/6/13	F	18.0	BUGOYE	Pos
4885	8/6/13	F	33.0	IZINGA	Neg
4886	8/6/13	M	9.0	IZINGA	Pos
4887	8/6/13	M	50.0	ISULE	Pos
4888	8/6/13	F	2.0	NDUGHUTU	Neg
4889	8/6/13	M	16.0	IZINGA	Neg
4890	8/6/13	F	28.0	KIKOKERA	Neg
4891	8/6/13	M	13.0	IZINGA	Pos
4892	8/6/13	F	15.0	MURAMBA	Pos
4893	8/6/13	M	4.0	MURAMBA	Pos
4894	8/6/13	F	50.0	NDUGHUTU	Pos
4895	8/6/13	F	10.0	NDUGHUTU	Pos
4896	8/6/13	M	7.0	KANYAN	Pos
4897	8/6/13	M	4.0	KANYAN	Pos
4898	8/6/13	M	11.0	KANYAN	Pos
4899	8/6/13	F	37.0	KIRULI	Pos
4900	8/7/13	M	27.0	NDUGHUTU	Pos
4901	8/7/13	F	30.0	MURAMBA	Pos

4902	8/7/13	M	6.0	KANYAN	Neg
4903	8/7/13	F	53.0	MURAMBA	Pos
4904	8/7/13	F	26.0	KANYAN	Pos
4905	8/7/13	F	3.0	IZINGA	Pos
4906	8/7/13	F	1.7	IZINGA	Pos
4907	8/7/13	M	55.0	KYARUGOMOKA	Pos
4908	8/7/13	M	53.0	KATUMBA	Pos
4909	8/7/13	F	26.0	KANYAN	Neg
4910	8/7/13	F	50.0	RWAKING	Pos
4911	8/7/13	F	30.0	NYAKARUNGU	Pos
4912	8/7/13	F	5.0	KANYAN	Pos
4913	8/7/13	F	33.0	RWAKING	Pos
4914	8/7/13	M	27.0	NYAKARUNGU	Pos
4915	8/7/13	M	4.0	NDUGHUTU	Pos
4916	8/7/13	F	9.0	RWAKING	Neg
4917	8/7/13	M	16.0	MALIBA	Pos
4918	8/7/13	M	10.0	KATOOKE	Pos
4919	8/7/13	M	21.0	KANYAN	Neg
4920	8/7/13	F	1.0	KATOOKE	Neg
4921	8/7/13	F	12.0	BUNYANGONI	Pos
4922	8/7/13	M	5.0	KATUMBA	Neg
4923	8/7/13	F	20.0	KISANGA	Pos
4924	8/7/13	F	22.0	BUGOYE	Neg
4925	8/7/13	F	1.6	KISANZI	Neg
4926	8/9/13	M	1.3	KITOMA	Pos
4927	8/9/13	M	40.0	KISANGA	Pos
4928	8/9/13	F	4.0	MURAMBA	Pos
4929	8/9/13	F	22.0	KANYAN	Neg
4930	8/9/13	F	44.0	BUGOYE	Neg
4931	8/9/13	F	35.0	RWAKING	Pos
4932	8/9/13	F	2.2	KIKOKERA	Neg
4933	8/9/13	F	40.0	KIKOKERA	Neg
4934	8/9/13	F	23.0	NDUGHUTU	Neg
4935	8/9/13	F	21.0	MURAMBA	Pos
4936	8/9/13	M	21.0	BUGOYE	Neg
4937	8/9/13	F	14.0	MUHAMBO	Neg
4938	8/9/13	F	3.0	BUGOYE	Pos
4939	8/9/13	F	2.0	MALIBA	Pos
4940	8/9/13	F	26.0	MUBUKU	Pos
4941	8/9/13	F	25.0	NDUGHUTU	Pos
4942	8/9/13	F	4.0	NDUGHUTU	Pos
4943	8/9/13	F	10.0	NDUGHUTU	Pos
4944	8/9/13	M	9.0	KISANGA	Pos

4945	8/9/13	M	12.0	KYANYA	Pos
4946	8/9/13	M	10.0	KIKOKERA	Pos
4947	8/12/13	F	8.0	MALIBA	Pos
4948	8/12/13	F	10.0	MALIBA	Pos
4949	8/12/13	M	14.0	BUGOYE	Pos
4950	8/12/13	M	11.0	IZINGA	Pos
4951	8/12/13	M	32.0	IHANI	Pos
4952	8/12/13	F	34.0	MALIBA	Pos
4953	8/12/13	M	72.0	KANYAN	Neg
4954	8/12/13	M	13.0	BUGOYE	Neg
4955	8/12/13	F	29.0	IZINGA	Neg
4956	8/12/13	F	1.2	MURAMBA	Neg
4957	8/12/13	F	51.0	RWAKING	Pos
4958	8/12/13	M	2.0	BUGOYE	Neg
4959	8/12/13	M	2.0	BUGOYE	Neg
4960	8/12/13	M	4.0	BUGOYE	Pos
4961	8/12/13	M	23.0	MALIBA	Neg
4962	8/12/13	F	40.0	MURAMBA	Neg
4963	8/12/13	F	16.0	RWAKING	Neg
4964	8/12/13	M	4.0	RWAKING	Neg
4965	8/12/13	M	25.0	IZINGA	Pos
4966	8/12/13	F	7.0	KIBOTA	Pos
4967	8/12/13	F	19.0	KIHINDI	Pos
4968	8/12/13	F	21.0	MALIBA	Neg
4969	8/12/13	M	1.0	MALIBA	Pos
4970	8/12/13	F	50.0	KANYAN	Pos
4971	8/12/13	F	29.0	KANYAN	Pos
4972	8/12/13	F	3.0	KAKINDO	Pos
4973	8/12/13	F	5.0	MALIBA	Neg
4974	8/12/13	F	40.0	IBANDA	Neg
4975	8/12/13	F	2.0	KAKINDO	Neg
4976	8/12/13	F	21.0	MURAMBA	Neg
4977	8/12/13	F	20.0	RWAKING	Pos
4978	8/12/13	M	5.0	KIHARARA	Pos
4979	8/12/13	M	3.0	KIHARARA	Pos
4980	8/12/13	M	4.0	KIHARARA	Pos
4981	8/12/13	F	0.2	NDUGHUTU	Neg
4982	8/12/13	F	18.0	KIKOKERA	Pos
4983	8/12/13	F	2.1	KANYAN	Pos
4984	8/12/13	F	6.0	RWAKING	Pos
4985	8/12/13	F	12.0	KAKINDO	Pos
4986	8/12/13	F	30.0	KANYAN	Neg
4987	8/12/13	F	12.0	BUNYANGONI	Neg

4988	8/12/13	M	19.0	KANYAN	Neg
4989	8/12/13	M	1.0	KIHARARA	Pos
4990	8/12/13	M	6.0	MURAMBA	Neg
4991	8/12/13	M	5.0	BUGOYE	Pos
4992	8/12/13	M	12.0	IZINGA	Neg
4993	8/13/13	F	18.0	BUGOYE	Neg
4994	8/13/13	F	26.0	BUGOYE	Neg
4995	8/13/13	F	2.3	BUZIBWERA	Neg
4996	8/13/13	F	19.0	MURAMBA	Pos
4997	8/13/13	F	29.0	KATOOKE	Neg
4998	8/14/13	F	1.5	BUNYANGONI	Pos
4999	8/14/13	M	1.1	BUNYANGONI	Pos
5000	8/14/13	M	12.0	IBANDA	Pos
5001	8/14/13	M	0.1	RWAKING	Pos
5002	8/14/13	F	26.0	MALIBA	Neg
5003	8/14/13	F	12.0	BUGOYE	Neg
5004	8/14/13	F	55.0	MALIBA	Neg
5005	8/14/13	M	42.0	IZINGA	Pos
5006	8/14/13	M	30.0	MALIBA	Neg
5007	8/14/13	F	36.0	MALIBA	Pos
5008	8/14/13	M	4.0	MALIBA	Pos
5009	8/14/13	F	18.0	KIRULI	Pos
5010	8/14/13	M	60.0	MALIBA	Neg
5011	8/14/13	F	4.0	IZINGA	Pos
5012	8/14/13	F	20.0	KIBIRIZI	Neg
5013	8/14/13	M	33.0	KIBIRIZI	Neg
5014	8/14/13	M	2.0	KIRONGO	Pos
5015	8/14/13	M	1.3	MALIBA	Pos
5016	8/14/13	F	39.0	NYAKARUNGU	Neg
5017	8/14/13	M	2.0	KARUBATE	Neg
5018	8/14/13	F	1.5	NDUGHUTU	Pos
5019	8/14/13	M	1.6	MALIBA	Neg
5020	8/14/13	F	35.0	NDUGHUTU	Neg
5021	8/14/13	F	10.0	IZINGA	Pos
5022	8/15/13	F	35.0	KANYAN	Pos
5023	8/15/13	M	2.0	BUGOYE	Pos
5024	8/15/13	M	49.0	MUKATI	Pos
5025	8/15/13	F	39.0	BUGOYE	Pos
5026	8/15/13	M	11.0	KANYAN	Neg
5027	8/15/13	M	0.3	MUHAMBO	Pos
5028	8/15/13	F	30.0	MURAMBA	Pos
5029	8/15/13	M	22.0	NYAKARUNGU	Pos
5030	8/15/13	M	17.0	BUNYANGONI	Pos

5031	8/15/13	F	23.0	IBANDA	Neg
5032	8/15/13	M	60.0	MURAMBA	Pos
5033	8/15/13	M	1.5	NDUGHUTU	Pos
5034	8/15/13	F	25.0	IZINGA	Pos
5035	8/15/13	F	37.0	BUNYANGONI	Neg
5036	8/15/13	F	5.0	IZINGA	Pos
5037	8/15/13	M	2.3	BUNYANGONI	Pos
5038	8/15/13	F	18.0	KYARUGOMOKA	Neg
5039	8/15/13	M	13.0	BUGOYE	Pos
5040	8/15/13	M	17.0	KIHINDI	Neg
5041	8/15/13	F	0.5	KIHINDI	Neg
5042	8/16/13	F	40.0	IZINGA	Neg
5043	8/16/13	F	30.0	KISENDERA	Neg
5044	8/16/13	M	4.0	KISENDERA	Pos
5045	8/16/13	M	1.3	NDUGHUTU	Neg
5046	8/16/13	F	68.0	IBANDA	Neg
5047	8/16/13	M	7.0	KANYAN	Pos
5048	8/16/13	F	30.0	MURAMBA	Pos
5049	8/16/13	M	0.6	NDUGHUTU	Neg
5050	8/16/13	F	18.0	KANYAN	Neg
5051	8/19/13	M	36.0	MALIBA	Pos
5052	8/19/13	F	5.0	MALIBA	Pos
5053	8/19/13	F	28.0	KANYAN	Neg
5054	8/19/13	F	54.0	IBANDA	Neg
5055	8/19/13	F	65.0	MURAMBA	Neg
5056	8/19/13	F	45.0	KIBOTA	Neg
5057	8/19/13	F	60.0	IZINGA	Neg
5058	8/19/13	M	7.0	KANYAN	Pos
5059	8/19/13	M	4.0	MURAMBA	Pos
5060	8/19/13	M	1.3	KIRULI	Neg
5061	8/19/13	M	0.8	KIHINDI	Neg
5062	8/19/13	M	2.5	MALIBA	Pos
5063	8/19/13	M	1.6	MALIBA	Neg
5064	8/19/13	F	31.0	IBANDA	Pos
5065	8/19/13	F	6.0	KIBIRIZI	Pos
5066	8/19/13	F	16.0	RWAKING	Pos
5067	8/19/13	F	24.0	MURAMBA	Neg
5068	8/19/13	M	9.0	NDUGHUTU	Pos
5069	8/19/13	F	13.0	RWAKING	Pos
5070	8/19/13	M	21.0	IZINGA	Neg
5071	8/19/13	M	27.0	.	Pos
5072	8/19/13	F	18.0	IZINGA	Neg
5073	8/19/13	F	21.0	BUGOYE	Neg

5074	8/19/13	F	50.0	MURAMBA		Neg
5075	8/19/13	F	1.5	MURAMBA		Pos
5076	8/19/13	F	14.0	MALIBA		Neg
5077	8/19/13	F	21.0	MALIBA		Neg
5078	8/19/13	M	30.0	KYARUGOMOKA		Pos
5079	8/19/13	F	2.0	MALIBA		Pos
5080	8/19/13	M	70.0	MURAMBA		Neg
5081	8/19/13	M	45.0	BUGOYE		Neg
5082	8/19/13	F	9.0	BUGOYE		Pos
5083	8/19/13	F	9.0	KIHINDI		Neg
5084	8/19/13	F	1.0	MALIBA		Pos
5085	8/19/13	F	44.0	IZINGA		Pos
5086	8/19/13	F	1.1	MURAMBA		Pos
5087	8/19/13	M	0.6	NYABUSISI		Pos
5088	8/20/13	F	35.0	KISANZI		Pos
5089	8/20/13	M	4.0	MALIBA		Pos
5090	8/20/13	M	3.0	BUNYANGONI		Neg
5091	8/20/13	F	40.0	KYARUGOMOKA		Pos
5092	8/20/13	M	60.0	MALIBA		Pos
5093	8/20/13	F	30.0	MURAMBA		Pos
5094	8/20/13	F	4.0	RWAKING		Pos
5095	8/20/13	M	2.0	NDUGHUTU		Pos
5096	8/20/13	F	35.0	ISENGERA		Neg
5097	8/20/13	M	23.0	KANYAN		Neg
5098	8/20/13	M	20.0	BUNYANGONI		Pos
5099	8/20/13	M	27.0	KEMIHOKO		Neg
5100	8/20/13	F	6.0	BUNYANGONI		Pos
5101	8/20/13	F	28.0	BUNYANGONI		Pos
5102	8/20/13	M	5.0	RWAKING		Pos
5103	8/20/13	F	4.5	MALIBA		Pos
5104	8/20/13	F	2.0	KYANYA		Pos
5105	8/20/13	F	29.0	MURAMBA		Neg
5106	8/20/13	F	25.0	MALIBA		Pos
5107	8/20/13	F	1.0	KANYAN		Pos
5108	8/20/13	F	25.0	KANYAN		Neg
5109	8/20/13	F	2.0	RWAKING		Neg
5110	8/21/13	F	24.0	BUGOYE	Neg	
5111	8/21/13	F	51.0	IZINGA	Pos	
5112	8/21/13	M	23.0	BUGOYE	Neg	
5113	8/21/13	M	20.0	BUGOYE	Neg	
5114	8/21/13	F	7.0	MALIBA	Neg	
5115	8/21/13	M	39.0	MALIBA	Neg	
5116	8/21/13	M	0.6	RWAKING	Neg	

5117	8/21/13	M	15.0	KANYAN	Pos
5118	8/21/13	M	60.0	IBANDA	Pos
5119	8/21/13	M	20.0	MURAMBA	Neg
5120	8/21/13	M	50.0	MALIBA	Pos
5121	8/21/13	M	63.0	NDUGHUTU	Neg
5122	8/21/13	F	4.0	NDUGHUTU	Neg
5123	8/21/13	F	38.0	KIRULI	Neg
5124	8/21/13	M	10.0	KIRULI	Neg
5125	8/21/13	M	4.0	KIBIRIZI	Neg
5126	8/21/13	F	24.0	KANYAN	Neg
5127	8/21/13	M	50.0	KAKINDO	Neg
5128	8/21/13	F	0.2	KANYAN	Neg
5129	8/21/13	M	21.0	MURAMBA	Neg
5130	8/21/13	M	11.0	IZINGA	Pos
5131	8/21/13	F	20.0	BUZIBWERA	Pos
5132	8/21/13	F	50.0	BUGOYE	Pos
5133	8/21/13	M	0.4	MUHAMBO	Neg
5134	8/21/13	M	13.0	MURAMBA	Pos
5135	8/21/13	F	3.0	IZINGA	Neg
5136	8/21/13	F	32.0	IZINGA	Neg
5137	8/21/13	M	6.0	BUGOYE	Pos
5138	8/21/13	M	14.0	IZINGA	Neg
5139	8/21/13	F	17.0	NYAKARUNGU	Neg
5140	8/21/13	M	4.0	MUKATI	Neg
5141	8/21/13	M	30.0	RWAKING	Pos
5142	8/21/13	F	40.0	MURAMBA	Neg
5143	8/22/13	F	23.0	KANYAN	Neg
5144	8/22/13	M	39.0	NYANGONGE	Pos
5145	8/22/13	M	2.0	IZINGA	Pos
5146	8/22/13	M	2.5	KAKINDO	Neg
5147	8/22/13	F	40.0	KAKINDO	Neg
5148	8/22/13	F	24.0	KATOOKE	Neg
5149	8/22/13	F	10.0	KANYAN	Pos
5150	8/22/13	F	23.0	KATABUKENA	Neg
5151	8/22/13	F	0.3	NDUGHUTU	Neg
5152	8/22/13	F	3.5	RWAKING	Neg
5153	8/22/13	F	50.0	NDUGHUTU	Neg
5154	8/22/13	F	70.0	KISENDERA	Pos
5155	8/22/13	F	0.3	BUGOYE	Neg
5156	8/22/13	F	3.1	IZINGA	Neg
5157	8/22/13	F	37.0	MURAMBA	Neg
5158	8/22/13	M	21.0	BUZIBWERA	Neg
5159	8/22/13	F	3.5	RWAKING	Pos

5160	8/22/13	F	29.0	KISAMBA	Neg
5161	8/22/13	F	2.5	KAKINDO	Pos
5162	8/22/13	M	0.8	KYAKIMA	Neg
5163	8/22/13	F	30.0	KYARUGOMOKA	Pos
5164	8/22/13	M	2.0	NDUGHUTU	Pos
5165	8/22/13	M	4.0	NDUGHUTU	Pos
5166	8/22/13	F	16.0	KYAKIMA	Pos
5167	8/22/13	M	23.0	KIBIRIZI	Neg
5168	8/22/13	M	12.0	KAKINDO	Neg
5169	8/22/13	F	45.0	NDUGHUTU	Neg
5170	8/23/13	F	35.0	BUGOYE	Pos
5171	8/23/13	F	35.0	MALIBA	Pos
5172	8/23/13	M	4.0	MALIBA	Neg
5173	8/23/13	F	27.0	BUGOYE	Neg
5174	8/23/13	F	27.0	KANYAN	Neg
5175	8/23/13	M	13.0	KANYAN	Pos
5176	8/23/13	F	10.0	IZINGA	Pos
5177	8/23/13	F	26.0	KANYAN	Neg
5178	8/23/13	M	1.0	MURAMBA	Pos
5179	8/23/13	M	25.0	KANYAN	Pos
5180	8/23/13	F	50.0	MALIBA	Neg
5181	8/23/13	F	13.0	NDUGHUTU	Neg
5182	8/23/13	M	15.0	RWAKING	Pos
5183	8/23/13	F	5.0	MALIBA	Neg
5184	8/23/13	F	41.0	MALIBA	Neg
5185	8/23/13	F	0.8	RWAKING	Pos
5186	8/23/13	M	2.0	MALIBA	Pos
5187	8/23/13	M	11.0	KIKOKERA	Pos
5188	8/23/13	M	3.0	MALIBA	Pos
5189	8/23/13	F	18.0	NDUGHUTU	Neg
5190	8/23/13	M	8.0	KABUYIRI	Neg
5191	8/23/13	F	12.0	NDUGHUTU	Neg
5192	8/23/13	M	0.9	MALIBA	Pos
5193	8/23/13	F	55.0	KAGANDO	Pos
5194	8/23/13	F	14.0	MALIBA	Neg
5195	8/23/13	M	0.8	KATOOKE	Neg
5196	8/23/13	M	55.0	BUNYANGONI	Neg
5197	8/23/13	F	24.0	NDUGHUTU	Neg
5198	8/23/13	F	34.0	NDUGHUTU	Pos
5199	8/23/13	F	4.0	RWAKING	Pos
5200	8/23/13	F	50.0	MALIBA	Neg
5201	8/23/13	F	0.4	KANYAN	Neg
5202	8/23/13	F	52.0	BUGOYE	Neg

5203	8/23/13	M	39.0	IBANDA	Pos	
5204	8/23/13	F	40.0	MURAMBA	Pos	
5205	8/23/13	F	25.0	NDUGHUTU	Neg	
5206	8/23/13	F	20.0	BUZIBWERA	Pos	
5207	8/23/13	M	21.0	MUHAMBO	Pos	
5208	8/23/13	F	0.8	RWAKING	Neg	
5209	8/23/13	M	50.0	IBANDA	Neg	
5210	8/23/13	F	19.0	KANYAN	Neg	
5211	8/23/13	M	4.0	IZINGA	Pos	
5212	8/23/13	F	0.7	IZINGA	Pos	
5213	8/23/13	F	21.0	RWAKING	Pos	
5214	8/26/13	M	2.0	MALIBA	Pos	Pos
5215	8/26/13	F	20.0	KISANZI	Neg	Neg
5216	8/26/13	F	50.0	KAKINDO	Neg	
5217	8/26/13	F	2.0	NDUGHUTU	Pos	Pos
5218	8/26/13	F	1.5	KANYAN	Pos	
5219	8/26/13	M	9.0	MALIBA	Pos	
5220	8/26/13	F	65.0	MALIBA	Pos	
5221	8/26/13	F	17.0	NDUGHUTU	Pos	
5222	8/26/13	M	11.0	MURAMBA	Neg	
5223	8/26/13	F	12.0	NDUGHUTU	Pos	
5224	8/26/13	F	2.0	NDUGHUTU	Pos	
5225	8/26/13	F	32.0	IZINGA	Pos	
5226	8/26/13	F	24.0	BUGOYE	Neg	
5227	8/26/13	F	35.0	KAKINDO	Pos	
5228	8/26/13	M	9.0	MALIBA	Pos	
5229	8/26/13	F	4.0	NDUGHUTU	Pos	
5230	8/26/13	M	5.0	MALIBA	Neg	
5231	8/26/13	F	13.0	.	Pos	
5232	8/26/13	F	19.0	KANYAN	Neg	
5233	8/26/13	F	8.0	NDUGHUTU	Pos	
5234	8/26/13	F	6.0	NDUGHUTU	Pos	
5235	8/26/13	F	29.0	MALIBA	Pos	
5236	8/26/13	F	5.0	KISOJO	Pos	
5237	8/26/13	F	34.0	MALIBA	Neg	
5238	8/26/13	F	12.0	MURAMBA	Pos	
5239	8/26/13	F	30.0	MALIBA	Pos	
5240	8/26/13	F	2.0	BUNYANGONI	Neg	
5241	8/26/13	F	9.0	.	Neg	
5242	8/26/13	F	35.0	RWAKING	Pos	
5243	8/26/13	F	0.3	RWAKING	Neg	
5244	8/26/13	M	0.9	IZINGA	Pos	
5245	8/26/13	F	9.0	KANYAN	Pos	

5246	8/26/13	F	1.8	IZINGA	Pos	
5247	8/26/13	F	17.0	MALIBA	Pos	
5248	8/26/13	F	2.0	MALIBA	Pos	
5249	8/26/13	M	8.0	MALIBA	Neg	
5250	8/26/13	F	33.0	BUGOYE	Pos	
5251	8/26/13	M	2.0	MURAMBA	Neg	
5252	8/26/13	F	7.0	MALIBA	Neg	
5253	8/26/13	M	1.5	BUGOYE	Neg	
5254	8/26/13	F	34.0	.	Neg	
5255	8/26/13	F	13.0	NDUGHUTU	Pos	
5256	8/26/13	F	39.0	MURAMBA	Neg	
5257	8/26/13	F	5.0	NDUGHUTU	Neg	
5258	8/26/13	F	29.0	KATUMBA	Neg	
5259	8/26/13	F	1.8	MALIBA	Pos	
5260	8/26/13	M	70.0	KIKOKERA	Neg	
5261	8/27/13	M	20.0	RWAKING	Pos	
5262	8/27/13	F	32.0	KIKOKERA	Neg	
5263	8/27/13	F	40.0	RWAKING	Neg	
5264	8/27/13	F	24.0	NDUGHUTU	Pos	
5265	8/27/13	F	27.0	KAKINDO	Pos	
5266	8/27/13	M	1.8	KAKINDO	Neg	
5267	8/27/13	F	10.0	MURAMBA	Neg	
5268	8/27/13	M	7.0	IBANDA	Neg	
5269	8/27/13	M	5.0	IZINGA	Pos	
5270	8/27/13	F	9.0	MALIBA	Pos	
5271	8/27/13	M	6.0	MALIBA	Pos	
5272	8/27/13	F	0.4	KYARUGOMOKA	Neg	
5273	8/27/13	F	1.5	BIKONE	Pos	
5274	8/27/13	F	3.0	NYABUSISI	Neg	
5275	8/27/13	M	36.0	BUGOYE	Pos	
5276	8/27/13	F	1.6	BUGOYE	Pos	Pos
5277	8/27/13	F	51.0	RWAKING	Neg	
5278	8/27/13	M	13.0	KAKINDO	Pos	
5279	8/27/13	M	4.0	KISANZI	Pos	
5280	8/27/13	M	17.0	MURAMBA	Neg	
5281	8/27/13	F	18.0	IBANDA	Neg	
5282	8/27/13	F	48.0	IBANDA	Pos	
5283	8/27/13	F	23.0	MUBUKU	Neg	
5284	8/27/13	M	0.3	MALIBA	Neg	
5285	8/27/13	F	14.0	KATOOKE		Neg
5286	8/28/13	F	10.0	IBANDA		Pos
5287	8/28/13	F	1.8	IZINGA	Pos	
5288	8/28/13	F	60.0	KYANYA	Neg	

5289	8/28/13	M	3.0	BUGOYE	Pos
5290	8/28/13	M	1.0	KIKOKERA	Neg
5291	8/28/13	F	19.0	MURAMBA	Neg
5292	8/28/13	F	8.0	NDUGHUTU	Pos
5293	8/28/13	F	7.0	MURAMBA	Pos
5294	8/28/13	F	11.0	RWAKING	Pos
5295	8/28/13	M	24.0	KAKINDO	Pos
5296	8/28/13	F	50.0	IBANDA	Neg
5297	8/28/13	F	20.0	KIKOKERA	Neg
5298	8/28/13	F	6.0	MURAMBA	Neg
5299	8/28/13	M	11.0	MURAMBA	Neg
5300	8/28/13	F	25.0	KATUMBA	Neg
5301	8/28/13	M	4.5	IZINGA	Pos
5302	8/28/13	F	54.0	BUNYANGONI	Neg
5303	8/28/13	F	1.0	BUGOYE	Pos
5304	8/28/13	F	32.0	MURAMBA	Neg
5305	8/28/13	M	23.0	KANYAN	Neg
5306	8/28/13	F	5.0	KANYAN	Pos
5307	8/28/13	M	12.0	IZINGA	Pos
5308	8/28/13	M	15.0	IZINGA	Neg
5309	8/28/13	F	28.0	IZINGA	Pos
5310	8/28/13	F	40.0	KANYAN	Neg
5311	8/28/13	F	31.0	NDUGHUTU	Neg
5312	8/28/13	F	19.0	MURAMBA	Neg
5313	8/28/13	M	.	KATUMBA	Neg
5314	8/28/13	F	4.0	MALIBA	Pos
5315	8/28/13	M	3.0	BUZIBWERA	Pos
5316	8/28/13	M	7.0	MALIBA	Pos
5317	8/28/13	F	2.0	NYAKARUNGU	Pos
5318	8/28/13	F	8.0	RWAKING	Pos
5319	8/28/13	F	3.0	KISOJO	Pos
5320	8/28/13	M	7.0	MALIBA	Pos
5321	8/28/13	M	17.0	NDUGHUTU	Neg
5322	8/29/13	M	30.0	RWAKING	Neg
5323	8/29/13	F	21.0	BUNYANGONI	Pos
5324	8/29/13	F	30.0	MURAMBA	Neg
5325	8/29/13	M	15.0	RWAKING	Pos
5326	8/29/13	M	23.0	KIKOKERA	Pos
5327	8/29/13	M	5.0	KANYAN	Pos
5328	8/29/13	M	12.0	MURAMBA	Pos
5329	8/29/13	M	6.0	MURAMBA	Pos
5330	8/29/13	F	3.3	RWAKING	Pos
5331	8/29/13	M	71.0	MURAMBA	Neg

5332	8/29/13	F	22.0	NYAKARUNGU	Neg	
5333	8/29/13	F	1.3	NYAKARUNGU	Neg	
5334	8/29/13	M	14.0	NYAKARUNGU	Neg	
5335	8/29/13	M	2.1	KAKINDO	Pos	Pos
5336	8/29/13	F	35.0	RWAKING	Neg	
5337	8/29/13	M	22.0	KYAYABO	Neg	
5338	8/29/13	F	12.0	BUGOYE	Pos	
5339	8/29/13	F	5.0	BUGOYE	Pos	
5340	8/30/13	F	54.0	KANYAN	Neg	
5341	8/30/13	M	25.0	NDUGHUTU	Pos	
5342	8/30/13	F	6.0	KYARUGOMOKA	Pos	
5343	8/30/13	M	60.0	MALIBA	Neg	
5344	8/30/13	F	2.0	KIKOKERA	Pos	
5345	8/30/13	M	49.0	KISOJO	Neg	
5346	8/30/13	M	1.7	NDUGHUTU	Neg	
5347	8/30/13	M	26.0	NDUGHUTU	Pos	
5348	8/30/13	F	9.0	BUGOYE	Pos	
5349	8/30/13	F	15.0	IZINGA	Pos	
5350	8/30/13	F	34.0	BUGOYE	Neg	
5351	8/30/13	F	35.0	RWAKING	Neg	
5352	8/30/13	M	1.7	BUNYANGONI	Neg	
5353	8/30/13	M	18.0	MALIBA	Pos	
5354	8/30/13	F	18.0	IZINGA	Pos	
5355	8/30/13	F	12.0	BUGOYE	Neg	
5356	8/30/13	M	14.0	BUGOYE	Pos	
5357	8/30/13	F	5.0	IZINGA	Neg	
5358	8/30/13	F	5.0	MALIBA	Neg	
5359	8/30/13	F	45.0	MALIBA	Neg	
5360	8/30/13	F	60.0	KIBOTA	Pos	
5361	8/30/13	F	48.0	KISOJO	Pos	Pos
5362	8/30/13	F	7.0	RWAKING	Neg	
5363	8/30/13	F	7.0	MALIBA	Pos	
5364	8/30/13	F	1.0	MUHAMBO	Neg	
5365	8/30/13	M	4.0	RWAKING	Pos	
5366	8/30/13	M	15.0	KISANZI	Pos	
5367	8/30/13	F	80.0	MURAMBA	Neg	
5368	8/30/13	F	9.0	BUZIBWERA	Pos	
5369	8/30/13	F	13.0	MALIBA	Neg	
5370	8/30/13	M	1.5	MALIBA	Pos	
5371	8/30/13	F	4.0	IZINGA	Pos	
5372	8/30/13	M	1.5	BUZIBWERA	Pos	
5373	8/30/13	F	4.0	KAKINDO	Pos	
5374	8/30/13	M	1.3	NYANGONGE	Neg	

5375	9/2/13	F	31.0	KANYAN	Neg	
5376	9/2/13	F	2.0	KANYAN	Neg	
5377	9/2/13	F	0.3	KANYAN	Neg	
5378	9/2/13	M	1.1	IBANDA	Neg	
5379	9/2/13	M	2.0	RWAKING	Pos	
5380	9/2/13	F	32.0	RWAKING	Pos	
5381	9/2/13	F	3.0	NDUGHUTU	Pos	
5382	9/2/13	F	7.0	IBANDA	Neg	
5383	9/2/13	F	49.0	KANYAN	Pos	
5384	9/2/13	M	4.0	KANYAN	Pos	
5385	9/2/13	F	7.0	BUNYANGONI	Pos	
5386	9/2/13	M	5.0	NDUGHUTU	Pos	
5387	9/2/13	M	11.0	BUNYANGONI	Neg	
5388	9/2/13	F	4.0	BUNYANGONI	Neg	
5389	9/2/13	F	8.0	BUNYANGONI	Neg	
5390	9/2/13	F	0.7	BUNYANGONI	Neg	
5391	9/2/13	F	32.0	KANYAN	Neg	
5392	9/2/13	M	27.0	MALIBA	Neg	
5393	9/2/13	M	90.0	IBANDA	Neg	
5394	9/2/13	F	3.0	NDUGHUTU	Pos	
5395	9/2/13	F	35.0	BUNYANGONI	Neg	
5396	9/2/13	F	10.0	NDUGHUTU	Neg	
5397	9/2/13	F	50.0	BUNYANGONI	Neg	
5398	9/2/13	F	23.0	BUGOYE	Neg	
5399	9/2/13	M	4.0	MALIBA	Neg	Neg
5400	9/2/13	F	28.0	MALIBA	Neg	
5401	9/2/13	F	2.0	RWAKING	Neg	
5402	9/2/13	M	9.0	RWAKING	Pos	
5403	9/2/13	F	23.0	BUGOYE	Pos	
5404	9/2/13	M	7.0	KATOOKE	Neg	
5405	9/2/13	F	9.0	KATOOKE	Neg	
5406	9/2/13	F	22.0	MUHAMBO	Pos	Pos
5407	9/2/13	M	15.0	KYABUKO	Pos	
5408	9/2/13	F	28.0	ISULE	Neg	
5409	9/2/13	F	4.0	MURAMBA	Neg	
5410	9/2/13	M	17.0	MALIBA	Neg	
5411	9/2/13	F	38.0	KIGORO	Pos	
5412	9/2/13	M	40.0	ISULE	Neg	
5413	9/2/13	F	4.0	KYANYA	Pos	
5414	9/2/13	M	33.0	MUHAMBO	Neg	
5415	9/2/13	M	2.0	MUHAMBO	Neg	
5416	9/2/13	M	16.0	IZINGA	Pos	
5417	9/2/13	M	17.0	RWAKING		Pos

5418	9/2/13	M	4.7	RWAKING	Pos	
5419	9/2/13	F	0.6	BULYANDEKE	Pos	
5420	9/2/13	F	18.0	NDUGHUTU	Neg	
5421	9/2/13	M	2.3	IZINGA		Neg
5422	9/2/13	M	4.0	MALIBA	Neg	
5423	9/2/13	F	18.0	MALIBA		Neg
5424	9/2/13	F	0.8	IZINGA	Pos	
5425	9/2/13	F	6.0	RWAKING	Pos	
5426	9/2/13	F	4.0	NYAKARUNGU	Pos	
5427	9/2/13	M	20.0	KATOOKE	Pos	
5428	9/2/13	F	0.5	MALIBA	Pos	
5429	9/2/13	M	19.0	IHANI	Neg	
5430	9/2/13	F	20.0	MURAMBA	Neg	
5431	9/2/13	M	9.0	IZINGA	Neg	
5432	9/2/13	F	30.0	KYARUGOMOKA	Neg	
5433	9/2/13	F	21.0	BUGOYE	Neg	
5434	9/3/13	F	17.0	KIBOTA	Pos	
5435	9/3/13	F	2.0	RWAKING	Neg	
5436	9/3/13	F	50.0	MURAMBA	Neg	
5437	9/3/13	M	0.8	BUNYANGONI	Neg	
5438	9/3/13	M	20.0	NYAKARUNGU	Neg	
5439	9/3/13	F	58.0	IBANDA	Neg	
5440	9/3/13	M	57.0	IBANDA	Neg	
5441	9/3/13	M	16.0	MURAMBA	Neg	
5442	9/3/13	F	9.0	KIKOKERA	Neg	
5443	9/3/13	F	20.0	RWAKING	Neg	
5444	9/3/13	M	2.0	ISENGERA	Neg	
5445	9/3/13	M	3.0	RWAKING	Neg	
5446	9/3/13	M	4.5	HIMA	Neg	
5447	9/3/13	M	2.0	NYAKARUNGU	Neg	
5448	9/3/13	M	7.0	RWAKING	Neg	
5449	9/3/13	F	42.0	RWAKING	Neg	
5450	9/3/13	F	10.0	NDUGHUTU	Neg	
5451	9/3/13	F	45.0	IZINGA	Neg	
5452	9/3/13	F	0.4	BUGOYE	Neg	
5453	9/3/13	M	0.5	BUGOYE	Pos	
5454	9/3/13	F	1.0	MAGHOMA	Neg	
5455	9/3/13	F	1.4	BUZIBWERA	Pos	
5456	9/4/13	F	1.0	IZINGA	Pos	
5457	9/4/13	M	4.0	BUGOYE	Neg	
5458	9/4/13	F	43.0	MALIBA	Neg	
5459	9/4/13	F	0.6	MUBUKU	Neg	
5460	9/4/13	F	50.0	NDUGHUTU	Neg	

5461	9/4/13	F	2.0	NDUGHUTU	Neg
5462	9/4/13	F	18.0	MALIBA	Pos
5463	9/4/13	F	59.0	RWAKING	Neg
5464	9/4/13	M	4.0	.	Pos
5465	9/4/13	M	0.3	NDUGHUTU	Neg
5466	9/4/13	F	1.5	BUGOYE	Neg
5467	9/4/13	F	0.3	MURAMBA	Neg
5468	9/4/13	F	20.0	.	Neg
5469	9/4/13	F	33.0	IZINGA	Pos
5470	9/4/13	F	0.9	IZINGA	Pos
5471	9/4/13	M	80.0	MALIBA	Neg
5472	9/4/13	F	22.0	MALIBA	Neg
5473	9/4/13	F	28.0	MURAMBA	Pos
5474	9/4/13	M	0.8	MALIBA	Neg
5475	9/4/13	M	3.0	RWAKING	Neg
5476	9/4/13	F	5.0	MALIBA	Pos
5477	9/4/13	F	5.0	MALIBA	Pos
5478	9/4/13	M	1.0	NDUGHUTU	Neg
5479	9/4/13	M	23.0	BUZIBWERA	Pos
5480	9/4/13	F	25.0	BUGOYE	Pos
5481	9/4/13	F	50.0	KANYAN	Pos
5482	9/4/13	F	35.0	ISULE	Neg
5483	9/4/13	M	9.0	MURAMBA	Neg
5484	9/4/13	F	13.0	MURAMBA	Neg
5485	9/4/13	M	50.0	MALIBA	Neg
5486	9/4/13	M	26.0	MALIBA	Neg
5487	9/4/13	M	20.0	NDUGHUTU	Pos
5488	9/4/13	F	0.8	IZINGA	Neg
5489	9/4/13	M	13.0	MALIBA	Pos
5490	9/4/13	F	0.1	KANYAN	Neg
5491	9/4/13	F	7.0	BUZIBWERA	Pos
5492	9/4/13	M	8.0	RWAKING	Pos
5493	9/4/13	M	0.8	MURAMBA	Neg
5494	9/4/13	M	19.0	NDUGHUTU	Pos
5495	9/4/13	M	20.0	KASESE	Neg
5496	9/5/13	F	18.0	BUGOYE	Pos
5497	9/5/13	F	62.0	KANYAN	Neg
5498	9/5/13	F	13.0	KYANYA	Pos
5499	9/5/13	F	3.0	MUHAMBO	Pos
5500	9/5/13	F	21.0	KISENDERA	Neg
5501	9/5/13	M	0.3	NDUGHUTU	Pos
5502	9/5/13	F	38.0	KANYAN	Pos
5503	9/5/13	F	25.0	IZINGA	Pos

5504	9/5/13	F	24.0	MUBUKU	Neg
5505	9/5/13	M	7.0	KANYAN	Pos
5506	9/5/13	F	46.0	BUNYANGONI	Neg
5507	9/5/13	M	22.0	KIBIRIZI	Neg
5508	9/5/13	M	1.5	KATUMBA	Neg
5509	9/5/13	F	36.0	KYANYA	Pos
5510	9/5/13	M	13.0	MALIBA	Pos
5511	9/6/13	M	14.0	NDUGHUTU	Pos
5512	9/6/13	F	13.0	IZINGA	Pos
5513	9/6/13	F	4.0	IZINGA	Pos
5514	9/6/13	F	15.0	NDUGHUTU	Pos
5515	9/6/13	f	37.0	KANYAN	Pos
5516	9/6/13	F	28.0	BUHUNGA	Neg
5517	9/6/13	F	28.0	BUGOYE	Neg
5518	9/6/13	F	3.0	BUGOYE	Neg
5519	9/6/13	F	7.0	KIKOKERA	Pos
5520	9/6/13	F	0.3	KIRONGO	Neg
5521	9/6/13	F	26.0	KANYAN	Pos
5522	9/6/13	F	19.0	NDUGHUTU	Pos
5523	9/6/13	F	35.0	KATUMBA	Neg
5524	9/6/13	F	37.0	MURAMBA	Neg
5525	9/6/13	F	0.8	MALIBA	Neg
5526	9/6/13	F	12.0	MALIBA	Neg
5527	9/6/13	M	40.0	IZINGA	Pos
5528	9/6/13	F	16.0	KANYAN	Neg
5529	9/6/13	F	13.0	IZINGA	Pos
5530	9/6/13	F	19.0	MALIBA	Pos
5531	9/6/13	M	7.0	BUZIBWERA	Pos
5532	9/6/13	M	2.0	NDUGHUTU	Neg
5533	9/6/13	M	5.0	NDUGHUTU	Pos
5534	9/6/13	M	2.5	BUNYANGONI	Pos
5535	9/6/13	F	80.0	MURAMBA	Neg
5536	9/6/13	M	23.0	BUZIBWERA	Neg
5537	9/6/13	M	20.0	IBANDA	Neg
5538	9/9/13	M	21.0	BUGOYE	Neg
5539	9/9/13	F	22.0	BUGOYE	Pos
5540	9/9/13	M	48.0	KYANYA	Pos
5541	9/9/13	F	23.0	RWAKING	Neg
5542	9/9/13	M	7.0	MALIBA	Pos
5543	9/9/13	F	30.0	MALIBA	Neg
5544	9/9/13	F	0.3	MALIBA	Neg
5545	9/9/13	F	35.0	MALIBA	Neg
5546	9/9/13	F	20.0	KYARUGOMOKA	Neg

5547	9/9/13	F	40.0	RUBONI	Neg	
5548	9/9/13	M	0.8	MURAMBA	Neg	
5549	9/9/13	M	0.6	KATOOKE	Pos	
5550	9/9/13	F	25.0	KATOOKE	Neg	
5551	9/9/13	M	7.0	MALIBA	Pos	
5552	9/9/13	M	16.0	BUGOYE	Pos	
5553	9/9/13	M	10.0	IZINGA	Pos	
5554	9/9/13	F	22.0	IZINGA	Neg	
5555	9/9/13	M	9.0	BUNYANGONI	Neg	
5556	9/9/13	F	40.0	MAGHOMA	Neg	
5557	9/9/13	F	34.0	BUNYANGONI	Neg	
5558	9/9/13	F	12.0	KAKINDO	Pos	
5559	9/9/13	M	9.0	BUGOYE	Pos	Pos
5560	9/9/13	F	4.0	BULINDUGURU	Neg	Neg
5561	9/9/13	F	35.0	BUGOYE	Neg	
5562	9/9/13	M	20.0	BUNYANGONI	Neg	
5563	9/9/13	F	29.0	NDUGHUTU	Neg	
5564	9/9/13	F	17.0	IZINGA	Pos	
5565	9/9/13	F	40.0	BIKONE	Neg	
5566	9/9/13	M	11.0	IBANDA	Pos	
5567	9/9/13	F	4.0	MURAMBA	Neg	
5568	9/9/13	F	40.0	MURAMBA	Neg	
5569	9/9/13	M	2.0	MURAMBA	Pos	
5570	9/9/13	M	47.0	RWAKING	Pos	
5571	9/9/13	F	17.0	BULYANDEKE	Pos	
5572	9/9/13	M	4.0	RWAKING	Neg	
5573	9/9/13	M	35.0	IBANDA	Neg	
5574	9/9/13	M	17.0	IZINGA	Pos	
5575	9/9/13	M	20.0	KYARUGOMOKA	Neg	
5576	9/9/13	F	20.0	BUNYANGONI	Neg	
5577	9/9/13	M	12.0	KYARUGOMOKA	Pos	
5578	9/9/13	M	17.0	NYAKARUNGU	Neg	
5579	9/9/13	F	14.0	RWAKING	Pos	
5580	9/9/13	M	4.0	IZINGA	Pos	
5581	9/9/13	M	5.0	MUHAMBO	Neg	
5582	9/9/13	M	0.8	MURAMBA	Neg	
5583	9/9/13	M	0.9	MURAMBA	Neg	
5584	9/9/13	F	36.0	KIRONGO	Neg	
5585	9/9/13	M	1.5	KUNYANGABA	Pos	
5586	9/9/13	M	0.7	NDUGHUTU	Neg	
5587	9/9/13	M	13.0	KISAMBA	Neg	
5588	9/9/13	F	0.2	BUGOYE	Neg	
5589	9/9/13	F	0.9	KANYAN	Pos	

5590	9/9/13	F	3.0	MALIBA	Neg
5591	9/9/13	F	35.0	IBANDA	Neg
5592	9/9/13	F	25.0	IBANDA	Neg
5593	9/9/13	M	12.0	IHANI	Neg
5594	9/9/13	F	30.0	MURAMBA	Neg
5595	9/9/13	F	40.0	KANYAN	Neg
5596	9/9/13	M	12.0	BUGOYE	Neg
5597	9/9/13	M	6.0	IHANI	Neg
5598	9/9/13	M	90.0	IBANDA	Neg
5599	9/11/13	M	1.5	KYARUGOMOKA	Neg
5600	9/11/13	M	20.0	MURAMBA	Neg
5601	9/11/13	M	10.0	MURAMBA	Neg
5602	9/11/13	F	17.0	RWAKING	Neg
5603	9/11/13	M	36.0	MALIBA	Neg
5604	9/11/13	F	0.9	IBANDA	Neg
5605	9/11/13	M	42.0	MALIBA	Neg
5606	9/11/13	F	24.0	BUZIBWERA	Neg
5607	9/11/13	M	29.0	NDUGHUTU	Neg
5608	9/11/13	F	7.0	NYAKABUGHA	Neg
5609	9/11/13	F	0.5	NYAKABUGHA	Neg
5610	9/11/13	M	20.0	KIKOKERA	Neg
5611	9/11/13	M	25.0	RWAKING	Neg
5612	9/11/13	F	24.0	BUGOYE	Pos
5613	9/11/13	F	0.8	MUBUKU	Neg
5614	9/11/13	F	23.0	MALIBA	Neg
5615	9/11/13	F	33.0	MURAMBA	Neg
5616	9/11/13	F	9.0	IZINGA	Pos
5617	9/11/13	M	34.0	KIBIRIZI	Neg
5618	9/11/13	M	50.0	MALIBA	Neg
5619	9/11/13	F	50.0	IZINGA	Neg
5620	9/11/13	F	50.0	IHANI	Neg
5621	9/11/13	F	25.0	IZINGA	Pos
5622	9/11/13	F	1.5	BUGOYE	Neg
5623	9/11/13	F	6.0	RUBONI	Neg
5624	9/11/13	F	39.0	NYAKALENGIJO	Pos
5625	9/11/13	F	1.6	KAKINDO	Pos
5626	9/11/13	M	6.0	IBANDA	Pos
5627	9/11/13	M	29.0	MALIBA	Pos
5628	9/11/13	M	5.0	BUGOYE	Pos
5629	9/11/13	M	45.0	BUHUNGA	Pos
5630	9/11/13	M	21.0	IZINGA	Pos
5631	9/11/13	F	10.0	KATOOKE	Neg
5632	9/11/13	M	3.0	IZINGA	Neg

5633	9/11/13	F	27.0	RUBONI	Neg	
5634	9/11/13	F	33.0	RWAKING	Neg	
5635	9/11/13	M	0.1	MUHAMBO	Neg	
5636	9/11/13	F	0.1	MUHAMBO	Neg	
5637	9/11/13	F	1.0	MALIBA	Neg	
5638	9/11/13	F	38.0	KATUMBA	Neg	
5639	9/13/13	F	20.0	MURAMBA	Neg	
5640	9/13/13	M	55.0	BUNYANGONI	Neg	
5641	9/13/13	F	40.0	NDUGHUTU	Pos	
5642	9/13/13	M	60.0	BUGOYE	Pos	
5643	9/13/13	M	4.0	IZINGA	Neg	
5644	9/13/13	F	65.0	BUNYANGONI	Neg	
5645	9/13/13	M	70.0	KYANYA	Neg	
5646	9/13/13	F	4.0	KISENDERA	Pos	
5647	9/13/13	F	60.0	KYANYA	Neg	
5648	9/13/13	M	5.0	MALIBA	Pos	
5649	9/13/13	F	32.0	KYARUGOMOKA	Neg	
5650	9/13/13	F	3.0	KYARUGOMOKA	Pos	
5651	9/13/13	F	17.0	KISAMBA	Neg	
5652	9/13/13	M	2.2	KISENDERA	Neg	
5653	9/13/13	F	22.0	KABUYIRI	Neg	
5654	9/13/13	M	5.0	KABUYIRI	Pos	
5655	9/13/13	M	1.5	MURAMBA	Neg	
5656	9/13/13	M	10.0	KAKINDO	Pos	Pos
5657	9/13/13	F	2.0	NDUGHUTU	Pos	
5658	9/13/13	F	27.0	NDUGHUTU	Neg	
5659	9/13/13	M	0.4	KYAHUNDE	Neg	
5660	9/13/13	M	30.0	MALIBA	Neg	
5661	9/13/13	M	9.0	KAKINDO	Neg	
5662	9/13/13	F	28.0	NDUGHUTU	Neg	
5663	9/13/13	F	9.0	KAKINDO	Pos	
5664	9/13/13	M	12.0	BUGOYE	Pos	
5665	9/13/13	M	12.0	IBANDA	Neg	
5666	9/13/13	F	32.0	MALIBA	Pos	
5667	9/13/13	F	16.0	BUGOYE	Neg	
5668	9/13/13	F	20.0	RWAKING	Neg	
5669	9/13/13	M	0.7	RWAKING	Neg	
5670	9/13/13	M	69.0	IHANI	Neg	
5671	9/13/13	M	3.0	BULYANDEKE	Pos	
5672	9/13/13	F	23.0	MALIBA	Neg	
5673	9/13/13	F	70.0	KANYAN	Neg	
5674	9/13/13	F	7.0	BUGOYE	Neg	
5675	9/13/13	F	0.7	BULYANDEKE	Neg	

5676	9/13/13	M	9.0	NDUGHUTU	Neg	
5677	9/13/13	F	1.0	RWAKING	Neg	
5678	9/13/13	F	49.0	IBANDA	Neg	
5679	9/13/13	F	1.0	BUGOYE	Neg	
5680	9/13/13	F	20.0	BUGOYE	Neg	
5681	9/13/13	M	16.0	IZINGA	Pos	
5682	9/13/13	F	70.0	BULYANDEKE	Neg	
5683	9/13/13	F	36.0	NYANGONGE	Neg	
5684	9/13/13	F	30.0	NYANGONGE	Neg	
5685	9/13/13	F	12.0	BUHUNGA	Neg	
5686	9/13/13	F	12.0	.		Pos
5687	9/13/13	F	12.0	BIKONE	Pos	
5688	9/13/13	F	8.0	MALIBA	Pos	
5689	9/13/13	M	40.0	MALIBA	Neg	
5690	9/13/13	F	9.0	BUHUNGA	Pos	
5691	9/13/13	F	2.0	BUHUNGA	Pos	
5692	9/13/13	F	3.0	NDUGHUTU	Pos	
5693	9/13/13	F	13.0	KANYAN	Pos	
5694	9/13/13	M	0.9	MALIBA	Neg	
5695	9/13/13	F	1.8	NDUGHUTU	Pos	
5696	9/13/13	F	13.0	RWAKING	Pos	
5697	9/13/13	M	1.1	KISANZI	Pos	
5698	9/13/13	M	4.0	MALIBA	Pos	
5699	9/13/13	M	13.0	BUGOYE	Pos	
5700	9/13/13	M	8.0	BUGOYE	Pos	
5701	9/13/13	F	2.0	MURAMBA	Pos	
5702	9/13/13	M	1.4	MALIBA	Pos	Pos
5703	9/17/13	F	40.0	BUGOYE	Neg	
5704	9/17/13	M	47.0	RWAKING	Neg	
5705	9/17/13	F	26.0	NDUGHUTU	Neg	
5706	9/17/13	F	30.0	NDUGHUTU	Neg	
5707	9/17/13	F	26.0	MURAMBA	Neg	
5708	9/17/13	M	47.0	BUNYANGONI	Neg	
5709	9/17/13	F	50.0	MURAMBA	Neg	
5710	9/17/13	F	16.0	MALIBA	Pos	
5711	9/17/13	F	5.0	MALIBA	Pos	
5712	9/17/13	F	28.0	BUGOYE	Pos	
5713	9/17/13	M	7.0	BUHUNGA	Pos	
5714	9/17/13	F	5.0	BUHUNGA	Pos	
5715	9/17/13	M	0.8	BUGOYE	Pos	
5716	9/17/13	F	36.0	NDUGHUTU	Pos	
5717	9/17/13	M	0.3	KANYAN	Neg	
5718	9/17/13	M	12.0	KYARUGOMOKA	Pos	

5719	9/17/13	F	7.0	BUGOYE	Pos
5720	9/17/13	F	30.0	NDUGHUTU	Pos
5721	9/17/13	M	13.0	BUGOYE	Neg
5722	9/17/13	M	7.0	KEMIHOKO	Neg
5723	9/17/13	M	1.0	NDUGHUTU	Pos
5724	9/18/13	F	1.3	MURAMBA	Neg
5725	9/18/13	F	30.0	KYARUGOMOKA	Neg
5726	9/18/13	F	25.0	BUZIBWERA	Pos
5727	9/18/13	M	0.2	NDUGHUTU	Pos
5728	9/18/13	F	18.0	NDUGHUTU	Pos
5729	9/18/13	F	0.6	NDUGHUTU	Neg
5730	9/18/13	M	9.0	RWAKING	Pos
5731	9/18/13	M	6.0	BIKONE	Neg
5732	9/18/13	F	35.0	NYAKARUNGU	Neg
5733	9/18/13	F	18.0	KIRONGO	Pos
5734	9/18/13	F	6.0	NDUGHUTU	Pos
5735	9/18/13	F	5.0	NDUGHUTU	Pos
5736	9/18/13	M	6.0	KIBOTA	Pos
5737	9/18/13	M	0.3	NDUGHUTU	Pos
5738	9/18/13	M	49.0	MUBUKU	Pos
5739	9/18/13	M	2.1	MURAMBA	Neg
5740	9/19/13	M	9.0	MALIBA	Pos
5741	9/19/13	F	29.0	MALIBA	Neg
5742	9/19/13	F	16.0	RWAKING	Pos
5743	9/19/13	F	33.0	BUGOYE	Neg
5744	9/19/13	F	25.0	MURAMBA	Pos
5745	9/19/13	M	18.0	KANYAN	Neg
5746	9/19/13	M	.	KIRULI	Pos
5747	9/19/13	M	30.0	KAKINDO	Neg
5748	9/19/13	F	24.0	KANYAN	Neg
5749	9/19/13	M	70.0	BUNYANGONI	Neg
5750	9/19/13	M	20.0	BUGOYE	Neg
5751	9/19/13	M	1.0	MALIBA	Neg
5752	9/19/13	F	0.1	BUNYANGONI	Neg
5753	9/19/13	F	37.0	KAKINDO	Neg
5754	9/19/13	M	11.0	MALIBA	Pos
5755	9/19/13	F	3.0	BUGOYE	Neg
5756	9/19/13	F	60.0	MAGHOMA	Pos
5757	9/19/13	F	18.0	KIKOKERA	Neg
5758	9/19/13	F	22.0	IHANI	Pos
5759	9/19/13	M	17.0	NDUGHUTU	Pos
5760	9/19/13	M	4.0	KANYAN	Neg
5761	9/19/13	M	5.0	KISANGA	Pos

5762	9/19/13	F	20.0	IBANDA	Neg
5763	9/19/13	F	30.0	BUGOYE	Pos
5764	9/19/13	F	0.0	KISANGA	Neg
5765	9/19/13	F	29.0	KISANGA	Pos
5766	9/20/13	F	43.0	KIKOKERA	Pos
5767	9/20/13	F	26.0	BUNYANGONI	Neg
5768	9/20/13	F	4.0	NDUGHUTU	Pos
5769	9/20/13	M	15.0	MURAMBA	Pos
5770	9/20/13	F	17.0	KIKOKERA	Neg
5771	9/20/13	F	17.0	NDUGHUTU	Pos
5772	9/20/13	F	24.0	BUGOYE	Neg
5773	9/20/13	F	29.0	MURAMBA	Neg
5774	9/20/13	F	2.0	RWAKING	Neg
5775	9/20/13	F	10.0	IZINGA	Neg
5776	9/20/13	M	6.0	IZINGA	Pos
5777	9/20/13	M	15.0	IZINGA	Pos
5778	9/20/13	M	20.0	RWAKING	Neg
5779	9/20/13	M	60.0	MALIBA	Pos
5780	9/20/13	M	5.2	BULINDUGURU	Neg
5781	9/25/13	F	25.0	BUGOYE	Neg
5782	9/25/13	F	21.0	IBANDA	Neg
5783	9/25/13	F	17.0	BUGOYE	Pos
5784	9/25/13	F	7.0	MUHAMBO	Pos
5785	9/25/13	M	3.0	KANYAN	Neg
5786	9/25/13	F	7.0	BUGOYE	Neg
5787	9/25/13	F	18.0	KYANYA	Neg
5788	9/25/13	M	23.0	MALIBA	Pos
5789	9/25/13	F	1.2	RWAKING	Pos
5790	9/25/13	F	70.0	NYAKALENGIJO	Neg
5791	9/25/13	F	13.0	IZINGA	Neg
5792	9/25/13	F	37.0	KANYAN	Neg
5793	9/25/13	M	3.3	BUGOYE	Pos
5794	9/25/13	F	17.0	NDUGHUTU	Pos
5795	9/25/13	F	8.0	BUGOYE	Pos
5796	9/25/13	F	26.0	KANYAN	Neg
5797	9/25/13	F	2.5	KIKOKERA	Pos
5798	9/25/13	F	13.0	MURAMBA	Pos
5799	9/25/13	F	40.0	MALIBA	Neg
5800	9/25/13	F	7.0	BUGOYE	Neg
5801	9/25/13	M	1.5	MUHAMBO	Neg
5802	9/25/13	M	46.0	MAGHOMA	Neg
5803	9/25/13	M	22.0	NDUGHUTU	Neg
5804	9/25/13	F	20.0	MAGHOMA	Neg

5805	9/25/13	F	17.0	KANYAN	Neg	
5806	9/25/13	M	40.0	BUGOYE	Neg	
5807	9/26/13	F	22.0	IZINGA	Pos	
5808	9/26/13	M	47.0	BUNYANGONI	Pos	
5809	9/26/13	F	10.0	BUGOYE	Pos	
5810	9/26/13	F	7.0	KANYAN	Pos	
5811	9/26/13	F	18.0	KIKOKERA	Neg	
5812	9/26/13	F	7.0	KANYAN	Pos	
5813	9/26/13	M	5.0	KYARUGOMOKA	Pos	
5814	9/26/13	M	12.0	MALIBA	Pos	
5815	9/26/13	F	8.0	MALIBA	Pos	
5816	9/26/13	M	21.0	NDUGHUTU	Neg	
5817	9/26/13	F	48.0	BUGOYE	Neg	
5818	9/26/13	F	28.0	MURAMBA	Pos	
5819	9/26/13	M	4.0	NDUGHUTU	Pos	
5820	9/26/13	M	13.0	BUGOYE	Neg	
5821	9/26/13	M	18.0	RWAKING	Pos	
5822	9/26/13	F	42.0	IHANI	Neg	
5823	9/26/13	M	6.0	IHANI	Neg	
5824	9/26/13	F	26.0	MURAMBA	Neg	
5825	9/26/13	M	17.0	BUNYANGONI	Neg	
5826	9/26/13	M	16.0	BUGOYE	Neg	
5827	9/26/13	M	2.0	KANYAN	Neg	
5828	9/26/13	F	1.2	MURAMBA	Neg	
5829	9/27/13	M	13.0	BUGOYE	Neg	
5830	9/27/13	F	2.5	MALIBA	Pos	
5831	9/27/13	M	7.0	NDUGHUTU	Pos	
5832	9/27/13	M	5.0	NDUGHUTU	Pos	
5833	9/27/13	F	24.0	NDUGHUTU	Neg	Neg
5834	9/27/13	M	12.0	KATOOKE	Pos	
5835	9/27/13	F	0.3	KATOOKE	Pos	
5836	9/27/13	F	2.0	KYARUGOMOKA		Pos
5837	9/27/13	M	5.0	NDUGHUTU	Neg	
5838	9/27/13	F	0.3	KIBIRIZI	Neg	
5839	9/27/13	M	46.0	BUZIBWERA	Neg	
5840	9/27/13	M	36.0	BUGOYE	Neg	
5841	9/27/13	M	26.0	BUGOYE	Neg	
5842	9/27/13	M	9.0	BUGOYE	Neg	
5843	9/27/13	M	1.0	KYARUGOMOKA	Pos	
5844	9/30/13	F	2.3	KYARUGOMOKA	Pos	
5845	9/30/13	F	20.0	BUGOYE	Pos	Neg
5846	9/30/13	F	0.3	MAGHOMA	Neg	Neg
5847	9/30/13	F	13.0	NYABUSISI	Neg	

5848	9/30/13	F	37.0	BUGOYE	Neg
5849	9/30/13	M	55.0	NYAKARUNGU	Pos
5850	9/30/13	F	3.0	KATUMBA	Pos
5851	9/30/13	M	12.0	BUZIBWERA	Neg
5852	9/30/13	F	20.0	NYAKARUNGU	Neg
5853	9/30/13	M	22.0	IBANDA	Neg
5854	9/30/13	M	8.0	NYAKARUNGU	Pos
5855	9/30/13	F	10.0	KANYAN	Pos
5856	9/30/13	M	5.0	RWAKING	Pos
5857	9/30/13	M	15.0	MALIBA	Pos
5858	9/30/13	M	27.0	MURAMBA	Neg
5859	9/30/13	F	16.0	NDUGHUTU	Pos
5860	9/30/13	F	20.0	BUGOYE	Neg
5861	9/30/13	M	42.0	MURAMBA	Neg
5862	9/30/13	M	3.8	BUGOYE	Pos
5863	9/30/13	M	1.6	BUGOYE	Pos
5864	9/30/13	M	50.0	IZINGA	Neg
5865	9/30/13	F	5.0	NDUGHUTU	Pos
5866	9/30/13	F	7.0	KAKINDO	Pos
5867	9/30/13	M	2.0	NDUGHUTU	Pos
5868	9/30/13	M	1.8	NDUGHUTU	Neg
5869	9/30/13	F	19.0	BUNYANGONI	Pos
5870	9/30/13	F	8.0	MALIBA	Neg
5871	9/30/13	F	9.0	KIRONGO	Neg
5872	9/30/13	M	30.0	KANYAN	Neg
5873	9/30/13	F	4.0	MURAMBA	Neg
5874	9/30/13	M	15.0	MALIBA	Neg
5875	9/30/13	M	24.0	MALIBA	Pos
5876	9/30/13	M	20.0	MURAMBA	Neg
5877	9/30/13	F	0.8	NYANGONGE	Neg
5878	9/30/13	F	46.0	NYAKARUNGU	Neg
5879	9/30/13	F	47.0	BUGOYE	Neg
5880	9/30/13	F	67.0	BUGOYE	Neg
5881	10/1/13	M	0.7	RWAKING	Neg
5882	10/1/13	F	1.8	BUGOYE	Neg
5883	10/1/13	F	26.0	BUGOYE	Neg
5884	10/1/13	F	30.0	BUGOYE	Neg
5885	10/1/13	F	18.0	BUZIBWERA	Neg
5886	10/1/13	M	3.8	BUGOYE	Neg
5887	10/1/13	F	3.1	MURAMBA	Neg
5888	10/1/13	M	0.0	KANYAN	Neg
5889	10/1/13	M	32.0	MUHAMBO	Neg
5890	10/1/13	F	0.4	KIGANDO	Neg

5891	10/1/13	M	20.0	MALIBA	Neg
5892	10/2/13	F	16.0	MURAMBA	Neg
5893	10/2/13	F	27.0	KANYAN	Pos
5894	10/2/13	F	17.0	MURAMBA	Neg
5895	10/2/13	M	0.4	MUBUKU	Pos
5896	10/2/13	F	15.0	RWAKING	Neg
5897	10/2/13	M	26.0	RWAKING	Pos
5898	10/2/13	F	25.0	MUBUKU	Pos
5899	10/2/13	F	30.0	KANYAN	Neg
5900	10/2/13	F	25.0	MURAMBA	Neg
5901	10/2/13	M	4.0	KANYAN	Pos
5902	10/2/13	F	38.0	BUGOYE	Neg
5903	10/2/13	M	0.7	KANYAN	Pos
5904	10/2/13	F	18.0	KANYAN	Neg
5905	10/2/13	F	20.0	BULYANDEKE	Pos
5906	10/2/13	F	3.0	KANYAN	Neg
5907	10/2/13	M	5.0	BUGOYE	Neg
5908	10/2/13	F	13.0	KYANYA	Pos
5909	10/2/13	M	12.0	KARUSULMBA	Neg
5910	10/2/13	F	8.0	RWAKING	Pos
5911	10/2/13	F	40.0	BUZIBWERA	Pos
5912	10/2/13	F	35.0	NDUGHUTU	Pos
5913	10/2/13	F	30.0	IBANDA	Neg
5914	10/2/13	M	8.0	RWAKING	Pos
5915	10/2/13	M	1.2	NYAKARUNGU	Pos
5916	10/2/13	F	1.1	MALIBA	Pos
5917	10/2/13	M	0.7	BUGOYE	Neg
5918	10/2/13	F	5.0	BUGOYE	Neg
5919	10/2/13	F	0.7	BUGOYE	Pos
5920	10/3/13	M	50.0	MALIBA	Neg
5921	10/3/13	M	68.0	KAKINDO	Neg
5922	10/3/13	F	41.0	MALIBA	Neg
5923	10/3/13	F	18.0	MURAMBA	Neg
5924	10/3/13	M	12.0	KIKOKERA	Pos
5925	10/3/13	F	2.2	RWAKING	Pos
5926	10/3/13	M	1.9	RWAKING	Pos
5927	10/3/13	F	39.0	RWAKING	Pos
5928	10/3/13	M	10.0	NDUGHUTU	Pos
5929	10/3/13	M	8.0	IZINGA	Neg
5930	10/3/13	F	1.1	MALIBA	Pos
5931	10/3/13	M	17.0	MALIBA	Neg
5932	10/3/13	F	12.0	BUGOYE	Pos
5933	10/3/13	F	16.0	MURAMBA	Pos

5934	10/3/13	M	7.0	NDUGHUTU	Pos	
5935	10/3/13	F	13.0	KANYAN	Pos	
5936	10/3/13	F	17.0	MALIBA	Pos	
5937	10/3/13	F	14.0	RWAKING	Neg	
5938	10/3/13	M	3.5	KATOOKE	Pos	
5939	10/3/13	F	5.5	MURAMBA	Neg	
5940	10/3/13	M	9.0	IZINGA	Neg	
5941	10/3/13	M	18.0	IZINGA	Pos	
5942	10/3/13	M	14.0	ISULE	Neg	
5943	10/3/13	F	9.0	MAGHOMA	Neg	
5944	10/3/13	M	4.0	KYAJUMA	Neg	
5945	10/3/13	M	39.0	KYAJUMA	Neg	
5946	10/3/13	M	44.0	MALIBA	Neg	
5947	10/3/13	M	29.0	MALIBA	Neg	
5948	10/3/13	M	6.0	MUWELO	Neg	
5949	10/3/13	M	3.0	BUGOYE	Neg	
5950	10/3/13	F	25.0	BUGOYE	Neg	
5951	10/3/13	F	10.0	BUNYANGONI	Neg	
5952	10/3/13	F	13.0	NYAKALENGIJO	Neg	
5953	10/3/13	F	4.0	MALIBA	Neg	
5954	10/3/13	F	0.8	BUGOYE	Neg	
5955	10/3/13	M	0.8	MURAMBA	Neg	
5956	10/3/13	M	0.8	MURAMBA	Neg	
5957	10/3/13	F	0.1	MALIBA	Neg	
5958	10/3/13	M	2.0	IZINGA	Pos	
5959	10/3/13	M	1.1	NDUGHUTU	Neg	
5960	10/3/13	F	23.0	NDUGHUTU	Neg	
5961	10/4/13	M	0.8	BUNYANGONI	Neg	
5962	10/4/13	M	10.0	MALIBA	Neg	
5963	10/4/13	M	9.0	IBANDA	Pos	
5964	10/4/13	M	19.0	BUNYANGONI	Neg	
5965	10/4/13	M	17.0	IBANDA	Neg	
5966	10/4/13	F	10.0	RWAKING	Neg	
5967	10/4/13	F	16.0	BUGOYE	Neg	
5968	10/4/13	M	4.5	MURAMBA	Neg	
5969	10/4/13	M	9.0	BUGOYE	Neg	
5970	10/4/13	F	1.0	KIHINDI	Neg	
5971	10/4/13	F	0.7	MURAMBA	Neg	
5972	10/4/13	F	18.0	MURAMBA	Pos	
5973	10/4/13	M	19.0	BUGOYE	Neg	
5974	10/4/13	F	26.0	MALIBA	Neg	
5975	10/4/13	M	1.2	KIBIRIZI	Neg	
5976	10/4/13	F	28.0	KYARUGOMOKA		Neg

5977	10/7/13	F	32.0	BUGOYE		Neg
5978	10/7/13	F	26.0	NYAKARUNGU	Neg	
5979	10/7/13	F	23.0	RWAKING	Neg	
5980	10/7/13	M	20.0	BUGOYE	Pos	
5981	10/7/13	M	19.0	NYAMBUKU		Pos
5982	10/7/13	M	23.0	BUGOYE	Pos	
5983	10/7/13	M	3.0	NDUGHUTU	Neg	
5984	10/7/13	F	4.0	MALIBA	Neg	
5985	10/7/13	F	3.0	KIKOKERA	Neg	
5986	10/7/13	F	27.0	RWAKING	Neg	
5987	10/7/13	M	1.3	BUNYANGONI	Neg	
5988	10/7/13	M	0.5	NDUGHUTU	Pos	
5989	10/7/13	F	5.0	RUBONI	Pos	
5990	10/7/13	M	4.5	RUBONI	Pos	
5991	10/7/13	F	8.5	NDUGHUTU	Pos	
5992	10/7/13	F	5.5	NDUGHUTU	Neg	
5993	10/7/13	M	10.0	MALIBA	Pos	
5994	10/7/13	M	5.0	KYARUGOMOKA	Neg	
5995	10/7/13	F	10.0	KATUMBA	Pos	
5996	10/7/13	F	19.0	BUGOYE	Pos	
5997	10/7/13	F	2.8	RWAKING	Pos	
5998	10/7/13	M	17.0	BUGOYE	Pos	
5999	10/7/13	M	1.0	BUGOYE	Neg	
6000	10/7/13	F	3.0	BUNYANGONI	Pos	
6001	10/7/13	M	11.0	MURAMBA	Neg	
6002	10/7/13	F	25.0	MALIBA	Neg	
6003	10/7/13	M	18.0	IBANDA	Pos	
6004	10/7/13	F	0.5	MURAMBA	Neg	
6005	10/7/13	F	21.0	BIKONE	Neg	
6006	10/7/13	M	3.0	KIRULI	Neg	
6007	10/7/13	M	10.0	BULYANDEKE	Pos	
6008	10/7/13	F	16.0	KANYAN	Pos	
6009	10/7/13	M	5.0	BULYANDEKE	Pos	
6010	10/7/13	F	5.0	NDUGHUTU	Neg	
6011	10/7/13	F	4.5	IZINGA	Pos	
6012	10/7/13	M	21.0	BUHUNGA	Neg	
6013	10/7/13	M	48.0	IZINGA	Neg	
6014	10/7/13	F	25.0	MURAMBA	Neg	
6015	10/7/13	F	25.0	NDUGHUTU	Neg	
6016	10/7/13	F	3.2	RWAKING	Neg	
6017	10/7/13	F	24.0	KANYAN	Pos	
6018	10/7/13	M	8.0	IZINGA	Pos	
6019	10/8/13	F	5.5	BUGOYE	Neg	

6020	10/8/13	F	5.0	BUGOYE	Neg
6021	10/8/13	F	17.0	KATOOKE	Neg
6022	10/8/13	F	35.0	BUGOYE	Neg
6023	10/8/13	M	0.5	MURAMBA	Neg
6024	10/8/13	F	0.2	MURAMBA	Neg
6025	10/8/13	M	80.0	KAKINDO	Neg
6026	10/8/13	F	21.0	KYARUGOMOKA	Neg
6027	10/8/13	F	4.0	MUHAMBO	Neg
6028	10/8/13	F	23.0	NDUGHUTU	Neg
6029	10/8/13	F	1.0	NYAKABUGHA	Neg
6030	10/8/13	F	10.0	IZINGA	Neg
6031	10/8/13	M	3.0	MURAMBA	Neg
6032	10/8/13	F	20.0	MURAMBA	Neg
6033	10/8/13	F	0.7	RWAKING	Neg
6034	10/8/13	F	0.5	KANYAN	Neg
6035	10/8/13	F	1.2	MAGHOMA	Neg
6036	10/8/13	M	3.0	MURAMBA	Neg
6037	10/8/13	F	32.0	MASAKA	Neg
6038	10/8/13	F	17.0	BUGOYE	Pos
6039	10/8/13	F	7.0	MURAMBA	Pos
6040	10/8/13	F	12.0	MURAMBA	Pos
6041	10/8/13	F	45.0	MURAMBA	Neg
6042	10/8/13	M	2.0	BUGOYE	Neg
6043	10/8/13	M	13.0	KAKINDO	Pos
6044	10/8/13	M	10.0	KYARUGOMOKA	Pos
6045	10/8/13	F	7.0	NDUGHUTU	Neg
6046	10/8/13	F	40.0	MALIBA	Neg
6047	10/8/13	M	16.0	NDUGHUTU	Pos
6048	10/8/13	F	1.0	MALIBA	Pos
6049	10/8/13	M	4.0	IBANDA	Neg
6050	10/8/13	M	6.0	IBANDA	Neg
6051	10/8/13	F	28.0	BUGOYE	Pos
6052	10/8/13	F	42.0	MURAMBA	Neg
6053	10/8/13	F	0.4	KIRONGO	Neg
6054	10/8/13	F	25.0	KYANYA	Neg
6055	10/8/13	M	0.3	NDUGHUTU	Neg
6056	10/10/13	M	2.0	KANYAN	Neg
6057	10/10/13	F	14.0	MURAMBA	Neg
6058	10/10/13	F	19.0	KIGORO	Neg
6059	10/10/13	M	18.0	IZINGA	Pos
6060	10/10/13	F	10.0	BUGOYE	Neg
6061	10/10/13	F	16.0	IZINGA	Neg
6062	10/10/13	F	23.0	KATOOKE	Neg

6063	10/10/13	M	0.5	KANYAN	Neg	
6064	10/10/13	M	1.5	KANYAN	Neg	
6065	10/10/13	F	17.0	KANYAN	Neg	
6066	10/10/13	M	19.0	MURAMBA	Pos	
6067	10/10/13	M	15.0	KANYAN	Pos	
6068	10/10/13	F	12.0	BUZIBWERA	Neg	
6069	10/10/13	M	0.5	BUGOYE	Neg	
6070	10/10/13	F	29.0	BUGOYE	Neg	
6071	10/10/13	F	17.0	MAPATI	Neg	
6072	10/10/13	M	10.0	MURAMBA	Neg	
6073	10/10/13	F	30.0	NYAKARUNGU	Neg	
6074	10/10/13	M	3.0	NDUGHUTU	Neg	
6075	10/10/13	M	12.0	BUZIBWERA	Pos	
6076	10/10/13	M	0.8	KIRULI	Pos	
6077	10/10/13	F	13.0	NDUGHUTU	Pos	
6078	10/10/13	F	15.0	MALIBA	Neg	
6079	10/10/13	M	8.0	IZINGA	Pos	
6080	10/10/13	F	17.0	KYANYA	Neg	
6081	10/10/13	M	22.0	KATOOKE	Neg	
6082	10/10/13	F	26.0	KISANGA	Neg	
6083	10/10/13	F	35.0	IBANDA	Neg	
6084	10/10/13	F	27.0	NYAMBUKU	Neg	
6085	10/10/13	F	10.0	MALIBA	Neg	
6086	10/10/13	F	24.0	KATUMBA	Neg	
6087	10/10/13	M	1.0	MURAMBA	Neg	
6088	10/11/13	M	80.0	BUGOYE	Neg	
6089	10/11/13	M	21.0	BUGOYE	Neg	Neg
6090	10/11/13	F	18.0	KYARUGOMOKA	Pos	
6091	10/11/13	F	15.0	BUGOYE	Neg	
6092	10/11/13	F	10.0	BUGOYE	Pos	
6093	10/11/13	M	5.0	IZINGA	Pos	
6094	10/11/13	M	0.8	IHANI	Pos	Neg
6095	10/11/13	F	20.0	MURAMBA	Pos	
6096	10/11/13	M	1.0	MURAMBA	Neg	
6097	10/11/13	F	66.0	BUNYANGONI	Neg	
6098	10/11/13	F	7.0	KISANGA	Pos	
6099	10/11/13	M	1.8	IZINGA	Pos	
6100	10/11/13	M	0.8	NDUGHUTU	Neg	
6101	10/11/13	M	10.0	KISAMBA	Neg	
6102	10/11/13	F	2.0	RWAKING	Neg	Neg
6103	10/11/13	F	7.0	IZINGA	Pos	
6104	10/11/13	M	12.0	KYARUGOMOKA	Pos	
6105	10/11/13	F	50.0	KYARUGOMOKA		Neg

6106	10/11/13	M	30.0	KISOJO	Neg	
6107	10/11/13	M	67.0	MURAMBA	Pos	
6108	10/11/13	M	5.0	NDUGHUTU	Pos	
6109	10/11/13	F	2.0	MAGHOMA	Neg	
6110	10/11/13	F	12.0	NDUGHUTU	Pos	
6111	10/11/13	M	0.8	MURAMBA	Pos	
6112	10/11/13	M	12.0	KANYAN	Neg	
6113	10/11/13	M	5.0	NDUGHUTU	Pos	
6114	10/11/13	M	7.0	NDUGHUTU	Neg	
6115	10/14/13	M	2.2	BUGOYE	Pos	
6116	10/14/13	F	0.8	MALIBA	Neg	
6117	10/14/13	F	33.0	MALIBA	Neg	
6118	10/14/13	F	0.6	MALIBA	Neg	
6119	10/14/13	F	33.0	BUGOYE	Neg	
6120	10/14/13	F	10.0	BUGOYE	Pos	
6121	10/14/13	F	22.0	NDUGHUTU	Neg	
6122	10/14/13	F	14.0	MALIBA	Neg	Neg
6123	10/14/13	M	3.5	MURAMBA	Pos	
6124	10/14/13	F	12.0	BUGOYE	Neg	
6125	10/14/13	F	0.8	MALIBA	Neg	
6126	10/14/13	F	38.0	MUHAMBO	Neg	
6127	10/14/13	F	0.8	IZINGA	Pos	
6128	10/14/13	F	22.0	IZINGA	Neg	
6129	10/14/13	M	0.3	IZINGA	Neg	
6130	10/14/13	M	9.0	MURAMBA	Pos	
6131	10/14/13	M	3.8	MALIBA	Pos	
6132	10/14/13	F	29.0	MALIBA	Pos	
6133	10/14/13	F	12.0	BULYANDEKE	Neg	
6134	10/14/13	F	10.0	MURAMBA	Neg	
6135	10/14/13	F	7.0	BUGOYE	Pos	
6136	10/14/13	F	30.0	BUGOYE	Neg	
6137	10/14/13	F	25.0	BUNYANGONI	Neg	
6138	10/14/13	F	2.3	BUNYANGONI	Pos	
6139	10/14/13	F	20.0	NYAKARUNGU	Pos	
6140	10/14/13	F	38.0	BUGOYE	Pos	
6141	10/14/13	F	75.0	IBANDA	Neg	
6142	10/14/13	F	18.0	NDUGHUTU	Neg	
6143	10/14/13	F	17.0	NDUGHUTU	Pos	
6144	10/14/13	F	21.0	KANYAN	Neg	
6145	10/14/13	M	4.7	MALIBA	Pos	
6146	10/14/13	M	10.0	IZINGA	Pos	
6147	10/14/13	M	46.0	BUZIBWERA	Neg	
6148	10/14/13	M	4.0	IZINGA	Pos	

6149	10/14/13	M	0.5	MAGHOMA	Neg	
6150	10/14/13	F	3.0	IZINGA	Neg	
6151	10/14/13	F	15.0	RWAKING	Neg	
6152	10/14/13	F	56.0	BUNYANGONI	Neg	
6153	10/14/13	F	18.0	BUGOYE	Neg	
6154	10/14/13	F	20.0	BUGOYE	Pos	
6155	10/14/13	M	2.5	KATOOKE	Neg	
6156	10/14/13	F	14.0	NDUGHUTU	Neg	
6157	10/14/13	M	18.0	IZINGA	Pos	
6158	10/14/13	M	17.0	KATOOKE	Pos	
6159	10/14/13	F	9.0	KIBOTA	Neg	
6160	10/14/13	F	13.0	KAKINDO	Neg	
6161	10/14/13	F	10.0	MURAMBA	Neg	
6162	10/14/13	F	37.0	KIRONGO	Neg	
6163	10/14/13	F	12.0	MURAMBA	Neg	
6164	10/14/13	F	11.0	MURAMBA	Neg	
6165	10/14/13	F	24.0	KATUMBA	Neg	
6166	10/14/13	F	2.0	MURAMBA	Neg	
6167	10/14/13	M	3.0	MURAMBA	Pos	
6168	10/14/13	M	7.0	MURAMBA	Neg	
6169	10/14/13	M	9.0	MURAMBA	Pos	
6170	10/14/13	F	16.0	KIKOKERA	Neg	
6171	10/14/13	F	60.0	MAGHOMA	Neg	
6172	10/14/13	F	12.0	KAKINDO	Pos	
6173	10/14/13	F	11.0	IZINGA	Pos	
6174	10/14/13	M	2.0	.	Neg	
6175	10/14/13	F	10.0	MURAMBA	Neg	
6176	10/14/13	F	50.0	KYANYA	Neg	
6177	10/14/13	F	3.5	IHANI	Neg	
6178	10/14/13	F	50.0	IHANI	Neg	
6179	10/14/13	F	15.0	MALIBA	Neg	
6180	10/14/13	M	10.0	MURAMBA	Neg	
6181	10/14/13	F	36.0	KAKINDO	Pos	
6182	10/14/13	F	23.0	MURAMBA	Neg	
6183	10/14/13	M	4.0	BUGOYE	Neg	
6184	10/15/13	F	27.0	KIRULI	Pos	Pos
6185	10/15/13	F	1.2	RWAKING	Pos	Pos
6186	10/15/13	F	18.0	BUNYANGONI	Neg	
6187	10/15/13	M	36.0	KANYAN	Neg	
6188	10/15/13	M	2.5	RWAKING	Pos	
6189	10/15/13	M	7.0	IZINGA	Pos	
6190	10/15/13	F	19.0	MURAMBA	Neg	
6191	10/15/13	F	11.0	BUGOYE	Neg	

6192	10/15/13	F	10.0	BUGOYE	Neg
6193	10/15/13	F	72.0	BUNYANGONI	Neg
6194	10/15/13	M	5.0	NDUGHUTU	Pos
6195	10/15/13	M	2.0	NDUGHUTU	Pos
6196	10/15/13	M	0.3	KANYAN	Neg
6197	10/15/13	F	30.0	BUGOYE	Neg
6198	10/16/13	M	13.0	IBANDA	Pos
6199	10/16/13	F	13.0	BUGOYE	Neg
6200	10/16/13	F	55.0	MURAMBA	Neg
6201	10/16/13	M	13.0	KANYAN	Pos
6202	10/16/13	M	8.0	BUGOYE	Neg
6203	10/16/13	F	6.0	MALIBA	Pos
6204	10/16/13	F	10.0	NDUGHUTU	Neg
6205	10/16/13	F	11.0	NDUGHUTU	Neg
6206	10/16/13	F	20.0	BUGOYE	Neg
6207	10/16/13	M	5.0	MURAMBA	Pos
6208	10/16/13	F	5.0	NDUGHUTU	Pos
6209	10/16/13	F	20.0	RWAKING	Neg
6210	10/16/13	F	60.0	IBANDA	Neg
6211	10/16/13	M	12.0	BUGOYE	Neg
6212	10/16/13	M	30.0	MAGHOMA	Neg
6213	10/16/13	F	13.0	BUGOYE	Pos
6214	10/16/13	F	10.0	NDUGHUTU	Neg
6215	10/16/13	F	23.0	MURAMBA	Pos
6216	10/16/13	F	22.0	KAKINDO	Neg
6217	10/16/13	F	0.3	NDUGHUTU	Neg
6218	10/16/13	M	8.0	BUGOYE	Neg
6219	10/16/13	F	38.0	MALIBA	Neg
6220	10/16/13	F	13.0	RWAKING	Neg
6221	10/16/13	F	13.0	BUGOYE	Neg
6222	10/16/13	F	13.0	BUGOYE	Neg
6223	10/16/13	F	9.0	BUGOYE	Neg
6224	10/16/13	F	5.0	MALIBA	Pos
6225	10/16/13	F	1.7	MALIBA	Neg
6226	10/16/13	F	3.0	BUNYANGONI	Pos
6227	10/16/13	M	1.2	BUNYANGONI	Neg
6228	10/16/13	F	25.0	MALIBA	Pos
6229	10/16/13	M	0.3	MALIBA	Pos
6230	10/16/13	F	55.0	MALIBA	Neg
6231	10/16/13	F	56.0	BUNYANGONI	Pos
6232	10/16/13	M	2.5	BUNYANGONI	Pos
6233	10/16/13	F	31.0	BUNYANGONI	Pos
6234	10/16/13	F	38.0	MALIBA	Neg

6235	10/16/13	M	1.2	KYANDALE	Neg	
6236	10/16/13	F	45.0	RWAKING	Neg	
6237	10/16/13	F	22.0	KYARUGOMOKA	Neg	
6238	10/18/13	M	7.0	MURAMBA	Neg	
6239	10/18/13	F	56.0	NDUGHUTU	Neg	
6240	10/18/13	F	54.0	KYARUGOMOKA	Pos	
6241	10/18/13	F	50.0	MURAMBA	Pos	
6242	10/18/13	F	31.0	IBANDA	Neg	
6243	10/18/13	F	4.0	MURAMBA	Pos	
6244	10/18/13	M	6.0	MURAMBA	Pos	
6245	10/18/13	M	9.0	KANYAN	Neg	
6246	10/18/13	M	7.0	MURAMBA	Neg	
6247	10/18/13	F	19.0	NDUGHUTU	Neg	
6248	10/18/13	F	10.0	MALIBA	Neg	
6249	10/18/13	F	50.0	MURAMBA	Pos	
6250	10/18/13	M	40.0	BUGOYE	Neg	
6251	10/18/13	M	72.0	IZINGA	Neg	
6252	10/18/13	M	38.0	BUGOYE	Neg	
6253	10/18/13	F	30.0	KANYAN	Neg	
6254	10/18/13	F	62.0	KIKOKERA	Neg	
6255	10/18/13	F	48.0	NDUGHUTU	Neg	
6256	10/18/13	F	0.8	MURAMBA	Neg	
6257	10/18/13	F	10.0	NDUGHUTU	Pos	
6258	10/18/13	F	1.8	NDUGHUTU	Neg	
6259	10/18/13	F	24.0	KAKINDO	Pos	
6260	10/18/13	M	28.0	MURAMBA	Neg	
6261	10/18/13	F	5.0	KIBOTA	Neg	
6262	10/18/13	F	10.0	MURAMBA	Pos	
6263	10/18/13	F	0.2	BUNYANGONI	Neg	
6264	10/18/13	M	0.6	KILEMBE	Neg	
6265	10/22/13	F	19.0	BUGOYE	Pos	
6266	10/22/13	M	9.0	KAKINDO	Pos	
6267	10/22/13	M	2.0	KIRONGO	Pos	
6268	10/22/13	F	27.0	RWAKING	Neg	
6269	10/22/13	F	62.0	KANYAN	Neg	
6270	10/22/13	F	17.0	KIKOKERA	Neg	
6271	10/22/13	F	20.0	BUGOYE	Neg	
6272	10/22/13	F	0.5	MUHAMBO	Neg	
6273	10/22/13	F	29.0	BUGOYE	Neg	
6274	10/22/13	F	29.0	NDUGHUTU	Neg	
6275	10/22/13	M	18.0	BUGOYE	Neg	
6276	10/23/13	M	4.0	NDUGHUTU	Pos	Pos
6277	10/23/13	F	70.0	KYANYA	Neg	

6278	10/23/13	F	27.0	BUGOYE	Neg	
6279	10/23/13	F	45.0	NDUGHUTU	Neg	
6280	10/23/13	F	14.0	MALIBA	Pos	
6281	10/23/13	M	23.0	KIKOKERA	Pos	
6282	10/23/13	M	1.3	IBANDA	Pos	
6283	10/23/13	M	4.0	IBANDA	Neg	
6284	10/23/13	F	40.0	MURAMBA	Pos	
6285	10/23/13	F	24.0	IBANDA	Neg	
6286	10/23/13	F	28.0	IBANDA	Pos	
6287	10/23/13	F	20.0	MURAMBA	Neg	
6288	10/23/13	F	23.0	MURAMBA	Pos	
6289	10/23/13	F	5.0	RWAKING	Pos	
6290	10/23/13	F	33.0	KANYAN	Neg	
6291	10/23/13	F	3.5	MURAMBA	Pos	
6292	10/23/13	F	13.0	MURAMBA	Pos	
6293	10/23/13	F	21.0	MALIBA	Neg	
6294	10/23/13	F	21.0	KISENDERA	Neg	
6295	10/23/13	M	1.5	IZINGA	Neg	
6296	10/23/13	M	16.0	NDUGHUTU	Pos	
6297	10/23/13	F	1.9	RWAKING	Neg	Neg
6298	10/23/13	M	2.0	KYARUGOMOKA	Neg	
6299	10/23/13	F	4.0	KATUMBA	Pos	
6300	10/23/13	F	7.0	KANYAN	Pos	
6301	10/23/13	M	43.0	KANYAN	Neg	
6302	10/24/13	F	35.0	KANYAN	Neg	
6303	10/24/13	M	13.0	RWAKING	Neg	Neg
6304	10/24/13	F	20.0	MURAMBA	Neg	
6305	10/24/13	M	20.0	KIHINDI	Neg	
6306	10/24/13	M	0.9	KYARUGOMOKA	Neg	
6307	10/24/13	M	23.0	IZINGA	Neg	
6308	10/24/13	F	14.0	KYARUGOMOKA	Pos	
6309	10/24/13	F	72.0	BUNYANGONI	Neg	
6310	10/24/13	F	29.0	BUGOYE	Pos	Pos
6311	10/24/13	F	50.0	KINYANGONGE	Neg	
6312	10/24/13	M	3.0	IZINGA	Pos	
6313	10/24/13	F	45.0	IZINGA	Neg	
6314	10/24/13	F	26.0	BUGOYE	Neg	
6315	10/25/13	F	7.0	KANYAN	Neg	
6316	10/25/13	F	24.0	KANYAN	Pos	
6317	10/25/13	M	10.0	IZINGA	Pos	
6318	10/25/13	M	2.7	KANYAN	Neg	
6319	10/25/13	M	59.0	IZINGA	Neg	
6320	10/25/13	M	1.3	MURAMBA	Pos	

6321	10/25/13	F	30.0	RWAKING	Neg	
6322	10/28/13	F	11.0	RWAKING	Neg	
6323	10/28/13	F	14.0	BUNYANGONI	Neg	
6324	10/28/13	F	40.0	IBANDA	Neg	
6325	10/28/13	M	0.4	KANYAN	Neg	
6326	10/28/13	M	0.4	RWAKING	Neg	
6327	10/28/13	F	27.0	NDUGHUTU	Neg	
6328	10/28/13	F	20.0	MURAMBA	Neg	
6329	10/28/13	M	22.0	BUGOYE	Neg	
6330	10/28/13	M	11.0	KANYAN	Pos	
6331	10/28/13	F	1.5	BUNYANGONI	Neg	
6332	10/28/13	F	37.0	BUNYANGONI	Neg	
6333	10/28/13	F	25.0	KYAMBUGHA	Neg	
6334	10/28/13	F	24.0	BUGOYE	Neg	
6335	10/28/13	M	0.8	RWAKING	Neg	
6336	10/28/13	F	24.0	KANYAN	Pos	Neg
6337	10/28/13	F	40.0	KISAMBA	Neg	
6338	10/28/13	F	50.0	NDUGHUTU	Pos	Pos
6339	10/28/13	F	25.0	KISAMBA	Neg	
6340	10/28/13	F	18.0	BULYANDEKE	Neg	
6341	10/28/13	M	14.0	KANYAN	Neg	
6342	10/28/13	F	50.0	KANYAN	Pos	
6343	10/28/13	F	7.0	MALIBA	Neg	
6344	10/28/13	F	12.0	BUGOYE	Pos	
6345	10/28/13	F	13.0	KYARUGOMOKA	Neg	
6346	10/28/13	F	19.0	KANYAN	Neg	
6347	10/28/13	F	20.0	MURAMBA	Neg	
6348	10/28/13	F	32.0	BUNYANGONI	Neg	
6349	10/28/13	M	10.0	KANYAN	Pos	
6350	10/28/13	M	20.0	KANYAN	Neg	
6351	10/28/13	F	14.0	RWAKING	Neg	
6352	10/28/13	F	19.0	KANYAN	Neg	
6353	10/28/13	F	39.0	MUMWERNGO	Neg	
6354	10/28/13	F	1.8	MALIBA	Neg	
6355	10/28/13	M	42.0	MUHAMBO	Neg	
6356	10/28/13	F	35.0	MALIBA	Neg	
6357	10/28/13	F	11.0	MURAMBA	Neg	
6358	10/28/13	F	10.0	BUGOYE	Pos	
6359	10/28/13	M	2.0	IZINGA	Pos	
6360	10/28/13	F	6.0	MUBUKU	Pos	
6361	10/28/13	M	2.0	BUGOYE	Neg	
6362	10/28/13	M	1.4	MURAMBA	Neg	
6363	10/28/13	F	1.5	RWAKING	Neg	

6364	10/28/13	F	24.0	RWAKING	Neg	
6365	10/28/13	F	40.0	BUGOYE	Neg	
6366	10/28/13	M	34.0	BUGOYE	Pos	
6367	10/28/13	F	40.0	KANGWANJI	Pos	
6368	10/28/13	F	1.1	IZINGA	Pos	
6369	10/28/13	M	1.0	MURAMBA	Neg	
6370	10/28/13	F	21.0	MURAMBA	Neg	
6371	10/28/13	F	5.0	BUZIBWERA	Pos	
6372	10/28/13	F	7.0	IHANI	Pos	
6373	10/28/13	M	45.0	NYABISUSI	Neg	
6374	10/28/13	F	50.0	MURAMBA	Pos	
6375	10/28/13	F	22.0	MURAMBA	Neg	
6376	10/28/13	F	3.0	MALIBA	Pos	
6377	10/28/13	F	0.8	MALIBA	Pos	
6378	10/28/13	F	20.0	MURAMBA	Neg	
6379	10/28/13	F	2.8	MURAMBA	Neg	
6380	10/28/13	F	0.3	IHANI	Neg	
6381	10/28/13	F	35.0	KISAMBA	Neg	
6382	10/28/13	F	40.0	BUZIBWERA	Pos	
6383	10/28/13	F	11.0	MURAMBA	Neg	
6384	10/28/13	F	0.1	NDUGHUTU	Neg	
6385	10/28/13	F	7.0	IHANI	Pos	
6386	10/28/13	F	21.0	KAKINDO	Neg	
6387	10/29/13	F	4.0	RWAKING	Neg	
6388	10/29/13	F	49.0	NDUGHUTU	Neg	
6389	10/29/13	F	29.0	KANYAN	Pos	
6390	10/29/13	M	40.0	RWAKING	Neg	
6391	10/29/13	M	50.0	IBANDA	Neg	Neg
6392	10/29/13	M	2.0	BUGOYE	Pos	
6393	10/29/13	F	1.2	RWAKING	Neg	
6394	10/29/13	F	12.0	IZINGA	Neg	
6395	10/29/13	F	3.0	MURAMBA	Pos	
6396	10/29/13	F	32.0	BULINDUGURU	Neg	
6397	10/29/13	F	1.2	MURAMBA	Neg	
6398	10/29/13	F	42.0	RWAKING	Neg	
6399	10/29/13	F	29.0	MAGHOMA	Neg	
6400	10/29/13	F	17.0	BUGOYE	Neg	
6401	10/29/13	F	0.1	NDUGHUTU	Neg	
6402	10/29/13	F	24.0	NDUGHUTU	Neg	
6403	10/29/13	F	42.0	BIKONE	Pos	
6404	10/29/13	F	90.0	KISAMBA	Neg	
6405	10/29/13	M	11.0	MURAMBA	Neg	
6406	10/29/13	F	19.0	KISANGA	Neg	

6407	10/29/13	M	17.0	KANYAN	Neg	
6408	10/29/13	F	39.0	NDUGHUTU	Neg	
6409	10/29/13	F	40.0	NDUGHUTU	Pos	
6410	10/29/13	F	7.0	NDUGHUTU	Neg	
6411	10/29/13	F	0.6	KIBOTA	Neg	
6412	10/29/13	F	33.0	KIBOTA	Neg	
6413	10/29/13	M	1.3	KIRONGO	Neg	
6414	10/29/13	F	40.0	KYANYA	Neg	
6415	10/29/13	F	35.0	KISENDERA	Neg	
6416	10/29/13	F	5.0	MURAMBA	Pos	
6417	10/29/13	F	3.0	RWAKING	Neg	
6418	10/29/13	M	5.0	RWAKING	Neg	
6419	10/29/13	F	12.0	BUGOYE	Neg	
6420	10/29/13	F	28.0	KANYAN	Neg	
6421	10/29/13	F	40.0	KYANYA	Neg	
6422	10/29/13	F	35.0	MALIBA	Neg	
6423	10/29/13	M	1.2	MURAMBA	Pos	
6424	10/29/13	F	18.0	BUGOYE	Neg	
6425	10/29/13	M	3.0	RWAKING	Pos	
6426	10/29/13	F	78.0	RWAKING	Neg	
6427	10/29/13	M	1.3	BUGOYE	Neg	
6428	10/29/13	F	21.0	BUNYANGONI	Neg	
6429	10/29/13	F	70.0	NDUGHUTU	Neg	
6430	10/29/13	M	1.2	MURAMBA	Neg	
6431	10/29/13	F	20.0	MUHAMBO	Neg	
6432	10/29/13	F	9.0	IZINGA	Pos	
6433	10/29/13	M	1.5	KISANGA	Pos	
6434	10/29/13	M	0.1	BUNYANGONI	Neg	
6435	10/29/13	F	1.0	IZINGA	Pos	
6436	10/30/13	F	6.0	KATOOKE	Pos	
6437	10/30/13	F	7.0	BUGOYE	Pos	
6438	10/30/13	M	9.0	MALIBA	Pos	
6439	10/30/13	M	3.0	KAKINDO	Neg	
6440	10/30/13	M	57.0	KAKINDO	Neg	
6441	10/30/13	F	24.0	BUGOYE	Neg	Neg
6442	10/30/13	F	18.0	KANYAN	Neg	
6443	10/30/13	F	40.0	KYANYA	Neg	
6444	10/30/13	F	16.0	KIKOKERA	Neg	
6445	10/30/13	F	51.0	IZINGA	Neg	
6446	10/30/13	M	15.0	MURAMBA	Neg	
6447	10/30/13	F	10.0	BUNYANGONI	Pos	
6448	10/30/13	M	11.0	MURAMBA	Pos	
6449	10/30/13	M	80.0	KAKINDO	Neg	

6450	10/30/13	F	39.0	MALIBA	Pos	
6451	10/30/13	F	30.0	KYANYA	Neg	
6452	10/30/13	F	14.0	MURAMBA	Neg	
6453	10/30/13	M	5.0	MURAMBA	Pos	
6454	10/30/13	M	50.0	MURAMBA	Neg	
6455	10/30/13	F	66.0	KISENDERA	Neg	
6456	10/30/13	M	2.0	KATOOKE	Neg	
6457	10/30/13	M	16.0	BUGOYE	Neg	
6458	10/30/13	M	18.0	BUGOYE	Neg	
6459	10/30/13	M	2.0	MURAMBA	Neg	
6460	10/30/13	F	57.0	KIKOKERA	Neg	
6461	10/30/13	F	18.0	MALIBA	Pos	
6462	10/30/13	M	15.0	KYARUGOMOKA	Pos	
6463	10/30/13	F	4.0	IZINGA	Pos	
6464	10/30/13	F	4.0	IZINGA	Neg	
6465	10/30/13	F	56.0	IZINGA	Neg	
6466	10/30/13	F	11.0	KIKOKERA	Pos	
6467	10/30/13	F	37.0	KYANYA	Neg	
6468	10/30/13	M	13.0	BUGOYE	Pos	
6469	10/30/13	F	40.0	KANYAN	Neg	
6470	10/31/13	M	21.0	KANYAN	Pos	
6471	10/31/13	F	17.0	KANYAN		Pos
6472	10/31/13	F	15.0	RWAKING	Neg	
6473	10/31/13	F	8.0	RWAKING		Pos
6474	10/31/13	F	36.0	MURAMBA	Pos	
6475	10/31/13	F	59.0	KATOOKE	Neg	
6476	10/31/13	M	23.0	IBANDA	Neg	
6477	10/31/13	F	2.3	KEMIHOKO	Neg	Neg
6478	10/31/13	M	1.3	NDUGHUTU	Neg	
6479	10/31/13	F	60.0	.	Neg	Neg
6480	10/31/13	F	16.0	KISANZI	Pos	
6481	10/31/13	F	8.0	NDUGHUTU	Neg	
6482	10/31/13	M	0.3	BUNYANGONI	Neg	
6483	10/31/13	M	18.0	BUNYANGONI	Neg	
6484	10/31/13	M	3.0	BUGOYE	Neg	
6485	10/31/13	F	37.0	BUGOYE	Neg	
6486	10/31/13	F	0.4	MURAMBA	Neg	
6487	10/31/13	F	6.5	IZINGA	Pos	
6488	10/31/13	M	12.0	IZINGA	Pos	
6489	10/31/13	M	4.0	IZINGA	Pos	
6490	10/31/13	F	43.0	MUBUKU	Neg	
6491	10/31/13	M	12.0	IZINGA	Pos	
6492	10/31/13	M	5.0	IZINGA	Pos	

6493	10/31/13	M	0.8	MURAMBA	Neg	
6494	10/31/13	M	8.0	IZINGA		Pos
6495	10/31/13	F	25.0	KISENDERA	Pos	
6496	10/31/13	F	28.0	KIHINDI	Neg	
6497	10/31/13	M	4.0	MURAMBA	Neg	
6498	10/31/13	M	35.0	BUGOYE	Neg	
6499	11/1/13	F	7.0	MURAMBA	Pos	
6500	11/1/13	F	3.0	KANYAN	Neg	
6501	11/1/13	M	13.0	NDUGHUTU	Neg	
6502	11/1/13	F	8.0	BUNYANGONI	Pos	
6503	11/1/13	M	3.5	BUNYANGONI	Pos	
6504	11/1/13	F	13.0	KANYAN	Pos	
6505	11/1/13	F	22.0	MAHUGU	Pos	
6506	11/1/13	M	33.0	IBANDA	Neg	
6507	11/1/13	F	1.3	RWAKING	Pos	
6508	11/1/13	M	2.0	KAKINDO	Pos	
6509	11/1/13	F	3.0	BUNYANGONI	Neg	
6510	11/1/13	M	47.0	KAKINDO	Neg	
6511	11/1/13	F	23.0	KATUMBA	Neg	
6512	11/1/13	M	32.0	MURAMBA	Neg	
6513	11/1/13	M	4.5	NDUGHUTU	Neg	
6514	11/1/13	F	9.0	MALIBA	Neg	
6515	11/1/13	M	2.0	MALIBA	Pos	
6516	11/1/13	F	50.0	KANYAN	Neg	
6517	11/1/13	F	9.0	KIHARARA	Pos	
6518	11/1/13	F	54.0	MURAMBA	Neg	
6519	11/1/13	F	17.0	KANYAN	Pos	
6520	11/1/13	M	1.0	NDUGHUTU	Neg	
6521	11/1/13	F	16.0	BUGOYE	Neg	
6522	11/1/13	F	21.0	RWAKING	Neg	
6523	11/1/13	M	13.0	MALIBA	Pos	
6524	11/1/13	F	0.9	BUGOYE	Neg	
6525	11/1/13	M	8.0	MALIBA	Pos	
6526	11/1/13	F	6.0	MALIBA	Pos	
6527	11/1/13	F	9.0	MURAMBA	Pos	
6528	11/1/13	F	16.0	BUGOYE	Neg	
6529	11/1/13	F	35.0	KASESE	Neg	
6530	11/1/13	M	14.0	IZINGA	Pos	
6531	11/1/13	F	34.0	KANYAN	Neg	
6532	11/1/13	F	17.0	KANYAN	Pos	
6533	11/1/13	F	23.0	KYANYA	Neg	
6534	11/1/13	M	3.0	KYANYA	Pos	
6535	11/1/13	F	30.0	KANYAN	Neg	

6536	11/1/13	F	27.0	BUGOYE	Neg	
6537	11/1/13	F	1.1	BUGOYE	Neg	
6538	11/1/13	F	25.0	BUGOYE	Neg	
6539	11/1/13	F	8.0	MURAMBA	Pos	Pos
6540	11/1/13	F	11.0	MURAMBA	Pos	2
6541	11/1/13	F	24.0	IZINGA	Neg	
6542	11/1/13	F	24.0	MURAMBA	Pos	
6543	11/1/13	F	21.0	MURAMBA	Neg	
6544	11/1/13	M	3.0	MALIBA	Neg	Neg
6545	11/1/13	F	17.0	BUGOYE	Neg	
6546	11/1/13	F	17.0	MUHAMBO	Neg	Neg
6547	11/1/13	M	1.5	MURAMBA	Neg	
6548	11/1/13	M	9.0	MURAMBA	Pos	
6549	11/1/13	F	1.7	IBANDA	Pos	
6550	11/1/13	F	47.0	MALIBA	Neg	
6551	11/1/13	M	7.0	MALIBA	Pos	
6552	11/1/13	F	56.0	KYARUGOMOKA	Neg	Neg
6553	11/1/13	M	21.0	BUGOYE	Neg	
6554	11/1/13	M	3.0	NDUGHUTU	Neg	
6555	11/1/13	F	5.0	KISANZI	Pos	
6556	11/1/13	M	6.0	NDUGHUTU	Neg	
6557	11/1/13	F	3.0	NDUGHUTU	Pos	
6558	11/1/13	M	5.0	NDUGHUTU	Neg	
6559	11/1/13	F	20.0	KANYAN	Neg	
6560	11/1/13	M	15.0	KANYAN	Neg	
6561	11/1/13	M	9.5	NDUGHUTU	Pos	
6562	11/1/13	M	2.0	KATOOKE	Neg	
6563	11/1/13	F	10.0	IZINGA	Neg	
6564	11/1/13	M	1.5	MALIBA	Pos	
6565	11/1/13	M	3.5	MURAMBA	Neg	
6566	11/1/13	M	0.8	MALIBA	Neg	
6567	11/1/13	F	21.0	KABUYIRI	Neg	
6568	11/1/13	F	0.6	MALIBA	Neg	
6569	11/1/13	F	29.0	KIBIRIZI	Neg	
6570	11/1/13	F	32.0	IBANDA	Neg	
6571	11/1/13	F	23.0	MURAMBA	Pos	
6572	11/5/13	F	6.5	KISAZNI		Neg
6573	11/5/13	M	0.5	BULINDUGURU	Neg	
6574	11/5/13	F	6.0	KAKINDO	Pos	
6575	11/5/13	F	3.0	IZINGA	Neg	
6576	11/5/13	M	3.0	MUBUKU	Pos	
6577	11/5/13	F	13.0	KANYAN	Neg	
6578	11/5/13	M	13.0	IZINGA	Neg	

6579	11/5/13	F	16.0	IBANDA	Neg	
6580	11/5/13	M	0.9	MALIBA	Neg	
6581	11/5/13	F	58.0	NYAKABUGHA	Neg	
6582	11/5/13	F	56.0	KYARUGOMOKA	Neg	
6583	11/5/13	F	4.0	MALIBA	Pos	
6584	11/5/13	F	0.8	MALIBA	Neg	
6585	11/5/13	F	10.0	MALIBA	Neg	
6586	11/5/13	M	6.0	IZINGA	Pos	
6587	11/5/13	F	40.0	IZINGA	Pos	
6588	11/5/13	M	2.0	KATEBE	Neg	
6589	11/5/13	F	2.3	KAKINDO	Pos	
6590	11/5/13	F	18.0	KATUMBA	Pos	
6591	11/5/13	F	16.0	IZINGA	Pos	
6592	11/5/13	F	30.0	MURAMBA	Neg	
6593	11/5/13	M	10.0	IZINGA	Pos	
6594	11/5/13	M	0.5	MURAMBA	Neg	
6595	11/5/13	F	0.8	KYANYA	Neg	
6596	11/6/13	F	13.0	IZINGA	Neg	
6597	11/6/13	M	0.0	KISOJO	Neg	
6598	11/6/13	F	0.9	BUNYANGONI	Neg	
6599	11/6/13	M	0.8	MUHAMBO	Neg	
6600	11/6/13	M	2.0	MURAMBA	Pos	
6601	11/6/13	F	48.0	KYANYA	Neg	
6602	11/6/13	F	42.0	KYARUGOMOKA	Neg	
6603	11/6/13	F	13.0	KYARUGOMOKA	Neg	
6604	11/6/13	M	14.0	IZINGA	Pos	
6605	11/6/13	M	2.0	KIRONGO	Neg	
6606	11/6/13	M	18.0	KANYAN	Pos	
6607	11/6/13	F	17.0	KANYAN	Pos	Neg
6608	11/6/13	F	45.0	BUNYANGONI	Pos	
6609	11/6/13	M	1.5	MUHAMBO	Pos	
6610	11/6/13	M	5.0	BUNYANGONI	Pos	
6611	11/6/13	M	24.0	BUGOYE	Neg	
6612	11/6/13	M	1.3	BUNYANGONI		Pos
6613	11/6/13	F	19.0	MURAMBA	Neg	
6614	11/6/13	F	0.1	KIBIRIZI	Neg	
6615	11/7/13	F	46.0	RWAKING	Neg	
6616	11/7/13	F	25.0	MURAMBA	Neg	
6617	11/7/13	F	35.0	RWAKING	Neg	
6618	11/7/13	F	13.0	BUGOYE	Pos	
6619	11/7/13	F	3.0	MALIBA	Pos	
6620	11/7/13	M	18.0	NDUGHUTU	Neg	
6621	11/7/13	F	0.1	NDUGHUTU	Neg	

6622	11/7/13	F	17.0	MURAMBA	Neg	
6623	11/7/13	F	0.2	MAGHOMA	Neg	
6624	11/7/13	M	8.0	KANYAN	Pos	
6625	11/7/13	F	7.0	KANYAN	Neg	
6626	11/7/13	F	38.0	BUGOYE	Pos	
6627	11/7/13	M	9.0	MALIBA	Pos	
6628	11/7/13	F	60.0	MURAMBA	Pos	
6629	11/7/13	F	11.0	MURAMBA	Neg	
6630	11/7/13	M	0.2	KISOJO	Neg	
6631	11/7/13	F	5.0	KYARUGOMOKA	Neg	
6632	11/7/13	F	28.0	MALIBA	Neg	
6633	11/7/13	M	2.3	BUNYANGONI	Pos	
6634	11/7/13	M	32.0	KIKOKERA	Pos	
6635	11/7/13	F	3.3	KANYAN	Pos	
6636	11/7/13	F	1.2	IZINGA	Neg	
6637	11/8/13	M	23.0	MALIBA	Pos	
6638	11/8/13	F	1.1	BUGOYE		Neg
6639	11/8/13	M	4.0	IBANDA	Neg	
6640	11/8/13	F	1.3	RWAKING	Pos	
6641	11/8/13	F	13.0	RWAKING	Pos	
6642	11/8/13	F	4.0	BUGOYE	Pos	
6643	11/8/13	F	5.0	BUNYANGONI		Pos
6644	11/8/13	F	6.0	MALIBA	Neg	
6645	11/8/13	M	2.0	BUGOYE	Pos	
6646	11/8/13	M	9.0	KANYAN	Neg	
6647	11/8/13	F	50.0	BUGOYE	Neg	
6648	11/8/13	M	7.0	BUNYANGONI	Pos	
6649	11/8/13	F	40.0	.		
6650	11/8/13	F	14.0	KYARUGOMOKA	Neg	
6651	11/8/13	F	10.0	KYARUGOMOKA	Pos	
6652	11/8/13	F	9.0	KIKOKERA	Neg	
6653	11/8/13	F	32.0	BUNYANGONI	Pos	
6654	11/8/13	F	3.5	MAGHOMA	Neg	
6655	11/8/13	M	7.5	BUGOYE	Neg	
6656	11/8/13	M	40.0	KYARUGOMOKA	Pos	
6657	11/8/13	M	11.0	KYARUGOMOKA	Pos	
6658	11/8/13	F	1.7	BUNYANGONI	Neg	
6659	11/8/13	F	52.0	BUGOYE	Pos	
6660	11/8/13	F	6.0	KANYAN	Pos	
6661	11/8/13	M	34.0	BUGOYE	Neg	
6662	11/8/13	F	8.0	KYARUGOMOKA	Pos	
6663	11/8/13	M	12.0	KATUMBA	Pos	
6664	11/8/13	M	0.2	BUNYANGONI	Neg	

6665	11/11/13	F	75.0	KIHINDI		Pos
6666	11/11/13	M	9.0	MURAMBA	Pos	
6667	11/11/13	F	12.0	BUGOYE	Neg	
6668	11/11/13	F	3.0	BUGOYE	Neg	
6669	11/11/13	F	0.8	IZINGA	Pos	
6670	11/11/13	F	30.0	KANYAN	Neg	
6671	11/11/13	F	1.8	MURAMBA	Neg	
6672	11/11/13	M	0.6	.	Neg	
6673	11/11/13	F	0.4	MURAMBA	Neg	
6674	11/11/13	F	0.9	MURAMBA	Neg	
6675	11/11/13	M	10.0	KANYAN	Neg	
6676	11/11/13	F	6.5	KANYAN	Neg	
6677	11/11/13	M	3.5	KANYAN	Neg	
6678	11/11/13	F	0.8	MALIBA	Pos	
6679	11/11/13	F	46.0	KANYAN	Neg	
6680	11/11/13	M	7.0	KANYAN	Neg	
6681	11/11/13	F	15.0	MURAMBA	Neg	
6682	11/11/13	M	6.0	RWAKING	Neg	
6683	11/11/13	M	9.0	RWAKING	Neg	
6684	11/11/13	M	2.0	KANYAN	Pos	
6685	11/11/13	M	0.8	RWAKING	Neg	
6686	11/11/13	F	16.0	KANYAN	Neg	
6687	11/11/13	F	18.0	BUGOYE	Neg	
6688	11/11/13	M	18.0	MURAMBA	Pos	
6689	11/11/13	M	42.0	KIRONGO	Neg	
6690	11/11/13	F	26.0	RWAKING	Neg	
6691	11/11/13	F	23.0	NYAKARUNGU	Neg	
6692	11/11/13	F	1.5	KIRULI	Neg	
6693	11/11/13	M	5.0	NDUGHUTU	Neg	
6694	11/11/13	F	3.0	MALIBA	Pos	
6695	11/11/13	M	2.0	KIGANDO	Neg	
6696	11/11/13	F	20.0	IZINGA	Neg	
6697	11/11/13	M	2.0	IZINGA	Pos	
6698	11/11/13	F	5.0	MALIBA	Pos	
6699	11/11/13	F	0.9	KIKOKERA	Neg	
6700	11/11/13	F	14.0	MALIBA	Pos	
6701	11/11/13	M	10.0	MALIBA	Pos	
6702	11/11/13	F	1.1	KIKOKERA	Neg	
6703	11/11/13	M	3.0	BUGOYE	Pos	
6704	11/11/13	F	11.0	RWAKING	Pos	
6705	11/11/13	F	16.0	BUGOYE	Neg	
6706	11/11/13	F	12.0	KABUYIRI	Neg	
6707	11/11/13	F	30.0	MALIBA	Neg	

6708	11/11/13	F	55.0	MURAMBA	Pos	
6709	11/11/13	F	2.0	KANYAN	Neg	
6710	11/11/13	F	1.5	MUHAMBO	Neg	
6711	11/11/13	F	45.0	KATUMBA	Neg	
6712	11/11/13	M	16.0	BUNYANGONI	Neg	
6713	11/11/13	M	72.0	KATOOKE	Pos	
6714	11/11/13	F	26.0	IHANI	Neg	
6715	11/11/13	F	2.8	KIRULI	Neg	
6716	11/11/13	F	28.0	KIKOKERA	Neg	
6717	11/11/13	M	12.0	MURAMBA	Neg	
6718	11/11/13	F	12.0	NDUGHUTU	Neg	
6719	11/11/13	M	10.0	NDUGHUTU	Neg	
6720	11/11/13	M	4.0	MALIBA	Pos	
6721	11/11/13	M	19.0	BUNYANGONI	Neg	
6722	11/11/13	M	13.0	KANYAN	Pos	
6723	11/11/13	F	26.0	MALIBA	Pos	
6724	11/11/13	F	32.0	BULINDUGURU	Neg	
6725	11/12/13	M	16.0	MURAMBA	Pos	
6726	11/12/13	F	58.0	KIBIRIZI	Neg	
6727	11/12/13	M	16.0	MURAMBA	Pos	
6728	11/12/13	M	13.0	RWAKING	Neg	
6729	11/12/13	F	60.0	KISAMBA	Neg	
6730	11/12/13	M	5.0	IZINGA	Pos	
6731	11/12/13	F	45.0	BUGOYE	Neg	
6732	11/12/13	F	21.0	NDUGHUTU	Pos	Pos
6733	11/12/13	F	32.0	ISULE	Neg	
6734	11/12/13	F	30.0	ISENGERA	Pos	
6735	11/12/13	M	0.9	ISENGERA	Pos	
6736	11/12/13	F	5.0	ISENGERA	Pos	
6737	11/12/13	F	19.0	KANYAN	Neg	
6738	11/12/13	F	1.8	IZINGA	Neg	
6739	11/12/13	F	4.0	KANYAN	Pos	
6740	11/12/13	M	38.0	ISULE	Neg	
6741	11/12/13	F	31.0	BUGOYE	Neg	
6742	11/12/13	F	11.0	NDUGHUTU	Neg	
6743	11/12/13	F	2.0	KANYAN	Neg	
6744	11/12/13	F	37.0	KYANYA	Neg	
6745	11/12/13	M	55.0	MURAMBA	Pos	
6746	11/12/13	M	47.0	RWAKING	Neg	
6747	11/12/13	F	15.0	KANYAN	Pos	
6748	11/12/13	M	10.0	BUNYANGONI	Pos	
6749	11/12/13	F	9.0	NYAKALENGIJO	Pos	
6750	11/12/13	M	22.0	MALIBA	Pos	

6751	11/12/13	M	30.0	MURAMBA	Neg	
6752	11/12/13	M	17.0	KANYAN	Neg	
6753	11/12/13	F	9.0	BUZIBWERA	Pos	
6754	11/12/13	F	1.5	KYANYA	Neg	
6755	11/12/13	F	30.0	KANYAN	Pos	
6756	11/12/13	M	7.0	BUZIBWERA	Pos	
6757	11/12/13	M	13.0	KISENDERA		Neg
6758	11/12/13	F	10.0	.	Neg	
6759	11/14/13	F	9.0	.	Pos	
6760	11/14/13	F	18.0	.	Neg	
6761	11/14/13	F	79.0	.	Neg	
6762	11/14/13	F	80.0	.	Neg	
6763	11/14/13	M	9.0	.	Pos	
6764	11/14/13	M	1.5	.	Pos	
6765	11/14/13	M	60.0	.	Neg	
6766	11/14/13	F	20.0	.	Pos	
6767	11/14/13	F	4.0	.	Neg	
6768	11/14/13	F	45.0	.	Pos	
6769	11/14/13	M	20.0	.	Pos	
6770	11/14/13	F	8.0	.	Pos	
6771	11/14/13	M	4.0	.	Neg	
6772	11/14/13	M	2.0	.	Neg	
6773	11/14/13	F	14.0	.	Neg	
6774	11/14/13	F	10.0	.	Neg	
6775	11/14/13	M	5.0	.	Neg	
6776	11/14/13	F	44.0	.	Neg	
6777	11/14/13	M	10.0	.	Neg	
6778	11/14/13	F	12.0	.	Pos	
6779	11/14/13	M	8.0	.	Pos	
6780	11/14/13	M	3.0	.	Pos	
6781	11/14/13	M	13.0	.	Pos	
6782	11/14/13	M	3.5	.	Neg	
6783	11/14/13	M	0.4	.	Neg	
6784	11/14/13	F	47.0	.	Neg	
6785	11/14/13	M	25.0	.	Neg	
6786	11/14/13	F	45.0	.	Neg	
6787	11/14/13	F	45.0	.	Neg	
6788	11/14/13	M	0.7	.	Neg	
6789	11/14/13	F	18.0	NYANGONGE	Neg	
6790	11/14/13	M	2.5	KAKINDO	Pos	
6791	11/14/13	F	12.0	.	Neg	
6792	11/14/13	F	25.0	MURAMBA	Pos	
6793	11/14/13	F	11.0	IBANDA	Neg	

6794	11/14/13	F	11.0	IBANDA	Neg	
6795	11/14/13	F	13.0	IBANDA	Neg	
6796	11/14/13	F	16.0	IZINGA	Pos	
6797	11/14/13	M	1.3	KIHARARA	Neg	
6798	11/14/13	F	3.0	BUNYANGONI	Pos	
6799	11/14/13	F	11.0	BUNYANGONI	Pos	
6800	11/14/13	F	38.0	KANYAN	Neg	
6801	11/14/13	M	0.8	KIHARARA	Neg	
6802	11/14/13	M	19.0	KANYAN	Neg	
6803	11/14/13	M	17.0	IHANI	Neg	
6804	11/14/13	M	10.0	MURAMBA	Neg	
6805	11/14/13	M	13.0	KIKOKERA	Neg	
6806	11/15/13	F	17.0	MURAMBA	Pos	Neg
6807	11/15/13	F	33.0	BUGOYE	Neg	
6808	11/15/13	M	0.5	IHANI	Neg	
6809	11/15/13	F	7.0	NDUGHUTU	Pos	
6810	11/15/13	F	7.0	NDUGHUTU	Neg	
6811	11/15/13	F	13.0	KANYAN	Pos	
6812	11/15/13		14.0	BUNYANGONI	Neg	
6813	11/15/13	F	19.0	MALIBA	Neg	
6814	11/15/13	F	9.0	MALIBA	Pos	
6815	11/15/13	M	3.0	IZINGA	Pos	
6816	11/15/13	F	40.0	MALIBA	Neg	
6817	11/15/13	M	17.0	BUGOYE	Neg	
6818	11/15/13	F	44.0	BUGOYE	Neg	
6819	11/15/13	M	0.0	KIRONGO	Neg	
6820	11/15/13	F	23.0	KIRONGO	Neg	
6821	11/15/13	F	19.0	BUGOYE	Neg	
6822	11/18/13	F	47.0	BUNYANGONI	Neg	
6823	11/18/13	F	35.0	RWAKING	Neg	
6824	11/18/13	F	5.0	MAGHOMA	Neg	
6825	11/18/13	F	2.0	MAGHOMA	Neg	
6826	11/18/13	M	0.8	MAGHOMA	Neg	
6827	11/18/13	M	45.0	IZINGA	Neg	
6828	11/18/13	F	12.0	MURAMBA	Neg	
6829	11/18/13	M	4.0	IZINGA	Neg	
6830	11/18/13	M	22.0	IBANDA	Neg	
6831	11/18/13	M	72.0	IZINGA	Neg	
6832	11/18/13	F	11.0	NDUGHUTU	Neg	
6833	11/18/13	F	0.0	RWAKING	Neg	
6834	11/18/13	F	12.0	MALIBA	Neg	
6835	11/18/13	M	13.0	BUGOYE	Neg	
6836	11/18/13	M	0.1	NYAKABUGHA	Neg	

6837	11/18/13	F	10.0	BUZIBWERA	Pos	Pos
6838	11/18/13	F	4.0	KAKINDO	Pos	
6839	11/18/13	F	15.0	MALIBA	Pos	
6840	11/18/13	M	46.0	IZINGA	Pos	
6841	11/18/13	M	1.1	KANYAN	Neg	
6842	11/18/13	F	24.0	BUHUNGA	Neg	
6843	11/18/13	F	25.0	BUGOYE	Neg	
6844	11/18/13	M	6.0	MALIBA	Neg	
6845	11/18/13	F	50.0	NYAKABUGHA	Neg	
6846	11/18/13	M	0.8	IBANDA	Neg	
6847	11/18/13	M	10.0	IZINGA	Neg	
6848	11/18/13	F	4.0	KAKINDO	Neg	
6849	11/18/13	M	38.0	IZINGA	Pos	
6850	11/18/13	M	14.0	IZINGA	Neg	
6851	11/18/13	F	35.0	BUNYANGONI	Neg	
6852	11/18/13	M	4.0	BUNYANGONI	Pos	
6853	11/18/13	M	8.0	BUNYANGONI	Pos	
6854	11/18/13	F	2.2	NYAKELENGIJO	Pos	
6855	11/18/13	M	7.0	BUNYANGONI	Neg	
6856	11/18/13	F	45.0	NDUGHUTU	Neg	
6857	11/18/13	F	13.0	IZINGA	Neg	
6858	11/18/13	F	7.0	MALIBA	Neg	
6859	11/18/13	F	34.0	IZINGA	Neg	
6860	11/18/13	F	12.0	IZINGA	Pos	
6861	11/18/13	M	40.0	IBANDA	Pos	
6862	11/18/13	M	14.0	IZINGA	Pos	
6863	11/18/13	F	14.0	KYANYA	Neg	
6864	11/18/13	F	42.0	IHANI	Pos	
6865	11/18/13	F	23.0	MURAMBA	Neg	
6866	11/18/13	M	2.0	MURAMBA	Neg	
6867	11/18/13	F	4.0	KANYAN	Pos	
6868	11/18/13	M	2.5	BUNYANGONI	Neg	
6869	11/18/13	M	9.0	MALIBA	Pos	
6870	11/18/13	M	60.0	IBANDA	Pos	
6871	11/18/13	F	30.0	MALIBA	Neg	
6872	11/18/13	F	30.0	KYARUGOMOKA	Neg	
6873	11/18/13	M	19.0	MURAMBA		Neg
6874	11/18/13	F	1.5	KYARUGOMOKA	Pos	
6875	11/18/13	M	20.0	BUGOYE		Neg
6876	11/18/13	M	49.0	BUGOYE		Neg
6877	11/18/13	M	29.0	BUGOYE		Neg
6878	11/18/13	M	40.0	BUGOYE		Neg
6879	11/18/13	M	31.0	BUGOYE		Neg

6880	11/18/13	M	24.0	BUGOYE		Neg
6881	11/18/13	M	2.5	BUGOYE	Pos	
6882	11/19/13	M	14.0	KANYAN		Neg
6883	11/19/13	F	49.0	KISANZI	Pos	
6884	11/19/13	F	18.0	MURAMBA	Neg	
6885	11/19/13	M	8.0	MURAMBA	Neg	
6886	11/19/13	M	18.0	RWENZORI	Neg	
6887	11/19/13	M	25.0	RWAKING	Pos	
6888	11/19/13	M	13.0	BUGOYE	Pos	
6889	11/19/13	F	1.4	MURAMBA		Neg
6890	11/19/13	F	12.0	MALIBA	Pos	
6891	11/19/13	F	7.0	MALIBA	Pos	
6892	11/19/13	F	13.0	MALIBA	Pos	
6893	11/19/13	M	14.0	RWAKING	Pos	
6894	11/19/13	M	27.0	RWAKING	Neg	
6895	11/19/13	M	10.0	BUGOYE	Neg	
6896	11/19/13	F	52.0	KYARUGOMOKA	Pos	
6897	11/19/13	F	8.0	KYARUGOMOKA	Pos	
6898	11/19/13	F	11.0	BUGOYE	Neg	
6899	11/19/13	F	2.5	KISANZI	Neg	
6900	11/19/13	M	12.0	BUNYANGONI	Pos	
6901	11/19/13	F	1.8	KANYAN	Pos	
6902	11/19/13	M	10.0	NDUGHUTU	Neg	
6903	11/19/13	F	38.0	NDUGHUTU	Neg	
6904	11/19/13	F	10.0	MALIBA	Neg	
6905	11/19/13	F	26.0	BUGOYE	Neg	Neg
6906	11/19/13	M	1.0	NDUGHUTU	Neg	
6907	11/19/13	F	0.5	KIRONGO	Neg	
6908	11/19/13	F	11.0	KANYAN	Neg	
6909	11/19/13	F	23.0	NDUGHUTU	Neg	
6910	11/19/13	M	13.0	RWAKING	Neg	
6911	11/19/13	F	5.0	IHANI	Pos	
6912	11/19/13	F	12.0	IHANI	Pos	
6913	11/19/13	M	3.0	RWAKING	Neg	
6914	11/19/13	F	2.5	MALIBA	Pos	
6915	11/19/13	F	8.0	KIRONGO	Neg	
6916	11/19/13	F	5.0	KIRONGO	Neg	
6917	11/19/13	F	11.0	MURAMBA	Neg	
6918	11/19/13	M	10.0	MURAMBA	Neg	
6919	11/19/13	F	1.3	RWAKING	Neg	
6920	11/19/13	F	30.0	KANYAN	Pos	
6921	11/19/13	F	50.0	BUGOYE	Neg	
6922	11/19/13	F	0.5	KIGANDO	Neg	

6923	11/19/13	F	50.0	BUGOYE		Neg
6924	11/19/13	F	40.0	BUGOYE		Neg
6925	11/19/13	F	30.0	BUGOYE		Neg
6926	11/19/13	M	35.0	BUGOYE		Neg
6927	11/19/13	F	48.0	BUGOYE		Neg
6928	11/19/13	F	32.0	BUGOYE		Neg
6929	11/19/13	F	35.0	BUGOYE		Neg
6930	11/19/13	F	20.0	BUGOYE		Neg
6931	11/20/13	F	28.0	BUGOYE	Neg	
6932	11/20/13	F	10.0	RWAKING	Pos	Pos
6933	11/20/13	F	20.0	KISANZI	Neg	
6934	11/20/13	F	1.4	IZINGA	Pos	
6935	11/20/13	F	0.3	KIRULI		Neg
6936	11/20/13	M	7.0	KIRULI	Pos	
6937	11/20/13	F	36.0	KIRULI	Neg	
6938	11/20/13	M	0.5	MURAMBA	Pos	
6939	11/20/13	F	32.0	BUNYANGONI	Neg	
6940	11/20/13	F	5.0	KYANYA	Neg	
6941	11/20/13	F	10.0	KYANYA	Pos	
6942	11/20/13	M	0.5	MALIBA	Pos	
6943	11/20/13	M	52.0	NYAKABUGHA	Neg	
6944	11/20/13	F	16.0	MALIBA	Neg	
6945	11/20/13	F	18.0	NYAKALENGIJO	Neg	
6946	11/20/13	F	28.0	RWAKING	Neg	
6947	11/20/13	M	9.0	RWAKING	Pos	
6948	11/20/13	M	18.0	MALIBA	Pos	
6949	11/20/13	F	6.0	IZINGA	Neg	
6950	11/20/13	M	55.0	RWAKING	Neg	
6951	11/20/13	F	44.0	IZINGA	Neg	
6952	11/20/13	M	6.0	IZINGA	Pos	
6953	11/20/13	F	23.0	MUKATI	Neg	
6954	11/20/13	F	0.7	KISANDE	Pos	
6955	11/20/13	F	18.0	MALIBA	Pos	
6956	11/20/13	M	25.0	MALIBA	Neg	
6957	11/20/13	F	26.0	KANYAN	Pos	
6958	11/20/13	F	0.7	KATOOKE	Neg	
6959	11/20/13	F	10.0	MASAKA	Neg	
6960	11/20/13	F	7.0	MASAKA	Neg	
6961	11/20/13	F	1.3	KIRULI	Pos	
6962	11/21/13	M	0.8	MURAMBA	Neg	
6963	11/21/13	F	2.8	MURAMBA	Pos	
6964	11/21/13	F	11.0	MURAMBA	Pos	
6965	11/21/13	F	20.0	RWAKING	Pos	

6966	11/21/13	F	11.0	BUGOYE	Neg	
6967	11/21/13	F	13.0	MALIBA	Pos	
6968	11/21/13	F	5.0	MALIBA	Pos	
6969	11/21/13	M	4.0	MALIBA	Pos	
6970	11/21/13	F	13.0	BUGOYE	Neg	
6971	11/21/13	M	1.0	NDUGHUTU	Neg	
6972	11/21/13	F	1.5	MURAMBA	Neg	
6973	11/21/13	F	18.0	MURAMBA	Pos	
6974	11/21/13	M	1.7	KANYAN	Pos	
6975	11/21/13	F	40.0	NYAKABUGHA	Neg	
6976	11/21/13	F	18.0	NYAKABUGHA	Pos	
6977	11/21/13	M	1.4	KISANZI	Pos	
6978	11/21/13	M	0.3	BUGOYE	Neg	Neg
6979	11/21/13	F	7.0	BUNYANGONI	Pos	
6980	11/21/13	F	30.0	MURAMBA	Pos	
6981	11/21/13	M	34.0	BUNYANGONI	Neg	
6982	11/21/13	M	5.0	KANYAN	Pos	
6983	11/21/13	M	4.5	KANYAN	Pos	
6984	11/21/13	F	40.0	KATOOKE	Neg	
6985	11/21/13	F	23.0	MURAMBA	Pos	
6986	11/21/13	M	22.0	KYARUGOMOKA	Pos	
6987	11/21/13	F	25.0	MURAMBA	Neg	
6988	11/21/13	M	0.5	KANYAN	Neg	
6989	11/21/13	F	19.0	KIKOKERA	Neg	
6990	11/21/13	F	1.3	KATOOKE	Neg	
6991	11/21/13	M	10.0	MURAMBA	Neg	
6992	11/22/13	F	0.2	KANYAN	Neg	
6993	11/22/13	F	10.0	KISANGA	Pos	
6994	11/22/13	F	0.8	RWAKING	Neg	
6995	11/22/13	M	45.0	NYAKARUNGU	Neg	
6996	11/22/13	M	21.0	KISAMBA	Neg	
6997	11/22/13	F	3.8	MALIBA	Pos	Pos
6998	11/22/13	F	5.5	BUGOYE	Neg	
6999	11/22/13	M	1.0	NYABISUSI	Pos	
7000	11/22/13	F	17.0	KIBOTA	Pos	
7001	11/25/13	F	0.7	KANYAN	Neg	
7002	11/25/13	M	2.3	IZINGA	Neg	
7003	11/25/13	F	4.0	BUGOYE	Pos	
7004	11/25/13	M	34.0	BUGOYE	Neg	
7005	11/25/13	F	19.0	BUGOYE	Neg	
7006	11/25/13	M	29.0	MALIBA	Pos	
7007	11/25/13	M	1.1	MALIBA	Pos	Pos
7008	11/25/13	M	50.0	MALIBA	Neg	

7009	11/25/13	M	21.0	IZINGA	Pos	
7010	11/25/13	F	45.0	BUGOYE	Neg	
7011	11/25/13	F	13.0	BUGOYE	Neg	
7012	11/25/13	M	50.0	KANYAN	Neg	
7013	11/25/13	M	20.0	MAGHOMA	Pos	
7014	11/25/13	F	19.0	.	Neg	
7015	11/25/13	F	46.0	KYARUGOMOKA	Neg	
7016	11/25/13	M	12.0	MURAMBA	Neg	
7017	11/25/13	M	2.0	NDUGHUTU	Neg	Neg
7018	11/25/13	M	1.3	NDUGHUTU	Pos	
7019	11/25/13	M	32.0	KANYAN	Pos	
7020	11/25/13	F	49.0	BUNYANGONI	Neg	
7021	11/25/13	F	29.0	KYANYA	Neg	
7022	11/25/13	F	1.0	KYANYA	Neg	
7023	11/25/13	F	20.0	MURAMBA	Pos	
7024	11/25/13	F	3.4	.	Pos	
7025	11/25/13	F	11.0	.	Neg	
7026	11/25/13	M	23.0	.	Neg	
7027	11/25/13	M	0.3	MURAMBA	Neg	
7028	11/25/13	F	2.5	BUNYANGONI	Pos	
7029	11/25/13	F	17.0	MURAMBA	Pos	
7030	11/25/13	F	16.0	BUGOYE	Neg	
7031	11/25/13	F	38.0	IHANI	Pos	
7032	11/25/13	M	20.0	MALIBA	Pos	
7033	11/25/13	F	30.0	MALIBA	Neg	
7034	11/25/13	M	0.9	MUHAMBO	Neg	
7035	11/25/13	F	14.0	RWAKING	Neg	
7036	11/25/13	F	13.0	IZINGA	Pos	
7037	11/25/13	F	27.0	BULINDUGURU	Neg	
7038	11/25/13	M	54.0	BUNYANGONI	Neg	
7039	11/25/13	F	43.0	KYARUGOMOKA	Neg	
7040	11/25/13	M	26.0	MURAMBA	Neg	Neg
7041	11/25/13	M	15.0	RWAKING	Pos	
7042	11/25/13	F	1.3	RWAKING	Neg	
7043	11/25/13	F	3.5	NDUGHUTU	Neg	
7044	11/25/13	F	29.0	BUZIBWERA	Neg	
7045	11/25/13	F	1.3	BUNYANGONI	Pos	
7046	11/25/13	F	21.0	BUNYANGONI	Neg	
7047	11/25/13	F	1.5	MALIBA	Neg	
7048	11/25/13	F	25.0	MURAMBA	Neg	
7049	11/25/13	F	0.1	MURAMBA	Neg	
7050	11/25/13	F	15.0	KYARUGOMOKA	Neg	
7051	11/25/13	F	5.0	BUGOYE	Neg	

7052	11/25/13	F	50.0	BUGOYE	Neg	
7053	11/25/13	F	38.0	NYANUAMGA	Neg	
7054	11/25/13	F	28.0	BUGOYE	Pos	
7055	11/25/13	F	12.0	MURAMBA	Pos	
7056	11/25/13	F	16.0	NYAKARUNGU	Neg	
7057	11/25/13	M	1.3	KATUMBA	Neg	
7058	11/26/13	F	1.0	RWAKING	Pos	
7059	11/26/13	F	24.0	IBANDA	Neg	
7060	11/26/13	F	10.0	IBANDA	Pos	
7061	11/26/13	F	30.0	KYANYA	Neg	
7062	11/26/13	F	30.0	KYANYA	.	Pos
7063	11/26/13	F	26.0	MUHAMBO	Pos	
7064	11/26/13	F	3.0	KANYAN	Pos	
7065	11/26/13	F	0.6	RWAKING	Neg	
7066	11/26/13	F	13.0	KANYAN	Neg	
7067	11/26/13	F	20.0	KYANYA	Neg	
7068	11/26/13	F	16.0	BUGOYE	Neg	
7069	11/26/13	M	50.0	KAKINDO	Neg	
7070	11/26/13	M	0.4	RWAKING	Neg	
7071	11/26/13	F	32.0	MUBUKU	Neg	
7072	11/26/13	F	17.0	IZINGA	Pos	
7073	11/26/13	F	27.0	KANYAN	Neg	
7074	11/26/13	F	11.0	NDUGHUTU	Pos	
7075	11/26/13	F	3.0	BUGOYE	Pos	
7076	11/26/13	M	0.9	BUGOYE	Neg	
7077	11/26/13	F	5.0	BUNYANGONI	Neg	
7078	11/26/13	F	2.0	NDUGHUTU	Pos	
7079	11/26/13	F	35.0	NDUGHUTU	Neg	
7080	11/26/13	M	2.5	BUGOYE	Pos	
7081	11/26/13	M	2.0	BUGOYE	Pos	
7082	11/26/13	F	28.0	NDUGHUTU	Neg	
7083	11/26/13	F	22.0	NYAKARUNGU	Neg	
7084	11/26/13	M	18.0	IBANDA	Pos	
7085	11/26/13	M	1.1	MURAMBA	Neg	
7086	11/26/13	M	1.3	KATUMBA	Neg	
7087	11/26/13	M	14.0	KANYAN	Pos	
7088	11/26/13	M	14.0	BUGOYE	Neg	
7089	11/26/13	F	2.4	KYARUGOMOKA	Pos	
7090	11/27/13	F	1.2	IBANDA	Neg	
7091	11/27/13	F	50.0	IZINGA	Neg	
7092	11/27/13	M	4.0	IZINGA	Pos	
7093	11/27/13	F	0.5	RWAKING	Neg	
7094	11/27/13	M	11.0	KYANYA	Pos	

7095	11/27/13	F	0.9	BUGOYE	Neg
7096	11/27/13	M	1.8	KIHINDI	Neg
7097	11/27/13	F	20.0	MURAMBA	Pos
7098	11/27/13	M	1.3	MUHAMBO	Neg
7099	11/27/13	F	4.0	BUGOYE	Neg
7100	11/27/13	F	19.0	BUNYANGONI	Neg
7101	11/27/13	F	20.0	NDUGHUTU	Neg
7102	11/27/13	M	12.0	IBANDA	Pos
7103	11/27/13	M	15.0	MUHAMBO	Neg
7104	11/27/13	M	0.1	RWAKING	Neg
7105	11/27/13	M	6.0	ISULE	Neg
7106	11/27/13	F	26.0	BUGOYE	Neg
7107	11/27/13	M	3.7	KASESE	Pos
7108	11/27/13	M	11.0	BUGOYE	Pos
7109	11/27/13	M	4.5	BUGOYE	Pos
7110	11/27/13	F	35.0	MUHAMBO	Neg
7111	11/27/13	F	1.8	.	Pos
7112	11/27/13	F	2.5	.	Pos
7113	11/27/13	M	5.5	IZINGA	Pos
7114	11/27/13	F	9.0	IZINGA	Pos
7115	11/27/13	F	8.0	MUHAMBO	Neg
7116	11/27/13	M	0.3	KATOOKE	Neg
7117	11/27/13	M	7.0	RWAKING	Neg
7118	11/27/13	F	55.0	NYAKALENGIJO	Neg
7119	11/27/13	M	9.0	MUHAMBO	Neg
7120	11/28/13	F	5.0	MURAMBA	Pos
7121	11/28/13	F	23.0	MURAMBA	Pos
7122	11/28/13	F	22.0	IZINGA	Neg
7123	11/28/13	F	25.0	BUGOYE	Neg
7124	11/28/13	F	20.0	MURAMBA	Neg
7125	11/28/13	M	5.0	MURAMBA	Neg
7126	11/28/13	M	1.3	BUNYANGONI	Pos
7127	11/28/13	M	0.5	KANYAN	Neg
7128	11/28/13	M	12.0	MURAMBA	Pos
7129	11/28/13	F	10.0	BUGOYE	Pos
7130	11/28/13	F	39.0	KAKINDO	Neg
7131	11/28/13	M	11.0	MALIBA	Neg
7132	11/28/13	M	4.8	KAKINDO	Neg
7133	11/28/13	F	50.0	NDUGHUTU	Neg
7134	11/28/13	F	70.0	MURAMBA	Neg
7135	11/28/13	F	27.0	IBANDA	Neg
7136	11/28/13	F	17.0	RWAKING	Pos
7137	11/28/13	F	27.0	IBANDA	Neg

7138	11/28/13	F	35.0	IBANDA	Neg
7139	11/28/13	F	10.0	KIKOKERA	Neg
7140	11/28/13	M	21.0	BUHUNGA	Neg
7141	11/28/13	F	7.0	KIKOKERA	Pos
7142	11/28/13	M	22.0	IBANDA	Neg
7143	11/28/13	F	1.2	MUHAMBO	Neg
7144	11/28/13	M	15.0	BUGOYE	Neg
7145	11/28/13	F	45.0	KIGANDO	Neg
7146	11/28/13	F	16.0	IZINGA	Neg
7147	11/28/13	F	32.0	.	Neg
7148	11/28/13	M	12.0	IZINGA	Pos
7149	11/28/13	M	0.3	KATOOKE	Neg
7150	11/28/13	F	62.0	MURAMBA	Pos
7151	11/28/13	F	1.2	MURAMBA	Neg
7152	11/28/13	M	50.0	KYANYA	Neg
7153	11/28/13	M	37.0	BUGOYE	Neg
7154	11/28/13	F	19.0	KYANJIMA	Neg
7155	11/28/13	M	1.1	.	Neg
7156	11/29/13	M	1.3	BUGOYE	Neg
7157	11/29/13	F	3.0	IZINGA	Pos
7158	11/29/13	F	24.0	MBABAZI	Neg
7159	11/29/13	F	0.1	BUGOYE	Neg
7160	11/29/13	F	19.0	KISAMBA	Neg
7161	11/29/13	F	12.0	KISAMBA	Pos
7162	11/29/13	F	47.0	KABUYIRI	Neg
7163	11/29/13	F	0.3	KAKINDO	Neg
7164	11/29/13	F	46.0	RWAKING	Neg
7165	11/29/13	F	5.0	BUGOYE	Neg
7166	11/29/13	F	4.0	IZINGA	Neg
7167	11/29/13	M	3.0	BUGOYE	Pos
7168	11/29/13	F	0.8	IZINGA	Neg
7169	11/29/13	M	5.0	IZINGA	Pos
7170	11/29/13	M	3.0	IZINGA	Neg
7171	11/29/13	F	25.0	BUGOYE	Neg
7172	11/29/13	F	30.0	KANYAN	Neg
7173	11/29/13	F	12.0	MURAMBA	Pos
7174	11/29/13	F	5.0	NDUGHUTU	Neg
7175	11/29/13	F	12.0	KISANZI	Neg
7176	11/29/13	F	.	MURAMBA	Pos
7177	11/29/13	M	40.0	BUNYANGONI	Neg
7178	11/29/13	M	3.9	MALIBA	Pos
7179	12/2/13	F	17.0	BUGOYE	Neg
7180	12/2/13	M	21.0	BUGOYE	Neg

7181	12/2/13	F	1.0	BUGOYE	Pos
7182	12/2/13	F	2.8	MURAMBA	Pos
7183	12/2/13	M	69.0	MURAMBA	Pos
7184	12/2/13	F	6.0	MALIBA	Pos
7185	12/2/13	F	4.0	MALIBA	Pos
7186	12/2/13	F	1.8	KANYAN	Pos
7187	12/2/13	F	47.0	IBANDA	Pos
7188	12/2/13	M	23.0	BUNYANGONI	Neg
7189	12/2/13	F	36.0	MURAMBA	Pos
7190	12/2/13	F	0.9	MUHAMBO	Neg
7191	12/2/13	M	5.0	BUNYANGONI	Neg
7192	12/2/13	M	4.0	MURAMBA	Neg
7193	12/2/13	F	8.5	MURAMBA	Pos
7194	12/2/13	M	12.0	KATUMBA	Pos
7195	12/2/13	F	18.0	MURAMBA	Pos
7196	12/2/13	F	40.0	BUGOYE	Neg
7197	12/2/13	M	5.0	IZINGA	Neg
7198	12/2/13	M	0.3	BUNYANGONI	Neg
7199	12/2/13	F	21.0	IZINGA	Pos
7200	12/2/13	F	0.4	BUNYANGONI	Neg
7201	12/2/13	M	1.7	BUNYANGONI	Pos
7202	12/2/13	M	18.0	NDUGHUTU	Neg
7203	12/2/13	F	0.1	KAKINDO	Neg
7204	12/2/13	F	4.0	IZINGA	Neg
7205	12/2/13	F	7.0	NDUGHUTU	Neg
7206	12/2/13	F	11.0	NDUGHUTU	Pos
7207	12/2/13	M	14.0	MURAMBA	Neg
7208	12/2/13	F	2.3	MURAMBA	Pos
7209	12/2/13	F	1.6	KYANYA	Neg
7210	12/2/13	F	1.0	BUNYANGONI	Pos
7211	12/2/13	F	3.0	BUNYANGONI	Neg
7212	12/2/13	F	42.0	MALIBA	Neg
7213	12/2/13	M	4.0	MALIBA	Neg
7214	12/2/13	F	18.0	MALIBA	Neg
7215	12/2/13	M	5.0	BUNYANGONI	Neg
7216	12/2/13	F	26.0	MURAMBA	Neg
7217	12/2/13	F	18.0	MAGHOMA	Pos
7218	12/2/13	F	17.0	MALIBA	Neg
7219	12/3/13	F	27.0	KISENDERA	Neg
7220	12/3/13	F	4.0	MALIBA	Pos
7221	12/3/13	F	38.0	KATUMBA	Pos
7222	12/3/13	F	29.0	BUGOYE	Neg
7223	12/3/13	M	3.0	MALIBA	Neg

7224	12/3/13	M	21.0	BUGOYE	Neg	
7225	12/3/13	M	14.0	IZINGA	Pos	
7226	12/3/13	M	30.0	MALIBA	Neg	
7227	12/3/13	F	20.0	BUGOYE	Neg	
7228	12/3/13	M	3.0	BUGOYE	Neg	
7229	12/3/13	M	0.4	MALIBA	Neg	
7230	12/3/13	M	15.0	NYABUSISI	Neg	
7231	12/3/13	M	15.0	KYARUGOMOKA	Neg	
7232	12/3/13	F	22.0	IZINGA	Pos	
7233	12/3/13	M	2.0	BUZIBWERA	Pos	
7234	12/3/13	F	0.9	MUHAMBO	Neg	
7235	12/3/13	F	7.0	MALIBA	Neg	
7236	12/3/13	F	17.0	MALIBA	Neg	
7237	12/3/13	M	2.0	BUGOYE	Pos	
7238	12/3/13	F	4.0	RWAKING	Neg	
7239	12/3/13	F	24.0	NDUGHUTU	Neg	
7240	12/3/13	F	29.0	KATOOKE	Neg	
7241	12/3/13	M	3.0	MURAMBA	Pos	
7242	12/3/13	F	16.0	IZINGA	Pos	
7243	12/3/13	M	2.0	MALIBA	Neg	
7244	12/3/13	M	12.0	BUHWEZA	Neg	
7245	12/3/13	F	37.0	MURAMBA	Neg	
7246	12/3/13	M	45.0	KANYAN	Neg	
7247	12/3/13	F	0.4	BUGOYE	Neg	
7248	12/3/13	M	9.0	IZINGA	Pos	
7249	12/3/13	F	20.0	MALIBA	Neg	
7250	12/3/13	M	21.0	BUGOYE	Neg	
7251	12/3/13	F	43.0	BUGOYE	Neg	
7252	12/3/13	F	20.0	MAGHOMA	Neg	
7253	12/3/13	F	37.0	KISANZI	Neg	
7254	12/3/13	M	78.0	IHANI	Pos	
7255	12/3/13	F	9.0	IBANDA	Pos	
7256	12/3/13	M	54.0	MALIBA	Neg	
7257	12/3/13	M	17.0	IZINGA	Neg	
7258	12/3/13	M	8.0	KYANYA	Pos	
7259	12/4/13	M	0.3	BUNYANGONI		Neg
7260	12/4/13	F	7.0	KANYAN	Pos	
7261	12/4/13	F	13.0	KIKOKERA	Neg	
7262	12/4/13	F	40.0	KYANYA	Neg	
7263	12/4/13	F	12.0	IBANDA	Neg	
7264	12/4/13	F	22.0	KANYAN	Neg	
7265	12/4/13	M	24.0	RWAKING	Neg	
7266	12/4/13	M	2.0	KIBIRIZI	Pos	

7267	12/4/13	M	1.9	IBANDA	Neg
7268	12/4/13	F	30.0	NDUGHUTU	Neg
7269	12/4/13	M	8.0	KANYAN	Pos
7270	12/4/13	F	31.0	BUNYANGONI	Pos
7271	12/4/13	F	1.6	BUNYANGONI	Neg
7272	12/4/13	F	5.5	BUNYANGONI	Pos
7273	12/4/13	M	2.5	BUNYANGONI	Pos
7274	12/4/13	F	13.0	MALIBA	Pos
7275	12/4/13	M	35.0	MURAMBA	Pos
7276	12/4/13	F	1.6	BULYANDEKE	Pos
7277	12/4/13	F	2.5	MALIBA	Pos
7278	12/4/13	M	54.0	MURAMBA	Pos
7279	12/4/13	M	7.0	IBANDA	Neg
7280	12/4/13	F	9.0	IBANDA	Neg
7281	12/4/13	F	1.1	MALIBA	Pos
7282	12/4/13	F	2.0	NDUGHUTU	Pos
7283	12/4/13	F	20.0	RWAKING	Neg
7284	12/4/13	M	1.5	KATUMBA	Neg
7285	12/4/13	F	70.0	IZINGA	Neg
7286	12/4/13	F	19.0	MALIBA	Neg
7287	12/4/13	F	35.0	MUHAMBO	Pos
7288	12/4/13	F	1.0	BUNYANGONI	Pos
7289	12/4/13	M	14.0	MURAMBA	Neg
7290	12/4/13	M	0.5	MURAMBA	Neg
7291	12/4/13	F	3.1	MALIBA	Neg
7292	12/4/13	M	0.3	BUNYANGONI	Neg
7293	12/4/13	M	4.0	MALIBA	Pos
7294	12/4/13	M	5.0	MURAMBA	Neg
7295	12/4/13	M	2.7	MURAMBA	Neg
7296	12/4/13	M	3.0	IHANI	Pos
7297	12/4/13	M	11.0	KANYAN	Pos
7298	12/4/13	M	15.0	MURAMBA	Neg
7299	12/4/13	F	2.1	BUNYANGONI	Pos
7300	12/4/13	M	0.3	BUHUNGA	Neg
7301	12/4/13	M	0.3	MURAMBA	Neg
7302	12/4/13	F	30.0	MURAMBA	Neg
7303	12/4/13	F	11.0	BUGOYE	Pos
7304	12/4/13	M	24.0	IBANDA	Neg
7305	12/4/13	F	1.3	BUGOYE	Neg
7306	12/4/13	M	1.3	KISENDERA	Pos
7307	12/4/13	F	28.0	KATOOKE	Neg
7308	12/5/13	F	7.0	BUZIBWERA	Pos
7309	12/5/13	F	22.0	IBANDA	Neg

7310	12/5/13	F	10.0	BUGOYE	Neg
7311	12/5/13	F	9.0	MALIBA	Neg
7312	12/5/13	F	10.0	BUZIBWERA	Pos
7313	12/5/13	F	70.0	NDUGHUTU	Neg
7314	12/5/13	M	30.0	BUGOYE	Neg
7315	12/5/13	F	82.0	IZINGA	Neg
7316	12/5/13	F	20.0	BUGOYE	Pos
7317	12/5/13	M	2.4	BUNYANGONI	Pos
7318	12/5/13	M	10.0	RWAKING	Pos
7319	12/5/13	F	60.0	MAGHOMA	Pos
7320	12/5/13	F	1.0	IZINGA	Neg
7321	12/5/13	M	4.0	IZINGA	Pos
7322	12/5/13	F	0.3	MURAMBA	Neg
7323	12/5/13	M	14.0	BULYANDEKE	Pos
7324	12/5/13	F	40.0	BULYANDEKE	Neg
7325	12/5/13	F	40.0	MURAMBA	Neg
7326	12/5/13	F	6.0	BULYANDEKE	Neg
7327	12/5/13	M	30.0	MALIBA	Pos
7328	12/5/13	F	19.0	BULYANDEKE	Neg
7329	12/5/13	F	34.0	IZINGA	Neg
7330	12/5/13	F	30.0	NYAKARUNGU	Neg
7331	12/5/13	F	14.0	NYAKARUNGU	Pos
7332	12/5/13	M	73.0	NDUGHUTU	Neg
7333	12/5/13	M	2.0	MALIBA	Pos
7334	12/5/13	M	4.0	NYAKARUNGU	Pos
7335	12/5/13	F	21.0	KATOOKE	Neg
7336	12/5/13	F	0.5	MURAMBA	Neg
7337	12/5/13	M	12.0	MALIBA	Pos
7338	12/5/13	F	26.0	MALIBA	Pos
7339	12/5/13	F	29.0	MALIBA	Neg
7340	12/5/13	F	30.0	KANYAN	Neg
7341	12/5/13	F	24.0	KANYAN	Pos
7342	12/5/13	M	4.0	BUNYANGONI	Pos
7343	12/5/13	M	8.0	BUNYANGONI	Neg
7344	12/5/13	F	38.0	BUZIBWERA	Neg
7345	12/5/13	F	1.5	MALIBA	Neg
7346	12/5/13	M	5.0	NYABISUSI	Neg
7347	12/5/13	F	6.0	BUZIBWERA	Pos
7348	12/5/13	F	45.0	KISAMBA	Neg
7349	12/5/13	M	7.0	KISAMBA	Neg
7350	12/5/13	F	1.1	BUGOYE	Neg
7351	12/5/13	F	20.0	IHANI	Neg
7352	12/5/13	M	19.0	KATOOKE	Neg

7353	12/5/13	F	20.0	MURAMBA	Neg
7354	12/5/13	F	7.0	KANYAN	Neg
7355	12/5/13	F	12.0	MURAMBA	Neg
7356	12/6/13	F	35.0	MURAMBA	Pos
7357	12/6/13	F	73.0	BUGOYE	Neg
7358	12/6/13	F	7.0	IZINGA	Neg
7359	12/6/13	M	5.0	IZINGA	Pos
7360	12/6/13	M	42.0	IZINGA	Neg
7361	12/6/13	F	3.0	MALIBA	Pos
7362	12/6/13	M	20.0	BUGOYE	Pos
7363	12/6/13	F	4.0	RWAKING	Neg
7364	12/6/13	F	7.0	MURAMBA	Neg
7365	12/6/13	M	2.3	BUZIBWERA	Pos
7366	12/6/13	M	0.4	IZINGA	Pos
7367	12/6/13	M	40.0	IBANDA	Neg
7368	12/6/13	M	22.0	MURAMBA	Neg
7369	12/6/13	F	20.0	MURAMBA	Neg
7370	12/6/13	F	6.0	MURAMBA	Neg
7371	12/6/13	F	3.0	BUGOYE	Pos
7372	12/6/13	F	21.0	BUGOYE	Pos
7373	12/6/13	F	35.0	BUNYANGONI	Neg
7374	12/6/13	M	13.0	MBUMURO	Neg
7375	12/6/13	F	49.0	NDUGHUTU	Neg
7376	12/6/13	M	13.0	MBUMURO	Neg
7377	12/6/13	F	2.0	KANYAN	Neg
7378	12/6/13	F	35.0	BUGOYE	Neg
7379	12/6/13	F	35.0	MURAMBA	Neg
7380	12/6/13	M	72.0	MURAMBA	Neg
7381	12/6/13	F	14.0	MBUMURO	Neg
7382	12/6/13	M	1.2	NYAKARUNGU	Neg
7383	12/6/13	F	3.8	BUGOYE	Neg
7384	12/6/13	M	0.3	KIRONGO	Neg
7385	12/6/13	M	16.0	BUGOYE	Neg
7386	12/6/13	M	15.0	KATUMBA	Neg
7387	12/6/13	F	2.8	BUZIBWERA	Pos
7388	12/6/13	F	32.0	MALIBA	Pos
7389	12/6/13	F	23.0	NDUGHUTU	Neg
7390	12/6/13	F	4.0	RWAKING	Neg
7391	12/6/13	M	20.0	MALIBA	Neg
7392	12/6/13	F	17.0	BUGOYE	Neg
7393	12/6/13	M	3.0	MALIBA	Pos
7394	12/6/13	F	3.0	IZINGA	Pos
7395	12/6/13	F	80.0	MURAMBA	Pos

7396	12/6/13	F	23.0	MURAMBA	Neg
7397	12/6/13	F	44.0	IZINGA	Pos
7398	12/6/13	F	48.0	BUGOYE	Pos
7399	12/6/13	M	1.5	BUGOYE	Pos
7400	12/6/13	F	2.0	BUGOYE	Neg
7401	12/6/13	F	1.5	IZINGA	Neg
7402	12/6/13	F	28.0	RWAKING	Neg
7403	12/6/13	M	1.1	RWAKING	Neg
7404	12/9/13	F	1.0	MURAMBA	Neg
7405	12/9/13	M	2.5	NDUGHUTU	Neg
7406	12/9/13	M	2.0	BUGOYE	Neg
7407	12/9/13	F	19.0	BUGOYE	Neg
7408	12/9/13	F	30.0	KANYAN	Neg
7409	12/9/13	M	56.0	BUNYANGONI	Neg
7410	12/9/13	F	22.0	KATOOKE	Neg
7411	12/9/13	F	18.0	NDUGHUTU	Neg
7412	12/9/13	M	19.0	MALIBA	Neg
7413	12/9/13	F	22.0	IZINGA	Pos
7414	12/9/13	F	9.0	IBANDA	Neg
7415	12/9/13	M	0.4	.	Neg
7416	12/9/13	M	6.0	MALIBA	Neg
7417	12/9/13	M	0.9	MALIBA	Neg
7418	12/9/13	M	3.0	RWAKING	Neg
7419	12/9/13	F	40.0	MURAMBA	Pos
7420	12/9/13	M	2.6	KYANYA	Neg
7421	12/9/13	F	3.0	KYANYA	Neg
7422	12/9/13	M	9.0	KIKOKERA	Pos
7423	12/9/13	F	1.8	MURAMBA	Neg
7424	12/9/13	M	2.0	BUNYANGONI	Neg
7425	12/9/13	M	5.0	MURAMBA	Pos
7426	12/9/13	F	3.0	MURAMBA	Neg
7427	12/9/13	F	34.0	MALIBA	Neg
7428	12/9/13	F	20.0	BUNYANGONI	Pos
7429	12/9/13	F	3.5	BUNYANGONI	Pos
7430	12/9/13	M	5.5	BUNYANGONI	Pos
7431	12/9/13	F	39.0	KIBIRIZI	Neg
7432	12/9/13	F	40.0	MURAMBA	Neg
7433	12/9/13	F	0.6	MUHAMBO	Neg
7434	12/9/13	F	22.0	MURAMBA	Pos
7435	12/9/13	F	8.0	RWAKING	Neg
7436	12/9/13	M	10.0	MURAMBA	Pos
7437	12/9/13	F	20.0	IHANI	Neg
7438	12/9/13	F	14.0	MALIBA	Neg

7439	12/9/13	F	20.0	RWAKING	Pos
7440	12/9/13	F	21.0	KANYAN	Neg
7441	12/9/13	F	26.0	NDUGHUTU	Neg
7442	12/9/13	F	9.0	IBANDA	Neg
7443	12/9/13	M	1.5	NDUGHUTU	Neg
7444	12/9/13	F	28.0	IHANI	Pos
7445	12/9/13	F	40.0	MURAMBA	Pos
7446	12/9/13	F	13.0	MALIBA	Pos
7447	12/9/13	F	3.3	KANYAN	Neg
7448	12/9/13	F	30.0	RWAKING	Pos
7449	12/9/13	F	30.0	KANYAN	Pos
7450	12/9/13	F	4.5	KANYAN	Pos
7451	12/9/13	F	4.0	BUNYANGONI	Neg
7452	12/9/13	M	43.0	MALIBA	Neg
7453	12/9/13	M	50.0	MALIBA	Neg
7454	12/9/13	F	5.0	IZINGA	Pos
7455	12/9/13	F	25.0	MURAMBA	Pos
7456	12/9/13	M	25.0	BUGOYE	Neg
7457	12/9/13	F	39.0	BUGOYE	Neg
7458	12/10/13	F	0.4	BUGOYE	Neg
7459	12/10/13	F	17.0	MURAMBA	Neg
7460	12/10/13	M	2.5	RWAKING	Neg
7461	12/10/13	M	5.0	RWAKING	Neg
7462	12/10/13	F	3.0	BUNYANGONI	Pos
7463	12/10/13	M	3.0	BUGOYE	Pos
7464	12/10/13	F	5.0	KAKINDO	Neg
7465	12/10/13	F	18.0	BUGOYE	Neg
7466	12/10/13	F	3.0	MURAMBA	Pos
7467	12/10/13	M	22.0	MURAMBA	Pos
7468	12/10/13	F	38.0	MURAMBA	Pos
7469	12/10/13	M	24.0	KANYAN	Neg
7470	12/10/13	F	29.0	MURAMBA	Neg
7471	12/10/13	M	10.0	MURAMBA	Pos
7472	12/10/13	F	6.0	KANYAN	Neg
7473	12/10/13	M	7.0	MALIBA	Pos
7474	12/10/13	F	8.0	MALIBA	Pos
7475	12/10/13	F	12.0	KANYAN	Neg
7476	12/10/13	M	22.0	BUGOYE	Neg
7477	12/10/13	F	31.0	BUGOYE	Pos
7478	12/10/13	F	19.0	KANYAN	Neg
7479	12/10/13	F	23.0	MURAMBA	Neg
7480	12/10/13	F	3.0	MUBUKU	Neg
7481	12/10/13	M	10.0	MURAMBA	Pos

7482	12/10/13	F	32.0	KISOJO	Pos	
7483	12/10/13		18.0	IZINGA	Pos	
7484	12/10/13	F	15.0	MURAMBA	Neg	
7485	12/10/13	F	22.0	NDUGHUTU	Neg	
7486	12/10/13		21.0	KANYAN	Neg	
7487	12/10/13	F	46.0	MURAMBA	Neg	
7488	12/10/13	M	12.0	BUGOYE	Pos	
7489	12/10/13	F	25.0	IZINGA	Neg	
7490	12/10/13	F	18.0	.	Neg	
7491	12/10/13	F	15.0	IZINGA	Pos	
7492	12/10/13	F	18.0	IBANDA	Neg	
7493	12/10/13	F	19.0	IZINGA	Neg	
7494	12/10/13	F	18.0	BUZIBWERA	Neg	
7495	12/10/13	M	40.0	IBANDA	Pos	
7496	12/12/13	F	3.7	RWAKING	Neg	Neg
7497	12/12/13	M	1.7	KISENDERA	Neg	Neg
7498	12/12/13	F	33.0	RWAKING	Neg	
7499	12/12/13	F	3.0	BUNYANGONI		Pos
7500	12/12/13	M	1.0	NDUGHUTU		Neg
7501	12/12/13	F	50.0	KANYAN	Neg	
7502	12/12/13	F	4.0	BUGOYE	Neg	
7503	12/12/13	F	1.7	KANYAN	Neg	
7504	12/12/13	F	29.0	MURAMBA	Pos	
7505	12/12/13	F	0.5	NYAKARUNGU	Neg	
7506	12/12/13	M	20.0	KISAMBA	Neg	
7507	12/12/13	F	15.0	MUHAMBO	Pos	
7508	12/12/13	F	9.0	BUZIBWERA	Neg	
7509	12/12/13	F	37.0	MURAMBA	Neg	
7510	12/12/13	F	1.5	MWARU	Pos	
7511	12/12/13	M	0.6	KANYAN	Neg	
7512	12/12/13	F	9.0	RWAKING	Neg	
7513	12/12/13	F	0.3	MWARU	Neg	
7514	12/12/13	F	40.0	MAGHOMA	Neg	
7515	12/12/13	F	39.0	RWAKING	Neg	
7516	12/12/13	M	24.0	KANYAN	Pos	
7517	12/12/13	M	6.0	KANYAN	Neg	
7518	12/12/13	M	20.0	KISAMBA	Neg	
7519	12/12/13	F	12.0	MURAMBA	Pos	
7520	12/12/13	F	10.0	KISENDERA	Pos	
7521	12/12/13	F	65.0	KATOOKE	Neg	
7522	12/12/13	F	30.0	MALIBA	Neg	
7523	12/12/13	F	0.9	MALIBA	Pos	
7524	12/12/13	F	58.0	MALIBA	Neg	

7525	12/12/13	F	33.0	KEGUZA	Neg
7526	12/12/13	F	2.5	KANYAN	Neg
7527	12/12/13	F	22.0	RWAKING	Pos
7528	12/12/13	F	62.0	BUZIBWERA	Neg
7529	12/12/13	F	0.4	RWAKING	Neg
7530	12/12/13	F	29.0	KAKINDO	Neg
7531	12/12/13	F	0.3	KISENDERA	Pos
7532	12/12/13	F	5.0	IHANI	Neg
7533	12/12/13	M	1.5	BUNYANGONI	Neg
7534	12/12/13	M	0.4	NDUGHUTU	Neg
7535	12/12/13	F	5.0	BUGOYE	Neg
7536	12/12/13	M	1.0	MALIBA	Pos
7537	12/12/13	F	25.0	BUGOYE	Neg
7538	12/12/13	F	0.8	BUNYANGONI	Neg
7539	12/12/13	F	52.0	BUGOYE	Neg
7540	12/12/13	F	31.0	BUGOYE	Neg
7541	12/12/13	F	18.0	KIRONGO	Neg
7542	12/12/13	M	30.0	MURAMBA	Pos
7543	12/12/13	F	17.0	KIKOKERA	Neg
7544	12/12/13	F	9.0	IZINGA	Pos
7545	12/12/13	M	11.0	IZINGA	Neg
7546	12/12/13	F	3.3	KANYAN	Neg
7547	12/13/13	F	50.0	MURAMBA	Neg
7548	12/13/13	F	45.0	KYARUGOMOKA	Neg
7549	12/13/13	F	50.0	MURAMBA	Neg
7550	12/13/13	F	46.0	MURAMBA	Pos
7551	12/13/13	F	30.0	NDUGHUTU	Neg
7552	12/13/13	F	1.8	MALIBA	Neg
7553	12/13/13	M	12.0	KISANZI	Neg
7554	12/13/13	M	10.0	KISANZI	Pos
7555	12/13/13	F	17.0	MALIBA	Pos
7556	12/13/13	M	1.5	BUHUNGA	Neg
7557	12/13/13	F	1.1	BUGOYE	Neg
7558	12/13/13	M	53.0	NDUGHUTU	Neg
7559	12/13/13	F	23.0	MALIBA	Pos
7560	12/13/13	F	30.0	MALIBA	Neg
7561	12/13/13	F	1.9	RWAKING	Pos
7562	12/13/13	F	19.0	RWAKING	Neg
7563	12/13/13	F	46.0	MURAMBA	Neg
7564	12/13/13	F	4.0	MURAMBA	Neg
7565	12/13/13	M	7.0	MALIBA	Pos
7566	12/13/13	M	6.0	BUNYANGONI	Neg
7567	12/13/13	M	0.8	IZINGA	Neg

7568	12/13/13	M	.	MURAMBA	Neg
7569	12/13/13	F	6.0	KYANYA	Pos
7570	12/13/13	F	4.0	KYANYA	Neg
7571	12/13/13	F	17.0	NDUGHUTU	Pos
7572	12/13/13	F	23.0	KYANYA	Neg
7573	12/13/13	M	2.9	KYANYA	Neg
7574	12/13/13	F	32.0	IZINGA	Neg
7575	12/13/13	F	50.0	KYANYA	Neg
7576	12/13/13	M	1.0	IZINGA	Pos
7577	12/13/13	F	47.0	IZINGA	Neg
7578	12/13/13	F	9.0	IZINGA	Pos
7579	12/13/13	F	6.0	KYARUGOMOKA	Pos
7580	12/13/13	M	10.0	MUBUKU	Neg
7581	12/13/13	F	5.0	RWAKING	Neg
7582	12/13/13	F	10.0	MALIBA	Pos
7583	12/13/13	F	2.5	MALIBA	Pos
7584	12/13/13	F	27.0	MAGHOMA	Neg
7585	12/13/13	M	18.0	MURAMBA	Neg
7586	12/13/13	M	1.5	BUNYANGONI	Pos
7587	12/13/13	F	30.0	KIBIRIZI	Neg
7588	12/13/13	M	9.0	KANYAN	Neg
7589	12/13/13	F	.	NDUGHUTU	Neg
7590	12/13/13	F	13.0	MURAMBA	Pos
7591	12/13/13	F	5.0	RWAKING	Pos
7592	12/13/13	M	2.5	KISENDERA	Pos
7593	12/13/13	F	13.0	BUGOYE	Neg
7594	12/13/13	F	22.0	BUNYANGONI	Neg
7595	12/13/13	F	52.0	KANYAN	Neg
7596	12/13/13	F	1.0	ISULE	Neg
7597	12/13/13	F	49.0	ISULE	Neg
7598	12/13/13	F	18.0	IZINGA	Neg
7599	12/13/13	F	2.3	MAGHOMA	Neg
7600	12/13/13	F	4.0	KYARUGOMOKA	Pos
7601	12/13/13	F	23.0	KYARUGOMOKA	Neg
7602	12/13/13	F	36.0	MURAMBA	Neg
7603	12/13/13	F	3.0	MUHAMBO	Neg
7604	12/13/13	M	4.0	NDUGHUTU	Neg
7605	12/13/13	M	30.0	KATUMBA	Neg
7606	12/13/13	M	3.5	NDUGHUTU	Neg
7607	12/13/13	F	7.0	NDUGHUTU	Neg
7608	12/13/13	F	8.0	IZINGA	Neg
7609	12/13/13	F	13.0	IZINGA	Pos
7610	12/13/13	F	3.0	RWAKING	Neg

7611	12/13/13	M	17.0	KIKOKERA	Pos	
7612	12/13/13	F	18.0	BUGOYE	Neg	
7613	12/13/13	F	6.0	BUGOYE	Neg	
7614	12/13/13	F	10.0	BUGOYE	Neg	
7615	12/13/13	M	55.0	BUGOYE	Neg	
7616	12/13/13	M	21.0	MALIBA	Pos	
7617	12/13/13	M	7.0	BUGOYE	Pos	
7618	12/13/13	M	51.0	KYANYA	Pos	
7619	12/13/13	M	55.0	MURAMBA	Pos	
7620	12/13/13	M	3.3	BUNYANGONI	Pos	
7621	12/13/13	F	47.0	RWAKING	Neg	
7622	12/16/13	F	21.0	KISENDERA	Neg	
7623	12/16/13	F	18.0	BUZIBWERA	Neg	
7624	12/16/13	F	17.0	MALIBA	Pos	
7625	12/16/13	M	21.0	BULINDUGURU	Neg	
7626	12/16/13	F	50.0	KAKINDO	Pos	
7627	12/16/13	F	7.0	KIBOTA	Neg	
7628	12/16/13	F	35.0	KIBOTA	Neg	
7629	12/16/13	F	7.0	MALIBA	Neg	
7630	12/16/13	F	0.4	NDUGHUTU	Neg	
7631	12/16/13	M	24.0	MURAMBA	Neg	
7632	12/16/13	M	13.0	KAKINDO	Neg	
7633	12/16/13	M	8.0	MURAMBA	Neg	
7634	12/16/13	M	7.0	MURAMBA	Neg	
7635	12/16/13	M	16.0	MURAMBA	Pos	
7636	12/16/13	M	1.0	BUGOYE	Neg	
7637	12/16/13	F	1.8	KABUGHO	Pos	Pos
7638	12/16/13	F	17.0	NYAKABUGHA	Neg	
7639	12/16/13	M	24.0	KIRULI	Pos	
7640	12/16/13	M	3.0	BUGOYE	Neg	
7641	12/16/13	M	12.0	KISANZI	Pos	
7642	12/16/13	F	13.0	MURAMBA	Neg	
7643	12/16/13	F	11.0	MURAMBA	Pos	
7644	12/16/13	F	17.0	BUGOYE	Neg	
7645	12/16/13	F	0.7	MURAMBA	Neg	
7646	12/16/13	M	73.0	KANYAN	Neg	
7647	12/16/13	F	19.0	RWAKING	Neg	
7648	12/16/13	M	7.0	BUGOYE	Neg	
7649	12/16/13	F	21.0	BUGOYE	Neg	
7650	12/16/13	F	50.0	MURAMBA	Pos	
7651	12/16/13	M	22.0	.	Neg	
7652	12/16/13	F	3.0	KAKINDO	Pos	
7653	12/16/13	F	39.0	BUGOYE	Neg	

7654	12/16/13	F	12.0	IZINGA	Pos
7655	12/16/13	F	2.5	BUNYANGONI	Neg
7656	12/16/13	M	1.5	MAGHOMA	Neg
7657	12/16/13	F	19.0	RWAKING	Neg
7658	12/16/13	F	45.0	MUHAMBO	Neg
7659	12/16/13	F	14.0	NDUGHUTU	Pos
7660	12/16/13	F	12.0	KAKINDO	Pos
7661	12/16/13	F	20.0	MURAMBA	Pos
7662	12/16/13	M	12.0	BUGOYE	Neg
7663	12/16/13	M	33.0	MURAMBA	Neg
7664	12/16/13	M	12.0	KANYAN	Neg
7665	12/16/13	F	17.0	MURAMBA	Neg
7666	12/16/13	F	25.0	MALIBA	Neg
7667	12/16/13	F	37.0	MURAMBA	Neg
7668	12/16/13	F	20.0	IHANI	Neg
7669	12/16/13	M	22.0	MURAMBA	Neg
7670	12/16/13	F	30.0	MUHAMBO	Neg
7671	12/16/13	F	23.0	IZINGA	Neg
7672	12/16/13	F	15.0	BUGOYE	Neg
7673	12/16/13	M	21.0	KIBIRIZI	Neg
7674	12/17/13	F	45.0	BUGOYE	Neg
7675	12/17/13	F	3.0	BIKONE	Neg
7676	12/17/13	F	2.0	KANYAN	Neg
7677	12/17/13	M	15.0	KAKINDO	Pos
7678	12/17/13	M	9.0	KAKINDO	Pos
7679	12/17/13	F	20.0	MURAMBA	Pos
7680	12/17/13	M	11.0	KAKINDO	Pos
7681	12/17/13	M	19.0	MURAMBA	Neg
7682	12/17/13	M	60.0	BIKONE	Neg
7683	12/17/13	F	35.0	KANYAN	Pos
7684	12/17/13	F	50.0	RWAKING	Neg
7685	12/17/13	M	13.0	.	Pos
7686	12/17/13	M	12.0	MALIBA	Pos
7687	12/17/13	M	27.0	NDUGHUTU	Pos
7688	12/17/13	F	10.0	MALIBA	Neg
7689	12/17/13	M	12.0	MURAMBA	Neg
7690	12/17/13	F	41.0	MURAMBA	Neg
7691	12/17/13	F	15.0	IZINGA	Pos
7692	12/17/13	F	17.0	NDUGHUTU	Neg
7693	12/17/13	F	0.5	MALIBA	Neg
7694	12/17/13	F	17.0	BUGOYE	Neg
7695	12/17/13	F	1.0	BUNYANGONI	Neg
7696	12/17/13	F	10.0	MURAMBA	Pos

7697	12/17/13	F	40.0	MAGHOMA	Neg	
7698	12/17/13	M	22.0	KYANYA	Pos	
7699	12/17/13	M	17.0	BUGOYE	Pos	
7700	12/17/13	F	29.0	BUGOYE	Pos	Pos
7701	12/17/13	M	6.0	BUGOYE	Neg	
7702	12/17/13	M	7.0	MURAMBA	Neg	
7703	12/17/13	M	42.0	MURAMBA	Neg	
7704	12/17/13	M	5.0	MURAMBA	Neg	
7705	12/17/13	F	23.0	MURAMBA	Neg	
7706	12/17/13	F	22.0	NYANGONGE	Neg	
7707	12/17/13	F	22.0	NDUGHUTU	Neg	
7708	12/17/13	F	7.0	BUGOYE	Neg	
7709	12/17/13	F	0.1	KATOOKE	Neg	
7710	12/17/13	F	25.0	MAGHOMA	Neg	
7711	12/17/13	F	1.0	MURAMBA	Neg	
7712	12/17/13	M	6.0	MURAMBA	Neg	
7713	12/17/13	F	12.0	MUHAMBO	Pos	
7714	12/17/13	M	12.0	BUGOYE	Neg	
7715	12/18/13	F	4.0	NDUGHUTU	Neg	
7716	12/18/13	F	43.0	IZINGA	Neg	
7717	12/18/13	F	29.0	MALIBA	Neg	
7718	12/18/13		59.0	IBANDA	Neg	
7719	12/18/13	F	35.0	KATUMBA	Neg	
7720	12/18/13	M	17.0	KATUMBA	Neg	
7721	12/18/13	F	3.5	MURAMBA	Pos	
7722	12/18/13	F	33.0	MURAMBA	Pos	
7723	12/18/13	F	32.0	KATOOKE	Neg	
7724	12/18/13	F	70.0	NDUGHUTU	Neg	
7725	12/18/13	M	1.1	KANYAN	Neg	
7726	12/18/13	M	70.0	BUGOYE	Neg	
7727	12/18/13	F	4.1	MURAMBA	Neg	
7728	12/18/13	F	38.0	BUNYANGONI	Neg	
7729	12/18/13	M	2.0	KANYAN	Neg	
7730	12/18/13	M	2.0	BUNYANGONI	Pos	
7731	12/18/13	M	38.0	KATOOKE	Neg	
7732	12/18/13	F	20.0	NYAKALENGIJO	Neg	
7733	12/18/13	F	12.0	MURAMBA	Pos	
7734	12/18/13	M	4.0	KISOJO	Pos	
7735	12/19/13	M	26.0	MURAMBA	Pos	
7736	12/19/13	M	3.0	NDUGHUTU	Pos	
7737	12/19/13	M	14.0	KANYAN	Pos	
7738	12/19/13	F	12.0	MURAMBA	Neg	
7739	12/19/13	M	3.1	KISANGA	Neg	

7740	12/19/13	M	9.0	.	Pos
7741	12/19/13	F	2.5	.	Neg
7742	12/19/13	F	4.0	.	Neg
7743	12/19/13	F	1.8	.	Neg
7744	12/19/13	F	29.0	.	Neg
7745	12/19/13	F	20.0	.	Neg
7746	12/19/13	F	39.0	BUGOYE	Neg
7747	12/19/13	M	0.2	BUNYANGONI	Pos
7748	12/19/13	F	50.0	BUNYANGONI	Neg
7749	12/19/13	F	3.0	BUGOYE	Neg
7750	12/19/13	M	0.6	MURAMBA	Neg
7751	12/19/13	F	.	RWAKING	Neg
7752	12/19/13	F	25.0	BUGOYE	Neg
7753	12/19/13	F	20.0	BUGOYE	Neg
7754	12/19/13	M	40.0	KANYAN	Neg
7755	12/19/13	M	60.0	MALIBA	Neg
7756	12/19/13	M	18.0	BUGOYE	Pos
7757	12/19/13	M	8.0	KANYAN	Pos
7758	12/19/13	F	0.3	BUGOYE	Neg
7759	12/19/13	F	12.0	.	Neg
7760	12/19/13	F	9.0	.	Neg
7761	12/19/13	M	5.0	.	Neg
7762	12/19/13	F	0.6	.	Pos
7763	12/19/13	M	2.2	.	Pos
7764	12/19/13	F	18.0	MALIBA	Pos
7765	12/19/13	M	16.0	IZINGA	Pos
7766	12/20/13	F	14.0	RWAKING	Pos
7767	12/20/13	F	35.0	BUGOYE	Neg
7768	12/20/13	M	8.0	BUGOYE	Neg
7769	12/20/13	M	21.0	KIRONGO	Pos
7770	12/20/13	F	0.8	KINYANGONGE	Neg
7771	12/20/13	F	30.0	.	Pos
7772	12/20/13	F	18.0	NYAKALENGIJO	Neg
7773	12/20/13	M	5.0	NDUGHUTU	Neg
7774	12/20/13	F	28.0	NYAKALENGIJO	Neg
7775	12/20/13	F	40.0	KANYAN	Pos
7776	12/20/13	F	0.4	NDUGHUTU	Neg
7777	12/20/13	M	10.0	MUHAMBO	Neg
7778	12/20/13	M	0.5	MUHAMBO	Neg
7779	12/20/13	F	24.0	NDUGHUTU	Neg
7780	12/20/13	F	28.0	BUNYANGONI	Pos
7781	12/20/13	F	37.0	IZINGA	Neg
7782	12/20/13	M	22.0	KANYAN	Pos

7783	12/20/13	F	8.0	KANYAN	Pos	
7784	12/20/13	F	9.0	MURAMBA	Neg	
7785	12/20/13	M	5.0	MURAMBA	Neg	
7786	12/20/13	F	24.0	BUNYANGONI	Neg	
7787	12/20/13	F	23.0	KIBIRIZI	Neg	
7788	12/20/13	F	20.0	BUGOYE	Pos	
7789	12/21/13	F	6.0	KISOJO	Pos	
7790	12/21/13	F	36.0	KISOJO	Neg	
7791	12/21/13	M	8.0	MALIBA	Neg	
7792	12/21/13	M	9.0	MALIBA	Neg	
7793	12/21/13	M	1.9	IBANDA	Neg	
7794	12/21/13	F	0.6	KIKOKERA	Neg	
7795	12/21/13	F	21.0	BUGOYE	Pos	
7796	12/21/13	F	25.0	BUGOYE	Neg	
7797	12/21/13	M	2.0	BUNYANGONI	Neg	
7798	12/21/13	F	37.0	BUNYANGONI	Neg	
7799	12/21/13	F	8.0	BUNYANGONI	Neg	
7800	12/21/13	F	2.3	MURAMBA	Neg	
7801	12/22/13	F	23.0	MURAMBA	Pos	
7802	12/22/13	F	22.0	KATOOKE	Neg	
7803	12/22/13	F	46.0	KIBIRIZI	Neg	
7804	12/22/13	F	28.0	MURAMBA	Neg	
7805	12/22/13	F	35.0	MURAMBA	Neg	
7806	12/22/13	F	33.0	IBANDA	Neg	
7807	12/22/13	F	39.0	BUGOYE	Neg	
7808	12/22/13	F	55.0	MURAMBA	Pos	
7809	12/23/13	M	2.5	BUZIBWERA	Pos	
7810	12/23/13	F	8.0	KYARUGOMOKA	Neg	
7811	12/23/13	F	4.5	KYARUGOMOKA	Pos	
7812	12/23/13	F	0.4	KISENDERA	Neg	
7813	12/23/13	F	0.6	MURAMBA	Neg	
7814	12/23/13	M	1.4	BIKONE	Neg	
7815	12/23/13	F	15.0	BUGOYE	Pos	
7816	12/23/13	M	24.0	IZINGA	Neg	
7817	12/23/13	F	0.2	NDUGHUTU	Neg	
7818	12/23/13	F	23.0	IBANDA	Pos	
7819	12/23/13	F	1.0	MURAMBA	Pos	
7820	12/23/13	M	2.2	NDUGHUTU	Neg	
7821	1/2/14	F	30.0	KIKOKERA	Neg	
7822	1/2/14	F	30.0	RWAKING	Pos	
7823	1/2/14	M	1.2	KYARUGOMOKA	Pos	Pos
7824	1/2/14	F	0.3	MURAMBA	Pos	
7825	1/2/14	F	4.0	MALIBA	Pos	

7826	1/2/14	M	32.0	NDUGHUTU	Neg	
7827	1/2/14	F	4.0	MALIBA	Pos	
7828	1/2/14	M	1.0	MALIBA	Neg	
7829	1/2/14	M	31.0	KANYAN	Neg	
7830	1/2/14	M	17.0	BUGOYE	Pos	
7831	1/2/14	F	45.0	NYAKARUNGU	Neg	
7832	1/2/14	F	44.0	MALIBA	Pos	
7833	1/2/14	F	5.0	MALIBA	Pos	
7834	1/2/14	F	0.5	MAGHOMA	Neg	
7835	1/2/14	M	0.2	KIRONGO	Neg	
7836	1/3/14	F	40.0	NDUGHUTU		Pos
7837	1/3/14	F	26.0	KYANYA	Pos	
7838	1/3/14	M	3.5	IBANDA	Pos	
7839	1/3/14	F	19.0	KIKOKERA	Neg	
7840	1/3/14	M	4.0	MALIBA	Pos	
7841	1/3/14	F	17.0	BUGOYE	Pos	
7842	1/3/14	F	2.0	BUGOYE	Pos	
7843	1/3/14	M	22.0	BUGOYE	Neg	
7844	1/3/14	M	0.6	.	Neg	
7845	1/3/14	M	50.0	NDUGHUTU	Neg	
7846	1/3/14	M	55.0	KANYAN	Pos	
7847	1/3/14	F	3.5	BUGOYE	Pos	
7848	1/3/14	M	30.0	KANYAN	Pos	
7849	1/3/14	M	28.0	RWAKING	Neg	
7850	1/3/14	F	40.0	NYAKARUNGU	Neg	
7851	1/3/14	F	35.0	NDUGHUTU	Neg	
7852	1/3/14	F	14.0	KIKOKERA	Pos	
7853	1/6/14	F	70.0	MUHAMBO	Neg	
7854	1/6/14	M	9.0	BUGOYE	Neg	
7855	1/6/14	F	40.0	IZINGA	Neg	
7856	1/6/14	F	0.3	NDUGHUTU	Neg	
7857	1/6/14	F	30.0	MALIBA	Neg	
7858	1/6/14	M	11.0	MALIBA	Neg	
7859	1/6/14	F	6.0	IZINGA	Neg	
7860	1/6/14	M	36.0	NDUGHUTU	Neg	
7861	1/6/14	M	0.8	BUGOYE	Neg	
7862	1/6/14	M	4.0	KISAMBA	Neg	
7863	1/6/14	M	1.8	MURAMBA	Neg	
7864	1/6/14	F	6.0	KYANYA	Pos	
7865	1/6/14	F	37.0	IBANDA	Neg	
7866	1/6/14	F	12.0	IBANDA	Pos	
7867	1/6/14	F	8.0	IBANDA	Neg	
7868	1/6/14	F	4.0	NDUGHUTU	Neg	

7869	1/6/14	F	11.0	MURAMBA	Pos	
7870	1/6/14	F	31.0	NYAKABUGHA	Pos	
7871	1/6/14	F	3.0	KIRULI	Pos	
7872	1/6/14	M	2.0	BUNYANGONI	Pos	
7873	1/6/14	M	0.3	MALIBA	Neg	
7874	1/6/14	F	3.0	KANYAN	Pos	
7875	1/6/14	M	10.0	IBANDA	Neg	
7876	1/6/14	M	27.0	MURAMBA	Neg	
7877	1/6/14	M	4.1	IBANDA	Neg	
7878	1/6/14	F	4.0	NDUGHUTU	Neg	
7879	1/6/14	F	2.0	MURAMBA	Neg	Neg
7880	1/6/14	M	13.0	KANYAN	Neg	Neg
7881	1/6/14	F	27.0	KATUMBA	Neg	
7882	1/6/14	M	56.0	BUNYANGONI	Neg	
7883	1/6/14	F	7.0	MURAMBA	Neg	
7884	1/6/14	M	22.0	MALIBA	Neg	
7885	1/6/14	F	23.0	NYANGONGE	Neg	
7886	1/6/14	F	1.0	MURAMBA	Pos	Pos
7887	1/6/14	F	26.0	MALIBA	Neg	
7888	1/6/14	M	44.0	IBANDA	Neg	
7889	1/6/14	F	2.2	MURAMBA	Pos	
7890	1/6/14	F	50.0	BUGOYE	Pos	
7891	1/6/14	M	67.0	BUGOYE	Neg	
7892	1/6/14	M	10.0	.	Pos	
7893	1/6/14	M	10.0	KISENDERA	Pos	
7894	1/6/14	M	1.8	MALIBA	Pos	
7895	1/6/14	F	21.0	BUNYANGONI	Neg	
7896	1/6/14	F	2.0	KIBIRIZI	Neg	
7897	1/6/14	M	42.0	KINYOKE	Neg	
7898	1/6/14	M	3.5	NYAKALENGIJO	Pos	
7899	1/6/14	F	8.0	KANYAN	Pos	
7900	1/6/14	F	2.0	MURAMBA	Neg	
7901	1/6/14	M	10.0	BUNYANGONI	Pos	
7902	1/6/14	M	67.0	MURAMBA	Neg	
7903	1/6/14	F	18.0	NDUGHUTU	Pos	
7904	1/6/14	F	17.0	MURAMBA	Neg	
7905	1/6/14	F	1.0	MURAMBA	Pos	
7906	1/6/14	F	4.0	MURAMBA	Pos	
7907	1/6/14	F	80.0	BUGOYE	Neg	
7908	1/6/14	M	7.0	NDUGHUTU	Pos	
7909	1/6/14	F	58.0	KAKINDO	Neg	
7910	1/6/14	F	1.8	NDUGHUTU	Pos	
7911	1/6/14	F	7.0	BUGOYE	Pos	

7912	1/6/14	M	0.4	MURAMBA	Neg	
7913	1/6/14	M	22.0	BUGOYE	Pos	
7914	1/7/14	F	19.0	MALIBA	Neg	
7915	1/7/14	F	67.0	BUNYANGONI	Pos	
7916	1/7/14	M	4.0	BUNYANGONI	Neg	
7917	1/7/14	F	2.3	RWAKING	Neg	
7918	1/7/14	F	13.0	KYARUGOMOKA	Neg	
7919	1/7/14	M	9.0	NDUGHUTU	Neg	
7920	1/7/14	M	8.0	MURAMBA	Pos	
7921	1/7/14	M	8.0	KYARUGOMOKA	Neg	
7922	1/7/14	M	11.0	KYARUGOMOKA	Pos	
7923	1/7/14	F	7.0	IZINGA	Pos	Neg
7924	1/7/14	M	35.0	KIKOKERA	Neg	
7925	1/7/14	M	8.0	KIKOKERA	Neg	
7926	1/7/14	M	19.0	MURAMBA	Pos	
7927	1/7/14	F	25.0	RWAKING	Neg	
7928	1/7/14	F	5.0	RWAKING	Neg	
7929	1/7/14	F	21.0	KANYAN	Neg	
7930	1/7/14	M	55.0	KIBIRIZI	Pos	
7931	1/7/14	M	1.1	IZINGA	Neg	
7932	1/7/14	F	10.0	KISENDERA	Pos	
7933	1/7/14	F	35.0	IZINGA	Neg	
7934	1/7/14	M	69.0	MURAMBA	Neg	
7935	1/7/14	M	40.0	KISANZI	Pos	
7936	1/7/14	F	24.0	BUGOYE	Neg	
7937	1/7/14	F	18.0	NDUGHUTU	Pos	
7938	1/7/14	F	7.0	NYAKARUNGU	Neg	
7939	1/7/14	M	7.0	NYAKARUNGU	Pos	
7940	1/7/14	F	27.0	IBANDA	Neg	
7941	1/7/14	F	17.0	BUGOYE	Pos	
7942	1/7/14	M	74.0	RWAKING	Pos	
7943	1/7/14	M	37.0	MUBUKU	Neg	
7944	1/7/14	F	40.0	MALIBA	Neg	
7945	1/7/14	F	8.0	KANYAN	Pos	
7946	1/7/14	M	2.7	IZINGA	Pos	
7947	1/7/14	F	34.0	IZINGA	Neg	
7948	1/7/14	F	6.0	MURAMBA	Pos	
7949	1/7/14	F	5.0	RWAKING	Neg	
7950	1/7/14	M	0.8	IZINGA	Pos	
7951	1/7/14	F	30.0	KYARUGOMOKA	Neg	
7952	1/8/14	F	2.0	MALIBA	Neg	
7953	1/8/14	F	0.7	NDUGHUTU	Neg	
7954	1/8/14	M	28.0	KYANYA	Neg	

7955	1/8/14	F	15.0	MURAMBA	Pos	
7956	1/8/14	M	18.0	MURAMBA		Pos
7957	1/8/14	F	22.0	NDUGHUTU	Pos	
7958	1/8/14	F	11.0	BUGOYE	Neg	
7959	1/8/14	F	12.0	MURAMBA	Pos	
7960	1/8/14	F	20.0	BUGOYE	Neg	
7961	1/8/14	F	54.0	BUNYANGONI	Neg	
7962	1/8/14	F	1.3	MURAMBA		Neg
7963	1/8/14	M	30.0	KYANYA	Neg	Neg
7964	1/8/14	F	24.0	KYANYA	Pos	
7965	1/8/14	F	21.0	MUHAMBO	Pos	
7966	1/8/14	M	1.0	BUGOYE	Neg	
7967	1/8/14	M	1.0	BUGOYE	Neg	
7968	1/8/14	F	5.0	MURAMBA		Pos
7969	1/8/14	F	35.0	MALIBA	Pos	
7970	1/8/14	F	20.0	KYANYA	Neg	
7971	1/8/14	F	1.0	KYANYA	Neg	
7972	1/8/14	F	22.0	IZINGA	Neg	
7973	1/8/14	M	9.0	KAKINDO	Neg	
7974	1/8/14	M	16.0	KAKINDO	Pos	
7975	1/8/14	F	45.0	IZINGA	Pos	
7976	1/8/14	F	4.0	IZINGA	Pos	
7977	1/8/14	M	7.0	IZINGA	Neg	
7978	1/8/14	F	64.0	IBANDA	Neg	
7979	1/8/14	M	17.0	MALIBA	Pos	
7980	1/8/14	F	18.0	NDUGHUTU	Neg	
7981	1/8/14	F	25.0	KULUNEEDE	Neg	
7982	1/8/14	M	35.0	BUGOYE	Neg	
7983	1/8/14	M	19.0	MURAMBA	Pos	
7984	1/8/14	F	0.9	KYANYA	Pos	
7985	1/8/14	F	18.0	MURAMBA	Pos	
7986	1/8/14	M	2.6	MURAMBA	Pos	
7987	1/9/14	F	40.0	KANYAN	Neg	
7988	1/9/14	F	36.0	RWAKING	Neg	
7989	1/9/14	F	49.0	KANYAN	Pos	
7990	1/9/14	F	0.1	IHANI	Neg	
7991	1/9/14	F	55.0	NYANGONGE	Neg	
7992	1/9/14	M	1.1	IBANDA	Neg	
7993	1/9/14	M	41.0	IZINGA	Neg	
7994	1/9/14	M	14.0	IZINGA	Pos	
7995	1/9/14	F	1.0	BUNYANGONI	Pos	
7996	1/9/14	F	12.0	BUNYANGONI	Pos	
7997	1/9/14	M	32.0	NDUGHUTU	Neg	

7998	1/9/14	F	50.0	KANYAN	Pos	
7999	1/9/14	F	8.0	BIKONE	Neg	
8000	1/9/14	M	1.0	MALIBA	Neg	
8001	1/9/14	F	38.0	RWAKING	Pos	
8002	1/9/14	M	6.0	IZINGA	Neg	
8003	1/9/14	F	1.8	BUGOYE	Neg	
8004	1/9/14	F	17.0	KANYAN	Pos	
8005	1/9/14	F	6.0	IZINGA	Pos	
8006	1/9/14	F	10.0	BUZIBWERA	Pos	
8007	1/9/14	M	18.0	IZINGA	Neg	
8008	1/9/14	M	0.2	MUHAMBO	Neg	
8009	1/9/14	M	14.0	BUGOYE	Pos	
8010	1/10/14	M	42.0	BUGOYE	Neg	
8011	1/10/14	F	70.0	IBANDA	Neg	
8012	1/10/14	M	64.0	NDUGHUTU	Neg	
8013	1/10/14	M	24.0	NDUGHUTU	Pos	Pos
8014	1/10/14	F	80.0	BUGOYE	Neg	
8015	1/10/14	F	3.0	KYANYA	Pos	Pos
8016	1/10/14	F	28.0	KYANYA	Pos	
8017	1/10/14	M	3.0	BUNYANGONI	Neg	
8018	1/10/14	F	6.0	BUNYANGONI	Neg	
8019	1/10/14	F	31.0	BUNYANGONI	Neg	
8020	1/10/14	F	8.0	BUGOYE	Pos	Pos
8021	1/10/14	M	2.0	MUHAMBO	Neg	
8022	1/10/14	F	44.0	MURAMBA	Neg	Pos
8023	1/10/14	F	90.0	MURAMBA	Pos	Pos
8024	1/10/14	F	8.0	RWAKING	Pos	
8025	1/10/14	F	4.0	RWAKING	Pos	Pos
8026	1/10/14	F	35.0	RWAKING	Pos	
8027	1/10/14	F	10.0	KYANYA		Pos
8028	1/10/14	M	5.0	IBANDA	Pos	Pos
8029	1/10/14	F	20.0	MURAMBA	Neg	
8030	1/10/14	F	20.0	IZINGA	Neg	
8031	1/10/14	M	5.0	IZINGA	Pos	Pos
8032	1/10/14	F	25.0	BUNYANGONI	Neg	
8033	1/10/14	F	5.0	NDUGHUTU	Neg	
8034	1/10/14	M	10.0	IZINGA	Pos	
8035	1/10/14	F	13.0	BUGOYE	Neg	
8036	1/10/14	F	6.0	NDUGHUTU	Pos	Pos
8037	1/10/14	F	24.0	NDUGHUTU		Neg
8038	1/10/14	F	38.0	BUNYANGONI	Neg	
8039	1/10/14	M	7.0	NDUGHUTU	Neg	
8040	1/10/14	F	6.0	BUGOYE	Neg	

8041	1/10/14	M	15.0	MALIBA	Neg	
8042	1/10/14	F	21.0	BUGOYE	Neg	
8043	1/13/14	F	15.0	BUGOYE	Neg	
8044	1/13/14	F	9.0	BUGOYE	Pos	
8045	1/13/14	F	12.0	BUGOYE	Neg	
8046	1/13/14	F	0.8	IZINGA	Neg	
8047	1/13/14	M	2.5	MURAMBA	Pos	
8048	1/13/14	M	32.0	MURAMBA	Neg	
8049	1/13/14	F	35.0	KATUMBA	Neg	
8050	1/13/14	F	12.0	KIBIRIZI	Neg	
8051	1/13/14	M	2.0	NDUGHUTU	Pos	
8052	1/13/14	F	20.0	NDUGHUTU	Neg	
8053	1/13/14	F	20.0	RWAKING	Pos	
8054	1/13/14	F	26.0	NDUGHUTU	Neg	
8055	1/13/14	M	47.0	.	Neg	
8056	1/13/14	M	27.0	NDUGHUTU	Pos	
8057	1/13/14	M	45.0	BUGOYE	Pos	
8058	1/13/14	F	1.3	IBANDA	Neg	
8059	1/13/14	F	23.0	MURAMBA	Neg	
8060	1/13/14	F	22.0	BUNYANGONI	Neg	
8061	1/13/14	F	40.0	MALIBA	Neg	
8062	1/13/14	F	62.0	KIKOKERA	Neg	
8063	1/13/14	F	32.0	MUHAMBO	Neg	
8064	1/13/14	M	1.7	RWAKING	Neg	Pos
8065	1/13/14	F	3.0	MURAMBA	Neg	
8066	1/13/14	M	7.0	RWAKING	Pos	
8067	1/13/14	M	12.0	RWAKING	Pos	
8068	1/13/14	F	20.0	MURAMBA	Pos	
8069	1/13/14	F	3.5	IZINGA	Pos	
8070	1/13/14	F	56.0	BUGOYE	Neg	
8071	1/13/14	F	18.0	BUGOYE	Neg	
8072	1/13/14	F	0.6	BUGOYE	Neg	
8073	1/13/14	M	4.0	RWAKING	Neg	
8074	1/13/14	F	57.0	KANYAN	Neg	
8075	1/13/14	F	2.5	KYARUGOMOKA	Pos	
8076	1/13/14	F	6.0	KANYAN	Neg	
8077	1/13/14	M	3.5	MALIBA	Pos	
8078	1/13/14	M	4.5	MALIBA	Pos	
8079	1/13/14	F	22.0	MALIBA	Neg	
8080	1/14/14	F	11.0	IZINGA	Pos	
8081	1/14/14	M	3.5	BUGOYE	Neg	
8082	1/14/14	M	1.4	BUGOYE	Neg	
8083	1/14/14	F	3.0	BUGOYE	Neg	

8084	1/14/14	F	0.7	KATOOKE	Neg	
8085	1/14/14	M	17.0	BUNYANGONI	Pos	
8086	1/14/14	M	15.0	IZINGA	Pos	
8087	1/14/14	M	15.0	NDUGHUTU	Pos	Neg
8088	1/14/14	F	43.0	BUZIBWERA	Neg	
8089	1/14/14	M	16.0	KANYAN	Neg	
8090	1/14/14	F	13.0	MURAMBA	Neg	
8091	1/14/14	M	7.0	MURAMBA	Pos	
8092	1/14/14	F	50.0	BUGOYE	Neg	
8093	1/14/14	F	49.0	HIMA	Neg	
8094	1/14/14	F	0.6	KANYAN	Neg	
8095	1/14/14	M	6.5	KYARUGOMOKA	Pos	
8096	1/14/14	M	49.0	MURAMBA	Pos	
8097	1/14/14	M	20.0	MALIBA	Pos	
8098	1/14/14	M	0.4	KAKINDO	Neg	
8099	1/14/14	M	2.0	MURAMBA	Neg	
8100	1/14/14	F	9.0	IZINGA	Neg	
8101	1/14/14	F	27.0	BUNYANGONI	Neg	
8102	1/14/14	F	3.0	MURAMBA	Pos	
8103	1/14/14	M	11.0	BUGOYE	Neg	
8104	1/14/14	F	9.0	KAKINDO	Pos	
8105	1/14/14	F	23.0	KAKINDO	Pos	
8106	1/14/14	F	37.0	MALIBA	Pos	
8107	1/14/14	M	9.0	BUNYANGONI	Neg	
8108	1/14/14	F	21.0	BUNYANGONI	Pos	Pos
8109	1/14/14	F	2.7	BUNYANGONI	Pos	Pos
8110	1/14/14	M	1.3	BUNYANGONI	Pos	Pos
8111	1/14/14	F	5.3	RWAKING	Pos	
8112	1/14/14	F	10.0	KISAMBA	Neg	
8113	1/14/14	M	18.0	RWAKING	Neg	
8114	1/14/14	F	14.0	IZINGA	Pos	
8115	1/14/14	M	1.9	NDUGHUTU	Neg	
8116	1/14/14	F	38.0	KATUMBA	Neg	
8117	1/14/14	F	4.0	KYANYA	Pos	
8118	1/14/14	F	0.8	KYANYA	Pos	
8119	1/14/14	F	14.0	NDUGHUTU	Pos	
8120	1/14/14	M	0.3	KISANZI	Neg	
8121	1/14/14	F	28.0	KYANYA	Pos	
8122	1/14/14	M	21.0	KISANZI	Pos	
8123	1/14/14	F	14.0	RWAKING	Neg	
8124	1/14/14	M	24.0	BUGOYE	Neg	
8125	1/14/14	F	39.0	BUGOYE	Neg	
8126	1/15/14	M	3.5	MALIBA	Neg	

8127	1/15/14	F	18.0	MALIBA	Neg	
8128	1/15/14	M	22.0	IZINGA	Pos	
8129	1/15/14	F	16.0	MURAMBA	Neg	
8130	1/15/14	F	0.7	MURAMBA	Pos	
8131	1/15/14		16.0	BUGOYE	Pos	
8132	1/15/14		.	MURAMBA	Pos	
8133	1/15/14	M	5.0	IZINGA	Pos	
8134	1/15/14	M	32.0	KANYAN	Neg	
8135	1/15/14	F	30.0	MALIBA	Pos	
8136	1/15/14	F	1.3	RWAKING	Neg	
8137	1/15/14	F	18.0	KYANYA	Neg	
8138	1/15/14	F	28.0	BUGOYE	Neg	
8139	1/15/14	M	13.0	BUGOYE	Neg	
8140	1/15/14	M	9.0	BUGOYE	Neg	
8141	1/15/14	F	13.0	BUGOYE	Pos	
8142	1/15/14	F	13.0	BUGOYE	Neg	
8143	1/15/14	F	7.0	NDUGHUTU	Neg	
8144	1/15/14		52.0	MURAMBA	Pos	
8145	1/15/14	F	1.0	NDUGHUTU	Neg	
8146	1/15/14	M	2.0	BUGOYE	Pos	
8147	1/15/14	M	7.0	MURAMBA	Pos	
8148	1/15/14	F	3.0	RWAKING	Pos	
8149	1/15/14		54.0	BUGOYE	Neg	Neg
8150	1/15/14	M	1.0	IZINGA	Pos	
8151	1/15/14	F	17.0	RWAKING	Pos	
8152	1/15/14	F	10.0	MURAMBA	Pos	
8153	1/15/14	F	36.0	KANYAN	Pos	
8154	1/15/14	F	7.0	KIKOKERA	Pos	
8155	1/15/14	M	1.8	MUHAMBO	Neg	
8156	1/15/14	F	1.7	KATOOKE	Neg	
8157	1/15/14	F	24.0	IBANDA	Neg	Neg
8158	1/15/14	F	33.0	RWAKING	Neg	
8159	1/15/14	F	7.0	RWAKING	Neg	
8160	1/15/14	F	6.5	.	Pos	
8161	1/15/14	F	10.0	MALIBA	Pos	
8162	1/15/14	F	4.0	KATOOKE	Neg	
8163	1/15/14	M	6.0	KATUMBA	Pos	
8164	1/15/14	F	28.0	BUGOYE	Neg	
8165	1/15/14	F	0.6	IZINGA	Neg	
8166	1/15/14	F	32.0	IZINGA	Neg	
8167	1/16/14	M	14.0	IZINGA	Pos	
8168	1/16/14	F	37.0	MURAMBA	Pos	
8169	1/16/14	F	38.0	KANYAN	Neg	

8170	1/16/14	F	0.7	KANYAN	Neg
8171	1/16/14	F	35.0	BUGOYE	Neg
8172	1/16/14	F	7.0	KANYAN	Pos
8173	1/16/14	F	38.0	BUGOYE	Pos
8174	1/16/14	M	25.0	BUGOYE	Pos
8175	1/16/14	F	23.0	KISENDERA	Neg
8176	1/16/14	M	12.0	MAPATI	Neg
8177	1/16/14	M	2.0	KISANZI	Pos
8178	1/16/14	F	22.0	KIRULI	Neg
8179	1/16/14	F	0.3	MURAMBA	Neg
8180	1/16/14	M	25.0	NYAKARUNGU	Neg
8181	1/16/14	F	17.0	BUVATO	Neg
8182	1/16/14	M	9.0	BUGOYE	Pos
8183	1/16/14	M	30.0	MALIBA	Pos
8184	1/16/14	M	13.0	MURAMBA	Pos
8185	1/16/14	F	1.3	MURAMBA	Pos
8186	1/16/14	M	32.0	MURAMBA	Pos
8187	1/16/14	F	2.1	MALIBA	Neg
8188	1/16/14	F	52.0	MALIBA	Neg
8189	1/16/14	F	30.0	MALIBA	Pos
8190	1/16/14	F	0.4	MURAMBA	Neg
8191	1/16/14	F	19.0	MALIBA	Neg
8192	1/16/14	F	10.0	MALIBA	Pos
8193	1/16/14	M	1.0	IZINGA	Neg
8194	1/16/14	F	38.0	KISENDERA	Neg
8195	1/16/14	F	3.0	BUNYANGONI	Pos
8196	1/16/14	M	1.0	BUZIBWERA	Pos
8197	1/16/14	F	8.0	BUZIBWERA	Pos
8198	1/16/14	M	8.0	IZINGA	Pos
8199	1/16/14	F	25.0	IZINGA	Pos
8200	1/16/14	M	1.3	KATOOKE	Neg
8201	1/16/14	M	1.8	IHANI	Pos
8202	1/16/14	M	40.0	KISANGA	Neg
8203	1/16/14	F	60.0	RWAKING	Pos
8204	1/16/14	M	3.0	RWAKING	Neg
8205	1/16/14	M	32.0	NYAKARUNGU	Neg
8206	1/16/14	F	19.0	MURAMBA	Pos
8207	1/16/14	M	1.2	IHANI	Neg
8208	1/16/14	F	18.0	KISANGA	Neg
8209	1/16/14	F	3.5	MURAMBA	Pos
8210	1/16/14	M	0.5	NDUGHUTU	Neg
8211	1/17/14	F	1.0	RWAKING	Neg
8212	1/17/14	F	5.0	MALIBA	Neg

8213	1/17/14	F	12.0	MALIBA	Neg	
8214	1/17/14	M	12.0	BUGOYE	Neg	
8215	1/17/14	M	10.0	BUGOYE	Pos	
8216	1/17/14	F	13.0	BUGOYE	Pos	
8217	1/17/14	M	10.0	BUGOYE	Pos	
8218	1/17/14	M	49.0	BUGOYE	Neg	
8219	1/17/14	M	14.0	RWAKING	Pos	
8220	1/17/14	M	27.0	MURAMBA	Pos	
8221	1/17/14	M	6.0	KAKINDO	Pos	
8222	1/17/14	F	15.0	KAKINDO	Neg	
8223	1/17/14	F	1.0	MURAMBA	Neg	
8224	1/17/14	F	26.0	KANYAN	Neg	
8225	1/17/14	M	2.7	KANYAN	Neg	Pos
8226	1/17/14	F	2.0	MALIBA	Pos	
8227	1/17/14	F	20.0	KAKINDO	Neg	
8228	1/17/14	F	17.0	IBANDA	Pos	
8229	1/17/14	F	12.0	NDUGHUTU	Neg	
8230	1/17/14	F	1.7	NDUGHUTU	Neg	
8231	1/17/14	F	34.0	KANYAN	Pos	
8232	1/17/14	F	38.0	KIBIRIZI	Neg	
8233	1/17/14	M	2.1	IZINGA	Pos	
8234	1/17/14	F	2.0	IZINGA	Pos	
8235	1/17/14	F	20.0	RWAKING	Pos	
8236	1/17/14	F	20.0	MURAMBA	Pos	
8237	1/17/14	F	32.0	KYARUGOMOKA	Neg	
8238	1/17/14	F	0.5	RWAKING	Neg	
8239	1/17/14	F	49.0	BUZIBWERA	Neg	
8240	1/17/14	M	10.0	IZINGA	Neg	
8241	1/17/14	M	1.1	BUGOYE	Neg	
8242	1/17/14	M	45.0	KATOOKE		Neg
8243	1/17/14	F	23.0	.	Neg	
8244	1/17/14	M	23.0	BUGOYE	Neg	
8245	1/20/14	M	36.0	MUHAMBO	Pos	
8246	1/20/14	M	3.0	KAKINDO	Pos	
8247	1/20/14	F	1.1	KAKINDO	Pos	
8248	1/20/14	F	58.0	BUGOYE	Pos	
8249	1/20/14	M	15.0	IBANDA	Neg	
8250	1/20/14	M	2.0	NDUGHUTU	Neg	Neg
8251	1/20/14	M	2.0	MALIBA	Pos	
8252	1/20/14	M	8.0	IBANDA	Pos	
8253	1/20/14	M	16.0	MALIBA	Pos	
8254	1/20/14	M	28.0	BUGOYE	Neg	
8255	1/20/14	F	12.0	MURAMBA	Pos	

8256	1/20/14	F	28.0	MURAMBA	Neg
8257	1/20/14	F	32.0	MURAMBA	Pos
8258	1/20/14	F	28.0	BUGOYE	Neg
8259	1/20/14	F	48.0	NYDOME	Neg
8260	1/20/14	M	5.0	KIKOKERA	Neg
8261	1/20/14	F	12.0	BUNYANGONI	Pos
8262	1/20/14	F	45.0	IZINGA	Pos
8263	1/20/14	F	38.0	KANYAN	Pos
8264	1/20/14	F	18.0	KYARUGOMOKA	Neg
8265	1/20/14	M	42.0	BIKONE	Pos
8266	1/20/14	M	0.8	KISANGA	Neg
8267	1/20/14	F	52.0	NYABISUSI	Neg
8268	1/20/14	M	51.0	MURAMBA	Neg
8269	1/20/14	F	16.0	NYABUSISI	Pos
8270	1/20/14	M	6.0	KYARUGOMOKA	Neg
8271	1/20/14	M	41.0	MURAMBA	Pos
8272	1/20/14	F	35.0	KIRULI	Neg
8273	1/20/14	F	15.0	KIRULI	Pos
8274	1/20/14	F	58.0	BUNYANGONI	Pos
8275	1/20/14	F	57.0	MALIBA	Neg
8276	1/20/14	F	28.0	KISAMBA	Neg
8277	1/20/14	F	31.0	NDUGHUTU	Neg
8278	1/20/14	F	37.0	NDUGHUTU	Neg
8279	1/20/14	F	4.0	BUGOYE	Pos
8280	1/20/14	F	19.0	KANYAN	Pos
8281	1/20/14	F	29.0	IBANDA	Neg
8282	1/20/14	M	15.0	BUGOYE	Pos
8283	1/20/14	M	70.0	BUGOYE	Pos
8284	1/20/14	F	28.0	MURAMBA	Neg
8285	1/20/14	M	7.0	BUGOYE	Pos
8286	1/20/14	F	15.0	NDUGHUTU	Neg
8287	1/20/14	F	9.0	IZINGA	Pos
8288	1/20/14	F	49.0	IBANDA	Neg
8289	1/20/14	F	35.0	KANYAN	Neg
8290	1/20/14	F	9.0	NDUGHUTU	Neg
8291	1/20/14	F	0.8	BUGOYE	Neg
8292	1/20/14	M	11.0	IBANDA	Pos
8293	1/20/14	M	50.0	KIBIRIZI	Pos
8294	1/20/14	F	25.0	NDUGHUTU	Neg
8295	1/20/14	M	4.0	MALIBA	Pos
8296	1/20/14	M	4.0	IBANDA	Neg
8297	1/20/14	F	24.0	IBANDA	Neg
8298	1/20/14	F	25.0	KATOOKE	Pos

8299	1/20/14	M	0.1	NDUGHUTU	Neg	
8300	1/20/14	F	18.0	IZINGA	Pos	
8301	1/20/14	M	1.7	SEELUTI	Neg	Neg
8302	1/21/14	F	27.0	MALIBA	Pos	
8303	1/21/14	F	3.0	MALIBA	Pos	
8304	1/21/14	M	23.0	KIBIRIZI	Pos	
8305	1/21/14	F	40.0	BUGOYE	Neg	
8306	1/21/14	F	20.0	BUNYANGONI	Pos	
8307	1/21/14	F	60.0	KANYAN	Pos	
8308	1/21/14	F	4.0	KANYAN	Pos	
8309	1/21/14	M	3.0	BUGOYE	Neg	
8310	1/21/14	F	39.0	BUGOYE	Pos	
8311	1/21/14	F	0.3	MALIBA	Neg	
8312	1/21/14	F	40.0	NDUGHUTU	Neg	
8313	1/21/14	F	30.0	MURAMBA	Neg	
8314	1/21/14	F	20.0	MURAMBA	Neg	
8315	1/21/14	M	0.2	MURAMBA	Neg	
8316	1/21/14	M	7.0	BUGOYE	Neg	
8317	1/21/14	M	5.0	KANYAN	Neg	
8318	1/21/14	M	10.0	MURAMBA	Pos	
8319	1/21/14	F	9.0	BUGOYE	Pos	
8320	1/21/14	M	13.0	BUGOYE	Pos	
8321	1/21/14	F	17.0	BUGOYE	Neg	
8322	1/21/14	F	21.0	MURAMBA	Pos	
8323	1/21/14	F	9.0	KATOOKE	Neg	
8324	1/21/14	M	40.0	MURAMBA	Pos	
8325	1/21/14	M	23.0	BUGOYE	Neg	
8326	1/21/14	F	38.0	KYANYA	Neg	
8327	1/21/14	F	8.0	BUGOYE	Pos	
8328	1/21/14	F	7.0	BUGOYE	Pos	
8329	1/21/14	M	6.0	BUGOYE	Pos	
8330	1/21/14	M	6.0	BUGOYE	Pos	
8331	1/21/14	M	1.1	IBANDA	Neg	
8332	1/21/14	F	1.4	MAGHOMA	Neg	
8333	1/21/14	F	2.0	KANYAN	Neg	
8334	1/21/14	F	6.0	BUGOYE	Neg	
8335	1/21/14	F	15.0	MALIBA	Neg	
8336	1/21/14	M	6.0	BUGOYE	Pos	
8337	1/21/14	F	36.0	KAKINDO	Neg	
8338	1/21/14	F	24.0	KIBIRIZI	Pos	
8339	1/21/14	F	20.0	BUGOYE	Pos	
8340	1/21/14	F	32.0	MALIBA	Neg	
8341	1/21/14	F	22.0	BUGOYE	Neg	

8342	1/21/14	F	24.0	BUGOYE	Neg	
8343	1/21/14	F	50.0	MURAMBA	Neg	
8344	1/21/14	F	16.0	MURAMBA	Pos	
8345	1/21/14	M	2.0	BUNYANGONI	Pos	
8346	1/21/14	M	49.0	MURAMBA	Neg	
8347	1/21/14	F	0.8	MURAMBA	Neg	
8348	1/21/14	F	55.0	NYABISUSI	Neg	
8349	1/21/14	F	56.0	BUZIBWERA	Pos	
8350	1/21/14	F	0.3	BUGOYE	Neg	
8351	1/21/14	M	46.0	BUGOYE	Neg	
8352	1/21/14	F	39.0	MURAMBA	Pos	
8353	1/22/14	F	19.0	MALIBA	Neg	
8354	1/22/14	M	1.3	MALIBA	Neg	
8355	1/22/14	F	35.0	KATOOKE	Pos	
8356	1/22/14	F	16.0	MALIBA	Neg	
8357	1/22/14	F	9.0	BUGOYE	Pos	
8358	1/22/14	M	36.0	BUGOYE	Pos	
8359	1/22/14	M	40.0	BUGOYE	Pos	
8360	1/22/14	M	3.0	MALIBA	Pos	
8361	1/22/14	F	40.0	MALIBA	Neg	
8362	1/22/14	F	80.0	BUGOYE	Neg	
8363	1/22/14	M	6.0	MALIBA	Pos	
8364	1/22/14	F	29.0	BUGOYE	Neg	
8365	1/22/14	F	17.0	KANYAN	Pos	
8366	1/22/14	F	6.0	MURAMBA	Pos	
8367	1/22/14	F	1.0	BUGOYE	Neg	
8368	1/22/14	F	21.0	KISAMBA	Neg	
8369	1/22/14	F	30.0	MURAMBA	Neg	
8370	1/22/14	M	24.0	MALIBA	Pos	
8371	1/22/14	M	21.0	MALIBA	Neg	
8372	1/22/14	M	0.5	MALIBA	Neg	
8373	1/22/14	M	31.0	MURAMBA	Neg	
8374	1/22/14	F	1.0	KATOOKE	Pos	
8375	1/22/14	F	2.0	KIBIRIZI	Neg	Neg
8376	1/22/14	F	8.0	KAKINDO	Pos	
8377	1/22/14	F	45.0	MALIBA	Neg	
8378	1/22/14	F	30.0	NDUGHUTU	Neg	
8379	1/22/14	M	45.0	MURAMBA	Pos	
8380	1/22/14	F	4.0	BUGOYE	Neg	
8381	1/22/14	M	32.0	IBANDA	Neg	
8382	1/22/14	F	21.0	KISENDERA	Neg	
8383	1/22/14	F	0.5	BUGOYE	Neg	
8384	1/22/14	F	23.0	BUGOYE	Neg	

8385	1/22/14	F	22.0	KIBIRIZI	Neg	
8386	1/22/14	F	13.0	IHANI	Neg	
8387	1/22/14	F	8.0	KIBIRIZI	Neg	
8388	1/22/14	M	75.0	KATOOKE	Neg	
8389	1/22/14	F	0.8	MURAMBA	Neg	
8390	1/22/14	F	20.0	MURAMBA	Neg	
8391	1/22/14	F	25.0	KYANYA	Neg	
8392	1/22/14	M	2.0	BUGOYE	Neg	Neg
8393	1/22/14	F	29.0	BUGOYE	Neg	
8394	1/22/14	M	3.2	BUGOYE	Neg	
8395	1/22/14	M	15.0	BUGOYE	Neg	
8396	1/22/14	M	11.0	BUGOYE	Neg	
8397	1/22/14	F	13.0	BUGOYE	Neg	
8398	1/22/14	F	1.5	BUGOYE	Pos	
8399	1/22/14	F	18.0	KIBIRIZI	Pos	
8400	1/22/14	F	35.0	MURAMBA	Pos	
8401	1/22/14	F	3.0	MURAMBA	Pos	
8402	1/22/14	F	50.0	KIHINDI	Neg	
8403	1/22/14	F	34.0	BUNYANGONI	Neg	
8404	1/22/14	M	9.0	KATOOKE	Pos	
8405	1/22/14	F	26.0	MUHAMBO	Pos	
8406	1/22/14	M	0.4	MUHAMBO	Neg	
8407	1/23/14	F	40.0	MALIBA	Neg	
8408	1/23/14	M	28.0	MALIBA	Pos	
8409	1/23/14	F	13.0	MUKATI	Pos	
8410	1/23/14	M	19.0	BUNYANGONI	Pos	
8411	1/23/14	F	1.2	MALIBA	Pos	
8412	1/23/14	M	16.0	BUGOYE	Neg	
8413	1/23/14	M	13.0	IZINGA	Neg	
8414	1/23/14	M	30.0	IZINGA	Neg	
8415	1/23/14	M	1.5	BUNYANGONI	Neg	
8416	1/23/14	F	40.0	MALIBA	Neg	
8417	1/23/14	F	8.0	IZINGA	Pos	
8418	1/23/14	M	9.0	IZINGA	Pos	
8419	1/23/14	F	28.0	BIKONE	Neg	
8420	1/23/14	M	27.0	MALIBA	Pos	
8421	1/23/14	F	52.0	MALIBA	Neg	
8422	1/23/14	F	22.0	KIBOTA	Neg	
8423	1/23/14	M	22.0	IZINGA	Pos	
8424	1/23/14	F	14.0	BIKONE	Pos	
8425	1/23/14	F	9.0	BIKONE	Pos	
8426	1/23/14	F	53.0	IZINGA	Neg	
8427	1/23/14	F	8.0	MALIBA	Pos	

8428	1/23/14	M	3.0	IZINGA	Pos
8429	1/23/14	M	2.0	MALIBA	Neg
8430	1/23/14	M	42.0	BUGOYE	Pos
8431	1/23/14	M	10.0	BUGOYE	Pos
8432	1/23/14	M	18.0	BUGOYE	Pos
8433	1/23/14	F	23.0	BUHUNGA	Neg
8434	1/23/14	F	20.0	KISENDERA	Pos
8435	1/23/14	M	4.5	MALIBA	Neg
8436	1/23/14	F	25.0	NDUGHUTU	Pos
8437	1/23/14	F	25.0	KANYAN	Neg
8438	1/23/14	F	1.2	KANYAN	Neg
8439	1/23/14	F	33.0	IBANDA	Pos
8440	1/23/14	F	13.0	IBANDA	Pos
8441	1/23/14	M	1.3	IBANDA	Neg
8442	1/23/14	M	26.0	MALIBA	Pos
8443	1/23/14	M	0.3	BUNYANGONI	Neg
8444	1/24/14	M	40.0	IBANDA	Neg
8445	1/24/14	F	82.0	BUGOYE	Neg
8446	1/24/14	M	0.4	BUGOYE	Neg
8447	1/24/14	F	0.4	BUGOYE	Neg
8448	1/24/14	F	8.0	BUGOYE	Neg
8449	1/24/14	F	20.0	MUHAMBO	Pos
8450	1/24/14	M	28.0	MALIBA	Neg
8451	1/24/14	F	18.0	MALIBA	Neg
8452	1/24/14	F	0.4	BUNYANGONI	Neg
8453	1/24/14	M	23.0	MALIBA	Pos
8454	1/24/14	F	19.0	IBANDA	Pos
8455	1/24/14	M	60.0	BUGOYE	Neg
8456	1/24/14	F	24.0	MURAMBA	Neg
8457	1/24/14	F	0.8	MURAMBA	Neg
8458	1/24/14	M	45.0	MUHAMBO	Neg
8459	1/24/14	F	15.0	MUHAMBO	Neg
8460	1/24/14	F	28.0	MALIBA	Neg
8461	1/24/14	M	4.0	KYARUGOMOKA	Pos
8462	1/24/14	M	16.0	MURAMBA	Pos
8463	1/24/14	M	11.0	KAKINDO	Pos
8464	1/24/14	F	64.0	KAKINDO	Neg
8465	1/24/14	F	25.0	MURAMBA	Neg
8466	1/24/14	F	9.0	MURAMBA	Pos
8467	1/24/14	F	9.0	MALIBA	Pos
8468	1/24/14	M	12.0	KAKINDO	Neg
8469	1/24/14	F	25.0	MALIBA	Neg
8470	1/24/14	F	18.0	MURAMBA	Pos

8471	1/24/14	F	28.0	BWERA	Neg	
8472	1/24/14	F	0.3	BUGOYE	Neg	
8473	1/24/14	F	19.0	BUGOYE	Neg	
8474	1/24/14	F	15.0	BUGOYE	Neg	
8475	1/24/14	F	17.0	MALIBA	Neg	
8476	1/24/14	M	11.0	MALIBA	Pos	
8477	1/24/14	M	38.0	KANYAN	Neg	
8478	1/24/14	M	3.0	IZINGA	Neg	
8479	1/24/14	M	56.0	BUGOYE	Pos	
8480	1/24/14	F	42.0	KANYAN	Neg	
8481	1/24/14	F	25.0	MALIBA	Neg	
8482	1/24/14	M	4.5	IZINGA	Pos	
8483	1/24/14	F	48.0	IZINGA	Neg	
8484	1/24/14	F	21.0	MURAMBA	Neg	
8485	1/24/14	F	22.0	KYARUGOMOKA	Pos	
8486	1/24/14	F	20.0	KIBOTA	Neg	
8487	1/24/14	M	4.0	MURAMBA	Pos	
8488	1/24/14	F	4.0	MURAMBA	Neg	
8489	1/24/14	F	4.0	KIKOKERA	Pos	
8490	1/24/14	F	0.6	BUHUNGA	Neg	
8491	1/24/14	F	8.0	IHANI	Pos	
8492	1/24/14	F	70.0	KANYAN	Pos	
8493	1/24/14	F	4.0	BUGOYE	Neg	Neg
8494	1/24/14	M	12.0	KANYAN	Pos	
8495	1/24/14	F	35.0	IHANI	Pos	
8496	1/24/14	M	7.0	BUNYANGONI	Pos	Pos
8497	1/24/14	F	8.0	KANYAN	Pos	Pos
8498	1/24/14	F	22.0	BIKONE	Neg	
8499	1/24/14	F	2.0	KANYAN	Neg	
8500	1/24/14	F	40.0	KYANYA	Neg	
8501	1/24/14	M	2.0	IZINGA	Pos	
8502	1/24/14	F	8.0	IZINGA	Pos	
8503	1/24/14	M	6.0	KATUMBA	Neg	
8504	1/24/14	M	21.0	IZINGA	Neg	Neg
8505	1/24/14	F	10.0	KANYAN	Neg	
8506	1/24/14	M	49.0	BUGOYE	Pos	
8507	1/24/14	M	26.0	MALIBA	Neg	
8508	1/24/14	F	24.0	IBANDA	Neg	
8509	1/24/14	F	24.0	MALIBA	Pos	
8510	1/24/14	F	22.0	IBANDA	Neg	
8511	1/24/14	F	0.3	MALIBA	Neg	
8512	1/24/14	F	4.0	KANYAN	Neg	
8513	1/24/14	F	50.0	BUGOYE	Neg	

8514	1/24/14	M	27.0	NYAKARUNGU	Pos	
8515	1/24/14	M	0.7	KIRONGO	Neg	Neg
8516	1/28/14	M	80.0	MAGHOMA	Pos	
8517	1/28/14	F	1.3	BUNYANGONI	Neg	
8518	1/28/14	F	7.0	MURAMBA	Neg	
8519	1/28/14	M	1.0	IZINGA	Neg	
8520	1/28/14	M	25.0	NDUGHUTU	Neg	
8521	1/28/14	F	13.0	BUGOYE	Pos	
8522	1/28/14	F	1.3	MURAMBA	Pos	
8523	1/28/14	F	1.0	BUGOYE	Neg	Neg
8524	1/28/14	F	32.0	IHANI	Pos	
8525	1/28/14	F	32.0	BUGOYE	Neg	
8526	1/28/14	F	0.1	NYABUSISI	Neg	
8527	1/28/14	F	11.0	KANYAN	Pos	
8528	1/28/14	F	42.0	IZINGA	Pos	
8529	1/28/14	F	19.0	KIRONGO	Pos	Pos
8530	1/28/14	M	2.0	KANYAN	Neg	
8531	1/28/14	F	6.0	NDUGHUTU	Pos	
8532	1/28/14	M	38.0	KATOOKE	Neg	
8533	1/28/14	M	7.0	KATOOKE	Pos	
8534	1/28/14	F	50.0	KASESE	Neg	
8535	1/28/14	F	40.0	NYABISUSI	Neg	
8536	1/28/14	F	6.0	BUNYANGONI	Pos	
8537	1/28/14	M	1.5	MUHAMBO	Neg	
8538	1/28/14	F	4.5	NDUGHUTU	Neg	
8539	1/28/14	F	18.0	KATOOKE	Pos	
8540	1/29/14	M	32.0	IBANDA	Neg	Neg
8541	1/29/14	M	6.0	KISANGA	Pos	
8542	1/29/14	F	8.5	MALIBA	Pos	
8543	1/29/14	F	40.0	MUHAMBO	Pos	
8544	1/29/14	F	6.0	KISANGA	Pos	
8545	1/29/14	F	14.0	RWAKING	Neg	
8546	1/29/14	F	69.0	MALIBA	Neg	
8547	1/29/14	F	8.0	KAKINDO	Pos	
8548	1/29/14	M	10.0	IZINGA	Pos	
8549	1/29/14	F	19.0	KANYAN	Neg	
8550	1/29/14	F	6.0	KANYAN	Neg	
8551	1/29/14	F	15.0	NDUGHUTU	Neg	
8552	1/29/14	F	34.0	MURAMBA	Neg	
8553	1/29/14	F	7.0	KANYAN	Neg	
8554	1/29/14	F	10.0	KANYAN	Pos	Pos
8555	1/29/14	F	28.0	KANYAN	Pos	Pos
8556	1/29/14	M	4.5	BUZIBWERA	Pos	

8557	1/29/14	F	1.0	BUGOYE	Neg	
8558	1/29/14	F	17.0	NDUGHUTU	Pos	
8559	1/29/14	M	7.0	MALIBA	Pos	Pos
8560	1/29/14	F	17.0	KANYAN	Neg	
8561	1/29/14	F	40.0	KATOOKE	Neg	
8562	1/29/14	M	5.5	MURAMBA	Pos	Pos
8563	1/29/14	F	19.0	MAGHOMA	Neg	
8564	1/29/14	M	1.3	NYAKARUNGU	Neg	
8565	1/29/14	M	20.0	KYARUGOMOKA	Neg	
8566	1/29/14	F	12.0	NDUGHUTU	Pos	Pos
8567	1/29/14	F	57.0	KANYAN	Neg	
8568	1/29/14	M	17.0	KIRONGO	Pos	Pos
8569	1/29/14	F	28.0	MURAMBA	Neg	
8570	1/29/14	F	13.0	IZINGA	Pos	Pos
8571	1/29/14	F	24.0	MURAMBA	Pos	
8572	1/29/14	F	13.0	IZINGA	Pos	
8573	1/29/14	M	62.0	MURAMBA	Neg	
8574	1/29/14	F	60.0	MALIBA	Pos	Pos
8575	1/29/14	F	55.0	IZINGA	Pos	
8576	1/29/14	F	25.0	KANYAN	Neg	
8577	1/29/14	F	9.0	RWAKING	Pos	Pos
8578	1/29/14	F	17.0	NDUGHUTU	Neg	
8579	1/30/14	F	1.0	RWAKING	Neg	
8580	1/30/14	F	1.3	BUGOYE	Neg	
8581	1/30/14	F	0.8	MUHAMBO	Neg	
8582	1/30/14	F	36.0	MURAMBA	Neg	
8583	1/30/14	F	1.8	MURAMBA	Neg	
8584	1/30/14	M	38.0	IBANDA	Neg	Neg
8585	1/30/14	M	0.8	RWAKING	Pos	Pos
8586	1/30/14	M	31.0	KANYAN	Neg	
8587	1/30/14	F	22.0	MALIBA	Pos	Pos
8588	1/30/14	M	50.0	MALIBA	Pos	Pos
8589	1/30/14	M	11.0	BUZIBWERA	Pos	Pos
8590	1/30/14	F	31.0	BUZIBWERA	Neg	
8591	1/30/14	M	9.0	BUZIBWERA	Pos	Pos
8592	1/30/14	M	23.0	IBANDA	Pos	Pos
8593	1/30/14	F	37.0	KISANZI	Pos	
8594	1/30/14	F	59.0	KATOOKE	Neg	
8595	1/30/14	F	8.0	MURAMBA	Pos	
8596	1/30/14	M	29.0	MURAMBA	Pos	Pos
8597	1/30/14	F	0.8	NYAKARUNGU	Neg	
8598	1/30/14	M	26.0	NDUGHUTU	Neg	
8599	1/30/14	F	48.0	BUGOYE	Neg	

8600	1/30/14	M	10.0	BUNYANGONI	Neg	
8601	1/30/14	M	3.0	KISANZI	Pos	
8602	1/30/14	M	1.3	RWAKING	Neg	
8603	1/30/14	M	3.0	IZINGA	Pos	
8604	1/30/14	F	0.5	KATUMBA	Neg	
8605	1/30/14	M	5.0	NDUGHUTU	Neg	
8606	1/30/14	M	21.0	MURAMBA	Neg	
8607	1/30/14	M	0.5	BUNYANGONI	Pos	
8608	1/31/14	M	60.0	KAKINDO	Pos	Pos
8609	1/31/14	M	72.0	IZINGA	Neg	
8610	1/31/14	M	14.0	KANYAN	Pos	
8611	1/31/14	F	39.0	KAKINDO	Pos	Pos
8612	1/31/14	M	2.0	KYARUGOMOKA	Pos	Pos
8613	1/31/14	F	28.0	IZINGA	Neg	
8614	1/31/14	F	37.0	BUGOYE	Neg	
8615	1/31/14	F	7.0	BUGOYE	Neg	
8616	1/31/14	M	9.0	NDUGHUTU	Neg	
8617	1/31/14	M	12.0	MURAMBA	Pos	
8618	1/31/14	F	26.0	RWAKING	Pos	Pos
8619	1/31/14	F	51.0	BUGOYE	Neg	
8620	1/31/14	M	2.3	KYARUGOMOKA	Pos	
8621	1/31/14	F	13.0	MURAMBA	Neg	
8622	1/31/14	M	11.0	MURAMBA	Pos	Pos
8623	1/31/14	F	9.0	MURAMBA	Pos	Pos
8624	1/31/14	F	38.0	BUNYANGONI	Pos	
8625	1/31/14	M	4.0	MBUMURO	Pos	Pos
8626	1/31/14	F	20.0	BUNYANGONI	Pos	
8627	1/31/14	F	23.0	IZINGA	Pos	
8628	1/31/14	F	2.8	IZINGA	Pos	Pos
8629	1/31/14	M	8.0	IZINGA	Pos	
8630	1/31/14	F	23.0	NYAKARUNGU	Neg	
8631	1/31/14	M	9.0	KANYAN	Pos	
8632	2/3/14	M	24.0	NYAKALENGIJO	Neg	
8633	2/3/14	F	0.5	MALIBA	Neg	
8634	2/3/14	F	32.0	MALIBA	Neg	
8635	2/3/14	F	24.0	KYARUGOMOKA	Pos	
8636	2/3/14	F	7.0	KYARUGOMOKA	Pos	Pos
8637	2/3/14	M	4.0	KYARUGOMOKA	Pos	Pos
8638	2/3/14	F	14.0	MALIBA	Neg	
8639	2/3/14	F	22.0	KIRONGO	Pos	Pos
8640	2/3/14	F	10.0	MURAMBA	Pos	Pos
8641	2/3/14	F	0.5	MALIBA	Pos	Pos
8642	2/3/14	M	10.0	BUGOYE	Pos	

8643	2/3/14	F	4.5	KISAMBA	Neg	
8644	2/3/14	F	42.0	BUGOYE	Neg	
8645	2/3/14	M	18.0	RWAKING	Pos	
8646	2/3/14	M	2.0	KYANYA	Pos	Pos
8647	2/3/14	F	24.0	KYANYA	Pos	
8648	2/3/14	F	7.0	KYANYA	Pos	Pos
8649	2/3/14	F	19.0	IZINGA	Pos	
8650	2/3/14	F	2.5	MALIBA	Pos	
8651	2/3/14	F	23.0	IZINGA	Neg	
8652	2/3/14	M	33.0	BUGOYE	Neg	
8653	2/3/14	M	13.0	KAKINDO	Pos	
8654	2/3/14	F	2.3	MALIBA	Pos	
8655	2/3/14	F	41.0	BUGOYE	Neg	Neg
8656	2/3/14	F	3.0	MALIBA	Pos	
8657	2/3/14	M	1.5	MALIBA	Neg	
8658	2/3/14	F	22.0	MALIBA	Neg	
8659	2/3/14	F	8.0	KANYAN	Pos	
8660	2/3/14	F	0.8	KIRULI	Neg	
8661	2/3/14	M	9.0	NDUGHUTU	Pos	
8662	2/3/14	F	27.0	MALIBA	Neg	
8663	2/3/14	F	34.0	IZINGA	Neg	
8664	2/3/14	F	8.0	IZINGA	Pos	Pos
8665	2/3/14	M	9.0	IZINGA	Pos	Pos
8666	2/3/14	M	4.0	KAKINDO	Pos	Pos
8667	2/3/14	F	30.0	KAKINDO	Neg	
8668	2/3/14	F	43.0	MALIBA	Neg	
8669	2/3/14	M	29.0	MURAMBA	Neg	
8670	2/3/14	M	16.0	MAGHOMA	Pos	
8671	2/3/14	M	3.0	BUNYANGONI	Pos	
8672	2/3/14	M	7.0	RWAKING	Neg	
8673	2/3/14	F	9.0	RWAKING	Pos	
8674	2/3/14	F	13.0	RWAKING	Pos	
8675	2/3/14	F	56.0	MURAMBA	Pos	
8676	2/3/14	M	0.5	KISANZI	Pos	
8677	2/3/14	F	35.0	IZINGA	Neg	
8678	2/3/14	F	0.5	NDUGHUTU	Neg	
8679	2/3/14	M	2.5	KANYAN	Pos	
8680	2/4/14	M	41.0	BUGOYE	Pos	
8681	2/4/14	M	45.0	BUGOYE	Pos	
8682	2/4/14	F	26.0	BUGOYE	Neg	
8683	2/4/14	M	1.0	BUGOYE	Neg	
8684	2/4/14	F	7.0	BUNYANGONI	Pos	Pos
8685	2/4/14	F	50.0	KABUKERA	Pos	

8686	2/4/14	M	17.0	MURAMBA	Pos	Pos
8687	2/4/14	M	45.0	BUGOYE	Neg	
8688	2/4/14	M	4.0	BUGOYE	Pos	Pos
8689	2/4/14	M	6.0	BUGOYE	Pos	
8690	2/4/14	F	9.0	BUGOYE	Pos	
8691	2/4/14	M	81.0	RWAKING	Neg	
8692	2/4/14	M	4.0	RWAKING	Pos	
8693	2/4/14	F	8.0	KYARUGOMOKA	Pos	
8694	2/4/14	F	3.5	MURAMBA	Pos	Pos
8695	2/4/14	M	3.0	MURAMBA	Pos	Pos
8696	2/4/14	M	12.0	BUGOYE	Pos	Pos
8697	2/4/14	F	1.0	BUGOYE	Neg	
8698	2/4/14	F	9.0	BUGOYE	Pos	
8699	2/4/14	M	6.0	MURAMBA	Pos	Pos
8700	2/4/14	M	1.5	RWAKING	Neg	
8701	2/4/14	M	4.0	RWAKING	Pos	
8702	2/4/14	F	4.0	MURAMBA	Pos	Pos
8703	2/4/14	F	5.0	MUHAMBO	Pos	
8704	2/4/14	F	12.0	NDUGHUTU	Pos	
8705	2/4/14	F	27.0	KATOOKE	Pos	Pos
8706	2/4/14	F	13.0	NDUGHUTU	Pos	
8707	2/4/14	F	25.0	NDUGHUTU	Pos	Pos
8708	2/4/14	F	2.0	NDUGHUTU	Pos	Pos
8709	2/4/14	F	1.5	IZINGA	Neg	
8710	2/4/14	M	5.0	BUGOYE	Pos	
8711	2/4/14	F	14.0	KANYAN	Pos	
8712	2/4/14	F	4.0	MALIBA	Neg	
8713	2/4/14	M	1.0	KISOJO	Pos	Pos
8714	2/4/14	F	21.0	BUGOYE	Neg	
8715	2/5/14	F	1.0	BUGOYE	Pos	Pos
8716	2/5/14	F	60.0	MURAMBA	Neg	
8717	2/5/14	M	1.0	MURAMBA	Pos	Pos
8718	2/5/14	F	85.0	KATOOKE	Neg	
8719	2/5/14	M	5.0	RWAKING	Neg	
8720	2/5/14	F	7.0	MALIBA	Pos	
8721	2/5/14	F	42.0	KYANYA	Pos	
8722	2/5/14	M	3.0	MURAMBA	Pos	Pos
8723	2/5/14	F	37.0	NDUGHUTU	Neg	
8724	2/5/14	F	23.0	IBANDA	Neg	
8725	2/5/14	F	70.0	NDUGHUTU	Neg	
8726	2/5/14	M	18.0	MUHAMBO	Pos	
8727	2/5/14	M	11.0	BUGOYE	Pos	
8728	2/5/14	M	4.0	NDUGHUTU	Pos	

8729	2/5/14	F	21.0	MURAMBA	Neg	
8730	2/5/14	M	0.5	BUGOYE	Neg	
8731	2/5/14	M	8.0	BUGOYE	Neg	
8732	2/5/14	F	25.0	BUGOYE	Neg	
8733	2/5/14	M	5.0	RWAKING	Pos	
8734	2/5/14	M	52.0	MALIBA	Neg	
8735	2/5/14	F	1.0	MALIBA	Pos	
8736	2/5/14	M	21.0	MURAMBA	Neg	
8737	2/5/14	M	2.5	MALIBA	Neg	
8738	2/5/14	F	17.0	MALIBA	Neg	
8739	2/5/14	M	4.0	MALIBA	Pos	
8740	2/5/14	M	6.0	MALIBA	Pos	Pos
8741	2/5/14	F	6.0	RWAKING	3	Pos
8742	2/5/14	M	10.0	MURAMBA	Pos	
8743	2/5/14	F	46.0	IBANDA	Neg	
8744	2/5/14	F	18.0	IZINGA	Pos	Pos
8745	2/5/14	F	18.0	KATUMBA	Neg	
8746	2/5/14	F	12.0	IZINGA	Pos	
8747	2/6/14	M	38.0	BUZIBWERA	Neg	Neg
8748	2/6/14	F	10.0	BUGOYE	Pos	
8749	2/6/14	F	23.0	KISANGA	Pos	
8750	2/6/14	F	6.0	MURAMBA	Pos	
8751	2/6/14	F	19.0	KANYAN	Pos	Pos
8752	2/6/14	F	3.0	RWAKING	Pos	Pos
8753	2/6/14	M	4.0	BUGOYE	Neg	
8754	2/6/14	F	40.0	KANYAN	Pos	
8755	2/6/14	F	41.0	IZINGA	Pos	Pos
8756	2/6/14	M	30.0	KANYAN	Neg	
8757	2/6/14	F	1.5	BUGOYE	Neg	
8758	2/6/14	M	0.5	NDUGHUTU	Neg	
8759	2/6/14	M	16.0	MALIBA	Pos	Pos
8760	2/6/14	F	24.0	IBANDA	Pos	Pos
8761	2/6/14	M	3.0	IZINGA	Pos	
8762	2/6/14	M	19.0	KISANGA	Neg	
8763	2/6/14	M	6.0	MURAMBA	Neg	
8764	2/6/14	F	40.0	MURAMBA	Neg	
8765	2/6/14	M	70.0	IZINGA	Neg	
8766	2/6/14	M	7.0	RWAKING	Neg	
8767	2/6/14	F	60.0	KIKOKERA	Pos	Pos
8768	2/6/14	F	16.0	NDUGHUTU	Pos	
8769	2/6/14	F	20.0	KIRULI	Neg	
8770	2/6/14	M	5.0	KANYAN	Neg	
8771	2/6/14	F	37.0	KANYAN	Neg	

8772	2/6/14	M	2.0	BUNYANGONI	Pos	
8773	2/6/14	M	13.0	MURAMBA	Pos	Pos
8774	2/6/14	M	25.0	NDUGHUTU	Neg	
8775	2/6/14	F	20.0	KYARUGOMOKA	Pos	Pos
8776	2/6/14	M	3.5	NDUGHUTU	Pos	Pos
8777	2/6/14	F	52.0	NDUGHUTU	Neg	
8778	2/6/14	F	4.0	IZINGA	Pos	Pos
8779	2/6/14	F	31.0	MURAMBA	Pos	Pos
8780	2/6/14	F	8.0	BUNYANGONI	Neg	
8781	2/6/14	F	15.0	MURAMBA	Neg	
8782	2/6/14	F	12.0	BUGOYE	Neg	
8783	2/6/14	M	7.0	RWAKING	Neg	
8784	2/6/14	M	9.0	NDUGHUTU	Neg	
8785	2/6/14	F	7.0	BUGOYE	Pos	
8786	2/6/14	F	2.0	MURAMBA	Pos	
8787	2/6/14	M	34.0	KIBIRIZI	Pos	
8788	2/7/14	M	3.5	RWAKING	Pos	
8789	2/7/14	F	31.0	RWAKING	Pos	
8790	2/7/14	F	6.0	IZINGA	Pos	Pos
8791	2/7/14	F	9.0	IZINGA	Pos	
8792	2/7/14	M	0.5	NDUGHUTU	Pos	
8793	2/7/14	M	5.0	IZINGA	Pos	
8794	2/7/14	F	28.0	MAGHOMA	Pos	
8795	2/7/14	F	23.0	BUGOYE	Neg	
8796	2/7/14	M	8.0	MURAMBA	Pos	Pos
8797	2/7/14	M	0.5	BUHWEZA	Neg	
8798	2/7/14	F	28.0	BUHWEZA	Neg	
8799	2/7/14	M	9.0	MALIBA	Neg	
8800	2/7/14	F	12.0	RWAKING	Pos	
8801	2/7/14	F	12.0	MURAMBA	Pos	
8802	2/7/14	F	7.0	MURAMBA	Pos	
8803	2/7/14	F	7.0	MURAMBA	Pos	Pos
8804	2/7/14	M	1.0	MURAMBA	Pos	Neg
8805	2/7/14	F	6.0	MURAMBA	Pos	
8806	2/7/14	M	40.0	BUHWEZA	Neg	
8807	2/7/14	M	4.0	KISOJO	Pos	Pos
8808	2/7/14	F	0.5	IZINGA	Pos	
8809	2/7/14	F	4.0	IZINGA	Pos	
8810	2/7/14	M	10.0	BUGOYE	Pos	
8811	2/7/14	F	44.0	IZINGA	Pos	
8812	2/7/14	M	1.5	MURAMBA	Pos	Neg
8813	2/7/14	M	18.0	NYAKALENGIJO	Neg	
8814	2/10/14	M	13.0	MURAMBA	Pos	Pos

8815	2/10/14	F	11.0	NDUGHUTU	Pos	
8816	2/10/14	M	18.0	NYAKALENGIJO	Neg	Neg
8817	2/10/14	F	0.5	KABUYIRI	Neg	
8818	2/10/14	F	29.0	BUGOYE	Neg	
8819	2/10/14	M	39.0	KIGANDO	Neg	
8820	2/10/14	M	3.0	KANYAN	Neg	
8821	2/10/14	F	29.0	MUHAMBO	Neg	
8822	2/10/14	F	17.0	IZINGA	Pos	
8823	2/10/14	F	50.0	KISANZI	Pos	Pos
8824	2/10/14	F	10.0	KISANZI	Pos	Pos
8825	2/10/14	F	25.0	MUHAMBO	Neg	
8826	2/10/14	F	25.0	IZINGA	Pos	
8827	2/10/14	F	27.0	BUNYANGONI	Neg	
8828	2/10/14	F	19.0	MURAMBA	Neg	
8829	2/10/14	F	3.8	KIRULI	3	Pos
8830	2/10/14	F	6.0	BUNYANGONI	Pos	Pos
8831	2/10/14	F	6.0	BUNYANGONI	Neg	
8832	2/10/14	F	47.0	MALIBA	Neg	
8833	2/10/14	M	12.0	RWAKING	Pos	Pos
8834	2/10/14	F	13.0	RWAKING	Pos	
8835	2/10/14	F	34.0	KIBOTA	Neg	
8836	2/10/14	F	36.0	BUGOYE	Neg	
8837	2/10/14	M	42.0	IZINGA	Pos	
8838	2/10/14	M	17.0	IZINGA	Neg	
8839	2/10/14	F	27.0	KANYAN	Neg	
8840	2/10/14	F	3.0	MALIBA	Neg	
8841	2/10/14	F	0.5	MALIBA	Neg	
8842	2/10/14	M	24.0	KANYAN	Pos	
8843	2/10/14	F	0.3	KATUMBA	Neg	
8844	2/10/14	F	11.0	NYAKARUNGU	Pos	Pos
8845	2/10/14	M	11.0	KATOOKE	Neg	
8846	2/10/14	F	44.0	IZINGA	Pos	
8847	2/10/14	F	23.0	KATOOKE	Neg	
8848	2/10/14	F	33.0	NYAKABUGHA	Neg	
8849	2/10/14	F	33.0	KYANYA	Neg	
8850	2/11/14	F	15.0	KASESE	Neg	
8851	2/11/14	M	17.0	BUHUNGA	Pos	Pos
8852	2/11/14	F	10.0	KANYAN	Pos	
8853	2/11/14	M	32.0	RWAKING	Neg	
8854	2/11/14	M	52.0	MURAMBA	Neg	
8855	2/11/14	F	1.5	BUGOYE	Pos	Pos
8856	2/11/14	M	21.0	MURAMBA	Pos	
8857	2/11/14	F	24.0	MURAMBA	Neg	

8858	2/11/14	F	38.0	MURAMBA	Pos	Pos
8859	2/11/14	F	11.0	KATUMBA	Pos	
8860	2/11/14	F	1.3	KANYAN	Neg	
8861	2/11/14	F	27.0	KANYAN	Neg	
8862	2/11/14	M	7.0	BUGOYE	Neg	Neg
8863	2/11/14	M	60.0	RWAKING	Neg	
8864	2/11/14	M	6.0	MURAMBA	Pos	
8865	2/11/14	F	0.5	BUNYANGONI	Neg	
8866	2/11/14	M	2.0	KISAMBA	Neg	
8867	2/11/14	F	14.0	IZINGA	Pos	
8868	2/11/14	F	48.0	RWAKING	Pos	Pos
8869	2/11/14	F	24.0	RWAKING	Pos	Pos
8870	2/11/14	F	55.0	RWAKING	Neg	
8871	2/11/14	F	44.0	KYANYA	Neg	
8872	2/11/14	F	18.0	KABALE	Neg	
8873	2/11/14	M	19.0	RWAKING	Neg	
8874	2/11/14	M	10.0	IZINGA	Pos	Pos
8875	2/11/14	F	8.0	IZINGA	Pos	Pos
8876	2/11/14	M	7.0	MURAMBA	Neg	
8877	2/11/14	F	80.0	KISAMBA	Neg	
8878	2/12/14	F	25.0	KANYAN	Neg	
8879	2/12/14	F	2.0	MURAMBA	Pos	Pos
8880	2/12/14	M	27.0	RWAKING	Pos	Pos
8881	2/12/14	M	80.0	MUHAMBO	Neg	
8882	2/12/14	F	27.0	KYARUGOMOKA	Neg	
8883	2/12/14	M	2.0	BUGOYE	Neg	
8884	2/12/14	M	6.0	MALIBA	Pos	
8885	2/12/14	M	2.0	NDUGHUTU	Pos	
8886	2/12/14	M	12.0	MURAMBA	Neg	
8887	2/12/14	F	10.0	IZINGA	Neg	
8888	2/13/14	M	38.0	MURAMBA	Neg	
8889	2/13/14	F	41.0	KIKOKERA	Pos	
8890	2/13/14	M	6.0	NDUGHUTU	Pos	Pos
8891	2/13/14	M	0.3	RWAKING	Neg	
8892	2/13/14	F	25.0	BUGOYE	Pos	
8893	2/13/14	M	10.0	KYANYA	Pos	
8894	2/13/14	F	20.0	MALIBA	Pos	
8895	2/13/14	F	29.0	BUGOYE	Pos	
8896	2/13/14	M	2.0	KIBIRIZI	Pos	
8897	2/13/14	M	7.0	IZINGA	Neg	
8898	2/13/14	F	5.0	BUZIBWERA	Pos	Pos
8899	2/13/14	F	23.0	MALIBA	Neg	
8900	2/13/14	F	2.0	BUGOYE	Neg	

8901	2/13/14	F	2.3	BUNYANGONI	Neg	
8902	2/13/14	F	26.0	KIBIRIZI	Neg	
8903	2/14/14	F	26.0	BUGOYE	Neg	
8904	2/14/14	M	32.0	IBANDA	Neg	
8905	2/14/14	F	1.8	MUBUKU	Pos	Pos
8906	2/14/14	F	5.0	MURAMBA	Pos	
8907	2/14/14	F	12.0	RWAKING	Pos	Pos
8908	2/14/14	F	20.0	BOHANYA	Pos	
8909	2/14/14	F	2.0	KISOJO	Pos	Pos
8910	2/14/14	M	13.0	BUGOYE	Neg	
8911	2/14/14	F	36.0	KANYAN	Neg	
8912	2/14/14	M	2.0	MURAMBA	Neg	Pos
8913	2/14/14	F	12.0	NDUGHUTU	Pos	Pos
8914	2/14/14	F	32.0	BUNYANGONI	Neg	
8915	2/14/14	M	9.0	MALIBA	Pos	
8916	2/14/14	F	0.5	BUNYANGONI	Neg	
8917	2/14/14	F	17.0	KIRULI	Pos	
8918	2/14/14	F	13.0	KANYAN	Neg	
8919	2/14/14	F	24.0	BUGOYE	Neg	
8920	2/14/14	F	38.0	BUGOYE	Neg	
8921	2/14/14	F	13.0	KATOOKE	Pos	
8922	2/14/14	F	0.5	NDUGHUTU	Pos	
8923	2/14/14	M	2.0	MUHAMBO	Neg	
8924	2/14/14	F	18.0	BUGOYE	Pos	Pos
8925	2/14/14	F	36.0	IBANDA	Neg	
8926	2/14/14	F	0.3	NDUGHUTU	Neg	
8927	2/14/14	F	1.3	IZINGA	Pos	Pos
8928	2/14/14	F	2.0	KANYAN	Pos	
8929	2/14/14	M	20.0	BUNYANGONI	Pos	
8930	2/14/14	M	29.0	BUGOYE	Neg	
8931	2/17/14	F	45.0	IZINGA	Neg	
8932	2/17/14	M	28.0	IBANDA	Neg	
8933	2/17/14	F	13.0	BUGOYE	Pos	Pos
8934	2/17/14	M	4.0	IBANDA	Pos	
8935	2/17/14	M	2.8	BUGOYE	Neg	
8936	2/17/14	M	4.0	BUGOYE	Neg	
8937	2/17/14	F	27.0	BUGOYE	Pos	
8938	2/17/14	F	32.0	RWAKING	Neg	
8939	2/17/14	M	10.0	BUGOYE	Pos	
8940	2/17/14	F	20.0	BUGOYE	Pos	Pos
8941	2/17/14	F	1.5	IZINGA	Pos	
8942	2/17/14	F	60.0	IZINGA	Pos	
8943	2/17/14	F	13.0	MURAMBA	Neg	

8944	2/17/14	F	23.0	MURAMBA	Neg	
8945	2/17/14	F	72.0	NYAKABUGHA	Neg	
8946	2/17/14	F	17.0	IZINGA	Pos	Pos
8947	2/17/14	M	14.0	BUGOYE	Neg	
8948	2/17/14	M	21.0	BUGOYE	Pos	Pos
8949	2/17/14	M	1.0	BUHUNGA	Neg	
8950	2/17/14	F	7.0	NDUGHUTU	3	Pos
8951	2/17/14	F	40.0	KIBIRIZI	Neg	
8952	2/17/14	M	3.0	BUGOYE	Pos	
8953	2/17/14	M	2.5	BUNYANGONI	Pos	
8954	2/17/14	F	5.5	BUNYANGONI	Neg	
8955	2/17/14	F	13.0	IZINGA	Pos	
8956	2/17/14	F	29.0	BUNYANGONI	Neg	
8957	2/17/14	F	25.0	MALIBA	Neg	
8958	2/17/14	F	12.0	BUZIBWERA	Pos	
8959	2/17/14	M	4.0	IBANDA	Neg	
8960	2/17/14	M	21.0	BUGOYE	Neg	
8961	2/17/14	F	12.0	IZINGA	Neg	
8962	2/17/14	F	3.0	KISANZI	Pos	Pos
8963	2/17/14	F	30.0	KISANZI	Pos	
8964	2/17/14	M	11.0	IZINGA	Pos	
8965	2/17/14	F	5.0	IZINGA	Neg	Neg
8966	2/17/14	F	7.0	IZINGA	Pos	
8967	2/17/14	F	7.0	IZINGA	Pos	
8968	2/17/14	F	21.0	IZINGA	Pos	
8969	2/17/14	F	3.0	IZINGA	Pos	
8970	2/17/14	F	4.0	KATEBE	Neg	
8971	2/17/14	F	5.0	KIHARARA	Neg	
8972	2/17/14	M	3.0	KIBOTA	Neg	
8973	2/17/14	M	0.3	BUNYANGONI	Neg	
8974	2/17/14	M	1.5	NDUGHUTU	Neg	
8975	2/17/14	F	43.0	KAKINDO	Neg	
8976	2/17/14	M	3.0	NDUGHUTU	Pos	
8977	2/17/14	M	37.0	IBANDA	Neg	
8978	2/17/14	F	13.0	KISOJO	Pos	
8979	2/17/14	M	9.0	BUNYANGONI	Pos	
8980	2/17/14	F	54.0	KISOJO	Neg	
8981	2/17/14	M	6.0	NDUGHUTU	Pos	
8982	2/17/14	F	1.3	KISANZI	Neg	
8983	2/17/14	F	1.3	KIBOTA	Neg	
8984	2/17/14	F	21.0	BUGOYE	Pos	
8985	2/17/14	F	20.0	MURAMBA	Neg	
8986	2/17/14	F	18.0	KIKOKERA	Pos	

8987	2/17/14	M	7.0	KANYAN	Pos	
8988	2/17/14	M	0.8	MALIBA	Neg	
8989	2/17/14	F	19.0	KIBIRIZI	Neg	
8990	2/17/14	F	25.0	IZINGA	Neg	
8991	2/17/14	F	55.0	RUBONI	Neg	
8992	2/17/14	F	34.0	IZINGA	Neg	
8993	2/17/14	F	50.0	KATHI	Neg	
8994	2/17/14	F	13.0	MURAMBA	Neg	
8995	2/18/14	M	70.0	RWAKING	Pos	
8996	2/18/14	M	14.0	RWAKING	Pos	
8997	2/18/14	M	8.0	BUGOYE	Pos	
8998	2/18/14	M	3.0	BUNYANGONI	Pos	Pos
8999	2/18/14	M	17.0	BUGOYE	Neg	
9000	2/18/14	F	12.0	MUBUKU	Pos	
9001	2/18/14	F	7.0	BUGOYE	Pos	
9002	2/18/14	F	13.0	MUBUKU	Pos	
9003	2/18/14	F	13.0	NYAKABUGHA	Pos	Pos
9004	2/18/14	M	5.0	MURAMBA	Neg	
9005	2/18/14	F	9.0	RUKOKI	Pos	Pos
9006	2/18/14	F	11.0	MUBUKU	Pos	Pos
9007	2/18/14	M	3.0	IZINGA	Pos	
9008	2/18/14	F	44.0	IZINGA	Neg	
9009	2/18/14	F	23.0	MURAMBA	Neg	
9010	2/18/14	F	42.0	BUNYANGONI	Neg	
9011	2/18/14	F	21.0	BUNYANGONI	Neg	
9012	2/18/14	M	1.5	MURAMBA	Neg	
9013	2/18/14	F	38.0	BUGOYE	Neg	
9014	2/18/14	F	50.0	IZINGA	Pos	
9015	2/18/14	F	32.0	IZINGA	Pos	Pos
9016	2/18/14	F	0.5	MUBUKU	Pos	
9017	2/18/14	F	19.0	KANYAN	Neg	
9018	2/18/14	F	4.0	MUBUKU	Pos	Pos
9019	2/18/14	F	70.0	BUNYANGONI	Neg	
9020	2/18/14	F	14.0	BUGOYE	Pos	
9021	2/18/14	F	15.0	BUGOYE	Pos	
9022	2/18/14	F	15.0	MURAMBA	Pos	
9023	2/18/14	F	50.0	KANYAN	Pos	
9024	2/18/14	F	13.0	BUNYANGONI	Neg	
9025	2/18/14	F	24.0	NDUGHUTU	Neg	
9026	2/18/14	M	4.3	BULYANDEKE	Pos	Pos
9027	2/18/14	F	18.0	IBANDA	Neg	
9028	2/18/14	F	17.0	KIKOKERA	Neg	
9029	2/18/14	M	60.0	MUBUKU	Neg	

9030	2/18/14	M	12.0	IZINGA	Pos
9031	2/18/14	M	4.0	MURAMBA	Pos
9032	2/18/14	M	0.8	NYAMBUKU	Neg
9033	2/19/14	M	3.0	IZINGA	Pos
9034	2/19/14	F	35.0	IBANDA	Neg
9035	2/19/14	M	28.0	BUGOYE	Pos
9036	2/19/14	F	25.0	KIBIRIZI	Neg
9037	2/19/14	F	31.0	BIKONE	Neg
9038	2/20/14	M	7.5	MURAMBA	Neg
9039	2/20/14	M	14.0	MURAMBA	Neg
9040	2/20/14	F	5.0	MURAMBA	Neg
9041	2/20/14	M	7.0	BUGOYE	Pos
9042	2/20/14	F	13.0	MURAMBA	Pos
9043	2/20/14	F	14.0	BUGOYE	Pos
9044	2/20/14	F	20.0	KANYAN	Neg
9045	2/20/14	F	15.0	NDUGHUTU	Neg
9046	2/20/14	F	0.3	BUGOYE	Neg
9047	2/20/14	F	19.0	IBANDA	Pos
9048	2/20/14	F	14.0	NDUGHUTU	Neg
9049	2/20/14	F	3.3	BUGOYE	Neg
9050	2/20/14	F	2.0	KISAMBA	Neg
9051	2/20/14	F	23.0	IZINGA	Neg
9052	2/20/14	F	22.0	BUNYANGONI	Neg
9053	2/20/14	M	5.0	BUNYANGONI	Neg
9054	2/20/14	M	17.0	MALIBA	Pos
9055	2/20/14	F	62.0	KANYAN	Neg
9056	2/20/14	M	30.0	RWAKING	Neg
9057	2/20/14	M	0.8	BUGOYE	Neg
9058	2/20/14	M	15.0	BUGOYE	Neg
9059	2/20/14	F	40.0	IZINGA	Pos
9060	2/20/14	M	10.0	IZINGA	Pos
9061	2/20/14	M	46.0	MUHAMBO	Pos
9062	2/20/14	F	40.0	BUGOYE	Neg
9063	2/20/14	F	20.0	BUGOYE	Neg
9064	2/20/14	M	8.0	BUGOYE	Pos
9065	2/20/14	M	2.0	MURAMBA	Neg
9066	2/20/14	M	13.0	MURAMBA	Neg
9067	2/20/14	F	12.0	BUGOYE	Neg
9068	2/20/14	F	12.0	BUNYANGONI	Neg
9069	2/20/14	F	3.0	BUGOYE	Neg
9070	2/20/14	F	11.0	BUGOYE	Neg
9071	2/20/14	F	10.0	KANYAN	Pos
9072	2/20/14	F	10.0	BUGOYE	Pos

9073	2/20/14	M	4.0	BUGOYE	Neg
9074	2/20/14	F	7.0	KANYAN	Pos
9075	2/20/14	F	16.0	BUGOYE	Neg
9076	2/20/14	F	20.0	MUBUKU	Neg
9077	2/20/14	F	18.0	BUGOYE	Pos
9078	2/20/14	F	46.0	KANYAN	Pos
9079	2/20/14	F	46.0	IZINGA	Neg
9080	2/20/14	F	6.0	BUGOYE	Neg
9081	2/20/14	F	80.0	KAKINDO	Neg
9082	2/20/14	M	4.0	BUGOYE	Neg
9083	2/20/14	F	60.0	BUGOYE	Neg
9084	2/20/14	M	10.0	NDUGHUTU	Pos
9085	2/20/14	F	36.0	MALIBA	Pos
9086	2/20/14	F	32.0	KYARUGOMOKA	Neg
9087	2/20/14	F	3.5	KYARUGOMOKA	Pos
9088	2/20/14	M	2.5	KYARUGOMOKA	Pos
9089	2/20/14	F	8.0	KYARUGOMOKA	Neg
9090	2/20/14	F	5.0	IZINGA	Neg
9091	2/20/14	F	18.0	KISOJO	Pos
9092	2/20/14	M	5.0	MALIBA	Neg
9093	2/20/14	F	30.0	BUGOYE	Neg
9094	2/20/14	F	1.3	MURAMBA	Neg
9095	2/20/14	F	34.0	KANYAN	Pos
9096	2/20/14	F	21.0	NYAKABUGHA	Neg
9097	2/20/14	M	6.3	IZINGA	Pos
9098	2/20/14	M	4.3	IZINGA	Neg
9099	2/20/14	F	1.5	IZINGA	Neg
9100	2/20/14	F	34.0	IZINGA	Neg
9101	2/20/14	F	26.0	NDUGHUTU	Neg
9102	2/20/14	M	14.0	KYARUGOMOKA	Pos
9103	2/20/14	F	34.0	IZINGA	Pos
9104	2/20/14	F	1.3	NDUGHUTU	Neg
9105	2/20/14	M	47.0	BUNYANGONI	Pos
9106	2/20/14	M	5.0	BUGOYE	Pos
9107	2/20/14	F	40.0	BUHUNGA	Neg
9108	2/20/14	F	23.0	NDUGHUTU	Neg
9109	2/20/14	M	11.0	MURAMBA	Neg
9110	2/20/14	F	4.0	RWAKING	Pos
9111	2/20/14	F	1.0	BIKONE	Neg
9112	2/20/14	F	22.0	NYAMBUKU	Neg
9113	2/20/14	F	14.0	KANYAN	Pos
9114	2/20/14	M	8.0	MAGHOMA	Neg
9115	2/20/14	F	10.0	BUGOYE	Neg

9116	2/20/14	F	30.0	MUBUKU	Neg	
9117	2/20/14	M	8.0	BUNYANGONI	Pos	
9118	2/20/14	F	31.0	BUNYANGONI	Pos	
9119	2/20/14	F	16.0	KANYAN	Neg	
9120	2/20/14	M	24.0	KYANYA	Neg	
9121	2/20/14	M	23.0	BUGOYE	Neg	
9122	2/24/14	M	24.0	BUGOYE	Pos	
9123	2/24/14	M	19.0	MURAMBA	Neg	
9124	2/24/14	F	20.0	BUGOYE	Pos	
9125	2/24/14	M	26.0	IZINGA	Neg	
9126	2/24/14	M	23.0	MURAMBA	Neg	
9127	2/24/14	F	19.0	IBANDA	Neg	
9128	2/24/14	M	13.0	MURAMBA	Pos	
9129	2/24/14	M	14.0	MURAMBA	Neg	
9130	2/24/14	F	7.0	MURAMBA	Neg	
9131	2/24/14	F	12.0	MURAMBA	Pos	
9132	2/24/14	M	21.0	BUGOYE	Neg	
9133	2/24/14	M	1.0	KIHARARA	Neg	
9134	2/24/14	F	26.0	IZINGA	Pos	
9135	2/24/14	F	2.0	MURAMBA	Pos	Pos
9136	2/24/14	F	22.0	IZINGA	Pos	
9137	2/24/14	M	5.0	BUNYANGONI	Neg	
9138	2/24/14	M	31.0	IBANDA	Pos	
9139	2/24/14	M	12.0	IBANDA	Pos	
9140	2/24/14	F	32.0	BUGOYE	Neg	
9141	2/24/14	M	9.0	KAKINDO	Pos	
9142	2/24/14	M	15.0	MUHAMBO	Neg	
9143	2/24/14	F	15.0	BUGOYE	Pos	
9144	2/24/14	F	0.3	BUGOYE	Neg	
9145	2/24/14	F	24.0	BUGOYE	Neg	
9146	2/24/14	F	28.0	BUGOYE	Pos	
9147	2/24/14	M	13.0	MURAMBA	Neg	
9148	2/24/14	M	10.0	IZINGA	Neg	
9149	2/24/14	F	13.0	MURAMBA	Pos	
9150	2/24/14	F	10.0	KANYAN	Pos	
9151	2/24/14	F	14.0	KANYAN	Pos	
9152	2/24/14	F	30.0	KYADIMA	Pos	
9153	2/24/14	M	10.0	IHANI	Neg	
9154	2/24/14	M	15.0	RWAKING	Pos	
9155	2/24/14	M	11.0	RWAKING	Pos	
9156	2/24/14	M	10.0	KEMIHOKO	Pos	Pos
9157	2/24/14	F	25.0	NYAKABUGHA	Neg	
9158	2/24/14	F	12.0	BUNYANGONI	Neg	

9159	2/24/14	M	13.0	MURAMBA	Neg
9160	2/24/14	F	3.8	BUGOYE	Pos
9161	2/24/14	F	16.0	BIKONE	Neg
9162	2/24/14	F	12.0	MURAMBA	Pos
9163	2/24/14	F	11.0	BUNYANGONI	Neg
9164	2/24/14	F	3.0	NYANGONGE	Neg
9165	2/24/14	F	44.0	KIHINDI	Neg
9166	2/24/14	M	4.5	KIHINDI	Neg
9167	2/24/14	F	41.0	KIHINDI	Neg
9168	2/24/14	F	49.0	IBANDA	Neg
9169	2/24/14	F	65.0	IZINGA	Pos
9170	2/24/14	F	24.0	KYARUGOMOKA	Neg
9171	2/24/14	F	1.5	IBANDA	Pos
9172	2/24/14	F	24.0	IZINGA	Pos
9173	2/24/14	F	1.5	IZINGA	Neg
9174	2/24/14	M	0.5	IZINGA	Pos
9175	2/24/14	F	18.0	IZINGA	Pos
9176	2/24/14	F	17.0	IBANDA	Neg
9177	2/25/14	F	29.0	KANYAN	Neg
9178	2/25/14	F	40.0	MURAMBA	Neg
9179	2/25/14	F	10.0	BUGOYE	Neg
9180	2/25/14	F	7.0	BUGOYE	Pos
9181	2/25/14	M	8.0	IZINGA	Pos
9182	2/25/14	F	13.0	IZINGA	Pos
9183	2/25/14	F	11.0	IZINGA	Pos
9184	2/25/14	M	17.0	BUZIBWERA	Pos
9185	2/25/14	M	17.0	BUZIBWERA	Pos
9186	2/25/14	M	13.0	BUGOYE	Pos
9187	2/25/14	F	2.5	BUNYANGONI	Neg
9188	2/25/14	F	0.8	BUNYANGONI	Neg
9189	2/25/14	F	11.0	BUNYANGONI	Neg
9190	2/25/14	F	8.0	IZINGA	Pos
9191	2/25/14	F	50.0	NYAMBUKU	Neg
9192	2/25/14	M	0.5	RWAKING	Neg
9193	2/25/14	M	45.0	KATOOKE	Neg
9194	2/25/14	F	65.0	BUGOYE	Neg
9195	2/25/14	F	13.0	IZINGA	Pos
9196	2/25/14	F	9.0	MURAMBA	Neg
9197	2/25/14	F	14.0	MURAMBA	Pos
9198	2/25/14	F	0.8	MUHAMBO	Neg
9199	2/25/14	F	7.0	IZINGA	Pos
9200	2/25/14	F	0.8	KANYAN	Neg
9201	2/25/14	F	20.0	KAKINDO	Neg

9202	2/25/14	F	32.0	RWAKING	Neg
9203	2/25/14	F	37.0	KIKUBUGALI	Neg
9204	2/25/14	F	13.0	BUNYANGONI	Neg
9205	2/25/14	F	13.0	BUGOYE	Neg
9206	2/26/14	F	8.0	IZINGA	Neg
9207	2/26/14	M	9.0	IZINGA	Pos
9208	2/26/14	M	22.0	KIKOKERA	Pos
9209	2/26/14	M	6.0	IZINGA	Pos
9210	2/26/14	F	44.0	IZINGA	Neg
9211	2/26/14	F	16.0	ISENGERA	Neg
9212	2/26/14	F	1.8	MAKRIMIANI	Pos
9213	2/26/14	M	4.0	MALIBA	Pos
9214	2/26/14	M	70.0	MUBUKU	Neg
9215	2/26/14	F	22.0	IZINGA	Pos
9216	2/26/14	F	35.0	BUNYANGONI	Neg
9217	2/26/14	M	1.5	KYANYA	Pos
9218	2/26/14	M	3.0	BUNYANGONI	Neg
9219	2/26/14	F	13.0	BUNYANGONI	Neg
9220	2/26/14	F	22.0	BUGOYE	Neg
9221	2/26/14	F	20.0	IBANDA	Pos
9222	2/26/14	F	1.5	BUGOYE	Neg
9223	2/26/14	F	16.0	KAKINDO	Neg
9224	2/26/14	F	40.0	KISAMBA	Neg
9225	2/26/14	M	28.0	RWAKING	Neg
9226	2/26/14	F	24.0	KAKINDO	Neg
9227	2/26/14	F	20.0	KAKINDO	Neg
9228	2/26/14	M	19.0	MURAMBA	Pos
9229	2/26/14	M	5.0	BUGOYE	Neg
9230	2/26/14	F	0.8	KISAMBA	Neg
9231	2/26/14	F	5.0	IBANDA	Neg
9232	2/26/14	M	6.0	MALIBA	Pos
9233	2/26/14	M	5.0	BUNYANGONI	Neg
9234	2/26/14	F	58.0	KANYAN	Neg
9235	2/26/14	M	26.0	MALIBA	Neg
9236	2/26/14	F	35.0	MURAMBA	Pos
9237	2/26/14	F	35.0	KYANYA	Neg
9238	2/26/14	F	23.0	BUGOYE	Neg
9239	2/26/14	M	57.0	KISAMBA	Neg
9240	2/26/14	F	34.0	MALIBA	Neg
9241	2/26/14	M	12.0	IZINGA	Pos
9242	2/26/14	F	15.0	NYANGONGE	Neg
9243	2/26/14	F	28.0	BUGOYE	Neg
9244	2/26/14	M	20.0	BUGOYE	Neg

9245	2/26/14	M	3.0	NYANGONGE	Neg
9246	2/26/14	M	11.0	BUZIBWERA	Pos
9247	2/26/14	F	13.0	MUHAMBO	Neg
9248	2/26/14	F	10.0	MUHAMBO	Neg
9249	2/26/14	M	8.0	MUHAMBO	Neg
9250	2/26/14	F	18.0	KIRONGO	Neg
9251	2/26/14	F	24.0	KATOOKE	Neg
9252	2/26/14	F	27.0	IBANDA	Pos
9253	2/26/14	M	30.0	KAKINDO	Neg
9254	2/26/14	M	37.0	KAKINDO	Neg
9255	2/26/14	M	73.0	MURAMBA	Pos
9256	2/26/14	M	1.0	MURAMBA	Neg
9257	2/26/14	F	13.0	BUGOYE	Neg
9258	2/26/14	F	14.0	BUGOYE	Neg
9259	2/26/14	F	7.0	BUGOYE	Neg
9260	2/26/14	F	6.0	RWAKING	Pos
9261	2/26/14	F	1.3	KIHINDI	Neg
9262	2/26/14	M	0.3	RWAKING	Neg
9263	2/26/14	F	36.0	KYANYA	Pos
9264	2/26/14	M	2.0	MALIBA	Neg
9265	2/26/14	F	29.0	MALIBA	Neg
9266	2/26/14	F	1.5	KYANYA	Neg
9267	2/28/14	F	21.0	MEWLHO	Pos
9268	2/28/14	M	17.0	NYAKABUGHA	Neg
9269	2/28/14	F	20.0	RWAKING	Neg
9270	2/28/14	F	11.0	KAKINDO	Pos
9271	2/28/14	M	18.0	NDUGHUTU	Pos
9272	2/28/14	M	11.0	MURAMBA	Neg
9273	2/28/14	M	29.0	IBANDA	Pos
9274	2/28/14	F	42.0	KAKINDO	Pos
9275	2/28/14	F	22.0	NDUGHUTU	Pos
9276	2/28/14	F	40.0	NYAKABUGHA	Neg
9277	2/28/14	F	30.0	MUBUKU	Neg
9278	2/28/14	F	0.5	KANYAN	Neg
9279	2/28/14	F	18.0	MAGHOMA	Neg
9280	2/28/14	M	1.3	MURAMBA	Neg
9281	2/28/14	F	12.0	KYAMOBA	Neg
9282	2/28/14	F	6.0	NDUGHUTU	Neg
9283	2/28/14	F	5.0	RWAKING	Neg
9284	2/28/14	M	40.0	BUGOYE	Neg
9285	2/28/14	F	20.0	RWAKING	Neg
9286	2/28/14	F	2.0	RWAKING	Neg
9287	2/28/14	M	43.0	BUGOYE	Neg

9288	2/28/14	F	6.0	BUGOYE	Neg	
9289	2/28/14	F	65.0	MALIBA	Neg	
9290	2/28/14	F	40.0	KIHARARA	Neg	
9291	2/28/14	F	22.0	IBANDA	Neg	
9292	2/28/14	F	11.0	BUZIBWERA	Neg	
9293	2/28/14	F	7.0	BUZIBWERA	Pos	
9294	2/28/14	F	5.0	BUZIBWERA	Pos	
9295	2/28/14	M	4.0	BUZIBWERA	Neg	
9296	2/28/14	F	50.0	BUZIBWERA	Neg	
9297	2/28/14	F	3.0	KATUMBA	Pos	
9298	2/28/14	F	17.0	BUGOYE	Neg	
9299	2/28/14	F	2.5	MURAMBA	Neg	Neg
9300	2/28/14	M	1.3	KASESE	Neg	
9301	2/28/14	F	26.0	IHANI	Pos	
9302	2/28/14	F	35.0	NYAKABUGHA	Neg	
9303	2/28/14	F	20.0	KANYAN	Neg	
9304	3/1/14	M	69.0	BUZIBWERA	Neg	
9305	3/1/14	M	42.0	IZINGA	Pos	
9306	3/1/14	F	27.0	KANYAN	Neg	
9307	3/1/14	M	75.0	KISANGA	Pos	
9308	3/1/14	F	38.0	KANYAN	Pos	
9309	3/1/14	F	5.0	KISOJO	Pos	
9310	3/1/14	M	3.5	NYAKARUNGU	Pos	
9311	3/1/14	F	40.0	NYAKARUNGU	Neg	
9312	3/1/14	M	45.0	MURAMBA	Neg	
9313	3/1/14	F	0.3	NDUGHUTU	Neg	
9314	3/1/14	F	3.0	KANYAN	Neg	
9315	3/1/14	F	6.0	KANYAN	Pos	
9316	3/1/14	F	28.0	RWAKING	Neg	
9317	3/1/14	M	0.8	RWAKING	Pos	
9318	3/1/14	F	27.0	BUGOYE	Neg	
9319	3/1/14	M	35.0	RWAKING	Neg	
9320	3/1/14	M	73.0	NDUGHUTU	Neg	
9321	3/1/14	F	3.0	KANYAN	Neg	
9322	3/1/14	F	54.0	KANYAN	Neg	
9323	3/1/14	F	14.0	MURAMBA	Neg	
9324	3/1/14	F	25.0	RWAKING	Neg	
9325	3/1/14	F	15.0	NDUGHUTU	Neg	
9326	3/1/14	M	9.0	NDUGHUTU	Neg	
9327	3/1/14	F	20.0	BUNYANGONI	Neg	
9328	3/1/14	F	11.0	IBANDA	Pos	
9329	3/1/14	F	2.8	BUNYANGONI	Pos	
9330	3/1/14	M	14.0	MURAMBA	Pos	

9331	3/1/14	F	32.0	BUGOYE	Neg
9332	3/1/14	F	0.8	IBANDA	Neg
9333	3/1/14	F	23.0	BUGOYE	Neg
9334	3/1/14	F	5.0	IBANDA	Pos
9335	3/1/14	F	0.8	BUGOYE	Neg
9336	3/1/14	M	8.0	BUNYANGONI	Neg
9337	3/1/14	F	14.0	KATOOKE	Neg
9338	3/1/14	F	60.0	KAKINDO	Neg
9339	3/1/14	M	8.0	KATUMBA	Neg
9340	3/1/14	F	30.0	KATUMBA	Neg
9341	3/1/14	M	1.5	NDUGHUTU	Neg
9342	3/1/14	F	40.0	KIRULI	Neg
9343	3/1/14	F	58.0	BUGOYE	Neg
9344	3/1/14	F	60.0	IZINGA	Neg
9345	3/1/14	M	20.0	KATOOKE	Pos
9346	3/1/14	F	0.3	RWAKING	Neg
9347	3/1/14	F	20.0	BUGOYE	Neg
9348	3/1/14	F	17.0	KANYAN	Neg
9349	3/1/14	M	0.5	KANYAN	Neg
9350	3/1/14	M	5.0	BUGOYE	Neg
9351	3/1/14	F	3.5	KISOJO	Neg
9352	3/1/14	F	10.0	KISOJO	Neg
9353	3/1/14	M	4.0	BUGOYE	Pos
9354	3/1/14	F	24.0	MUHAMBO	Neg
9355	3/1/14	F	13.0	NDUGHUTU	Neg
9356	3/1/14	M	0.5	KATUMBA	Neg
9357	3/1/14	M	2.5	MURAMBA	Pos
9358	3/1/14	F	14.0	BUGOYE	Neg
9359	3/1/14	F	11.0	BUGOYE	Pos
9360	3/1/14	F	4.0	MUHAMBO	Neg
9361	3/1/14	F	25.0	NDUGHUTU	Neg
9362	3/1/14	F	29.0	RWAKING	Neg
9363	3/1/14	F	2.0	RWAKING	Neg
9364	3/1/14	M	28.0	IBANDA	Neg
9365	3/1/14	F	2.0	RWAKING	Neg
9366	3/1/14	M	0.3	RWAKING	Neg
9367	3/1/14	F	9.0	NDUGHUTU	Neg
9368	3/1/14	F	4.0	NDUGHUTU	Pos
9369	3/1/14	F	30.0	MUHAMBO	Neg
9370	3/1/14	F	19.0	KANYAN	Pos
9371	3/1/14	M	1.3	NDUGHUTU	Neg
9372	3/1/14	F	11.0	IZINGA	Pos
9373	3/1/14	F	40.0	NDUGHUTU	Neg

9374	3/1/14	F	7.0	NDUGHUTU	Pos
9375	3/1/14	F	12.0	MUHAMBO	Pos
9376	3/1/14	M	14.0	KANYAN	Pos
9377	3/1/14	F	6.0	MALIBA	Pos
9378	3/1/14	F	10.0	IZINGA	Pos
9379	3/1/14	F	40.0	IZINGA	Pos
9380	3/1/14	F	21.0	KIBIRIZI	Pos
9381	3/1/14	F	36.0	KATOOKE	Neg
9382	3/1/14	F	46.0	KATOOKE	Neg
9383	3/1/14	F	25.0	MUWUER	Neg
9384	3/1/14	F	10.0	KYANYA	Neg
9385	3/1/14	F	10.0	MURAMBA	Neg
9386	3/1/14	M	1.0	MURAMBA	Neg
9387	3/1/14	M	1.5	MURAMBA	Pos
9388	3/1/14	F	4.0	BUNYANGONI	Neg
9389	3/1/14	F	2.5	MALIBA	Neg
9390	3/1/14	F	7.5	BUGOYE	Neg
9391	3/1/14	F	4.0	MURAMBA	Neg
9392	3/1/14	F	25.0	NYAKABUGHA	Neg
9393	3/1/14	F	1.3	BUGOYE	Neg
9394	3/1/14	F	0.3	BUGOYE	Pos
9395	3/1/14	M	3.0	BUGOYE	Neg
9396	3/1/14	F	50.0	BUGOYE	Neg
9397	3/1/14	M	15.0	BUGOYE	Pos
9398	3/1/14	M	25.0	NYAKARUNGU	Pos
9399	3/1/14	F	0.3	NYAKARUNGU	Neg
9400	3/1/14	M	38.0	BUGOYE	Neg
9401	3/1/14	M	1.3	MAGHOMA	Neg
9402	3/1/14	M	1.5	MUHAMBO	Pos
9403	3/4/14	F	0.3	NYAKARUNGU	Pos
9404	3/4/14	M	52.0	BUGOYE	Pos
9405	3/4/14	F	26.0	MURAMBA	Neg
9406	3/4/14	M	62.0	MURAMBA	Neg
9407	3/4/14	F	34.0	KISANZI	Neg
9408	3/4/14	F	72.0	BUGOYE	Neg
9409	3/4/14	F	46.0	MUBUKU	Neg
9410	3/4/14	F	36.0	BUGOYE	Neg
9411	3/4/14	F	60.0	BUGOYE	Pos
9412	3/4/14	F	3.0	BUGOYE	Neg
9413	3/4/14	F	22.0	BUGOYE	Pos
9414	3/4/14	F	32.0	BUGOYE	Pos
9415	3/4/14	F	14.0	IBANDA	Neg
9416	3/4/14	F	10.0	BUGOYE	Pos

9417	3/4/14	F	50.0	BUNYANGONI	Neg
9418	3/4/14	F	28.0	MURAMBA	Neg
9419	3/4/14	F	7.0	BUZIBWERA	Pos
9420	3/4/14	F	1.3	RWAKING	Pos
9421	3/4/14	M	36.0	RWAKING	Neg
9422	3/4/14	F	82.0	RUKOKI	Pos
9423	3/4/14	M	1.8	RUKOKI	Pos
9424	3/4/14	F	0.8	KIRULI	Pos
9425	3/4/14	F	23.0	RWAKING	Neg
9426	3/4/14	M	13.0	BUGOYE	Pos
9427	3/4/14	F	75.0	IBANDA	Neg
9428	3/4/14	M	17.0	BUGOYE	Neg
9429	3/4/14	F	28.0	NDUGHUTU	Neg
9430	3/4/14	F	1.3	NDUGHUTU	Neg
9431	3/4/14	F	21.0	NDUGHUTU	Neg
9432	3/4/14	M	41.0	NDUGHUTU	Neg
9433	3/4/14	F	26.0	KIKOKERA	Pos
9434	3/4/14	F	27.0	KASESE	Neg
9435	3/4/14	F	42.0	BIKONE	Neg
9436	3/4/14	F	0.3	RWAKING	Neg
9437	3/4/14	F	8.0	RWAKING	Pos
9438	3/4/14	F	22.0	BUGOYE	Neg
9439	3/4/14	F	50.0	BUZIBWERA	Neg
9440	3/4/14	F	1.3	KISOJO	Neg
9441	3/4/14	M	9.0	RWAKING	Pos
9442	3/4/14	F	32.0	RWAKING	Neg
9443	3/4/14	F	70.0	KISAMBA	Neg
9444	3/4/14	F	18.0	IBANDA	Neg
9445	3/4/14	M	4.0	KIRULI	Neg
9446	3/4/14	F	3.0	KIRULI	Neg
9447	3/4/14	F	17.0	NDUGHUTU	Neg
9448	3/4/14	F	1.0	NDUGHUTU	Neg
9449	3/4/14	M	1.3	BUZIBWERA	Neg
9450	3/4/14	M	48.0	IBANDA	Neg
9451	3/4/14	F	17.0	BUGOYE	Neg
9452	3/4/14	F	7.0	BUGOYE	Neg
9453	3/4/14	F	11.0	BUGOYE	Neg
9454	3/4/14	F	29.0	BUGOYE	Neg
9455	3/4/14	M	58.0	BUNYANGONI	Neg
9456	3/4/14	F	9.0	IZINGA	Pos
9457	3/4/14	F	15.0	KANYAN	Neg
9458	3/4/14	F	30.0	BUGOYE	Pos
9459	3/4/14	F	5.0	BUGOYE	Neg

9460	3/4/14	F	49.0	BUZIBWERA	Neg	
9461	3/4/14	F	31.0	IZINGA	Pos	
9462	3/4/14	F	6.0	BUGOYE	Pos	
9463	3/4/14	M	63.0	BUZIBWERA	Pos	
9464	3/4/14	F	58.0	KIRULI	Pos	
9465	3/4/14	M	42.0	KATUMBA	Pos	
9466	3/5/14	F	34.0	KANYAN	Neg	
9467	3/5/14	M	44.0	KANYAN	Neg	
9468	3/5/14	M	55.0	KANYAN	Neg	
9469	3/5/14	F	33.0	RWAKING	Neg	
9470	3/5/14	F	39.0	IBANDA	Pos	
9471	3/5/14	M	55.0	RWAKING	Neg	
9472	3/5/14	F	50.0	KANYAN	Pos	
9473	3/5/14	M	8.0	IZINGA	Pos	
9474	3/5/14	F	19.0	RWAKING	Pos	
9475	3/5/14	F	12.0	MUBUKU	Pos	
9476	3/5/14	M	11.0	MUBUKU	Pos	
9477	3/5/14	M	2.0	KATUMBA	Neg	
9478	3/5/14	M	3.0	NDUGHUTU	Neg	
9479	3/5/14	F	20.0	RWAKING	Pos	
9480	3/5/14	F	19.0	IZINGA	Pos	
9481	3/5/14	M	2.0	IZINGA	Pos	
9482	3/5/14	M	4.5	IZINGA	Pos	
9483	3/5/14	M	0.5	BUGOYE	Neg	
9484	3/5/14	M	21.0	IZINGA	Neg	
9485	3/5/14	F	12.0	BUNYANGONI	Neg	
9486	3/5/14	M	27.0	BUZIBWERA	Neg	
9487	3/5/14	M	0.3	KATUMBA	Neg	
9488	3/5/14	M	1.3	KASESE	Neg	
9489	3/5/14	M	13.0	IZINGA	Neg	
9490	3/5/14	M	20.0	BUZIBWERA	Pos	
9491	3/5/14	M	0.5	MURAMBA	Pos	
9492	3/5/14	M	0.3	MURAMBA	Neg	
9493	3/5/14	M	1.3	BUNYANGONI	Neg	
9494	3/5/14	F	22.0	IZINGA	Pos	Pos
9495	3/5/14	M	0.5	KANYAN	Neg	
9496	3/5/14	F	7.0	MUKATTI	Neg	
9497	3/5/14	F	22.0	KISAMBA	Pos	
9498	3/5/14	M	1.3	MURAMBA	Neg	
9499	3/5/14	M	11.0	MURAMBA	Pos	
9500	3/5/14	F	50.0	MURAMBA	Pos	
9501	3/5/14	F	8.0	BUNYANGONI	Pos	
9502	3/5/14	F	38.0	RWAKING	Neg	

9503	3/5/14	F	20.0	NDUGHUTU	Pos	
9504	3/5/14	F	19.0	BUNYANGONI	Pos	
9505	3/5/14	F	0.5	BUNYANGONI	Pos	
9506	3/5/14	F	0.5	RWAKING	Neg	
9507	3/5/14	F	23.0	KAKINDO	Neg	
9508	3/5/14	F	5.0	KISAMBA	Pos	
9509	3/5/14	F	3.0	IZINGA	Pos	
9510	3/5/14	M	2.0	IZINGA	Pos	Pos
9511	3/5/14	M	5.0	IZINGA	Pos	
9512	3/5/14	M	2.5	IZINGA	Pos	
9513	3/5/14	F	1.8	BUZIBWERA	Neg	
9514	3/5/14	F	45.0	NDUGHUTU	Neg	
9515	3/5/14	F	18.0	.	Pos	
9516	3/5/14	M	4.0	MURAMBA	Pos	
9517	3/5/14	M	8.0	BUGOYE	Pos	
9518	3/5/14	M	1.8	BUGOYE	Neg	
9519	3/5/14	M	55.0	BULYANDEKE	Neg	
9520	3/5/14	M	40.0	BUGOYE	Neg	
9521	3/6/14	M	8.0	IZINGA	Pos	Pos
9522	3/6/14	F	22.0	BUGOYE	Neg	
9523	3/6/14	F	9.0	IZINGA	Pos	Pos
9524	3/6/14	F	19.0	BUGOYE	Neg	
9525	3/6/14	M	3.0	MURAMBA	Pos	
9526	3/6/14	M	38.0	IZINGA	Neg	
9527	3/6/14	M	7.0	IHANI	Neg	
9528	3/6/14	M	2.0	KAKINDO	Neg	
9529	3/6/14	M	3.0	KAKINDO	Neg	
9530	3/6/14	F	40.0	KAKINDO	Neg	
9531	3/6/14	M	2.5	BUNYANGONI	Neg	
9532	3/6/14	F	20.0	MURAMBA	Pos	
9533	3/6/14	F	2.3	.	Neg	
9534	3/6/14	F	18.0	KIKOKERA	Pos	
9535	3/6/14	F	28.0	KIKOKERA	Neg	
9536	3/6/14	F	1.5	IBANDA	Pos	
9537	3/6/14	F	12.0	KYANYA	Neg	
9538	3/6/14	M	32.0	BUGOYE	Neg	
9539	3/6/14	F	5.0	MURAMBA	Pos	
9540	3/6/14	F	28.0	KIRULI	Neg	
9541	3/6/14	F	30.0	KANYAN	Neg	
9542	3/6/14	F	18.0	BUGOYE	Neg	
9543	3/6/14	F	38.0	RWAKING	Pos	
9544	3/6/14	F	63.0	RWAKING	Neg	
9545	3/6/14	F	44.0	IZINGA	Neg	

9546	3/6/14	F	6.0	IZINGA	Pos
9547	3/6/14	F	28.0	BUGOYE	Neg
9548	3/6/14	F	16.0	NDUGHUTU	Pos
9549	3/6/14	F	65.0	IHANI	Pos
9550	3/6/14	F	53.0	MUHAMBO	Pos
9551	3/6/14	F	40.0	MALIBA	Neg
9552	3/6/14	F	9.0	MALIBA	Pos
9553	3/6/14	F	8.0	KAKINDO	Pos
9554	3/6/14	M	6.0	KAKINDO	Pos
9555	3/6/14	F	12.0	KAKINDO	Pos
9556	3/6/14	M	8.0	IZINGA	Pos
9557	3/6/14	F	45.0	IZINGA	Neg
9558	3/6/14	M	2.3	MURAMBA	Pos
9559	3/6/14	F	21.0	MURAMBA	Pos
9560	3/6/14	M	8.0	NYAKABUGHA	Neg
9561	3/6/14	M	22.0	MUBUKU	Pos
9562	3/6/14	F	0.8	MALIBA	Neg
9563	3/6/14	M	21.0	BUGOYE	Neg
9564	3/6/14	F	0.8	KANYAN	Pos
9565	3/7/14	F	62.0	KIGORO	Pos
9566	3/7/14	F	32.0	RWAKING	Neg
9567	3/7/14	M	49.0	KANYAN	Neg
9568	3/7/14	F	1.8	KANYAN	Pos
9569	3/7/14	F	24.0	BUGOYE	Pos
9570	3/7/14	F	30.0	IZINGA	Pos
9571	3/7/14	F	30.0	BUGOYE	Neg
9572	3/7/14	F	13.0	MURAMBA	Pos
9573	3/7/14	F	40.0	MUBUKU	Pos
9574	3/7/14	F	34.0	NDUGHUTU	Neg
9575	3/7/14	F	22.0	KYARUGOMOKA	Pos
9576	3/7/14	M	38.0	MURAMBA	Pos
9577	3/7/14	F	16.0	KYANYA	Pos
9578	3/7/14	M	62.0	BUNYANGONI	Pos
9579	3/7/14	F	19.0	IZINGA	Pos
9580	3/7/14	M	0.3	NDUGHUTU	Neg
9581	3/7/14	F	23.0	NDUGHUTU	Neg
9582	3/7/14	M	40.0	KATUMBA	Neg
9583	3/7/14	F	24.0	MALIBA	Neg
9584	3/7/14	F	46.0	MUHAMBO	Neg
9585	3/7/14	F	30.0	MUBUKU	Neg
9586	3/7/14	M	48.0	KASANDERA	Neg
9587	3/7/14	F	19.0	NDUGHUTU	Neg
9588	3/7/14	F	50.0	MALIBA	Pos

9589	3/7/14	F	0.3	IBANDA	Neg
9590	3/7/14	M	4.0	NDUGHUTU	Neg
9591	3/7/14	M	44.0	NYAKABUGHA	Neg
9592	3/7/14	F	3.5	KANYAN	Pos
9593	3/7/14	M	7.0	KANYAN	Pos
9594	3/7/14	F	26.0	NDUGHUTU	Neg
9595	3/7/14	F	3.0	NDUGHUTU	Neg
9596	3/7/14	F	0.5	NDUGHUTU	Neg
9597	3/7/14	F	9.0	KAKINDO	Pos
9598	3/7/14	F	46.0	KAKINDO	Neg
9599	3/7/14	M	0.8	MURAMBA	Neg
9600	3/7/14	F	51.0	KANYAN	Neg
9601	3/7/14	F	14.0	BUZIBWERA	Pos
9602	3/7/14	M	26.0	MALIBA	Neg
9603	3/7/14	F	20.0	KANYAN	Neg
9604	3/7/14	F	50.0	NDUGHUTU	Pos
9605	3/7/14	M	19.0	BUNYANGONI	Neg
9606	3/7/14	F	48.0	KIRULI	Neg
9607	3/7/14	F	52.0	KISAMBA	Neg
9608	3/7/14	M	5.0	BUNYANGONI	Pos
9609	3/7/14	F	20.0	KATOOKE	Pos
9610	3/7/14	M	1.8	BUNYANGONI	Neg
9611	3/7/14	F	0.8	BUGOYE	Neg
9612	3/7/14	F	30.0	KYANYA	Neg
9613	3/7/14	F	3.8	KIKOKERA	Neg
9614	3/7/14	F	11.0	KIKOKERA	Neg
9615	3/7/14	M	19.0	BUGOYE	Neg
9616	3/7/14	M	4.0	MURAMBA	Neg
9617	3/7/14	F	25.0	NDUGHUTU	Neg
9618	3/7/14	F	17.0	KYANYA	Neg
9619	3/7/14	F	30.0	KANYAN	Neg
9620	3/7/14	F	22.0	IZINGA	Pos
9621	3/7/14	F	3.0	KATOOKE	Neg
9622	3/7/14	F	1.8	RWAKING	Pos
9623	3/7/14	F	0.5	IZINGA	Pos
9624	3/7/14	M	4.0	BUGOYE	Pos
9625	3/7/14	M	1.0	KYARUGOMOKA	Neg
9626	3/7/14	F	11.0	KYARUGOMOKA	Pos
9627	3/7/14	F	3.0	IZINGA	Pos
9628	3/7/14	F	5.5	IZINGA	Pos
9629	3/7/14	M	4.5	IZINGA	Pos
9630	3/7/14	F	8.0	IZINGA	Pos
9631	3/7/14	M	11.0	NDUGHUTU	Pos

9632	3/7/14	F	29.0	BUNYANGONI	Neg	
9633	3/7/14	F	0.5	RWAKING	Neg	
9634	3/7/14	M	32.0	NDUGHUTU	Neg	
9635	3/7/14	F	52.0	BUGOYE	Pos	
9636	3/7/14	F	8.0	BUGOYE	Neg	
9637	3/7/14	F	41.0	BUGOYE	Neg	
9638	3/7/14	F	13.0	KAKINDO	Pos	
9639	3/7/14	F	4.0	IZINGA	Pos	
9640	3/7/14	M	9.0	KANYAN	Pos	
9641	3/7/14	F	14.0	MURAMBA	Neg	
9642	3/7/14	F	18.0	KANYAN	Neg	
9643	3/7/14	F	6.0	NDUGHUTU	Neg	
9644	3/7/14	M	4.0	NDUGHUTU	Neg	
9645	3/7/14	M	13.0	BUGOYE	Neg	
9646	3/7/14	F	18.0	KAKINDO	Neg	
9647	3/7/14	F	16.0	MUBUKU	Pos	
9648	3/7/14	F	27.0	MUBUKU	Pos	
9649	3/7/14	F	8.0	IZINGA	Pos	
9650	3/7/14	F	19.0	MALIBA	Pos	
9651	3/11/14	F	2.3	IZINGA	Pos	Pos
9652	3/11/14	F	22.0	NDUGHUTU	Neg	
9653	3/11/14	F	50.0	IZINGA	Neg	
9654	3/11/14	F	40.0	IZINGA	Pos	
9655	3/11/14	F	5.0	IZINGA	Neg	
9656	3/11/14	F	3.0	MALIBA	Pos	
9657	3/11/14	F	17.0	NDUGHUTU	Neg	
9658	3/11/14	F	54.0	BUGOYE	Neg	
9659	3/11/14	M	43.0	IBANDA	Neg	
9660	3/11/14	F	2.0	MALIBA	Pos	
9661	3/11/14	M	19.0	IZINGA	Pos	
9662	3/11/14	F	33.0	RWAKING	Neg	
9663	3/11/14	F	6.0	MURAMBA	Pos	
9664	3/11/14	M	0.8	MAGHOMA	Neg	
9665	3/11/14	M	6.0	NYAKABUGHA	Neg	
9666	3/11/14	F	1.5	BUGOYE	Neg	
9667	3/11/14	F	16.0	BUGOYE	Neg	
9668	3/12/14	F	16.0	MALIBA	Neg	
9669	3/12/14	M	46.0	NYABUSISI	Neg	
9670	3/12/14	M	1.3	RWAKING	Neg	
9671	3/12/14	M	4.0	RWAKING	Pos	
9672	3/12/14	F	21.0	BUHUNGA	Neg	
9673	3/12/14	M	21.0	KIKOKERA	Pos	
9674	3/12/14	F	13.0	BUGOYE	Neg	

9675	3/12/14	M	48.0	IZINGA	Neg
9676	3/12/14	F	1.8	IZINGA	Neg
9677	3/12/14	F	36.0	RWAKING	Neg
9678	3/12/14	F	0.5	RWAKING	Neg
9679	3/12/14	F	40.0	BULYANDEKE	Pos
9680	3/12/14	F	12.0	RWAKING	Pos
9681	3/12/14	M	75.0	KATOOKE	Pos
9682	3/12/14	F	4.0	MALIBA	Pos
9683	3/12/14	F	18.0	IZINGA	Pos
9684	3/12/14	F	14.0	IZINGA	Pos
9685	3/12/14	M	0.5	MAGHOMA	Pos
9686	3/12/14	F	4.0	BUNYANGONI	Neg
9687	3/12/14	M	2.0	BUGOYE	Neg
9688	3/12/14	F	0.3	MURAMBA	Neg
9689	3/12/14	F	60.0	BUGOYE	Pos
9690	3/12/14	M	15.0	IZINGA	Pos
9691	3/12/14	F	18.0	KANYAN	Pos
9692	3/12/14	M	1.5	MURAMBA	Neg
9693	3/12/14	M	0.5	MALIBA	Pos
9694	3/12/14	F	2.5	MURAMBA	Neg
9695	3/12/14	F	2.0	KATOOKE	Neg
9696	3/12/14	F	2.0	RWAKING	Neg
9697	3/12/14	F	22.0	KISOJO	Neg
9698	3/12/14	F	30.0	BUGOYE	Neg
9699	3/12/14	F	4.0	BUGOYE	Neg
9700	3/12/14	F	18.0	BUGOYE	Neg
9701	3/12/14	F	3.5	MURAMBA	Neg
9702	3/12/14	M	11.0	IZINGA	Pos
9703	3/12/14	F	70.0	MUBUKU	Neg
9704	3/12/14	F	70.0	KATUMBA	Neg
9705	3/12/14	F	10.0	BUGOYE	Neg
9706	3/12/14	F	10.0	BUGOYE	Pos
9707	3/12/14	F	42.0	BUGOYE	Neg
9708	3/12/14	F	4.3	NYAKABUGHA	Pos
9709	3/12/14	F	21.0	NYAKABUGHA	Pos
9710	3/12/14	F	4.0	MURAMBA	Pos
9711	3/12/14	M	4.0	RWAKING	Pos
9712	3/12/14	M	0.8	IZINGA	Neg
9713	3/12/14	F	20.0	BUGOYE	Pos
9714	3/12/14	F	40.0	NDUGHUTU	Pos
9715	3/12/14	M	0.3	IBANDA	Neg
9716	3/14/14	M	20.0	BUGOYE	Neg
9717	3/14/14	F	19.0	IBANDA	Neg

9718	3/14/14	F	28.0	BUGOYE	Neg	
9719	3/14/14	M	70.0	BUNYANGONI	Neg	
9720	3/14/14	F	4.0	BUGOYE	Neg	Neg
9721	3/14/14	F	12.0	NDUGHUTU	Neg	
9722	3/14/14	M	2.0	NDUGHUTU	Neg	
9723	3/14/14	F	9.0	NDUGHUTU	Neg	
9724	3/14/14	M	4.5	NDUGHUTU	Pos	
9725	3/14/14	F	26.0	KIHARARA	Pos	
9726	3/14/14	M	10.0	NDUGHUTU	Neg	
9727	3/14/14	F	60.0	KIBIRIZI	Neg	
9728	3/14/14	F	23.0	NDUGHUTU	Neg	
9729	3/14/14	F	19.0	MUHAMBO	Neg	
9730	3/14/14	F	36.0	MURAMBA	Neg	
9731	3/14/14	M	6.0	RWAKING	Pos	
9732	3/14/14	F	35.0	RWAKING	Neg	
9733	3/14/14	F	5.0	MALIBA	Pos	
9734	3/14/14	F	18.0	IZINGA	Neg	
9735	3/14/14	F	21.0	KAKINDO	Neg	
9736	3/14/14	F	6.0	KAKINDO	Pos	
9737	3/14/14	F	1.3	IZINGA	Neg	
9738	3/14/14	F	5.0	IZINGA	Neg	
9739	3/14/14	M	1.5	MALIBA	Neg	
9740	3/14/14	F	14.0	BUGOYE	Neg	
9741	3/17/14	F	1.5	KIKOKERA	Neg	
9742	3/17/14	M	19.0	MURAMBA	Pos	
9743	3/17/14	F	4.0	KISOJO	Neg	
9744	3/17/14	F	12.0	RWAKING	Neg	
9745	3/17/14	M	18.0	MURAMBA	Pos	
9746	3/17/14	F	16.0	BUZIBWERA	Pos	
9747	3/17/14	F	4.0	KATUMBA	Pos	
9748	3/17/14	F	56.0	BUGOYE	Neg	
9749	3/17/14	F	12.0	BUGOYE	Neg	
9750	3/17/14	F	22.0	IZINGA	Neg	
9751	3/17/14	M	3.0	KANYAN	Neg	
9752	3/17/14	F	4.0	KANYAN	Neg	
9753	3/17/14	F	5.5	KANYAN	Neg	
9754	3/17/14	F	23.0	KANYAN	Neg	
9755	3/17/14	F	1.5	BUGOYE	Neg	
9756	3/17/14	M	1.3	KANYAN	Neg	
9757	3/17/14	M	2.0	IZINGA	Pos	
9758	3/17/14	M	36.0	MURAMBA	Pos	
9759	3/17/14	M	0.8	KISANZI	Neg	
9760	3/17/14	F	12.0	BUNYANGONI	Neg	

9761	3/17/14	M	0.8	KIHINDI	Neg
9762	3/17/14	F	11.0	BUGOYE	Neg
9763	3/17/14	F	7.0	BUGOYE	Neg
9764	3/17/14	M	1.0	IHANI	Neg
9765	3/17/14	F	60.0	NDUGHUTU	Neg
9766	3/17/14	F	7.0	BULINDUGURU	Neg
9767	3/17/14	M	1.3	BUGOYE	Neg
9768	3/17/14	F	44.0	BUGOYE	Neg
9769	3/17/14	M	14.0	BUNYANGONI	Pos
9770	3/17/14	M	40.0	KIBOTA	Pos
9771	3/17/14	F	1.5	NDUGHUTU	Neg
9772	3/17/14	F	18.0	BUGOYE	Neg
9773	3/17/14	F	20.0	MOFO	Pos
9774	3/17/14	M	35.0	MALIBA	Neg
9775	3/17/14	M	33.0	BUNYANGONI	Neg
9776	3/17/14	M	35.0	MALIBA	Neg
9777	3/17/14	M	1.5	BWEYALE	Neg
9778	3/17/14	M	6.0	MURAMBA	Neg
9779	3/17/14	F	1.5	IZINGA	Neg
9780	3/17/14	F	10.0	KYARUGOMOKA	Neg
9781	3/17/14	F	32.0	MURAMBA	Pos
9782	3/17/14	M	1.0	IHANI	Neg
9783	3/17/14	F	0.3	NDUGHUTU	Neg
9784	3/17/14	M	5.0	IHANI	Neg
9785	3/17/14	F	17.0	BUGOYE	Pos
9786	3/18/14	F	52.0	KANYAN	Neg
9787	3/18/14	M	2.0	KANYAN	Neg
9788	3/18/14	F	7.0	NDUGHUTU	Neg
9789	3/18/14	M	74.0	BUNYANGONI	Neg
9790	3/18/14	F	34.0	NDUGHUTU	Pos
9791	3/18/14	F	1.3	MAGHOMA	Neg
9792	3/18/14	F	34.0	IBANDA	Neg
9793	3/18/14	M	9.0	KIBOTA	Neg
9794	3/18/14	F	25.0	NDUGHUTU	Neg
9795	3/18/14	F	27.0	BUNYANGONI	Neg
9796	3/18/14	F	54.0	BUNYANGONI	Neg
9797	3/19/14	F	35.0	IBANDA	Neg
9798	3/19/14	M	2.0	NDUGHUTU	Neg
9799	3/19/14	M	30.0	KIKOKERA	Neg
9800	3/19/14	F	10.0	BUNYANGONI	Neg
9801	3/19/14	F	28.0	IZINGA	Neg
9802	3/19/14	M	4.0	IZINGA	Neg
9803	3/19/14	F	4.0	IZINGA	Pos

9804	3/19/14	F	7.0	IZINGA	Neg	
9805	3/19/14	F	9.0	IZINGA	Pos	
9806	3/19/14	F	40.0	BUZIBWERA	Neg	
9807	3/19/14	F	90.0	KYANDALE	Neg	
9808	3/19/14	F	20.0	BUNYANGONI	Pos	
9809	3/19/14	M	14.0	KYARUGOMOKA	Pos	
9810	3/19/14	F	0.8	NDUGHUTU	Neg	
9811	3/19/14	F	26.0	RWAKING	Pos	
9812	3/19/14	F	7.0	KANYAN	Pos	
9813	3/19/14	F	60.0	KATUMBA	Pos	
9814	3/19/14	F	18.0	IZINGA	Pos	Pos
9815	3/19/14	M	15.0	MURAMBA	Pos	
9816	3/19/14	M	1.0	NYAMBUKU	Neg	Neg
9817	3/19/14	F	9.0	IBANDA	Pos	
9818	3/19/14	F	45.0	BUNYANGONI	Neg	
9819	3/19/14	F	10.0	BUNYANGONI	Neg	
9820	3/19/14	F	8.0	KANYAN	Pos	
9821	3/19/14	F	13.0	BUGOYE	Pos	
9822	3/19/14	M	1.0	MALIBA	Neg	
9823	3/19/14	M	3.0	KATUMBA	Neg	
9824	3/19/14	M	40.0	MURAMBA	Pos	
9825	3/19/14	F	19.0	KATUMBA	Neg	
9826	3/19/14	F	3.0	MUHAMBO	Neg	
9827	3/19/14	M	1.0	MUHAMBO	Neg	
9828	3/19/14	M	17.0	BUGOYE	Neg	
9829	3/19/14	M	39.0	MURAMBA	Neg	
9830	3/19/14	F	48.0	NYAKARUNGU	Pos	
9831	3/20/14	F	54.0	KANYAN	Neg	Neg
9832	3/20/14	F	10.0	IZINGA	Neg	
9833	3/20/14	F	12.0	BUGOYE	Neg	
9834	3/20/14	F	16.0	IZINGA	Neg	
9835	3/20/14	M	1.0	KANYAN	Neg	Neg
9836	3/20/14	M	30.0	BUZIBWERA	Neg	
9837	3/20/14	F	9.0	IHANI	Pos	
9838	3/20/14	M	2.8	RWAKING	Neg	
9839	3/20/14	F	2.5	RWAKING	Neg	
9840	3/20/14	F	19.0	IBANDA	Pos	
9841	3/20/14	F	2.5	MALIBA	Neg	
9842	3/20/14	F	40.0	KANYAN	Neg	
9843	3/20/14	M	0.8	NDUGHUTU	Neg	
9844	3/20/14	M	13.0	BUGOYE	Pos	
9845	3/20/14	F	0.8	BULINDUGURU	Neg	
9846	3/20/14	F	11.0	KANYAN	Pos	

9847	3/20/14	F	0.5	KABUYIRI	Pos	
9848	3/20/14	F	20.0	RWAKING	Neg	
9849	3/21/14	F	22.0	IZINGA	Pos	
9850	3/21/14	F	5.0	IZINGA	Neg	
9851	3/21/14	M	15.0	.	Pos	Pos
9852	3/21/14	F	20.0	IBANDA	Neg	
9853	3/21/14	F	54.0	KIBIRIZI	Neg	
9854	3/21/14	F	1.5	NDUGHUTU	Pos	
9855	3/21/14	F	11.0	MURAMBA	Pos	
9856	3/21/14	M	2.5	BUZIBWERA	Pos	
9857	3/21/14	F	8.0	KYARUGOMOKA	Pos	
9858	3/21/14	M	10.0	BUGOYE	Pos	
9859	3/21/14	F	21.0	BUNYANGONI	Neg	
9860	3/21/14	M	1.3	KAKINDO	Pos	
9861	3/21/14	M	1.3	MURAMBA	Neg	
9862	3/21/14	F	1.3	MUHAMBO	Neg	
9863	3/21/14	M	3.0	IZINGA	Pos	
9864	3/21/14	M	0.8	MURAMBA	Pos	
9865	3/21/14	F	3.5	IZINGA	Neg	
9866	3/21/14	F	0.5	IZINGA	Pos	
9867	3/21/14	F	22.0	BUGOYE	Neg	
9868	3/21/14	M	4.0	BUHUNGA	Neg	
9869	3/21/14	M	2.5	BUHUNGA	Neg	
9870	3/24/14	F	30.0	KANYAN	Neg	
9871	3/24/14	F	14.0	RWAKING	Neg	
9872	3/24/14	M	8.0	IBANDA	Neg	
9873	3/24/14	M	54.0	NYAKARUNGU	Neg	
9874	3/24/14	F	0.5	MURAMBA	Neg	
9875	3/24/14	F	12.0	BUGOYE	Pos	
9876	3/24/14	F	18.0	KANYAN	Neg	
9877	3/24/14	F	19.0	MALIBA	Neg	
9878	3/24/14	F	12.0	MALIBA	Neg	
9879	3/24/14	F	45.0	BUGOYE	Pos	
9880	3/24/14	F	43.0	MURAMBA	Pos	
9881	3/24/14	F	10.0	MURAMBA	Pos	
9882	3/24/14	F	9.0	MURAMBA	Pos	
9883	3/24/14	F	14.0	MURAMBA	Pos	
9884	3/24/14	M	13.0	MURAMBA	Pos	
9885	3/24/14	M	7.0	BUGOYE	Neg	
9886	3/24/14	F	2.0	KIKOKERA	Pos	Pos
9887	3/24/14	F	52.0	KIGORO	Neg	
9888	3/24/14	M	53.0	IBANDA	Neg	
9889	3/24/14	F	40.0	MURAMBA	Pos	

9890	3/24/14	F	19.0	MURAMBA	Neg	
9891	3/24/14	M	0.3	MURAMBA	Neg	
9892	3/24/14	F	5.0	MURAMBA	Neg	
9893	3/24/14	F	18.0	KIKOKERA	Neg	
9894	3/24/14	F	0.8	NYAKARUNGU	Neg	
9895	3/24/14	M	73.0	IZINGA	Pos	
9896	3/24/14	F	0.5	MURAMBA	Neg	
9897	3/24/14	F	11.0	BIKONE	Neg	
9898	3/24/14	F	12.0	IZINGA	Pos	
9899	3/24/14	F	64.0	BUGOYE	Neg	
9900	3/24/14	F	1.0	KIKOKERA	Neg	Neg
9901	3/24/14	F	37.0	KIKOKERA	Neg	
9902	3/25/14	F	2.0	BUGOYE	Neg	Neg
9903	3/25/14	F	50.0	BUGOYE	Pos	Pos
9904	3/25/14	M	47.0	BUGOYE	Neg	
9905	3/25/14	M	1.8	KISAMBA	Neg	
9906	3/25/14	M	3.5	MURAMBA	Pos	
9907	3/25/14	F	28.0	MURAMBA	Neg	
9908	3/25/14	M	10.0	KANYAN	Neg	
9909	3/25/14	F	22.0	BUNYANGONI	Neg	
9910	3/25/14	M	8.0	NDUGHUTU	Neg	
9911	3/25/14	F	0.8	NDUGHUTU	Neg	Neg
9912	3/25/14	F	29.0	MURAMBA	Pos	Pos
9913	3/25/14	F	77.0	BUGOYE	Neg	
9914	3/25/14	F	16.0	BUGOYE	Neg	
9915	3/25/14	M	11.0	BUGOYE	Neg	
9916	3/25/14	M	8.0	BUGOYE	Pos	
9917	3/25/14	M	2.0	KYANYA	Neg	
9918	3/25/14	F	8.0	BUGOYE	Pos	
9919	3/25/14	F	21.0	MURAMBA	Neg	
9920	3/25/14	M	3.0	KYANYA	Pos	
9921	3/25/14	F	26.0	KIKOKERA	Neg	
9922	3/25/14	F	26.0	MALIBA	Neg	Neg
9923	3/26/14	M	15.0	RUBONI	Neg	
9924	3/26/14	F	48.0	BUGOYE	Pos	
9925	3/26/14	M	18.0	BUGOYE	Neg	
9926	3/26/14	F	50.0	KANYAN	Neg	
9927	3/26/14	M	50.0	MUHAMBO	Neg	
9928	3/26/14	F	35.0	BUGOYE	Neg	
9929	3/26/14	M	2.0	IBANDA	Neg	
9930	3/26/14	M	13.0	RWAKING	Pos	
9931	3/26/14	M	10.0	KISOJO	Neg	
9932	3/26/14	M	14.0	IZINGA	Pos	

9933	3/26/14	F	30.0	KANYAN	Neg	
9934	3/26/14	M	13.0	IZINGA	Pos	
9935	3/26/14	M	3.0	IZINGA	Pos	Neg
9936	3/26/14	F	10.0	BUNYANGONI	Pos	
9937	3/26/14	F	13.0	KANYAN	Pos	
9938	3/26/14	F	0.5	MAGHOMA	Neg	
9939	3/26/14	F	15.0	MURAMBA	Pos	
9940	3/26/14	F	17.0	KAKINDO	Pos	
9941	3/26/14	M	12.0	IHANI	Pos	
9942	3/26/14	M	8.0	KYARUGOMOKA	Pos	
9943	3/26/14	F	3.0	NDUGHUTU	Neg	
9944	3/26/14	F	30.0	KYARUGOMOKA	Pos	
9945	3/26/14	F	7.0	IZINGA	Pos	
9946	3/26/14	F	25.0	KISOJO	Pos	
9947	3/26/14	M	80.0	BUGOYE	Neg	
9948	3/26/14	F	37.0	BUGOYE	Neg	
9949	3/26/14	F	6.3	BUNYANGONI	Pos	
9950	3/26/14	M	4.0	IZINGA	Neg	
9951	3/26/14	M	29.0	NDUGHUTU	Neg	
9952	3/26/14	M	39.0	RWAKING	Neg	
9953	3/26/14	F	25.0	IZINGA	Neg	
9954	3/26/14	F	43.0	KISOJO	Neg	
9955	3/26/14	M	0.5	KAKINDO	Neg	
9956	3/26/14	M	4.0	MALIBA	Pos	
9957	3/26/14	F	6.0	MALIBA	Pos	
9958	3/26/14	F	2.5	KATUMBA	Neg	
9959	3/27/14	M	24.0	KANYAN	Pos	
9960	3/27/14	F	24.0	NDUGHUTU	Neg	
9961	3/27/14	M	3.0	BUNYANGONI	Pos	
9962	3/27/14	F	49.0	NDUGHUTU	Neg	
9963	3/27/14	M	3.0	KANYAN	Pos	
9964	3/27/14	M	13.0	KYARUGOMOKA	Neg	
9965	3/27/14	F	19.0	MURAMBA	Neg	
9966	3/27/14	F	11.0	IZINGA	Pos	
9967	3/27/14	M	16.0	BUNYANGONI	Neg	
9968	3/27/14	F	2.5	BUZIBWERA	Pos	
9969	3/27/14	M	14.0	MAGHOMA	Neg	
9970	3/27/14	M	11.0	MURAMBA	Pos	
9971	3/27/14	F	25.0	KANYAN	Neg	
9972	3/27/14	F	17.0	MUBUKU	Pos	
9973	3/27/14	M	63.0	NDUGHUTU	Pos	
9974	3/27/14	F	35.0	MURAMBA	Pos	
9975	3/27/14	F	23.0	KIKOKERA	Pos	

9976	3/27/14	F	50.0	MURAMBA	Neg	
9977	3/27/14	F	4.0	RWAKING	Pos	
9978	3/27/14	M	4.3	BUNYANGONI	Pos	
9979	3/27/14	M	22.0	IZINGA	Neg	
9980	3/27/14	M	15.0	IZINGA	Pos	
9981	3/27/14	M	5.3	IZINGA	3	Pos
9982	3/27/14	F	61.0	MALIBA	Neg	
9983	3/27/14	F	7.0	BUGOYE	Pos	
9984	3/27/14	F	4.3	MURAMBA	Pos	
9985	3/27/14	M	1.3	IZINGA	Neg	
9986	3/27/14	F	25.0	KIRONGO	Pos	
9987	3/27/14	F	13.0	BUGOYE	Neg	
9988	3/27/14	F	10.0	MURAMBA	Neg	
9989	3/27/14	F	14.0	KANYAN	Neg	
9990	3/27/14	M	12.0	MURAMBA	Neg	
9991	3/27/14	F	0.8	MURAMBA	Neg	
9992	3/27/14	M	0.5	MURAMBA	Pos	
9993	3/27/14	F	8.0	MALIBA	Neg	
9994	3/27/14	M	17.0	MALIBA	Pos	
9995	3/27/14	F	25.0	MURAMBA	Neg	
9996	3/27/14	M	0.5	NDUGHUTU	Neg	Neg
9997	3/28/14	M	38.0	BUZIBWERA	Neg	
9998	3/28/14	M	10.0	KANYAN	Pos	
9999	3/28/14	F	43.0	KANYAN	Neg	
10000	3/28/14	F	20.0	MURAMBA	Neg	
10001	3/28/14	M	1.3	MURAMBA	Pos	
10002	3/28/14	M	0.8	MURAMBA	Neg	
10003	3/28/14	F	20.0	MURAMBA	Neg	
10004	3/28/14	F	37.0	RWAKING	Neg	
10005	3/28/14	M	8.0	MURAMBA	Neg	
10006	3/28/14	F	40.0	RWAKING	Neg	
10007	3/28/14	F	21.0	MURAMBA	Neg	
10008	3/28/14	F	19.0	MURAMBA	Pos	
10009	3/28/14	F	26.0	BUNYANGONI	Neg	
10010	3/28/14	F	7.0	MURAMBA	Pos	
10011	3/28/14	F	20.0	MURAMBA	Neg	
10012	3/28/14	F	5.0	MURAMBA	Pos	
10013	3/28/14	F	26.0	BUGOYE	Neg	
10014	3/28/14	F	4.0	MURAMBA	Pos	
10015	3/28/14	F	32.0	MURAMBA	Neg	
10016	3/31/14	M	2.0	BUGOYE	Neg	
10017	3/31/14	M	32.0	KANYAN	Neg	
10018	3/31/14	F	42.0	RWAKING	Neg	

10019	3/31/14	F	35.0	MURAMBA	Neg	
10020	3/31/14	M	6.5	KYARUGOMOKA	Pos	
10021	3/31/14	M	6.0	MURAMBA	Pos	
10022	3/31/14	M	10.0	MURAMBA	Pos	
10023	3/31/14	F	40.0	NDUGHUTU	Pos	
10024	3/31/14	F	8.0	KYANYA	Neg	
10025	3/31/14	M	10.0	IZINGA	Pos	
10026	3/31/14	M	12.0	IZINGA	Pos	
10027	3/31/14	F	22.0	BUZIBWERA	Neg	
10028	3/31/14	M	4.0	RWAKING	Pos	
10029	3/31/14	F	0.8	RWAKING	Neg	
10030	3/31/14	F	22.0	RWAKING	Neg	
10031	3/31/14	M	3.5	IZINGA	Neg	
10032	3/31/14	M	19.0	KANYAN	Neg	
10033	3/31/14	M	4.0	BUZIBWERA	Pos	
10034	3/31/14	F	2.0	MURAMBA	Pos	
10035	3/31/14	F	20.0	KANYAN	Neg	
10036	3/31/14	F	31.0	IZINGA	Pos	
10037	3/31/14	F	2.5	IZINGA	Pos	
10038	3/31/14	F	45.0	KYANYA	Neg	
10039	3/31/14	F	0.5	KIBOTA	Neg	
10040	3/31/14	M	16.0	KATOOKE	Neg	
10041	3/31/14	F	7.0	KISAMBA	Neg	
10042	3/31/14	M	23.0	BUNYANGONI	Neg	
10043	3/31/14	M	5.0	KISAMBA	Neg	
10044	3/31/14	M	1.3	NDUGHUTU	Neg	
10045	3/31/14	F	22.0	MURAMBA	Neg	
10046	3/31/14	F	0.8	IZINGA	Neg	
10047	3/31/14	M	15.0	IZINGA	Neg	
10048	3/31/14	F	27.0	IBANDA	Neg	
10049	3/31/14	M	5.0	KYARUGOMOKA	Pos	
10050	3/31/14	M	3.0	KYARUGOMOKA	Neg	
10051	3/31/14	F	17.0	MALIBA	Neg	
10052	3/31/14	M	2.5	KATUMBA	Neg	
10053	3/31/14	F	39.0	KAKINDO	Neg	
10054	3/31/14	M	53.0	MURAMBA	Pos	
10055	3/31/14	M	1.5	MURAMBA	Neg	
10056	3/31/14	F	1.3	IZINGA	Pos	
10057	3/31/14	M	3.0	IZINGA	Pos	
10058	3/31/14	M	4.5	IZINGA	Pos	
10059	3/31/14	M	12.0	BUNYANGONI	Neg	
10060	3/31/14	F	13.0	KATOOKE	Neg	Neg
10061	4/1/14	M	22.0	BUGOYE	Neg	

10062	4/1/14	F	44.0	BUGOYE	Neg
10063	4/1/14	F	7.0	KISANZI	Pos
10064	4/1/14	F	1.7	RWAKING	Neg
10065	4/1/14	M	6.0	RWAKING	Neg
10066	4/1/14	M	4.0	BUGOYE	Neg
10067	4/1/14	M	45.0	KANYAN	Neg
10068	4/1/14	M	4.5	NYABUSISI	Pos
10069	4/1/14	F	25.0	KIRULI	Neg
10070	4/1/14	M	2.0	MURAMBA	Pos
10071	4/1/14	F	26.0	NDUGHUTU	Neg
10072	4/1/14	M	7.0	BUGOYE	Pos
10073	4/1/14	M	20.0	BUGOYE	Pos
10074	4/1/14	F	10.0	MALIBA	Neg
10075	4/1/14	M	11.0	MALIBA	Pos
10076	4/1/14	F	50.0	MALIBA	Neg
10077	4/1/14	F	17.0	NDUGHUTU	Neg
10078	4/1/14	F	40.0	IZINGA	Neg
10079	4/1/14	M	27.0	KISANGA	Neg
10080	4/2/14	M	35.0	MALIBA	Neg
10081	4/2/14	F	10.0	BUGOYE	Pos
10082	4/2/14	F	0.7	MAGHOMA	Neg
10083	4/2/14	M	6.0	IZINGA	Neg
10084	4/2/14	F	27.0	BUGOYE	Neg
10085	4/2/14	M	2.0	IZINGA	Neg
10086	4/2/14	M	0.8	MURAMBA	Neg
10087	4/2/14	F	18.0	MURAMBA	Neg
10088	4/2/14	M	0.6	MURAMBA	Neg
10089	4/2/14	F	13.0	IZINGA	Neg
10090	4/2/14	F	20.0	MURAMBA	Neg
10091	4/2/14	F	60.0	NDUGHUTU	Neg
10092	4/2/14	F	0.2	MUHAMBO	Neg
10093	4/2/14	M	21.0	IBANDA	Pos
10094	4/2/14	M	39.0	BUGOYE	Neg
10095	4/2/14	M	17.0	BUGOYE	Neg
10096	4/2/14	F	35.0	IZINGA	Pos
10097	4/2/14	F	0.6	KYANYA	Neg
10098	4/2/14	F	3.1	BUGOYE	Neg
10099	4/2/14	F	3.5	BUGOYE	Neg
10100	4/2/14	F	23.0	BUGOYE	Neg
10101	4/2/14	F	34.0	MURAMBA	Neg
10102	4/2/14	F	4.0	MURAMBA	Pos
10103	4/2/14	M	43.0	BUGOYE	Neg
10104	4/2/14	F	1.3	BUNYANGONI	Neg

10105	4/2/14	F	12.0	KISOJO	Neg	
10106	4/2/14	M	57.0	KANYAN	Pos	
10107	4/2/14	F	32.0	KYANYA	Neg	
10108	4/2/14	F	50.0	BUGOYE	Neg	
10109	4/2/14	M	3.0	BUGOYE	Neg	
10110	4/2/14	F	8.0	IZINGA	Pos	
10111	4/2/14	M	2.5	RWAKING	Neg	
10112	4/2/14	F	13.0	RWAKING	Pos	
10113	4/2/14	F	40.0	BUGOYE	Neg	
10114	4/2/14	F	0.5	IZINGA	Pos	
10115	4/2/14	F	44.0	KAKINDO	Neg	
10116	4/2/14	M	13.0	MURAMBA	Neg	
10117	4/2/14	F	7.0	BULYANDEKE	Neg	
10118	4/2/14	M	4.0	BUGOYE	Pos	
10119	4/3/14	M	27.0	BUGOYE	Neg	
10120	4/3/14	F	5.0	IBANDA	Pos	
10121	4/3/14	F	51.0	BUGOYE	Pos	
10122	4/3/14	M	7.0	KAKINDO	Pos	
10123	4/3/14	F	32.0	BUGOYE	Neg	
10124	4/3/14	M	2.3	NDUGHUTU	Neg	
10125	4/3/14	M	16.0	RWAKING	Pos	
10126	4/3/14	F	9.0	BUGOYE	Pos	
10127	4/3/14	F	32.0	BUGOYE	Neg	
10128	4/3/14	F	6.0	MURAMBA	Neg	
10129	4/3/14	F	4.0	MURAMBA	Neg	
10130	4/3/14	F	80.0	KANYAN	Neg	
10131	4/3/14	M	23.0	KISANGA	Pos	
10132	4/3/14	F	30.0	MURAMBA	Pos	
10133	4/3/14	F	4.0	BUGOYE	Pos	
10134	4/3/14	F	23.0	KISANGA	Pos	
10135	4/3/14	M	18.0	KIKONE	Neg	
10136	4/3/14	M	2.0	BUGOYE	Pos	
10137	4/3/14	F	42.0	RWAKING	Neg	
10138	4/3/14	F	2.3	RWAKING	Neg	
10139	4/3/14	F	2.5	IBANDA	Neg	
10140	4/3/14	F	3.0	KIKOKERA	Neg	
10141	4/3/14	F	10.0	KANYAN	Pos	
10142	4/3/14	M	45.0	BIKONE	Neg	
10143	4/3/14	F	32.0	BUGOYE	Neg	
10144	4/3/14	M	5.0	BUGOYE	Neg	
10145	4/3/14	M	2.0	MURAMBA	Neg	Neg
10146	4/3/14	M	1.0	MURAMBA	Pos	
10147	4/3/14	F	14.0	MURAMBA	Neg	

10148	4/3/14	F	42.0	BUGOYE	Pos	
10149	4/3/14	M	41.0	IZINGA	Pos	
10150	4/3/14	F	18.0	BUGOYE	Neg	
10151	4/3/14	M	28.0	BUGOYE	Neg	
10152	4/3/14	M	8.0	IZINGA	Pos	
10153	4/3/14	M	1.0	NDUGHUTU	Neg	
10154	4/3/14	F	30.0	KIRONGO	Neg	
10155	4/3/14	M	30.0	BUGOYE	Pos	
10156	4/3/14	M	40.0	BUGOYE	Neg	
10157	4/3/14	M	0.3	BUGOYE	Neg	
10158	4/4/14	M	40.0	MURAMBA	Pos	
10159	4/4/14	M	17.0	IZINGA	Neg	
10160	4/4/14	M	31.0	KAKINDO	Neg	
10161	4/4/14	F	25.0	BUGOYE	Neg	
10162	4/4/14	F	8.5	KIBIRIZI	Neg	
10163	4/4/14	F	50.0	BIKONE	Neg	
10164	4/4/14	F	3.0	BUZIBWERA	Pos	
10165	4/4/14	F	3.5	KYARUGOMOKA	Pos	
10166	4/4/14	F	2.0	KAKINDO	Neg	
10167	4/4/14	F	8.0	KAKINDO	Pos	
10168	4/4/14	F	17.0	NDUGHUTU	Neg	
10169	4/4/14	M	13.0	KANYAN	Neg	
10170	4/4/14	M	14.0	BUGOYE	Neg	
10171	4/4/14	F	30.0	BUNYANGONI	Neg	
10172	4/4/14	M	7.0	KANYAN	Neg	
10173	4/4/14	F	3.0	KYARUGOMOKA	Pos	
10174	4/4/14	M	12.0	MALIBA	Neg	
10175	4/4/14	F	0.9	KIKOKERA	Neg	
10176	4/4/14	M	0.5	MALIBA	Neg	
10177	4/4/14	F	22.0	IZINGA	Pos	
10178	4/4/14	F	2.0	MAGHOMA	Neg	
10179	4/4/14	M	78.0	MURAMBA	Neg	
10180	4/4/14	F	44.0	IZINGA	Neg	
10181	4/4/14	M	37.0	KYANYA	Neg	
10182	4/4/14	F	7.0	KYANYA	Pos	
10183	4/4/14	F	1.5	KIBIRIZI	Neg	
10184	4/4/14	F	16.0	MURAMBA	Pos	
10185	4/4/14	M	3.5	MURAMBA	Pos	
10186	4/4/14	F	2.0	MUKATI	Neg	
10187	4/4/14	F	23.0	BUNYANGONI	Neg	
10188	4/4/14	F	23.0	KIBIRIZI	Neg	
10189	4/4/14	M	1.3	KIKOKERA	Pos	Pos
10190	4/7/14	F	47.0	MURAMBA	Neg	

10191	4/7/14	F	40.0	MURAMBA	Pos
10192	4/7/14	M	13.0	IZINGA	Neg
10193	4/7/14	F	35.0	BUGOYE	Pos
10194	4/7/14	F	7.0	IZINGA	Neg
10195	4/7/14	F	10.0	RWAKING	Pos
10196	4/7/14	F	4.0	RWAKING	Pos
10197	4/7/14	F	2.0	MURAMBA	Pos
10198	4/7/14	F	29.0	BIKONE	Neg
10199	4/7/14	F	22.0	BUGOYE	Pos
10200	4/7/14	F	2.0	MURAMBA	Neg
10201	4/7/14	F	21.0	KIRONGO	Neg
10202	4/7/14	F	20.0	RWAKING	Pos
10203	4/7/14	M	13.0	IZINGA	Pos
10204	4/7/14	M	0.3	MUHAMBO	Neg
10205	4/7/14	F	12.0	BUGOYE	Neg
10206	4/7/14	M	1.5	MUBUKU	Pos
10207	4/7/14	F	22.0	KANYAN	Neg
10208	4/7/14	F	7.0	BUGOYE	Neg
10209	4/7/14	M	2.0	BUGOYE	Neg
10210	4/7/14	F	3.0	MUHAMBO	Pos
10211	4/7/14	F	50.0	RWAKING	Pos
10212	4/7/14	F	0.8	KANYAN	Pos
10213	4/7/14	F	5.0	BUGOYE	Neg
10214	4/7/14	F	1.3	KATUMBA	Neg
10215	4/7/14	F	35.0	NDUGHUTU	Neg
10216	4/7/14	F	0.6	MURAMBA	Pos
10217	4/7/14	M	3.0	KATUMBA	Pos
10218	4/7/14	F	2.7	MUHAMBO	Neg
10219	4/7/14	F	11.0	RWAKING	Pos
10220	4/7/14	F	37.0	BUHUNGA	Neg
10221	4/7/14	F	16.0	BUGOYE	Pos
10222	4/7/14	F	14.0	BUGOYE	Neg
10223	4/7/14	F	17.0	MURAMBA	Neg
10224	4/7/14	M	.	MURAMBA	Pos
10225	4/7/14	M	35.0	IZINGA	Pos
10226	4/7/14	F	60.0	IZINGA	Pos
10227	4/7/14	M	2.0	MURAMBA	Neg
10228	4/7/14	F	2.0	IBANDA	Neg
10229	4/7/14	F	0.5	IZINGA	Pos
10230	4/7/14	F	24.0	IZINGA	Neg
10231	4/7/14	M	6.0	IZINGA	Pos
10232	4/7/14	F	3.0	IZINGA	Pos
10233	4/7/14	M	0.7	IBANDA	Neg

10234	4/7/14	M	10.0	MURAMBA	Neg
10235	4/7/14	M	1.0	MURAMBA	Neg
10236	4/7/14	F	21.0	KYANYA	Neg
10237	4/7/14	F	3.0	KYANYA	Pos
10238	4/7/14	F	35.0	IBANDA	Neg
10239	4/7/14	M	0.3	BUGOYE	Neg
10240	4/7/14	M	20.0	BUGOYE	Neg
10241	4/7/14	F	30.0	KYANYA	Pos
10242	4/8/14	F	14.0	BUZIBWERA	Neg
10243	4/8/14	M	65.0	BUGOYE	Pos
10244	4/8/14	M	30.0	MUHAMBO	Neg
10245	4/8/14	M	12.0	BUGOYE	Neg
10246	4/8/14	M	15.0	BUGOYE	Pos
10247	4/8/14	M	9.0	IZINGA	Pos
10248	4/8/14	M	0.8	BUGOYE	Pos
10249	4/8/14	F	34.0	KIBIRIZI	Neg
10250	4/8/14	M	1.1	RWAKING	Neg
10251	4/8/14	M	19.0	MURAMBA	Pos
10252	4/8/14	F	0.7	RWAKING	Neg
10253	4/8/14	F	0.7	RWAKING	Neg
10254	4/8/14	F	1.5	BUGOYE	Neg
10255	4/8/14	M	30.0	BUHUNGA	Neg
10256	4/8/14	M	33.0	BUGOYE	Neg
10257	4/8/14	F	25.0	RWAKING	Neg
10258	4/8/14	F	5.0	RWAKING	Neg
10259	4/8/14	M	3.0	BUGOYE	Neg
10260	4/8/14	F	5.0	BUGOYE	Pos
10261	4/8/14	F	9.0	MURAMBA	Pos
10262	4/8/14	F	21.0	BUGOYE	Pos
10263	4/8/14	F	26.0	BUGOYE	Pos
10264	4/8/14	F	6.0	MURAMBA	Pos
10265	4/8/14	F	12.0	MURAMBA	Neg
10266	4/8/14	F	18.0	IBANDA	Neg
10267	4/9/14	F	42.0	NYAKARUNGU	Neg
10268	4/9/14	F	17.0	NYAKARUNGU	Neg
10269	4/9/14	F	6.0	BUGOYE	Pos
10270	4/9/14	F	18.0	IZINGA	Pos
10271	4/9/14	F	25.0	KIBIRIZI	Neg
10272	4/9/14	F	23.0	MURAMBA	Pos
10273	4/9/14	M	8.5	KATUMBA	Neg
10274	4/9/14	F	21.0	KATUMBA	Neg
10275	4/9/14	M	0.8	NDUGHUTU	Neg
10276	4/9/14	F	5.0	MUHAMBO	Neg

10277	4/9/14	F	20.0	MURAMBA	Neg	
10278	4/9/14	M	30.0	IZINGA	Neg	
10279	4/9/14	F	23.0	MURAMBA	Neg	
10280	4/9/14	F	24.0	BUGOYE	Pos	
10281	4/9/14	F	20.0	MUHAMBO	Neg	
10282	4/9/14	M	0.4	MUHAMBO	Neg	
10283	4/9/14	M	1.0	NDUGHUTU	Neg	
10284	4/9/14	F	36.0	BUGOYE	Neg	
10285	4/9/14	F	62.0	KATUMBA	Neg	
10286	4/9/14	M	16.0	MURAMBA	Pos	
10287	4/9/14	F	38.0	MURAMBA	Neg	
10288	4/9/14	F	14.0	BUGOYE	Pos	
10289	4/9/14	F	20.0	BUGOYE	Neg	
10290	4/9/14	F	0.3	KYARUGOMOKA	Pos	
10291	4/9/14	F	14.0	KYARUGOMOKA	Neg	
10292	4/9/14	F	45.0	MURAMBA	Neg	
10293	4/9/14	F	18.0	BUGOYE	Neg	
10294	4/9/14	M	11.0	KANYAN	Neg	
10295	4/9/14	M	20.0	BUGOYE	Neg	
10296	4/9/14	F	19.0	MURAMBA	Pos	
10297	4/9/14	F	18.0	BUNSONGORA	Neg	
10298	4/9/14	F	19.0	BUGOYE	Neg	
10299	4/9/14	M	7.0	BUGOYE	Neg	
10300	4/9/14	F	12.0	IZINGA	Pos	
10301	4/9/14	M	5.0	MURAMBA	Neg	
10302	4/9/14	F	13.0	KAKINDO	Neg	
10303	4/9/14	M	10.0	MALIBA	Pos	
10304	4/9/14	F	23.0	KAKINDO	Neg	
10305	4/9/14	M	9.0	MALIBA	Neg	
10306	4/9/14	F	28.0	RWAKING	Neg	
10307	4/10/14	F	4.5	MURAMBA	Pos	
10308	4/10/14	F	0.8	KANYAN	Neg	Neg
10309	4/10/14	F	14.0	IHANI	Pos	
10310	4/10/14	F	47.0	RWAKING	Neg	
10311	4/10/14	F	48.0	IZINGA	Neg	
10312	4/10/14	M	16.0	IHANI	Neg	
10313	4/10/14	F	18.0	KIHINDI	Pos	
10314	4/10/14	M	2.1	MURAMBA	Neg	
10315	4/10/14	F	26.0	MURAMBA	Neg	
10316	4/10/14	M	17.0	BULYANDEKE	Pos	
10317	4/10/14	F	22.0	KYARUGOMOKA	Pos	
10318	4/10/14	M	8.0	MURAMBA	Pos	
10319	4/10/14	F	3.5	BIKONE	Neg	

10320	4/10/14	F	11.0	MALIBA	Neg	
10321	4/10/14	F	17.0	MURAMBA	Pos	
10322	4/10/14	F	2.0	IZINGA	Pos	
10323	4/10/14	F	6.0	BUZIBWERA	Pos	
10324	4/10/14	F	6.0	BUZIBWERA	Pos	
10325	4/10/14	F	40.0	BUZIBWERA	Neg	
10326	4/10/14	M	30.0	KYARUGOMOKA	Neg	
10327	4/10/14	F	40.0	KYANYA	Neg	
10328	4/10/14	F	25.0	NYAKARUNGU	Neg	
10329	4/10/14	F	45.0	IHANI	Neg	
10330	4/10/14	M	0.3	KYARUGOMOKA	Neg	
10331	4/10/14	M	5.0	KYARUGOMOKA	Pos	
10332	4/10/14	F	16.0	MURAMBA	Pos	
10333	4/10/14	M	58.0	MUHAMBO	Neg	
10334	4/10/14	F	17.0	MUHAMBO	Neg	
10335	4/10/14	F	18.0	KANYAN	Neg	
10336	4/10/14	F	15.0	BUZIBWERA	Pos	
10337	4/10/14	F	40.0	IBANDA	Neg	
10338	4/10/14	F	15.0	BUGOYE	Pos	
10339	4/10/14	F	39.0	KANYAN	Neg	
10340	4/10/14	F	23.0	BUNYANGONI	Neg	
10341	4/10/14	M	1.5	IBANDA	Neg	
10342	4/10/14	F	6.0	MURAMBA	Pos	
10343	4/10/14	M	17.0	KANYAN	Pos	
10344	4/10/14	F	47.0	MURAMBA	Neg	
10345	4/10/14	F	23.0	BUZIBWERA	Neg	
10346	4/15/14	M	2.5	BUNYANGONI	Neg	
10347	4/15/14	M	20.0	IZINGA	Pos	
10348	4/15/14	F	22.0	MALIBA	Neg	
10349	4/15/14	F	30.0	KYARUGOMOKA	Neg	
10350	4/15/14	M	4.5	BUGOYE		Neg
10351	4/15/14	M	0.2	KIRONGO	Neg	
10352	4/15/14	F	0.7	KATOOKE	Neg	
10353	4/15/14	F	0.4	NDUGHUTU	Neg	
10354	4/15/14	M	0.6	MURAMBA	Pos	
10355	4/16/14	M	2.0	RWAKING	Neg	
10356	4/16/14	F	28.0	KANYAN	Neg	
10357	4/16/14	M	2.0	KATUMBA	Neg	
10358	4/16/14	M	4.3	BUNYANGONI		Pos
10359	4/16/14	M	18.0	BUGOYE	Neg	
10360	4/16/14	F	1.7	KINYEU	Neg	
10361	4/16/14	M	3.0	KIKOKERA	Neg	
10362	4/16/14	F	23.0	MURAMBA	Neg	

10363	4/16/14	F	26.0	IBANDA	Neg	
10364	4/16/14	M	2.0	BUNYANGONI	Neg	
10365	4/16/14	M	0.0	IZINGA	Neg	
10366	4/16/14	F	10.0	MURAMBA	Neg	
10367	4/16/14	F	0.8	MURAMBA	Neg	
10368	4/16/14	F	3.0	KIKOKERA	Neg	
10369	4/16/14	M	1.3	BUGOYE	Neg	
10370	4/16/14	M	6.0	MURAMBA		Neg
10371	4/16/14	F	5.5	IZINGA	Neg	
10372	4/16/14	M	20.0	KYANYA	Neg	
10373	4/16/14	F	20.0	KIHINDI	Neg	
10374	4/16/14	F	10.0	IBANDA	Pos	
10375	4/16/14	M	4.5	IZINGA	Neg	
10376	4/16/14	M	38.0	IHANI	Pos	
10377	4/16/14	F	12.0	KANYAN	Neg	
10378	4/16/14	F	5.0	BUNYANGONI	Neg	
10379	4/16/14	M	12.0	MURAMBA	Pos	
10380	4/16/14	M	50.0	BUGOYE	Neg	
10381	4/16/14	F	2.0	KYANYA	Neg	
10382	4/16/14	F	32.0	NDUGHUTU	Neg	
10383	4/16/14	F	31.0	BUNYANGONI	Neg	
10384	4/16/14	F	7.0	BUGOYE	Pos	
10385	4/16/14	F	23.0	BUGOYE	Neg	
10386	4/16/14	F	20.0	KYANYA	Neg	
10387	4/16/14	M	4.5	KISANZI	Pos	
10388	4/16/14	F	2.5	MURAMBA	Neg	
10389	4/16/14	F	27.0	NDUGHUTU	Neg	
10390	4/16/14	F	5.0	MUHAMBO	Neg	
10391	4/16/14	F	32.0	MUHAMBO	Pos	
10392	4/16/14	F	20.0	NDUGHUTU	Neg	
10393	4/16/14	F	5.0	NDUGHUTU	Neg	
10394	4/16/14	F	45.0	MURAMBA	Neg	
10395	4/16/14	F	1.5	KIBIRIZI	Neg	
10396	4/16/14	F	18.0	MUBUKU	Pos	
10397	4/16/14	M	4.0	KIBIRIZI	Neg	
10398	4/16/14	F	15.0	NDUGHUTU	Neg	
10399	4/17/14	M	5.0	RWAKING	Neg	
10400	4/17/14	M	16.0	MALIBA	Neg	
10401	4/17/14	M	16.0	IBANDA	Pos	
10402	4/17/14	F	40.0	KIKOKERA	Pos	
10403	4/17/14	F	6.5	KYARUGOMOKA	Neg	
10404	4/17/14	F	38.0	IBANDA	Pos	
10405	4/17/14	M	26.0	IZINGA	Pos	

10406	4/17/14	F	13.0	MUHAMBO	Neg
10407	4/17/14	M	2.0	MUHAMBO	Neg
10408	4/17/14	F	3.0	KIHINDI	Neg
10409	4/17/14	M	27.0	KIHINDI	Neg
10410	4/17/14	M	26.0	NDUGHUTU	Neg
10411	4/17/14	M	48.0	MURAMBA	Neg
10412	4/17/14	F	1.3	IZINGA	Pos
10413	4/17/14	F	8.0	IZINGA	Pos
10414	4/17/14	F	29.0	KYARUGOMOKA	Pos
10415	4/17/14	M	4.0	KYARUGOMOKA	Pos
10416	4/17/14	M	4.0	KIRONGO	Neg
10417	4/17/14	M	2.0	BUGOYE	Neg
10418	4/17/14	F	0.4	KYARUGOMOKA	Pos
10419	4/17/14	F	18.0	IBANDA	Pos
10420	4/17/14	M	0.8	IZINGA	Pos
10421	4/17/14	M	41.0	IZINGA	Neg
10422	4/17/14	F	1.7	KATOOKE	Pos
10423	4/17/14	M	2.0	KISANZI	Pos
10424	4/22/14	F	40.0	IBANDA	Pos
10425	4/22/14	M	1.7	KANYAN	Neg
10426	4/22/14	F	65.0	BUNYANGONI	Neg
10427	4/22/14	F	12.0	MURAMBA	Pos
10428	4/22/14	F	0.2	KAKINDO	Pos
10429	4/22/14	F	2.0	KANYAN	Pos
10430	4/22/14	M	11.0	KANYAN	Neg
10431	4/22/14	F	35.0	BUNYANGONI	Neg
10432	4/22/14	F	8.0	KAKINDO	Pos
10433	4/22/14	F	26.0	MUHAMBO	Pos
10434	4/22/14	M	10.0	IZINGA	Pos
10435	4/22/14	F	20.0	MURAMBA	Neg
10436	4/22/14	F	7.0	BUNYANGONI	Pos
10437	4/22/14	F	40.0	IZINGA	Neg
10438	4/22/14	F	23.0	KIRONGO	Neg
10439	4/22/14	F	10.0	KYARUGOMOKA	Neg
10440	4/22/14	M	1.5	BUHUNGA	Neg
10441	4/22/14	F	23.0	BUZIBWERA	Pos
10442	4/22/14	F	3.0	BUZIBWERA	Pos
10443	4/22/14	M	50.0	MURAMBA	Neg
10444	4/22/14	M	5.0	MURAMBA	Neg
10445	4/22/14	F	40.0	NDUGHUTU	Neg
10446	4/22/14	F	1.5	KYANYA	Neg
10447	4/22/14	F	24.0	KYANYA	Neg
10448	4/22/14	F	31.0	KYANYA	Neg

10449	4/22/14	F	14.0	KYANYA	Neg	
10450	4/22/14	F	12.0	BUGOYE	Neg	
10451	4/22/14	M	8.0	NYAKALENGIJO	Pos	
10452	4/22/14	M	0.7	KATUMBA	Neg	
10453	4/22/14	M	7.0	BUGOYE	Pos	
10454	4/22/14	M	12.0	BUNYANGONI	Neg	
10455	4/22/14	M	1.7	KATUMBA	Neg	
10456	4/22/14	F	0.8	KATUMBA	Neg	
10457	4/22/14	F	45.0	NYANGONGE	Neg	
10458	4/22/14	F	37.0	IZINGA	Neg	
10459	4/22/14	M	5.3	RWAKING	Neg	
10460	4/22/14	M	5.0	BUNYANGONI	Neg	
10461	4/22/14	F	38.0	BUNYANGONI	Neg	
10462	4/22/14	M	25.0	MUHAMBO	Neg	
10463	4/22/14	F	40.0	BUNYANGONI	Neg	
10464	4/22/14	F	6.0	BUNYANGONI	Neg	
10465	4/22/14	F	15.0	MALIBA	Neg	
10466	4/22/14	M	38.0	ISULE	Neg	
10467	4/22/14	F	30.0	MURAMBA	Neg	
10468	4/22/14	M	0.0	IZINGA	Neg	
10469	4/22/14	F	20.0	KYARUGOMOKA	Pos	
10470	4/22/14	F	10.0	BUNYANGONI	Neg	
10471	4/22/14	M	16.0	MURAMBA	Pos	
10472	4/22/14	F	10.0	BUGOYE	Neg	
10473	4/22/14	F	5.0	BIKONE	Pos	
10474	4/22/14	F	2.4	BUGOYE	Neg	
10475	4/22/14	F	29.0	BUGOYE	Neg	
10476	4/22/14	M	3.1	KIKOKERA	Pos	
10477	4/22/14	F	1.3	RWAKING	Pos	
10478	4/22/14	F	35.0	RWAKING	Neg	
10479	4/23/14	M	69.0	MURAMBA		Neg
10480	4/23/14	M	30.0	RWAKING	Neg	Neg
10481	4/23/14	F	22.0	BUNYANGONI	Neg	
10482	4/23/14	F	19.0	MAGHOMA	Neg	
10483	4/23/14	F	40.0	IZINGA	Neg	
10484	4/23/14	F	24.0	IHANI	Pos	
10485	4/23/14	F	32.0	BUGOYE	Neg	
10486	4/23/14	F	17.0	BUGOYE	Neg	
10487	4/23/14	M	0.2	KINYANGONGE	Neg	
10488	4/23/14	F	0.4	BUNYANGONI	Neg	
10489	4/23/14	F	1.3	RWAKING	Pos	
10490	4/23/14	M	1.7	KATUMBA	Neg	
10491	4/23/14	M	2.3	BULINDUGURU	Neg	

10492	4/23/14	F	17.0	KANYAN	Neg	
10493	4/23/14	M	0.5	BUNYANGONI	Neg	
10494	4/23/14	F	6.0	MALIBA	Pos	
10495	4/23/14	F	5.5	MALIBA	Pos	
10496	4/23/14	M	40.0	BUHUNGA	Neg	
10497	4/23/14	M	2.9	RWAKING	Neg	
10498	4/23/14	M	0.5	RWAKING	Neg	
10499	4/23/14	F	4.0	BUNYANGONI	Pos	
10500	4/23/14	F	4.0	KINYANGONGE	Neg	
10501	4/23/14	F	1.4	KINYANGONGE	Neg	
10502	4/23/14	F	14.0	KATOOKE	Neg	
10503	4/23/14	F	13.0	KINYANGONGE	Pos	Pos
10504	4/23/14	M	10.0	RUBONI	Neg	
10505	4/23/14	M	26.0	IBANDA	Neg	
10506	4/23/14	F	45.0	MALIBA	Neg	
10507	4/23/14	M	48.0	ISULE	Neg	
10508	4/23/14	F	55.0	MUBUKU	Neg	
10509	4/23/14	M	53.0	IBANDA	Neg	
10510	4/24/14	F	0.4	BUZIBWERA	Pos	
10511	4/24/14	F	55.0	MALIBA	Pos	
10512	4/24/14	M	68.0	RWAKING	Neg	
10513	4/24/14	M	5.0	NDUGHUTU	Pos	
10514	4/24/14	M	67.0	IZINGA	Neg	
10515	4/24/14	F	36.0	KISENDERA	Pos	
10516	4/24/14	M	8.0	RWAKING	Neg	
10517	4/24/14	F	42.0	BUGOYE	Neg	
10518	4/24/14	M	2.0	IZINGA	Pos	
10519	4/24/14	F	0.8	BIKONE	Neg	
10520	4/24/14	F	3.9	IZINGA	Pos	
10521	4/24/14	F	0.5	IZINGA	Pos	
10522	4/24/14	F	25.0	MUHAMBO	Neg	
10523	4/24/14	F	1.5	BUGOYE	Pos	
10524	4/24/14	F	70.0	KATOOKE	Neg	
10525	4/24/14	F	18.0	BUGOYE	Neg	
10526	4/24/14	F	7.5	BUGOYE	Pos	
10527	4/24/14	M	8.0	BUGOYE	Pos	
10528	4/25/14	F	1.0	MURAMBA	Pos	
10529	4/25/14	F	1.3	MALIBA	Neg	
10530	4/25/14	M	56.0	MUHAMBO	Neg	
10531	4/25/14	F	22.0	IZINGA	Neg	
10532	4/25/14	F	15.0	BIKONE	Pos	
10533	4/25/14	M	2.5	KIBIRIZI	Neg	
10534	4/25/14	F	60.0	MURAMBA	Neg	

10535	4/25/14	F	70.0	BUGOYE	Neg	
10536	4/25/14	F	70.0	NDUGHUTU	Neg	
10537	4/25/14	F	70.0	BUGOYE	Neg	
10538	4/25/14	F	42.0	BUZIBWERA	Neg	
10539	4/25/14	F	1.3	BUGOYE	Neg	
10540	4/25/14	M	0.4	MALIBA	Neg	
10541	4/25/14	F	40.0	BUHUNGA	Neg	
10542	4/25/14	M	11.0	BUHUNGA	Pos	
10543	4/25/14	F	30.0	MUHAMBO	Pos	
10544	4/25/14	F	22.0	KANYAN	Neg	
10545	4/25/14	M	40.0	ISULE	Neg	
10546	4/25/14	M	13.0	MURAMBA	Pos	
10547	4/25/14	F	17.0	IZINGA	Neg	
10548	4/25/14	F	0.1	BUGOYE	Neg	
10549	4/25/14	F	30.0	KIKOKERA	Neg	
10550	4/25/14	F	20.0	BUGOYE	Neg	
10551	4/25/14	M	25.0	BUGOYE	Neg	
10552	4/28/14	F	32.0	BUGOYE	Neg	
10553	4/28/14	F	48.0	MURAMBA	Neg	
10554	4/28/14	M	14.0	KANYAN	Pos	
10555	4/28/14	M	67.0	IZINGA	Pos	
10556	4/28/14	M	2.5	RWAKING	Neg	
10557	4/28/14	M	0.8	RWAKING	Neg	
10558	4/28/14	F	30.0	BUGOYE	Neg	
10559	4/28/14	M	1.9	BIKONE	Neg	Neg
10560	4/28/14	F	23.0	MURAMBA	Pos	
10561	4/28/14	M	54.0	IBANDA	Neg	
10562	4/28/14	M	13.0	NDUGHUTU	Neg	
10563	4/28/14	M	4.0	IZINGA	Pos	
10564	4/28/14	M	24.0	IZINGA	Neg	
10565	4/28/14	F	18.0	IZINGA	Neg	
10566	4/28/14	F	1.7	MUHAMBO	Neg	
10567	4/28/14	F	3.8	MALIBA	Pos	
10568	4/28/14	F	48.0	MURAMBA	Neg	
10569	4/28/14	F	10.0	IBANDA	Neg	
10570	4/28/14	F	8.0	IZINGA	Neg	
10571	4/28/14	F	40.0	IZINGA	Pos	
10572	4/28/14	F	32.0	MUHAMBO	Neg	
10573	4/28/14	F	0.5	BUGOYE	Neg	
10574	4/28/14	F	10.0	KAKINDO	Pos	
10575	4/28/14	M	27.0	IBANDA	Pos	
10576	4/28/14	M	14.0	MURAMBA	Pos	
10577	4/28/14	F	1.5	RWAKING	Neg	

10578	4/28/14	F	3.0	RWAKING	Pos	
10579	4/28/14	M	2.3	NDUGHUTU	Neg	
10580	4/28/14	M	1.2	MUHAMBO	Neg	
10581	4/28/14	M	0.3	MURAMBA	Neg	
10582	4/28/14	M	0.2	ISULE	Neg	
10583	4/28/14	F	11.0	ISULE	Pos	
10584	4/28/14	F	15.0	KIKOKERA	Neg	
10585	4/29/14	M	13.0	MURAMBA	Pos	
10586	4/29/14	M	65.0	BUGOYE	Neg	
10587	4/29/14	F	32.0	RWAKING	Pos	
10588	4/29/14	F	60.0	BUGOYE	Neg	
10589	4/29/14	M	25.0	MURAMBA	Pos	
10590	4/29/14	F	4.0	MALIBA	Neg	
10591	4/29/14	F	3.5	IZINGA	Neg	
10592	4/29/14	F	58.0	KANYAN	Neg	
10593	4/29/14	M	32.0	IBANDA	Neg	
10594	4/29/14	M	21.0	BUGOYE	Neg	
10595	4/29/14	F	7.0	MUHAMBO	Neg	
10596	4/29/14	M	27.0	KANYAN	Neg	Neg
10597	4/29/14	F	38.0	IZINGA	Pos	
10598	4/29/14	F	31.0	BUNYANGONI	Neg	
10599	4/29/14	M	15.0	IZINGA	Neg	
10600	4/29/14	F	7.0	KANYAN	Pos	
10601	4/29/14	F	2.5	KISANGA	Neg	
10602	4/29/14	F	2.0	MALIBA	Neg	
10603	4/29/14	F	1.9	BESENIA	Pos	
10604	4/29/14	F	14.0	MURAMBA	Pos	
10605	4/30/14	F	29.0	BIKONE	Neg	
10606	4/30/14	M	27.0	BUZIBWERA	Neg	
10607	4/30/14	F	25.0	IBANDA	Neg	
10608	4/30/14	F	47.0	IZINGA	Neg	
10609	4/30/14	M	40.0	KISANGA	Neg	
10610	4/30/14	F	55.0	BUGOYE	Neg	Neg
10611	4/30/14	M	12.0	MALIBA	Neg	
10612	4/30/14	M	42.0	KISANZI	Neg	
10613	4/30/14	M	2.0	BUGOYE	Neg	
10614	4/30/14	F	0.8	MURAMBA	Pos	
10615	4/30/14	M	13.0	BUGOYE	Pos	
10616	4/30/14	M	2.0	KYAHUNDE	Neg	
10617	4/30/14	F	28.0	BUGOYE	Neg	
10618	4/30/14	M	1.0	BUGOYE	Neg	
10619	4/30/14	M	6.0	MALIBA	Pos	
10620	4/30/14	F	10.0	MALIBA	Neg	

10621	4/30/14	F	29.0	KATOOKE	Neg	
10622	4/30/14	F	0.5	BUHUNGA	Neg	
10623	4/30/14	F	37.0	BUHUNGA	Neg	
10624	4/30/14	F	0.5	IBANDA	Neg	
10625	4/30/14	F	1.8	MURAMBA	Pos	
10626	4/30/14	M	2.3	MURAMBA	Pos	
10627	4/30/14	M	46.0	MALIBA	Pos	
10628	4/30/14	F	39.0	KISANZI	Neg	
10629	4/30/14	M	2.5	BUZIBWERA	Pos	
10630	4/30/14	F	52.0	KANYAN	Neg	
10631	4/30/14	F	18.0	IZINGA	Neg	
10632	4/30/14	M	3.0	KINYANGONGE	Neg	
10633	4/30/14	F	50.0	ISULE	Neg	
10634	4/30/14	M	2.0	BUGOYE	Neg	
10635	5/1/14	F	1.2	MURAMBA	Pos	
10636	5/2/14	F	26.0	KANYAN	Neg	
10637	5/2/14	M	16.0	KANYAN	Pos	
10638	5/2/14	M	18.0	MURAMBA	Pos	
10639	5/2/14	F	23.0	BUGOYE	Neg	
10640	5/2/14	M	0.8	IZINGA	Neg	
10641	5/2/14	F	25.0	IZINGA	Pos	
10642	5/2/14	M	72.0	KIKOKERA	Pos	
10643	5/2/14	M	8.0	KANYAN	Pos	
10644	5/2/14	F	25.0	RWAKING	Neg	Neg
10645	5/2/14	F	50.0	BUGOYE	Pos	
10646	5/2/14	F	23.0	MURAMBA	Neg	
10647	5/2/14	F	0.3	BUNYANGONI	Neg	
10648	5/2/14	M	1.5	BUNYANGONI	Neg	
10649	5/2/14	F	40.0	KANYAN		Neg
10650	5/2/14	F	2.8	MURAMBA	Pos	
10651	5/2/14	F	20.0	MURAMBA		Neg
10652	5/2/14	F	43.0	MURAMBA	Neg	
10653	5/2/14	F	10.0	NDUGHUTU	Neg	
10654	5/2/14	M	2.0	NDUGHUTU	Neg	
10655	5/2/14	F	37.0	RWAKING	Neg	
10656	5/2/14	F	0.5	MALIBA	Pos	
10657	5/2/14	M	7.0	KANYAN	Neg	
10658	5/2/14	M	0.8	MURAMBA	Neg	
10659	5/2/14	M	0.9	IZINGA	Neg	
10660	5/2/14	M	21.0	BUGOYE	Neg	
10661	5/2/14	M	30.0	MURAMBA	Neg	
10662	5/2/14	M	52.0	KATOOKE	Neg	
10663	5/2/14	F	19.0	IBANDA	Pos	

10664	5/2/14	F	1.5	MURAMBA	Neg	
10665	5/5/14	F	25.0	RWAKING	Neg	
10666	5/5/14	M	5.0	KYANYA	Pos	
10667	5/5/14	F	7.0	NYAKABUGHA	Neg	
10668	5/5/14	F	7.0	NYAKABUGHA	Neg	
10669	5/5/14	M	8.0	NYAKABUGHA	Neg	
10670	5/5/14	F	4.0	BUNYANGONI	Pos	
10671	5/5/14	M	2.0	BUNYANGONI	Pos	
10672	5/5/14	F	21.0	MURAMBA	Neg	
10673	5/5/14	F	42.0	KANYAN	Neg	
10674	5/5/14	M	55.0	BUNYANGONI	Pos	
10675	5/5/14	M	48.0	MURAMBA	Pos	Pos
10676	5/5/14	M	9.0	KANYAN	Neg	
10677	5/5/14	F	50.0	RWAKING	Pos	
10678	5/5/14	M	50.0	BUNYANGONI	Neg	
10679	5/5/14	M	2.0	KIBIRIZI	Pos	
10680	5/5/14	F	23.0	MURAMBA	Neg	
10681	5/5/14	F	0.6	NDUGHUTU	Neg	
10682	5/5/14	F	35.0	NDUGHUTU	Neg	
10683	5/5/14	F	30.0	BUNYANGONI	Neg	
10684	5/5/14	F	30.0	BUGOYE	Neg	
10685	5/5/14	F	28.0	KEMIHOKO	Neg	
10686	5/5/14	F	3.5	NYAKABUGHA	Neg	
10687	5/5/14	M	3.5	NYAKABUGHA	Neg	
10688	5/5/14	F	6.0	NYAKABUGHA	Neg	
10689	5/5/14	F	25.0	NYAKABUGHA	Neg	
10690	5/5/14	F	6.0	NYAKABUGHA	Neg	
10691	5/5/14	M	9.0	MALIBA	Pos	
10692	5/5/14	M	13.0	MURAMBA	Pos	
10693	5/5/14	F	23.0	MURAMBA	Pos	
10694	5/5/14	F	46.0	BUNYANGONI	Neg	
10695	5/5/14	F	2.3	BUGOYE	Neg	
10696	5/5/14	F	29.0	KIRULI	Neg	
10697	5/5/14	M	62.0	BUNYANGONI	Neg	
10698	5/5/14	F	3.0	KYARUGOMOKA	Pos	
10699	5/5/14	F	2.0	MURAMBA	Pos	
10700	5/5/14	F	30.0	MALIBA	Neg	
10701	5/5/14	F	32.0	MURAMBA	Pos	
10702	5/5/14	F	13.0	IZINGA	Pos	
10703	5/5/14	M	8.0	IZINGA	Pos	
10704	5/5/14	F	5.0	IZINGA	Pos	
10705	5/5/14	M	15.0	NYAKARUNGU	Neg	
10706	5/5/14	F	15.0	KANYAN	Neg	

10707	5/5/14	F	3.0	IHANI	Pos	
10708	5/6/14	M	0.9	NDUGHUTU	Neg	
10709	5/6/14	F	1.4	MAGHOMA	Neg	
10710	5/6/14	F	60.0	MURAMBA	Pos	
10711	5/6/14	F	37.0	KATOOKE	Neg	
10712	5/6/14	F	4.0	KIKOKERA	Neg	
10713	5/6/14	F	21.0	BUNYANGONI	Neg	
10714	5/6/14	M	4.0	MALIBA	Neg	
10715	5/6/14	F	30.0	RWAKING	Pos	
10716	5/6/14	M	5.0	MURAMBA	Pos	
10717	5/6/14	F	22.0	NDUGHUTU	Neg	
10718	5/6/14	F	7.0	BUGOYE	Pos	
10719	5/6/14	F	5.0	BUGOYE	Neg	
10720	5/6/14	F	4.0	BUGOYE	Neg	
10721	5/6/14	M	1.3	BUGOYE	Pos	
10722	5/6/14	M	28.0	BUGOYE	Neg	
10723	5/6/14	M	27.0	KATOOKE	Neg	
10724	5/6/14	F	12.0	MURAMBA	Pos	
10725	5/6/14	M	1.3	IZINGA	Pos	Pos
10726	5/6/14	F	0.8	RWAKING	Neg	
10727	5/6/14	F	3.5	KATOOKE	Neg	
10728	5/6/14	F	0.8	BUGOYE	Neg	
10729	5/6/14	M	12.0	MURAMBA	Pos	
10730	5/6/14	F	3.0	MURAMBA	Pos	
10731	5/6/14	F	30.0	MURAMBA	Neg	
10732	5/6/14	F	18.0	MURAMBA	Neg	Neg
10733	5/6/14	F	2.5	MUHAMBO	Neg	
10734	5/6/14	M	25.0	BUGOYE	Neg	
10735	5/6/14	M	19.0	MALIBA	Neg	Neg
10736	5/6/14	F	5.0	MURAMBA	Neg	
10737	5/6/14	F	3.0	MURAMBA	Pos	
10738	5/7/14	F	28.0	KIKOKERA	Neg	Neg
10739	5/7/14	M	5.0	BUGOYE	Neg	
10740	5/7/14	F	40.0	BUGOYE	Neg	
10741	5/7/14	F	53.0	RWAKING	Pos	
10742	5/7/14	F	18.0	NDUGHUTU	Neg	Neg
10743	5/7/14	M	4.0	KISANZI	Neg	Neg
10744	5/7/14	F	26.0	NDUGHUTU	Neg	
10745	5/7/14	M	5.0	NDUGHUTU	Neg	
10746	5/7/14	M	0.7	BUGOYE	Neg	
10747	5/7/14	F	28.0	MURAMBA	Pos	
10748	5/7/14	F	19.0	KISANZI	Neg	
10749	5/7/14	F	40.0	KYANYA	Neg	

10750	5/7/14	M	21.0	KIKOKERA	Neg	
10751	5/7/14	M	1.7	KATOOKE	Neg	
10752	5/7/14	F	25.0	BUHUNGA	Neg	
10753	5/7/14	M	14.0	KYANYA	Pos	
10754	5/7/14	F	20.0	BIKONE	Neg	
10755	5/7/14	M	0.9	RWAKING	Pos	
10756	5/7/14	F	15.0	BUGOYE	Pos	
10757	5/7/14	F	30.0	BIKONE	Neg	
10758	5/7/14	M	9.0	BIKONE	Pos	
10759	5/7/14	F	4.0	IZINGA	Pos	
10760	5/7/14	F	15.0	KAKINDO	Pos	
10761	5/7/14	F	6.0	KAKINDO	Pos	
10762	5/7/14	M	8.0	BIKONE	Pos	
10763	5/7/14	F	23.0	IBANDA	Neg	
10764	5/7/14	F	19.0	MALIBA	Pos	
10765	5/7/14	F	28.0	IBANDA	Pos	
10766	5/7/14	F	43.0	BIKONE	Neg	
10767	5/7/14	F	6.0	MURAMBA	Pos	
10768	5/7/14	F	16.0	BUGOYE	Pos	Pos
10769	5/7/14	F	45.0	MALIBA	Neg	
10770	5/7/14	F	15.0	MALIBA	Pos	
10771	5/7/14	F	31.0	BUGOYE	Neg	
10772	5/7/14	M	16.0	BUGOYE	Pos	
10773	5/7/14	M	13.0	KIBIRIZI	Pos	
10774	5/7/14	F	41.0	KISANZI	Neg	
10775	5/7/14	M	11.0	KISANZI	Neg	
10776	5/7/14	F	25.0	MUBUKU	Neg	
10777	5/7/14	F	3.0	NDUGHUTU	Pos	
10778	5/7/14	F	1.1	KYARUGOMOKA	Neg	
10779	5/7/14	F	24.0	NDUGHUTU	Neg	
10780	5/7/14	M	8.0	NDUGHUTU	Neg	
10781	5/7/14	F	4.0	BUZIBWERA	Pos	
10782	5/7/14	F	32.0	MALIBA	Pos	
10783	5/7/14	F	31.0	IZINGA	Pos	
10784	5/7/14	F	25.0	.	Neg	Neg
10785	5/7/14	F	2.0	MUHAMBO	Neg	
10786	5/7/14	F	12.0	NYAKALENGIJO	Pos	
10787	5/7/14	M	4.0	KATOOKE	Neg	
10788	5/8/14	F	30.0	MURAMBA	Neg	
10789	5/8/14	F	50.0	MURAMBA	Neg	
10790	5/8/14	F	40.0	BUGOYE	Neg	
10791	5/8/14	M	6.5	NDUGHUTU	Pos	
10792	5/8/14	F	4.5	NDUGHUTU	Pos	

10793	5/8/14	F	36.0	BUGOYE	Neg
10794	5/8/14	F	11.0	BUGOYE	Pos
10795	5/8/14	F	38.0	MALIBA	Neg
10796	5/8/14	M	12.0	MALIBA	Pos
10797	5/8/14	F	30.0	IZINGA	Neg
10798	5/8/14	F	8.5	BUGOYE	Neg
10799	5/8/14	F	3.1	MAGHOMA	Neg
10800	5/8/14	M	14.0	NYAKALENGIJO	Pos
10801	5/8/14	F	4.0	MUHAMBO	Pos
10802	5/8/14	M	4.1	NDUGHUTU	Neg
10803	5/8/14	F	0.8	MALIBA	Neg
10804	5/8/14	M	2.1	MURAMBA	Pos
10805	5/8/14	F	7.0	BUNYANGONI	Pos
10806	5/8/14	F	23.0	MURAMBA	Pos
10807	5/8/14	M	9.0	ISULE	Neg
10808	5/8/14	F	10.0	IZINGA	Pos
10809	5/8/14	F	1.0	IZINGA	Neg
10810	5/8/14	F	1.5	MURAMBA	Neg
10811	5/8/14	F	7.0	KISANZI	Pos
10812	5/8/14	F	31.0	KIBIRIZI	Neg
10813	5/8/14	M	1.2	MUBUKU	Neg
10814	5/9/14	F	12.0	MURAMBA	Neg
10815	5/9/14	F	6.0	MAGHOMA	Neg
10816	5/9/14	F	13.0	MURAMBA	Pos
10817	5/9/14	F	39.0	IZINGA	Pos
10818	5/9/14	F	22.0	BUGOYE	Neg
10819	5/9/14	F	9.0	NDUGHUTU	Neg
10820	5/9/14	M	1.3	NDUGHUTU	Pos
10821	5/9/14	M	10.0	NDUGHUTU	Neg
10822	5/9/14	M	0.5	IBANDA	Neg
10823	5/9/14	F	26.0	KANYAN	Neg
10824	5/9/14	F	35.0	ISULE	Neg
10825	5/9/14	M	11.0	IBANDA	Pos
10826	5/9/14	M	21.0	RUBONI	Neg
10827	5/9/14	F	10.0	IZINGA	Pos
10828	5/9/14	F	19.0	IBANDA	Neg
10829	5/9/14	F	12.0	MURAMBA	Pos
10830	5/9/14	M	1.3	MURAMBA	Pos
10831	5/9/14	F	30.0	MURAMBA	Pos
10832	5/12/14	F	10.0	MURAMBA	Pos
10833	5/12/14	F	49.0	IBANDA	Neg
10834	5/12/14	M	2.2	NDUGHUTU	Pos
10835	5/12/14	F	60.0	MURAMBA	Neg

10836	5/12/14	F	51.0	MURAMBA	Pos
10837	5/12/14	F	0.8	RWAKING	Pos
10838	5/12/14	F	10.0	BUNYANGONI	Neg
10839	5/12/14	F	36.0	KYANYA	Neg
10840	5/12/14	F	39.0	KYANYA	Neg
10841	5/12/14	F	8.0	MUBUKU	Pos
10842	5/12/14	F	27.0	MURAMBA	Neg
10843	5/12/14	F	11.0	IZINGA	Neg
10844	5/12/14	F	30.0	IZINGA	Pos
10845	5/12/14	M	20.0	KANYAN	Pos
10846	5/12/14	M	36.0	NDUGHUTU	Pos
10847	5/12/14	M	2.1	KATOOKE	Neg
10848	5/12/14	F	18.0	KANYAN	Neg
10849	5/12/14	F	31.0	NYANGONGE	Neg
10850	5/12/14	M	3.0	RWAKING	Neg
10851	5/12/14	F	21.0	BUGOYE	Neg
10852	5/12/14	F	0.6	MALIBA	Neg
10853	5/12/14	F	15.0	IBANDA	Neg
10854	5/12/14	F	16.0	IBANDA	Neg
10855	5/12/14	F	24.0	KANYAN	Neg
10856	5/12/14	M	4.0	BIKONE	Neg
10857	5/12/14	F	8.0	BIKONE	Neg
10858	5/12/14	F	24.0	BIKONE	Neg
10859	5/12/14	F	2.4	MALIBA	Pos
10860	5/12/14	M	9.0	MALIBA	Neg
10861	5/12/14	F	3.1	MALIBA	Pos
10862	5/12/14	F	14.0	IZINGA	Pos
10863	5/12/14	F	12.0	NDUGHUTU	Pos
10864	5/12/14	F	15.0	BUGOYE	Pos
10865	5/12/14	F	50.0	KYARUGOMOKA	Neg
10866	5/12/14	F	22.0	BIKONE	Neg
10867	5/12/14	F	25.0	BUNYANGONI	Pos
10868	5/12/14	M	3.0	NYAKABUGHA	Neg
10869	5/12/14	M	14.0	BUGOYE	Pos
10870	5/12/14	M	18.0	MALIBA	Pos
10871	5/12/14	M	38.0	NDUGHUTU	Neg
10872	5/12/14	F	48.0	BUZIBWERA	Pos
10873	5/12/14	F	30.0	NYAKALENGIJO	Pos
10874	5/12/14	F	24.0	MALIBA	Neg
10875	5/12/14	F	20.0	MALIBA	Neg
10876	5/12/14	M	29.0	MURAMBA	Pos
10877	5/12/14	F	10.0	MURAMBA	Pos
10878	5/12/14	F	40.0	BULYANDEKE	Neg

10879	5/12/14	M	43.0	BUNYANGONI	Neg
10880	5/12/14	M	7.0	BUGOYE	Pos
10881	5/12/14	F	50.0	MURAMBA	Pos
10882	5/12/14	M	21.0	KIRULI	Neg
10883	5/12/14	F	0.5	KIBOTA	Neg
10884	5/12/14	F	18.0	KANYAN	Neg
10885	5/12/14	M	3.0	NDUGHUTU	Neg
10886	5/12/14	F	17.0	IHANI	Neg
10887	5/12/14	F	6.0	NDUGHUTU	Neg
10888	5/12/14	F	8.0	MURAMBA	Pos
10889	5/13/14	M	30.0	KANYAN	Neg
10890	5/13/14	M	3.0	KANYAN	Neg
10891	5/13/14	F	25.0	BUGOYE	Neg
10892	5/13/14	F	4.0	BUGOYE	Neg
10893	5/13/14	F	16.0	NDUGHUTU	Neg
10894	5/13/14	F	27.0	BUGOYE	Neg
10895	5/13/14	F	21.0	BUGOYE	Pos
10896	5/13/14	F	40.0	KYANYA	Neg
10897	5/13/14	F	45.0	MURAMBA	Neg
10898	5/13/14	F	6.0	IZINGA	Pos
10899	5/13/14	M	30.0	NYANGONGE	Neg
10900	5/13/14	M	23.0	BUGOYE	Neg
10901	5/13/14	M	20.0	BUGOYE	Neg
10902	5/13/14	M	18.0	KATOOKE	Neg
10903	5/13/14	F	31.0	BUGOYE	Pos
10904	5/13/14	M	40.0	KISANGA	Pos
10905	5/13/14	M	24.0	NDUGHUTU	Neg
10906	5/13/14	F	54.0	MUHAMBO	Neg
10907	5/13/14	F	23.0	MURAMBA	Neg
10908	5/13/14	M	13.0	IZINGA	Pos
10909	5/13/14	M	35.0	MUBUKU	Neg
10910	5/13/14	F	6.0	MALIBA	Pos
10911	5/14/14	F	39.0	NDUGHUTU	Neg
10912	5/14/14	F	40.0	KISANZI	Pos
10913	5/14/14	F	0.4	KYANYA	Neg
10914	5/14/14	F	17.0	IBANDA	Pos
10915	5/14/14	F	8.0	IBANDA	Neg
10916	5/14/14	F	11.0	KIRULI	Neg
10917	5/14/14	F	4.0	IBANDA	Neg
10918	5/14/14	M	4.0	MUHAMBO	Neg
10919	5/14/14	F	27.0	KANYAN	Neg
10920	5/14/14	F	20.0	MALIBA	Neg
10921	5/14/14	M	30.0	KANYAN	Neg

10922	5/14/14	F	40.0	MAGHOMA	Neg
10923	5/14/14	F	19.0	BUGOYE	Neg
10924	5/14/14	F	4.0	MURAMBA	Pos
10925	5/14/14	M	1.5	BULYANDEKE	Neg
10926	5/14/14	F	19.0	NDUGHUTU	Neg
10927	5/14/14	F	30.0	KISANZI	Pos
10928	5/14/14	F	19.0	KYARUGOMOKA	Pos
10929	5/14/14	F	0.7	KYARUGOMOKA	Neg
10930	5/14/14	F	11.0	KYARUGOMOKA	Pos
10931	5/14/14	M	4.0	RWAKING	Neg
10932	5/14/14	F	6.0	BULYANDEKE	Pos
10933	5/14/14	F	55.0	IZINGA	Neg
10934	5/14/14	M	8.0	RWAKING	Pos
10935	5/14/14	F	3.0	MURAMBA	Pos
10936	5/14/14	M	1.5	MURAMBA	Pos
10937	5/14/14	F	12.0	IZINGA	Pos
10938	5/14/14	F	19.0	KIRULI	Neg
10939	5/14/14	F	18.0	MALIBA	Pos
10940	5/14/14	F	22.0	KISANZI	Neg
10941	5/14/14	F	15.0	KIBOTA	Pos
10942	5/14/14	F	0.8	KYANYA	Neg
10943	5/14/14	M	2.4	MALIBA	Neg
10944	5/14/14	F	2.0	BIKONE	Neg
10945	5/14/14	M	1.1	MALIBA	Pos
10946	5/14/14	F	45.0	BIKONE	Neg
10947	5/14/14	F	3.0	MUHAMBO	Pos
10948	5/14/14	F	3.0	IHANI	Pos
10949	5/15/14	F	18.0	NDUGHUTU	Pos
10950	5/15/14	F	0.2	BUNYANGONI	Neg
10951	5/15/14	F	30.0	BUGOYE	Pos
10952	5/15/14	F	1.8	IZINGA	Neg
10953	5/15/14	M	10.0	IZINGA	Pos
10954	5/15/14	M	42.0	BULYANDEKE	Pos
10955	5/15/14	F	1.0	BUNYANGONI	Neg
10956	5/15/14	F	18.0	BULYANDEKE	Neg
10957	5/15/14	F	33.0	IBANDA	Neg
10958	5/15/14	F	9.0	RWAKING	Neg
10959	5/15/14	F	33.0	BUNYANGONI	Neg
10960	5/15/14	F	1.1	MAGHOMA	Neg
10961	5/15/14	M	4.0	MALIBA	Pos
10962	5/15/14	M	8.0	MALIBA	Pos
10963	5/15/14	F	1.7	MUHAMBO	Neg
10964	5/15/14	F	44.0	MUHAMBO	Neg

10965	5/15/14	F	7.0	KANYAN	Neg	
10966	5/15/14	M	0.4	KANYAN	Pos	
10967	5/15/14	F	0.8	RWAKING	Neg	
10968	5/15/14	F	23.0	KATOOKE	Neg	
10969	5/16/14	F	10.0	KANYAN	Pos	
10970	5/16/14	F	20.0	MALIBA	Pos	
10971	5/16/14	M	40.0	MALIBA	Pos	
10972	5/16/14	F	20.0	RWAKING	Neg	
10973	5/16/14	F	4.5	KATOOKE	Pos	
10974	5/16/14	M	9.0	IZINGA	Pos	
10975	5/16/14	F	12.0	RWAKING	Neg	
10976	5/16/14	M	6.0	BUNYANGONI	Pos	
10977	5/16/14	M	8.0	MURAMBA	Pos	
10978	5/16/14	F	26.0	BUNYANGONI	Neg	
10979	5/16/14	F	21.0	IZINGA	Neg	
10980	5/16/14	F	30.0	BUNYANGONI	Neg	
10981	5/16/14	F	34.0	KANYAN	Neg	
10982	5/16/14	M	44.0	MALIBA	Neg	
10983	5/16/14	M	15.0	IZINGA	Neg	
10984	5/16/14	F	19.0	IHANI	Neg	
10985	5/16/14	F	5.0	BUNYANGONI	Neg	
10986	5/16/14	F	18.0	IZINGA	Pos	
10987	5/16/14	F	18.0	BUGOYE	Pos	
10988	5/16/14	M	0.7	MURAMBA	Neg	
10989	5/16/14	M	57.0	BUGOYE	Pos	
10990	5/19/14	F	23.0	MURAMBA	Pos	
10991	5/19/14	F	1.5	IZINGA	Pos	
10992	5/19/14	F	40.0	MURAMBA	Neg	Neg
10993	5/19/14	M	2.5	NDUGHUTU	Neg	
10994	5/19/14	M	10.0	MURAMBA	Pos	
10995	5/19/14	F	20.0	MALIBA	Pos	
10996	5/19/14	M	5.0	MURAMBA	Pos	
10997	5/19/14	M	2.3	MAGHOMA	Neg	
10998	5/19/14	F	12.0	KAKINDO	Pos	
10999	5/19/14	F	18.0	KYANYA	Neg	
11000	5/19/14	F	59.0	BUNYANGONI	Neg	
11001	5/19/14	F	18.0	KANYAN	Neg	
11002	5/19/14	M	1.5	BUGOYE	Neg	
11003	5/19/14	F	17.0	MURAMBA		Pos
11004	5/19/14	F	48.0	KIRULI	Neg	
11005	5/19/14	F	53.0	BUNYANGONI	Neg	
11006	5/19/14	F	7.0	KATUMBA	Pos	
11007	5/19/14	F	18.0	MURAMBA	Pos	

11008	5/19/14	M	34.0	KISANGA	Pos	
11009	5/19/14	F	0.5	IZINGA	Neg	
11010	5/20/14	F	30.0	KIBIRIZI	Neg	Neg
11011	5/20/14	F	1.0	KANYAN	Neg	
11012	5/20/14	M	5.0	IZINGA	Pos	
11013	5/20/14	F	26.0	MUKATI	Neg	
11014	5/20/14	M	6.0	MURAMBA	Pos	
11015	5/20/14	F	21.0	IZINGA	Pos	
11016	5/20/14	F	1.8	MUHAMBO	Pos	
11017	5/20/14	F	30.0	KANYAN	Pos	
11018	5/20/14	F	43.0	MUKATI	Neg	
11019	5/20/14	M	25.0	MURAMBA	Neg	
11020	5/20/14	M	50.0	KIRULI	Neg	
11021	5/20/14	M	2.0	IZINGA	Neg	
11022	5/20/14	F	28.0	BUZIBWERA	Neg	
11023	5/20/14	F	40.0	IZINGA	Neg	
11024	5/20/14	M	5.5	BUZIBWERA	Pos	
11025	5/20/14	F	33.0	MALIBA	Neg	
11026	5/20/14	F	5.0	KIBIRIZI	Pos	
11027	5/20/14	F	58.0	RWAKING	Neg	
11028	5/20/14	F	44.0	KANYAN	Pos	
11029	5/20/14	M	48.0	MURAMBA	Pos	
11030	5/20/14	F	25.0	NYAKABUGHA	Neg	
11031	5/20/14	F	60.0	KYANYA	Neg	
11032	5/20/14	F	5.0	NYAKABUGHA	Neg	
11033	5/20/14	F	45.0	KYANYA	Neg	
11034	5/20/14	F	20.0	NDUGHUTU	Neg	
11035	5/20/14	F	80.0	BUGOYE	Neg	
11036	5/20/14	F	11.0	BUNYANGONI	Pos	
11037	5/20/14	F	38.0	BUNYANGONI	Neg	
11038	5/20/14	M	54.0	KYANYA	Neg	
11039	5/20/14	F	20.0	KYANYA	Pos	
11040	5/20/14	F	19.0	KATUMBA	Pos	
11041	5/20/14	F	9.0	BUGOYE	Neg	
11042	5/20/14	F	23.0	IHANI	Neg	
11043	5/20/14	F	20.0	MALIBA	Neg	
11044	5/20/14	M	25.0	RWAKING	Pos	
11045	5/20/14	M	0.5	MALIBA	Pos	
11046	5/21/14	F	5.0	RWAKING	Neg	
11047	5/21/14	M	18.0	RWAKING	Pos	
11048	5/21/14	F	55.0	BUGOYE	Pos	
11049	5/21/14	F	27.0	MAGHOMA	Neg	Neg
11050	5/21/14	F	14.0	MURAMBA	Pos	

11051	5/21/14	F	11.0	MURAMBA	Pos	
11052	5/21/14	F	0.6	KIHINDI	Neg	
11053	5/21/14	F	0.4	KATUMBA	Neg	
11054	5/21/14	F	5.0	IBANDA	Pos	
11055	5/21/14	M	0.8	BUNYANGONI	Neg	
11056	5/21/14	F	18.0	IZINGA	Pos	
11057	5/21/14	F	20.0	BUGOYE	Neg	
11058	5/21/14	F	17.0	MURAMBA	Neg	
11059	5/21/14	F	20.0	BUGOYE	Neg	
11060	5/21/14	F	26.0	KANYAN	Neg	
11061	5/21/14	M	32.0	KIRONGO	Neg	
11062	5/21/14	M	1.3	KATUMBA	Neg	
11063	5/21/14	F	0.7	MAGHOMA	Neg	
11064	5/21/14	F	38.0	KAKINDO	Neg	
11065	5/21/14	M	0.3	KAKINDO	Neg	
11066	5/21/14	F	39.0	NYABUSISI	Neg	
11067	5/21/14	F	22.0	KYANDALE	Neg	
11068	5/21/14	F	34.0	IZINGA	Neg	
11069	5/21/14	F	8.0	IZINGA	Neg	
11070	5/21/14	M	8.0	IZINGA	Neg	
11071	5/21/14	F	2.0	BUGOYE	Pos	
11072	5/21/14	M	0.8	MURAMBA	Pos	
11073	5/21/14	F	49.0	RWAKING	Neg	
11074	5/21/14	F	25.0	KATUMBA	Neg	
11075	5/21/14	M	1.5	MAGHOMA	Neg	Neg
11076	5/21/14	F	28.0	KIBIRIZI	Neg	
11077	5/21/14	F	0.8	KATUMBA	Neg	
11078	5/21/14	M	8.0	BUGOYE	Pos	
11079	5/21/14	F	54.0	KYARUGOMOKA	Neg	
11080	5/21/14	F	55.0	KYANYA	Neg	
11081	5/21/14	M	68.0	BUGOYE	Neg	
11082	5/21/14	F	4.5	BUGOYE	Neg	
11083	5/22/14	F	3.0	KAKINDO	Pos	
11084	5/22/14	F	18.0	IZINGA	Neg	
11085	5/22/14	F	20.0	NYAKARUNGU	Neg	
11086	5/22/14	M	6.0	MUHAMBO	Neg	
11087	5/22/14	M	20.0	BIKONE	Pos	
11088	5/22/14	M	16.0	IZINGA	Pos	
11089	5/22/14	F	2.5	MURAMBA	Pos	
11090	5/22/14	F	9.0	MURAMBA	Pos	
11091	5/22/14	M	8.0	MURAMBA	Pos	
11092	5/22/14	M	7.0	KATOOKE	Pos	
11093	5/22/14	M	4.0	BUGOYE	Neg	

11094	5/22/14	F	45.0	MURAMBA	Neg
11095	5/22/14	F	41.0	MUHAMBO	Neg
11096	5/22/14	F	3.5	MUHAMBO	Neg
11097	5/22/14	M	1.2	MUHAMBO	Neg
11098	5/22/14	F	2.1	RWAKING	Neg
11099	5/22/14	M	55.0	BUHUNGA	Neg
11100	5/22/14	F	40.0	BIKONE	Neg
11101	5/22/14	F	68.0	BUGOYE	Neg
11102	5/22/14	M	32.0	MALIBA	Neg
11103	5/22/14	F	48.0	BUHUNGA	Neg
11104	5/22/14	F	50.0	KANYAN	Neg
11105	5/22/14	F	21.0	BUGOYE	Neg
11106	5/22/14	M	28.0	MURAMBA	Neg
11107	5/22/14	M	33.0	MUHAMBO	Neg
11108	5/22/14	M	12.0	MURAMBA	Pos
11109	5/22/14	F	79.0	KYARUGOMOKA	Neg
11110	5/22/14	M	7.0	KYANYA	Neg
11111	5/22/14	F	2.8	KYANYA	Neg
11112	5/22/14	M	4.5	KYANYA	Neg
11113	5/22/14	F	36.0	KYANYA	Neg
11114	5/22/14	F	0.0	KYANYA	Neg
11115	5/22/14	M	24.0	MALIBA	Neg
11116	5/22/14	M	37.0	KYANYA	Neg
11117	5/22/14	F	42.0	IHANI	Neg
11118	5/22/14	F	15.0	BIKONE	Neg
11119	5/22/14	F	32.0	IBANDA	Neg
11120	5/22/14	F	3.0	NDUGHUTU	Pos
11121	5/22/14	M	41.0	RWAKING	Neg
11122	5/22/14	M	13.0	MALIBA	Neg
11123	5/23/14	F	12.0	KARUSANKERA	Pos
11124	5/23/14	M	6.0	MALIBA	Neg
11125	5/23/14	M	11.0	MURAMBA	Pos
11126	5/23/14	F	23.0	KANYAN	Pos
11127	5/23/14	M	10.0	NYAKALENGIJO	Pos
11128	5/23/14	F	3.0	BUZIBWERA	Pos
11129	5/23/14	F	30.0	MALIBA	Neg
11130	5/23/14	F	9.0	MALIBA	Neg
11131	5/23/14	F	32.0	KIRULI	Neg
11132	5/23/14	F	28.0	BUGOYE	Pos
11133	5/23/14	M	9.0	RWAKING	Pos
11134	5/23/14	F	23.0	IZINGA	Neg
11135	5/23/14	F	1.0	IZINGA	Neg
11136	5/23/14	F	51.0	BUGOYE	Pos

11137	5/23/14	M	1.5	MALIBA	Pos
11138	5/23/14	F	30.0	KYARUGOMOKA	Neg
11139	5/23/14	F	24.0	KANYAN	Neg
11140	5/23/14	M	2.0	KIRULI	Neg
11141	5/23/14	M	1.2	IHANI	Neg
11142	5/23/14	M	3.5	NDUGHUTU	Neg
11143	5/23/14	F	0.9	NDUGHUTU	Neg
11144	5/23/14	M	35.0	KAKINDO	Neg
11145	5/23/14	F	2.5	RWAKING	Neg
11146	5/23/14	F	19.0	MALIBA	Neg
11147	5/23/14	M	4.0	IBANDA	Neg
11148	5/23/14	F	22.0	IBANDA	Neg
11149	5/23/14	M	0.3	IZINGA	Neg
11150	5/23/14	F	12.0	IZINGA	Pos
11151	5/26/14	M	6.0	BUGOYE	Pos
11152	5/26/14	M	0.6	NDUGHUTU	Pos
11153	5/26/14	M	1.4	BUNYANGONI	Pos
11154	5/26/14	F	30.0	NYABISUSI	Neg
11155	5/26/14	F	35.0	NDUGHUTU	Neg
11156	5/26/14	F	5.0	MURAMBA	Pos
11157	5/26/14	F	9.0	BUGOYE	Pos
11158	5/26/14	M	1.4	BUGOYE	Pos
11159	5/26/14	M	3.0	BUGOYE	Neg
11160	5/26/14	M	14.0	KANYAN	Neg
11161	5/26/14	F	30.0	BUNYANGONI	Pos
11162	5/26/14	M	9.0	BUNYANGONI	Pos
11163	5/26/14	M	18.0	KYANDALE	Pos
11164	5/26/14	M	22.0	NDUGHUTU	Neg
11165	5/26/14	F	40.0	MUBUKU	Neg
11166	5/26/14	F	42.0	KIKOKERA	Neg
11167	5/26/14	F	11.0	MALIBA	Pos
11168	5/26/14	F	12.0	KIRULI	Neg
11169	5/26/14	F	17.0	IHANI	Neg
11170	5/26/14	F	28.0	KAKINDO	Neg
11171	5/26/14	F	14.0	KANYAN	Neg
11172	5/26/14	F	5.0	KIKOKERA	Pos
11173	5/26/14	F	20.0	KIKOKERA	Neg
11174	5/26/14	M	1.0	KIRONGO	Neg
11175	5/26/14	F	20.0	KIBOTA	Neg
11176	5/26/14	F	6.0	BUHUNGA	Pos
11177	5/26/14	F	4.5	KANYAN	Neg
11178	5/26/14	M	20.0	MURAMBA	Neg
11179	5/26/14	F	1.3	RWAKING	Pos

11180	5/26/14	F	18.0	NDUGHUTU	Neg
11181	5/26/14	M	19.0	KANYAN	Neg
11182	5/26/14	M	5.5	IHANI	Pos
11183	5/26/14	F	21.0	BUHUNGA	Neg
11184	5/26/14	F	1.3	BUHUNGA	Pos
11185	5/26/14	F	22.0	KANYAN	Pos
11186	5/26/14	F	32.0	RWAKING	Neg
11187	5/26/14	M	38.0	IZINGA	Neg
11188	5/26/14	M	48.0	MURAMBA	Neg
11189	5/26/14	M	7.0	IZINGA	Pos
11190	5/26/14	F	24.0	MALIBA	Neg
11191	5/26/14	M	4.0	KIBIRIZI	Neg
11192	5/26/14	F	41.0	MALIBA	Neg
11193	5/26/14	M	11.0	BUGOYE	Pos
11194	5/26/14	M	13.0	MURAMBA	Neg
11195	5/26/14	F	33.0	MURAMBA	Neg
11196	5/26/14	F	0.3	BUGOYE	Neg
11197	5/26/14	F	7.0	MURAMBA	Neg
11198	5/26/14	F	30.0	KISAMBA	Neg
11199	5/26/14	F	0.3	KISANZI	Neg
11200	5/26/14	M	23.0	BUGOYE	Neg
11201	5/26/14	F	7.0	IZINGA	Pos
11202	5/26/14	F	6.0	IBANDA	Neg
11203	5/26/14	F	24.0	KIBIRIZI	Neg
11204	5/26/14	F	30.0	KATOOKE	Pos
11205	5/26/14	F	20.0	MALIBA	Neg
11206	5/26/14	M	1.0	KIHINDI	Neg
11207	5/26/14	F	0.3	KIHINDI	Neg
11208	5/26/14	F	14.0	BUGOYE	Pos
11209	5/26/14	M	0.1	IZINGA	Neg
11210	5/26/14	M	75.0	KYANYA	Neg
11211	5/26/14	F	24.0	MURAMBA	Pos
11212	5/26/14	M	2.3	NYAKARUNGU	Pos
11213	5/26/14	F	23.0	NYAKARUNGU	Neg
11214	5/26/14	F	33.0	IZINGA	Neg
11215	5/26/14	F	50.0	MURAMBA	Neg
11216	5/26/14	F	40.0	NYAKARUNGU	Neg
11217	5/26/14	F	16.0	RWAKING	Neg
11218	5/26/14	F	63.0	BUGOYE	Neg
11219	5/26/14	F	10.0	MALIBA	Pos
11220	5/26/14	F	8.0	MALIBA	Pos
11221	5/26/14	F	0.1	HIMA	Neg
11222	5/27/14	M	2.0	IZINGA	Pos

11223	5/27/14	F	50.0	BUGOYE	Neg	
11224	5/27/14	M	6.0	BUGOYE	Neg	
11225	5/27/14	F	10.0	IZINGA	Pos	
11226	5/27/14	M	5.0	IZINGA	Neg	
11227	5/27/14	F	40.0	BUGOYE	Neg	
11228	5/27/14	F	43.0	MUHAMBO	Neg	
11229	5/27/14	F	0.7	MUHAMBO	Neg	
11230	5/27/14	M	18.0	MURAMBA	Pos	
11231	5/27/14	F	32.0	MUHAMBO	Neg	
11232	5/27/14	F	4.0	MUHAMBO	Neg	
11233	5/27/14	M	3.0	BUNYANGONI	Neg	
11234	5/27/14	F	16.0	BUGOYE	Neg	
11235	5/27/14	M	2.0	MALIBA	Neg	
11236	5/27/14	F	7.0	MUBUKU	Pos	
11237	5/27/14	F	35.0	BUHUNGA	Neg	
11238	5/27/14	F	4.0	IZINGA	Neg	
11239	5/27/14	M	43.0	IZINGA	Neg	
11240	5/27/14	M	6.0	MALIBA	Pos	
11241	5/27/14	M	0.6	MUHAMBO	Neg	
11242	5/27/14	F	9.0	IBANDA	Pos	
11243	5/27/14	M	45.0	MIHUNGU	Pos	
11244	5/27/14	M	37.0	MALIBA	Pos	
11245	5/27/14	F	40.0	BUNYANGONI	Neg	
11246	5/27/14	M	13.0	MALIBA	Pos	
11247	5/27/14	F	15.0	MALIBA	Neg	
11248	5/27/14	M	13.0	KISANZI	Neg	
11249	5/27/14	F	22.0	NDUGHUTU	Neg	
11250	5/27/14	M	0.7	MURAMBA	Pos	
11251	5/27/14	M	0.7	KIHINDI	Neg	
11252	5/27/14	M	40.0	BIKONE	Neg	
11253	5/27/14	F	32.0	BIKONE	Neg	
11254	5/27/14	F	11.0	MURAMBA	Neg	
11255	5/28/14	F	9.0	MUHAMBO	Neg	Neg
11256	5/28/14	F	30.0	KIKOKERA		Neg
11257	5/28/14	F	3.5	MUBUKU	Neg	
11258	5/28/14	F	40.0	MUBUKU	Neg	
11259	5/28/14	F	30.0	IBANDA	Neg	
11260	5/28/14	F	4.0	MURAMBA	Neg	
11261	5/28/14	F	35.0	MURAMBA	Neg	
11262	5/28/14	M	12.0	MURAMBA	Neg	
11263	5/28/14	F	40.0	MUHAMBO	Neg	
11264	5/28/14	F	7.0	BUGOYE	Pos	
11265	5/28/14	F	50.0	BUGOYE	Neg	

11266	5/28/14	M	9.0	MALIBA	Pos
11267	5/28/14	M	78.0	IBANDA	Neg
11268	5/28/14	M	1.2	NDUGHUTU	Pos
11269	5/28/14	F	10.0	IBANDA	Pos
11270	5/28/14	M	16.0	IBANDA	Neg
11271	5/28/14	M	47.0	IBANDA	Pos
11272	5/28/14	F	12.0	MURAMBA	Pos
11273	5/28/14	M	1.4	BUGOYE	Pos
11274	5/28/14	F	50.0	MALIBA	Pos
11275	5/28/14	M	56.0	MALIBA	Neg
11276	5/28/14	M	14.0	IZINGA	Neg
11277	5/28/14	M	13.0	MURAMBA	Pos
11278	5/28/14	F	0.8	MALIBA	Neg
11279	5/28/14	F	15.0	IZINGA	Pos
11280	5/28/14	F	12.0	IZINGA	Neg
11281	5/28/14	F	19.0	NYAKARUNGU	Neg
11282	5/28/14	F	0.3	KYARUGOMOKA	Neg
11283	5/28/14	F	27.0	NYAKALENGIJO	Pos
11284	5/28/14	M	8.0	MUHAMBO	Neg
11285	5/28/14	F	18.0	KYARUGOMOKA	Neg
11286	5/28/14	M	1.3	IBANDA	Neg
11287	5/28/14	M	39.0	KISANGA	Pos
11288	5/28/14	M	45.0	IZINGA	Pos
11289	5/28/14	M	14.0	MUBUKU	Pos
11290	5/28/14	F	9.0	KIBIRIZI	Neg
11291	5/28/14	F	11.0	BUGOYE	Neg
11292	5/28/14	F	21.0	BUGOYE	Pos
11293	5/28/14	M	13.0	IZINGA	Neg
11294	5/28/14	F	15.0	KANYAN	Pos
11295	5/28/14	F	25.0	MUBUKU	Neg
11296	5/28/14	F	8.0	BUGOYE	Neg
11297	5/29/14	F	16.0	BUGOYE	Neg
11298	5/29/14	F	4.5	MURAMBA	Pos
11299	5/29/14	F	25.0	MURAMBA	Neg
11300	5/29/14	F	12.0	KIRONGO	Neg
11301	5/29/14	F	45.0	IZINGA	Pos
11302	5/29/14	F	2.3	KIRONGO	Neg
11303	5/29/14	F	25.0	KANYAN	Pos
11304	5/29/14	F	43.0	BUNYANGONI	Pos
11305	5/29/14	F	2.5	BUNYANGONI	Pos
11306	5/29/14	F	60.0	MALIBA	Neg
11307	5/29/14	M	0.1	IZINGA	Neg
11308	5/29/14	M	7.0	BUHUNGA	Neg

11309	5/29/14	M	30.0	BUHUNGA	Neg
11310	5/29/14	M	6.5	NYAKABUGHA	Pos
11311	5/29/14	F	54.0	KISANGA	Neg
11312	5/29/14	F	7.0	BUGOYE	Neg
11313	5/29/14	M	1.0	MALIBA	Neg
11314	5/29/14	F	2.5	KIRULI	Pos
11315	5/29/14	F	28.0	NDUGHUTU	Pos
11316	5/29/14	F	9.0	BUGOYE	Pos
11317	5/29/14	M	45.0	BUGOYE	Neg
11318	5/29/14	F	0.0	MURAMBA	Neg
11319	5/29/14	F	2.6	MAGHOMA	Neg
11320	5/29/14	M	17.0	IZINGA	Pos
11321	5/29/14	F	24.0	IZINGA	Pos
11322	5/29/14	F	24.0	BUGOYE	Neg
11323	5/29/14	F	30.0	NDUGHUTU	Neg
11324	5/29/14	F	30.0	NDUGHUTU	Neg
11325	5/29/14	F	35.0	MALIBA	Neg
11326	5/29/14	M	1.1	NDUGHUTU	Neg
11327	5/29/14	M	30.0	MURAMBA	Neg
11328	5/29/14	F	3.8	NDUGHUTU	Neg
11329	5/29/14	F	30.0	NDUGHUTU	Neg
11330	5/29/14	M	45.0	BUGOYE	Pos
11331	5/30/14	F	16.0	BUGOYE	Neg
11332	5/30/14	M	14.0	MALIBA	Neg
11333	5/30/14	M	16.0	IZINGA	Neg
11334	5/30/14	M	10.0	MALIBA	Neg
11335	5/30/14	M	50.0	IZINGA	Neg
11336	5/30/14	F	21.0	IZINGA	Pos
11337	5/30/14	M	2.0	NDUGHUTU	Neg
11338	5/30/14	M	4.0	KYANYA	Neg
11339	5/30/14	M	1.9	KYANYA	Neg
11340	5/30/14	F	21.0	MUHAMBO	Neg
11341	5/30/14	M	6.0	MURAMBA	Pos
11342	5/30/14	M	15.0	MALIBA	Neg
11343	5/30/14	F	6.0	MURAMBA	Pos
11344	5/30/14	F	10.0	MURAMBA	Pos
11345	5/30/14	M	1.8	MALIBA	Pos
11346	5/30/14	M	14.0	MURAMBA	Pos
11347	5/30/14	F	4.0	NDUGHUTU	Pos
11348	5/30/14	F	9.0	BUGOYE	Pos
11349	5/30/14	F	5.0	NDUGHUTU	Neg
11350	5/30/14	F	0.3	NDUGHUTU	Neg
11351	5/30/14	F	18.0	MAGHOMA	Neg

11352	5/30/14	F	17.0	IHANI	Neg
11353	5/30/14	F	23.0	IBANDA	Neg
11354	5/30/14	F	28.0	BUGOYE	Neg
11355	5/30/14	M	1.0	BUGOYE	Neg
11356	5/30/14	F	1.0	BUGOYE	Neg
11357	5/30/14	F	13.0	BUGOYE	Neg
11358	5/30/14	M	21.0	KANYAN	Neg
11359	5/30/14	M	19.0	RWAKING	Neg
11360	5/30/14	F	16.0	BUGOYE	Neg
11361	5/30/14	F	19.0	IBANDA	Neg
